# Convergent synthesis of phosphorodiamidate morpholino oligonucleotides (PMOs) by the *H*-phosphonate approach

**DOI:** 10.1038/s41598-023-38698-2

**Published:** 2023-08-03

**Authors:** Taiki Tsurusaki, Kazuki Sato, Hiroki Imai, Kunihiro Hirai, Daisuke Takahashi, Takeshi Wada

**Affiliations:** 1 Department of Medicinal and Life Sciences, Faculty of Pharmaceutical Sciences, Tokyo University of Science, 2641 Yamazaki, Noda, Chiba 278-8510 Japan; 2 Research Institute for Bioscience Products and Fine Chemicals, Ajinomoto Co., Inc., 1-1, Suzuki-Cho, Kawasaki, Kanagawa 210-8681 Japan

**Keywords:** Chemistry, Organic chemistry, Synthetic chemistry methodology

## Abstract

Phosphorodiamidate morpholino oligonucleotides (PMOs) are a promising type of antisense oligonucleotides, but their challenging synthesis makes them difficult to access. This research presents an efficient synthetic approach for PMOs using the *H*-phosphonate approach. The use of phosphonium-type condensing reagents significantly reduced coupling times compared with the current synthetic approach. Furthermore, phosphonium-type condensing reagents facilitated the fragment condensation of PMO, synthesizing up to 8-mer containing all four nucleobases with remarkable coupling efficacy. This is the first report on the convergent synthesis of PMOs. This approach would facilitate the large-scale synthesis of PMOs and accelerate their popularity and accessibility as a next-generation therapy.

## Introduction

**Figure 1:**
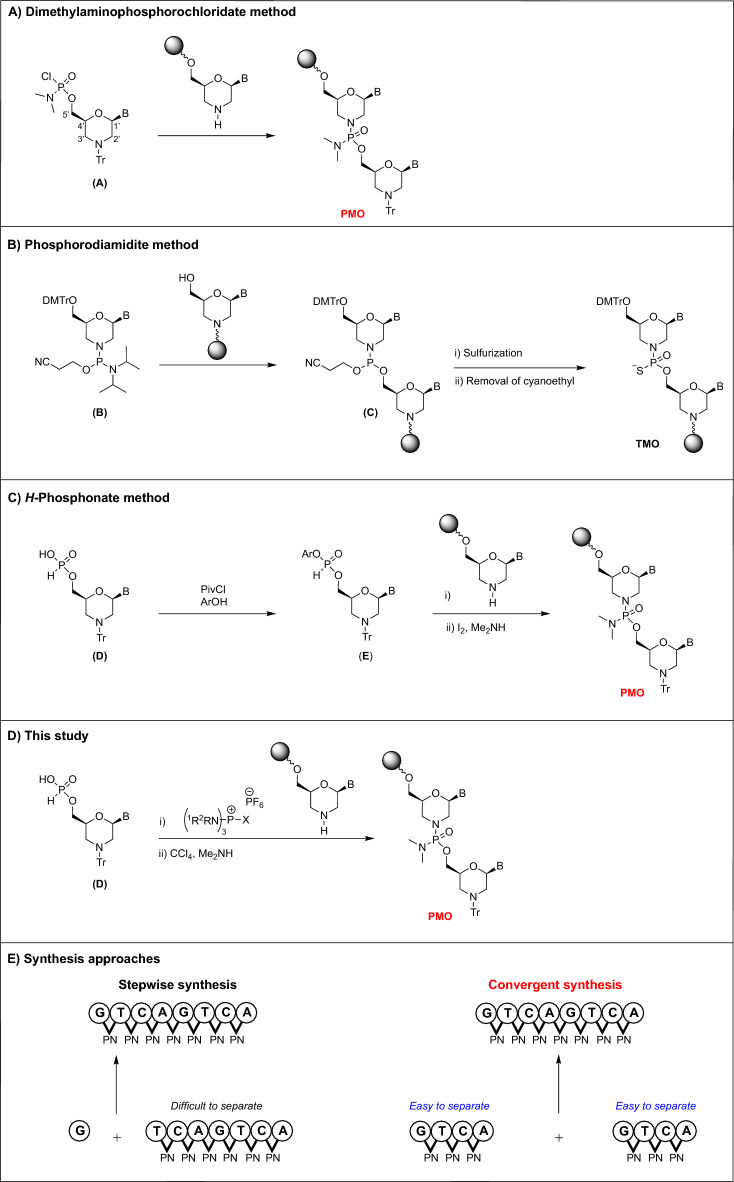
Current approaches for the synthesis of PMO derivatives.

The antisense approach has attracted attention from researchers as a potential future therapy for controlling the expression of disease-related genes^1–3^. Phosphorodiamidate morpholino oligonucleotides (PMOs), with nonionic internucleotidic linkages and a morpholino backbone, are considered promising antisense drugs^4,5^. The high binding affinity to target mRNA^4^, sequence specificity^6,7^, solubility in water^6^, and low toxicity of PMO^7–10^ have been confirmed in previous studies. To date, four antisense drugs for Duchenne muscular dystrophy have been approved (Exondys 51®^11^, Vyondys 53®^12^, Viltepso®^13^, Amondys 45®^14^) and more PMO drugs are expected to be approved in the future. Additionally, a new PMO analog called thiophosphoramidate morpholino oligonucleotides (TMOs) was synthesized and showed potential as a novel drug candidate in antisense therapy^15^. Rapid advances in PMOs have increased the requirement for developing efficient approaches for synthesizing of PMOs and PMOs analogs. Particularly, the synthetic approach, which can synthesize PMOs on a large-scale at once, is required for the availability of antisense therapy to patients. Until date, many synthetic approaches have been developed. The first synthetic approach for PMOs was reported by Summerton and Weller^5^ (Fig. 1-A). In this approach, morpholino nucleosides bearing a *N,N*-dimethylaminochlorophosphoramidate moiety on the 5ʹ-hydroxy group and trityl (Tr) group on the amino group were employed as monomer units (**A**). PMOs were synthesized by repeating the condensation of the 5ʹ-*N,N*-dimethylaminochlorophosphoramidate group of monomer (**A**) with the amino group at the 3ʹ-end of oligomer after removal of the Tr group at the 3'-end^5^. This approach is widely used but has several recognized issues, including low reactivity of monomers and long reaction times for completing the condensation reaction. In 2012, Harakawa et al. discovered that adding LiBr to a reaction mixture was effective and resulted in considerable acceleration of the reaction (ca. tenfold improvement)^16^. Additionally, Li et al.^17^ found that by increasing the reaction temperature to 90 °C, the condensation reaction was completed within 8 min, allowing them to synthesize an 18mer-PMO in just 3.5 h using a flow reactor. Kundu et al.^18^ reported the synthesis of PMO using automated oligo synthesizer. In this report, a solution-phase synthesis of 3-mer PMO was achieved using the Fmoc chemistry. Moreover, 30-mer PMOs were synthesized by a DNA synthesizer.

Recently, Langner et al.^15^ reported a novel synthetic approach for synthesizing of TMOs using phosphorodiamidite derivatives as monomers, (Fig. 1-B). In contrast to PMO, TMOs have phosphorothioamidate linkage, which showed promising properties as antisense oligonucleotides. TMOs showed high RNA binding affinity and high nuclease stability. Moreover, chimeric TMOs exhibited their potential as microRNA inhibitors. As shown in Fig. 1-B, this approach employs morpholino phosphorodiamidite derivatives bearing a 4,4ʹ-dimethoxytrithyl (DMTr) group on the 5ʹ-hydroxy group (**B**) as the monomer units. The synthesis process involved the condensation of a morpholino phosphorodiamidite monomer (**B**) with the hydroxy group at the 5ʹ-end of oligomer in the presence of an acidic activator, such as 5-ethylthio-1*H*-tetrazole (ETT), followed by sulfurization of a phosphoramidite intermediate (**C**). Subsequent detritylation followed by repetitive synthesis cycles produced TMOs. The reported coupling yield was 95–97%, with a condensation reaction time of 5 min^15^. Although this approach is effective for synthesizing TMOs, it is not applicable to the synthesis of PMOs. This method gave the phsphoramidite derivative (**C**) as an intermediate, but from this intermediate, the desired phosphorodiamidate linkage of PMO is not obtained.

In 2015, Bhadra et al.^19^ developed a new approach for synthesizing polythymidine PMOs employing *H*-phosphonate chemistry (Fig. 1-C). In this approach, 5ʹ-*H*-phosphonate monoester morpholino nucleosides (**D**) were employed as the monomer units. The 5ʹ-*H*-phosphonate monomers (**D**) were converted to aryl-*H-*phosphonate intermediates (**E**) employing a phenol derivative and pivaloyl chloride as a condensing reagent. Then, aryl-*H-*phosphonate intermediates (**E**) were condensed with the amino group at the 3ʹ-end of an oligomer. Subsequent detritylation followed by repetitive synthesis cycles led to morpholino oligomers having internucleotidic *H*-phosphonamidate linkages. Finally, internucleotidic *H*-phosphonamidate linkages were converted to phosphorodiamidate counterparts by treating with iodine and dimethylamine to generate PMOs. This approach achieved a high coupling yield (98–100%) with a short condensation reaction time (10 min)^19^. However, this approach required the preactivation of *H*-phosphonate monomers (**D**), making procedures more complex.

Previous studies have thoroughly investigated the synthesis of *H*-phosphonamidate^20–23^. These studies showed that a specific amine could be directly condensed with an *H-*phosphonate monoester using a specific condensing reagent^20–23^. Based on these findings, we recently synthesized an *H*-phosphonamidate having a morpholino group, by directly condensing an *H*-phosphonate monoester with morpholine using BOPCl as a condensing reagent^24^. Therefore, we attempted to directly condense *H*-phosphonate monomer of morpholino nucleoside with the amino group of 3ʹ-morpholino nucleoside using a specific condensing reagent (Fig. 1-D).

In most PMO syntheses, a linear synthetic method, where a monomer unit is coupled with an elongating oligomer, is commonly used (Fig. 1-E)^5,17,19^. This method results in the formation of N-1 mer, which is one base shorter than the product. The separation of N-1 mer tends to be difficult due to the similar lipophilicity between the desired product and N-1 mer. To overcome this issue, fragment condensation is effective as it avoids the formation of N-1 mer by condensing two oligomers together. Therefore, the separation of the product from the unreacted fragments would be simplified since there is a significant difference in lipophilicity between them. Furthermore, the fragment condensation approach reduces the reaction steps required for synthesizing oligomers. In oligodeoxynucleotide synthesis, the utility of the convergent synthetic method for manufacturing was demonstrated^25^. However, due to the low efficiency of the internucleotidic bond formation reaction of the existing approach, the fragment condensation of PMOs has not yet been investigated.

In this research, we developed an efficient synthetic approach for PMOs using *H*-phosphonate monoester derivatives as monomer units. The phosphonium-type condensing reagents provided the direct formation of an *H*-phosphonamidate linkage, and this approach enabled the fragment condensation.

## Results and discussion

### Synthesis of *H*-phosphonamidate 2-mers (C_PH_C)

First, 5ʹ-*O*-*tert*-butyldiphenylsilyl (TBDPS)-morpholino nucleosides **1** and *H*-phosphonate monomers **2** were synthesized from 5ʹ-hydroxy-*N*-trityl (Tr)-morpholino nucleosides of *N*^4^-benzoylcytidine (mC^bz^), *N*^6^-benzoyladenosine (mA^bz^), *N*^2^-isobutyryl-*O*^6^-cyanoethylguanosine (mG^*i*-bu,CE^), and thymidine (mT) (See SI). Next, a dimorpholinocytidine *H-*phosphonamidate derivative **3cc** was synthesized employing the obtained *H-*phosphonate monomer **2c** and morpholino nucleoside **1c**. The 1.2 equivalents of *H*-phosphonate monomer **2c** were condensed with the amino group of **1c** employing 3.0 equivalents of various condensing reagents in CD_3_CN–pyridine (1:1, v/v) at 0 °C for 20 min (Table 1). The conversion rate was determined by ^31^P nuclear magnetic resonance (NMR) analysis of the reaction mixture at room temperature. The condensation reaction using pivaloyl chloride (PivCl), which is commonly employed for the condensation reaction of DNA synthesis by the *H*-phosphonate approach, failed to produce the desired product (entry 1). We hypothesized that the amino group of morpholino nucleoside **1c** was reacted with PivCl and/or an acyl-*H*-phosphonate intermediate and capped as an amide derivative. Sobkowska et al.^20^ had previously confirmed a similar side reaction for synthesizing *H*-phosphonamidate derivatives using PivCl as a condensing reagent. Therefore, the result of entry 1 was in agreement with the previous report. However, using condensing reagents such as bis(2-oxo-3-oxazolidinyl)phosphinic chloride (BOPCl) (entry 2), benzotriazol-1-yloxy- tris(pyrrolidin-1-yl) phosphonium hexafluorophosphate (PyBOP) (entry 3), 2-(benzotriazol-1-yloxy)-1,3-dimethyl-2-pyrrolidin-1-yl-1,3,2-diazaphospholidinium hexafluorophosphate (BOMP)^26^ (entry 4), 3-nitro1,2,4-triazol-1-yl-tris(pyrrolidin-1-yl) phosphonium hexafluorophosphate (PyNTP)^27^ (entry 5), and 1,3-dimethyl-2-(3-nitro-1,2,4-triazol-1-yl)-2-pyrrolidin-1-yl-1,3,2-diazaphospholidinium hexafluorophosphate (MNTP)^27^ (entry 6) provided the *H*-phosphonamidate 2-mer **3cc** as the primary product. The formation of **3cc** was verified using ^31^P NMR (δ 13.3, 14.2 ppm, ^1^*J*_PH_ = 661.1, 654.6 Hz). Particularly, BOPCl, BOMP, and PyNTP gave **3cc** with over 98% NMR yields. BOMP, PyNTP, and MNTP are condensing reagents that we designed and synthesized for the condensation reactions of the *H*-phosphonate approach in a previous study^26,27^. These condensing reagents have a high reactivity as condensing reagents, whereas they are less reactive to the amino group^26,27^. We assumed that phosphonium-type condensing reagents gave preferable results for forming *H*-phosphonamidate linkages due to these advantages. Previous research has shown that the order of reactivity of condensing reagents is MNTP > PyNTP > PyBOP. This could be due to the ability of leaving groups (HOBt or 3-nitro 1,2,4-triazole (NT)) and the structure of the phosphonium center^26,27^. However, in this method, PyNTP showed a better result for the condensation reaction. The lower NMR yield using MNTP was due to the formation of by-products. Due to the higher activity as a condensing reagent, the activation of **2c** by MNTP might have led to the overactivation and caused inferior condensing efficacy. Thus, BOPCl, BOMP, and PyNTP were selected as condensing reagents for further investigation.

**Table 1:** Synthesis of dicytidine *H*-phosphonamidate 2-mers (C_PH_C).


Entry	Condensing reagent	NMR yield (%)^a^
1	PivCl	0
2	BOPCl	98
3	PyBOP	67
4	BOMP	98
5	PyNTP	98
6	MNTP	90

^a^Determined by ^31^P NMR.

In summary, we achieved the direct condensation of *H*-phosphonate monoester and the amino group of the morpholino nucleoside. Furthermore, the condensation reaction was completed within 20 min.

Although we attempted to isolate the *H*-phosphonamidate 2-mer **3cc**, **3cc** was unstable and readily hydrolyzed during extraction and purification by silica gel column chromatography. Thus, we attempted to convert the obtained *H*-phosphonamidate linkage to a stable phosphorodiamidate linkage as a one-pot reaction after the condensation reaction.

### Synthesis of phosphorodiamidate 2-mers (N_PN_N)

Next, we optimized the reaction conditions of a condensation reaction and an oxidative amination as a one-pot reaction (Table 2). To form an *H*-phosphonamidate linkage, the 1.2 equivalents of *H*-phosphonate monomer **2** were condensed with **1** bearing the amino group using BOPCl or PyNTP. Then, at 0 °C, the *H*-phosphonamidate derivative **3** was transformed to a phosphorodiamidate derivative **4 **by adding a mixture of dimethylamine and a halogenating reagent (I_2_, CBr_4_, or CCl_4_) to the reaction mixture. The conversion rate was determined using ^31^P NMR analysis of the reaction mixture. First, we investigated the impact of the halogenating reagents on the reaction outcomes and discovered that CCl_4_ gave the best results (See SI). The formation of **4cc** was confirmed using ^31^P NMR (δ 16.5, 16.9 ppm). However, the NMR yield of the desired product was moderate (47%) using BOPCl as a condensing reagent (Table 2, entry 1). Subsequently, PyNTP was employed as a condensing reagent instead of BOPCl, which significantly improved the result (91%, entry 2). We assumed that the residues of condensing reagent affected the oxidative amination reaction (See SI). The equivalent of dimethylamine was increased to 38 equivalents in entry 3, which improved the NMR yield to 98%. The oxidative amination reaction of *H*-phosphonamidate is thought to consist of a tautomerization to the tricoordinated phosporamidite intermediate, an oxidative halogenation reaction with CCl_4_, and a subsequent amination of the resultant intermediate^28–30^. Excess amounts of dimethylamine functioned as a base and accelerated a tautomerization to the tricoordinated phosporamidite intermediate, which promotes an oxidative amination reaction. The oxidative amination reaction time was set to 1 min in entry 4, and the NMR yield was increased to > 99%; therefore, the conditions in entry 4 were selected as optimal ones. With the optimized reaction conditions in hand, phosphorodiamidate 2-mers bearing other nucleobases were synthesized (entries 5, 6, and 7). All 2-mers were obtained in > 99% NMR yields, demonstrating that the reactions proceeded efficiently regardless of the specific nucleobase. Although dimethylamine in H_2_O solution was used as the optimized conditions, the hydrolysis of the *H*-phosphonamidate linkage was not observed. It is attributed to the much higher reactivity of dimethylamine than that of H_2_O to the chlorophosphoramidate intermediate. These results were in good agreement with the previous reports^29,30^.

**Table 2:** Synthesis of phosphprodiamidate 2-mers 4 (N_PN_N)^a^.


Entry	Monomer **2**	Nucleoside **1**	Condensing reagent	Equiv of Me_2_NH	Time (min)	Product	NMR yield (%)^b^
1	**2c**	**1c**	BOPCl	9.6^c^	30	C_PN_C (**4cc**)	47
2	**2c**	**1c**	PyNTP	9.6^c^	30	C_PN_C (**4cc**)	91
3	**2c**	**1c**	PyNTP	38^d^	30	C_PN_C (**4cc**)	98
4	**2c**	**1c**	PyNTP	38^d^	1	C_PN_C (**4cc**)	> 99
5	**2a**	**1a**	PyNTP	38^d^	1	A_PN_A (**4aa**)	> 99
6	**2g**	**1g**	PyNTP	38^d^	1	G_PN_G (**4gg**)	> 99
7	**2t**	**1t**	PyNTP	38^d^	1	T_PN_T (**4tt**)	> 99

^a^**1c**, **2c**, **3cc**, **4cc**: B = *N*^4^-benzoylcytosine, **1a**, **2a**, **3aa**, **4aa**: B = *N*^6^-benzoyladenine, **1g**, **2g**, **3gg**, **4gg**: B = *N*^2^-isobutyryl-*O*^6^-cyanoethylguanine, **1t**, **2t**, **3tt**, **4tt**: B = thymine.

^b^Determined by ^31^P NMR.

^c^2.0 M in THF solution was used.

^d^9.5 M in H_2_O solution was used.

The desired product and a triaminophosphine oxide derivative, which was the residue of PyNTP, were not separated by silica gel column chromatography despite our attempts to isolate phosphorodiamidate 2-mers **4**. Thus, the crude mixtures of **4** were employed for synthesizing 2-mer fragments without further purification.

### Synthesis of 2-mer fragments

**Figure 2:**
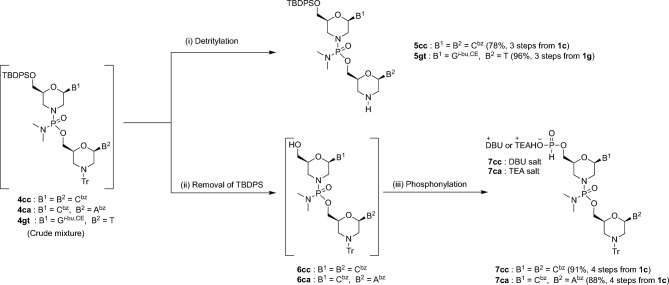
Synthesis of 2-mer fragments.

We analyzed the synthesis of two types of 2-mer fragments employing a crude mixture of phosphorodiamidate 2-mers **4** obtained by an optimized condensation and oxidative amination reaction as a one-pot reaction (Fig. 2). First, we attempted to synthesize 2-mer fragment bearing amino group **5cc** from a crude mixture of **4cc** through treatment with 3-cyanopyridine (CYP)-trifluoroacetic acid (TFA) in CH_2_Cl_2_-2,2,2-trifluoroethanol (TFE) (4:1, v/v). After a simple extraction and purification by silica gel column chromatography, we obtained the purified **5cc** in a 78% overall isolated yield (from **1c**, 3 steps). We used the same procedure to synthesize **5gt,** which was obtained in a 96% isolated yield (from **1g**, 3 steps). Next, we attempted to synthesize a 2-mer fragment **7cc** bearing 5ʹ-*H*-phosphonate from the crude mixture of **4cc** following the procedure in Fig. 2. First, we removed the 5ʹ-TBDPS group of **4cc** using TBAF, followed by a simple extraction to obtain compound **6cc**. The compound **6cc** was employed for the next phosphonylation reaction without further purification. Next, 5ʹ-*O*-phosphonylation of **6cc** was performed using pre-mixed PCl_3_ and imidazole as a phosphonylating reagent^19^. After a simple extraction and purification by silica gel column chromatography, we obtained the purified 5ʹ-*H*-phosphonate 2-mer **7cc** in a 91% overall isolated yield (from **1c**, 4 steps). **7ca** was also synthesized and obtained in an 88% isolated yield using the same procedure (from **1c**, 4 steps). These two types of fragments were easily separated from a triaminophosphine oxide derivative.

Reagents and conditions: (i) 3-cyanopyridine (10 equiv), CF_3_COOH (10 equiv), CH_2_Cl_2_–CF_3_CH_2_OH (4:1, v/v), rt, 1 h; (ii) TBAF (1.5 equiv), CH_3_COOH (1.5 equiv), THF, 0 °C, 1.5 h; (iii) PCl_3_ (5 equiv), imidazole (17 equiv), TEA (50 equiv), CH_2_Cl_2_, − 78 °C, 30 min.

### Synthesis of phosphorodiamidate 4-mer and 4-mer fragments

**Figure 3:**
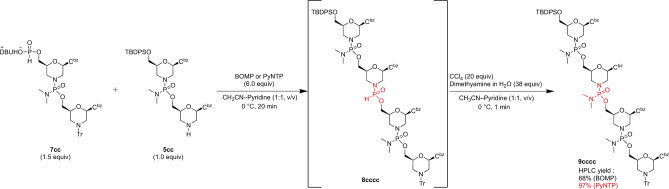
Synthesis of phosphorodiamidate 4-mer.

The successful synthesis of 2-mer fragments prompted the synthesis of a phoaphorodiamidate 4-mer using 2-mer fragments **5cc** and **7cc** through a fragment condensation reaction. We condensed the *H*-phosphonate 2-mer **7cc** with the amino group of **5cc** using PyNTP as the condensing reagent for 20 min, followed by the oxidative amination reaction employing CCl_4_ and dimethylamine to produce phosphorodiamidate 4-mer **9cccc** (Fig. 3). After removing any volatiles under reduced pressure, the resulting crude mixture was examined using reversed-phase high-performance liquid chromatography (RP-HPLC), and the condensation yields were estimated by the area ratios of the 4-mer **9cccc** to unreacted 2-mer **5cc**. The HPLC yield was 97%, indicating that the fragment condensation was proceeded almost quantitatively within 20 min. This was the first example of synthesizing PMOs using convergent synthesis. However, BOMP, which has HOBt as a leaving group gave a lower result (68%) than PyNTP, which has NT as a leaving group. This suggests that the presence of NT as a nucleophilic catalyst is essential for the fragment condensation reaction. Furthermore, the retention time of RP-HPLC of the product **9cccc** and 2-mer fragments (**5cc** and **7cc**) were significantly different, indicating a significant difference in lipophilicity between the 4-mer (**9cccc**) and 2-mer fragment (**5cc** and **7cc**) (See SI). This result shows that the convergent synthesis facilitates purification of the product.

Following the same procedure, a phosphorodiamidate 4-mer **9gtca** containing four types of nucleobases (A, C, G, and T) using 2-mer fragments (**5gt** and **7ca**) and PyNTP as a condensing reagent. After a simple extraction, 4-mers (**9cccc** and **9gtca**) were employed for the next reaction, without further purification.

**Figure 4:**
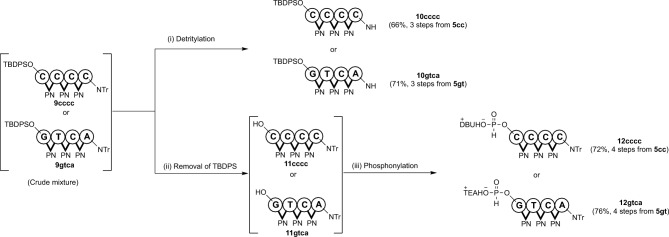
Synthesis of 4-mer fragments.

Subsequently, we studied the synthesis of two types of 4-mer fragments (**10** and **12**) following the same procedure used for the synthesis of 2-mer fragments (Fig. 4). The 4-mer fragments bearing amino groups **10cccc** and **10gtca** were obtained with 66% and 71% yields from **5cc** and **5gt**, respectively (3 steps). Furthermore, we obtained the 5ʹ-*H*-phosphonate 4-mer fragments of **12cccc** and **12gtca** in 72% and 76% yields from **5cc** and **5gt**, respectively (4 steps). In these reactions, any detectable side reactions to the nucleobases were not observed. The purity of these fragments was confirmed by NMR and HPLC (See SI).

Reagents and conditions: (i): 3-cyanopyridine (10 equiv), CF_3_COOH (10 equiv), CH_2_Cl_2_–CF_3_CH_2_OH (4:1, v/v), rt, 1 h; (ii) TBAF (1.5 equiv), CH_3_COOH (1.5 equiv), THF, 0 °C, 1.5 h; (iii) PCl_3_ (5 equiv), imidazole (17 equiv), TEA or *N*-methyl morpholine (50 equiv), CH_2_Cl_2_, − 78 °C, 30 min.

### Synthesis of phosphorodiamidate 6-mer and 8-mer

Next, we proceeded to synthesize 6-mer PMO and 8-mer PMO using 2-mer fragments and 4-mer fragments (Table 3). All oligomers were synthesized through condensation and oxidative amination procedures. After removing any volatiles under reduced pressure, we analyzed the resulting crude mixture using RP-HPLC and estimated the condensation yields by comparing the area ratios of the 6-mer or 8-mer to the unreacted 2-mer or 4-mer fragments bearing an amino group. Table 3 shows the results. First, we synthesized a 6-mer **15** by two different synthetic routes (Route A and B) to investigate the effect of a steric hindrance caused by the length of fragments. In route A, we condensed the 5ʹ-*H*-phosphonate 2-mer fragment **7cc** with the amino group of 4-mer fragment **10cccc**, whereas in route B, we condensed the 5ʹ-*H*-phosphonate 4-mer fragment **12cccc** with the amino group of 2-mer fragment **5cc**. In route A, we used PyNTP as a condensing reagent for the condensation reaction to produce the desired 6-mer **15** with a 95% HPLC yield (entry 1). However, in route B, the condensation reaction using PyNTP as a condensing reagent did not proceed sufficiently, leading to a low HPLC yield (26%, entry 2). Based on these results, we observed that longer 5ʹ-*H*-phosphonate fragments resulted in lower condensation efficacy. This data showed that the steric hindrance of 5ʹ-*H*-phosphonate fragments was crucial for the condensation efficacy. However, the effect of the steric hindrance of fragments bearing an amino group was less notable compared with 5ʹ-*H*-phosphonate fragments. To overcome the steric hindrance of 5ʹ-*H*-phosphonate fragments, we performed the condensation reaction of route B using MNTP, which has a higher activity as a condensing reagent than PyNTP (entry 3). To our delight, the HPLC yield was increased to 91%. The use of MNTP caused side reactions and was ineffective in synthesizing 2-mer; however, it gave the best result in the convergent synthesis due to its high activity. Next, we synthesized 8-mer **16** using PyNTP and MNTP as condensing reagents (entry 4 and 5, respectively; route C). As expected, MNTP gave higher condensation efficacy (92%, entry 5). Finally, we synthesized another 8-mer **17** bearing all four nucleobases using MNTP as a condensing reagent (route D), which yielded an 8-mer **17** with a 92% HPLC yield. These finding demonstrate that for the fragment condensation, the selection of condensing reagents is crucial, and MNTP is the optimal condensing reagent for the convergent synthesis of PMOs. Additionally, HPLC results showed a significant difference in lipophilicity between the 8-mer and 4-mer fragments (See SI).

**Table 3:** Synthesis of 6-mer and 8-mer.


Entry	Route	Condensing reagent	Product	HPLC yield (%)
1	A	PyNTP	6-mer** 15**	95
2	B	PyNTP	6-mer **15**	26
3	B	MNTP	6-mer **15**	91
4	C	PyNTP	8-mer **16**	41
5	C	MNTP	8-mer **16**	92
6	D	MNTP	8-mer **17**	92

Reagents and conditions of oxidative amination: CCl_4_ (20 equiv), dimethylamine in H_2_O (38 equiv), CH_3_CH–pyridine (1:1, v/v), 0 °C, 1 min.

In summary, we investigated the impact of a steric hindrance caused by the length of fragments and demonstrated that the steric hindrance of 5ʹ-*H*-phosphonate fragments was crucial for the condensation efficacy. By employing MNTP as a condensing reagent, we were able to overcome this issue and obtained 8-mer with outstanding HPLC yields.

### Deprotection and isolation

**Figure 5:**
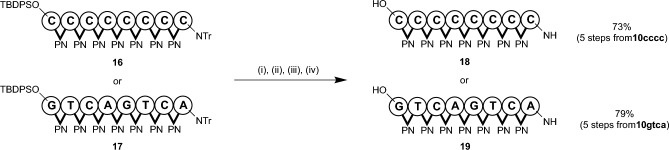
Deprotection and isolation of 8-mers.

Finally, we attempted to remove the protection groups of the fully protected 8-mers **16** and **17** using the following procedure: detritylation, removal of TBDPS group, deprotection of nucleobases, and purification by ODS column chromatography (Fig. 5). We obtained purified 8-mers **18** and **19** with 73% and 79% yields from **10cccc** and **10gtca**, respectively (5 steps). Product amounts were sufficient for the characterization by ESI–MS, ^1^H NMR, and ^31^P NMR.

Reagents and conditions: (i) 3-cyanopyridine (40 equiv), CF_3_COOH (40 equiv), CH_2_Cl_2_–CF_3_CH_2_OH (4:1, v/v), rt, 1 h; (ii) TBAF (40 equiv), THF, rt, 2 h; (iii) concentrated aqueous NH_3_–EtOH (3:1, v/v), 55 °C, 16 h; (iv) reverse phase column chromatography.

## Conclusion

In this study, we have developed a novel method for solution-phase synthesis of PMOs using an *H*-phosphonate approach. We directly condensed the *H*-phosphonate monomers with the amino group of morpholino nucleosides using specific phosphonium-type condensing reagents, including PyNTP, BOMP, and MNTP. The resulting *H*-phosphonamidate linkage was converted to a stable *N,N*-dimethylamino phosphorodiamidate counterpart as a one-pot reaction. These reactions substantially reduced the coupling time and yielded the product quantitatively. The obtained oligomers were converted to fragments (5ʹ-*H*-phosphonate monoester and 3ʹ-NH derivatives) for the next condensation reaction and isolated with substantial yields. We achieved fragment condensation with remarkable condensation efficacy to synthesize up to 8-mer using these fragments. This is the first report on solution-phase synthesis of PMOs using a fragment condensation reaction. Fragment condensation prevented the formation of N-1 mer of the product, making product isolation straightforward. Furthermore, since this approach yields *H*-phosphonamidate as intermediate, various *P*-modified PMOs such as TMOs would be obtained by an appropriate transformation of the intermediate^28^. This study’s approach is an effective alternative for synthesizing PMOs. The synthesis of various *P*-modified PMOs using the *H*-phosphonamidate derivative should be studied further.

## Methods

### General procedure for condensation reaction of amine and *H*-phosphonate derivatives

A morpholino nucleoside **1c** (0.0284 g, 0.050 mmol**)** and a *H*-phosphonate monomer **2c** (0.0473 g, 0.060 mmol) were dried by repeated coevaporation with dry pyridine and dissolved in a mixture of dry pyridine (0.5 mL) and acetonitrile-*d*_3_ (0.5 mL). A condensing reagent (0.15 mmol) was added to the solution at 0 °C and the mixture was stirred for 15 min at 0 °C. The solution was transferred into an NMR sample tube (5 mm × 180 mm) and a spectrum was recorded. The formation of **3cc** was confirmed by ^31^P NMR spectra (δ 14.2, 13.3 ppm, ^1^*J*_PH_ = 655, 661 Hz) (Fig. S1–S6).

### General procedure for condensation reaction and oxidative amination reaction as a one-pot reaction

A morpholino nucleoside (**1a**, **1g**, **1c**, or **1t**, 0.050 mmol**)** and 5ʹ-*H*-phosphonate (**2a**, **2g**, **2c**, or **2t,** 0.060 mmol) were dried by repeated coevaporation with dry pyridine and dissolved in a mixture of dry pyridine (0.5 mL) and acetonitrile-*d*_3_ (0.5 mL). A condensing reagent (0.15 mmol) was added to the solution at 0 °C and the mixture was stirred for 20 min at 0 °C. To the reaction mixture, a halogenation reagent (I_2_, CBr_4_, or CCl_4_) and dimethylamine were added at 25 °C or 0 °C and the mixture was stirred for designed time at 25 °C or 0 °C. The solution was transferred into an NMR sample tube (5 mm × 180 mm) and a spectrum was recorded. The formation of **4cc**, **4aa**, **4gg**, or **4tt** was confirmed by ^31^P NMR spectra (**4cc**: δ 16.9, 16.5 ppm, **4aa**: 16.8 ppm, **4gg**: 16.9, 16.7 ppm, and **4tt**: 17.0, 16.8 ppm) (Fig. S7–S21).

### General procedure for fragment condensation and oxidative amination as a one-pot reaction

The following procedure was used for the fragment condensation. 2-mer or 4-mer fragment bearing the 3ʹ-NH group (5 μmol) and 2-mer or 4-mer fragment bearing an *H*-phosphonate monoester on 5ʹ-OH group (7.5 μmol) was dried by repeated coevaporation with dry pyridine and dissolved in a mixture of dry pyridine (50 μL) and acetonitrile (50 μL). A condensing reagent (30 μmol) was added to the solution at 0 °C and the mixture was stirred for 20 min at 0 °C. To the mixture, CCl_4_ (10 μL, 100 μmol) and a 9.5 M dimethylamine aqueous solution (20 μL, 190 μmol) was added at 0 °C and the mixture was stirred for 1 min at 0 °C. Then, the mixture was diluted with CHCl_3_ (3 mL) and coevaporated with CHCl_3_ (3 × 3 mL), toluene (2 × 3 mL). The residue was analyzed by RP-HPLC. RP-HPLC was performed with a linear gradient of 0–60% MeCN for 60 min in a 0.1 M triethylammonium acetate buffer (pH 7.0) at 50 °C at a flow rate of 0.5 mL/min using a C18 column (100 Å, 3.9 mm × 150 mm). The condensation yields were estimated by the area rations of the 4-mer, 6-mer or 8-mer to unreacted 2-mer or 4-mer fragment bearing the 3ʹ-NH group.

### Supplementary Information

Supplementary Information.

## Data Availability

The data that support the findings of this study are available in the Supporting Information of this article.
