# Supplementary material for: Convergent synthesis of phosphorodiamidate morpholino oligonucleotides (PMOs) by the H-phosphonate approach
Source: Sci Rep. 2023 Aug 3;13:12576. doi: 10.1038/s41598-023-38698-2 (PMC10400599; doi:10.1038/s41598-023-38698-2)
Supplement: Supplementary file 1 — Supplementary Information. [file 41598_2023_38698_MOESM1_ESM.pdf]

Supplementary information

Convergent synthesis of Phosphorodiamidate Morpholino Oligonucleotides (PMOs) by the *H*-phosphonate approach

Taiki Tsurusaki[1], Kazuki Sato[1], Hiroki Imai[1], Kunihiro Hirai[2], Daisuke Takahashi[2], Takeshi Wada[1]*

1) *Department of Medicinal and Life Sciences, Faculty of Pharmaceutical Sciences, Tokyo University of Science, 2641 Yamazaki, Noda, Chiba 278-8510, Japan.*

2) *Research Institute For Bioscience Products & Fine Chemicals, Ajinomoto Co., Inc. 1-1, Suzuki-Cho, Kawasaki, Kanagawa 210-8681, Japan*

* Corresponding author
E-mail: twada@rs.tus.ac.jp

Table of contents

1. Experimental procedure and data S3

2. ^{31}P NMR analysis of the formation of *H*-phosphonamidate linkages (Table 1) S27

3. ^{31}P NMR analysis of the formation of phosphorodiamidate linkages (Table 2) S33

4. HPLC analysis of the fragment condensation reactions. S51

5. HPLC analysis of isolated compounds. S59

6. ^{1}H, ^{13}C, ^{31}P NMR spectra of isolated compounds. S69

1. Experimental section

General information

All reactions were conducted under an Ar atmosphere. Dry organic solvents were prepared by appropriate procedures. Additionally, ^{1}H NMR spectra were recorded at 400 or 600 MHz with tetramethylsilane (δ 0.0) as the internal standard in $CDCl_3$, CD_3CN, or pyridine-d_5 or CH_3CN (δ 2.06) as the internal standard in D_2O. Further, ^{13}C NMR spectra were recorded at 101 or 151 MHz with $CDCl_3$, which was used as the internal standard at δ 77.0 or CH_3CN (δ 1.47) as the internal standard in D_2O. Furthermore, ^{31}P NMR spectra were recorded at 162 MHz with H_3PO_4 (δ 0.0) as the external standard in $CDCl_3$, CD_3CN, or pyridine-d_5. Analytical thin-layer chromatography was performed on commercial glass plates with a 0.25 mm-thick silica gel layer. Manual silica gel column chromatography was performed on spherical, neutral, 63-210 μm silica gel. Automated flash chromatography was performed on silica gel (Yamazen UNIVERSAL Premium column (30 μm 60 Å)) or ODS silica gel (Yamazen UNIVERSAL Premium column (30 μm 120 Å)) (Yamazen Corporation) using an automated flash chromatography system W-prep 2XY (Yamazen Corporation). RP-HPLC for analysis and purification was performed using a μBondasphere 5 μm C18, 100 Å, 19 × 150 mm^2 (Waters).

Synthesis of compounds

General procedure for the synthesis of 5′-*O-tert*-butyldiphenylsilyl-morpholino nucleosides (1a, 1g, 1c, or 1t).

Scheme S 1

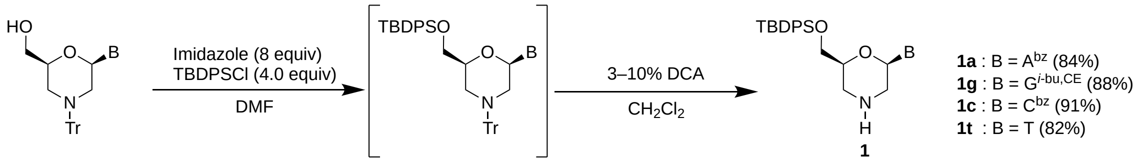

N^6-benzoyl-N'-trityl-morpholino adenosine (0.597 g, 1.0 mmol), N^2-isobutyryl-O^6-cyanoethyl-N'-trityl-morpholino guanosine (0.879 g, 1.39 mmol), N^4-benzoyl-N'-trityl-morpholino cytidine (5.72 g, 10 mmol) or N'-trityl-morpholino thymidine (0.967 g, 2.0 mmol) was dissolved in dry DMF (5 mL for the synthesis of **1a**, 6.95 mL for the synthesis of **1g**, 50 mL for the synthesis of **1c**, 10 mL for the synthesis of **1t**) under argon. Imidazole (0.545 g, 8.0 mmol for **1a**, 0.757 g, 11.12 mmol for **1g**, 5.45 g, 80 mmol for **1c**, and 1.09 g, 16 mmol for **1t**) and *tert*-butyldiphenylchlorosilane (TBDPSCl) (1.0 mL, 4.0 mmol for **1a**, 1.43 mL, 5.56 mmol for **1g**, 10.24 mL, 40 mmol for **1c**, and 2.05 mL, 8.0 mmol for **1t**) were successively added to the solution at 0 °C, and mixture was warmed to rt and allowed to stir for designated time (2 h for **1a**, 4 h for **1c**, 1 h for **1g**, and 45 min for **1t**).

MeOH (5 mL for **1a**, **1g,** and **1c**, 1 mL for **1t**) was added to the mixture. The mixture was diluted with EtOAc (30 mL for **1a**, 50 mL for **1g**, 100 mL for **1c**, and 20 mL for **1t**) and washed with H_2O (1×100 mL for **1c**, and 1×30 mL for **1t**) and brine (3×30 mL for **1a,** 3×50 mL for **1g**, and 2×100 mL for **1c**, and 2×30 mL for **1t**). The aqueous layers were combined and back-extracted with EtOAc (3×90 mL for **1a** and **1t**, 1×150 mL for **1g**, and 3×100 mL for **1c**). The combined organic layers were dried over with $MgSO_4$, filtered and concentrated under reduced pressure. The crude product was used in the next step for detritylation without further purification. The residue was dissolved in dry CH_2Cl_2 (20 mL for the synthesis of **1a**, 28 mL for the synthesis of **1g**, 50 mL for the synthesis of **1c**, 10 mL for the synthesis of **1t**) under argon. To a solution, dichloroacetic acid (DCA) solution in CH_2Cl_2 (6%, 20 mL for **1a**, 6%, 28 mL for **1g**, 16%, 50 mL for **1c** and 6%,10 mL for **1t**) was added at rt and allowed to stir for designated time (1 h for **1a** and **1g**, 2 h for **1c**, and 4 h for **1t**). For the synthesis of **1t**, DCA (1.4 mL) was added to the reaction mixture in an incremental manner. MeOH (5 mL for **1a, 1g,** and **1c**, and 1 mL for **1t**) was added to the mixture. The mixture was diluted with CH_2Cl_2 (10 mL for **1a**, **1g** and **1t**, and 50 mL for **1c**) and washed with saturated aqueous solutions of $NaHCO_3$ (3×30 mL for **1a** and **1t**, 3×50 mL for **1g**, and 3×100 mL for **1c**) and brine (1×100 mL for **1c**). The aqueous layers were combined and back-extracted with CH_2Cl_2 (3×90 mL for **1a**, 3×150 mL for **1g**, 1×100 mL for **1c**, and 4×90 mL for **1t**) and $CHCl_3$ (2×100mL for **1c**). The combined organic layers were dried over with Na_2SO_4, filtered and concentrated under reduced pressure. The residue was purified by manual silica gel chromatography (neutral silica gel, 20 g for **1a** and **1g**, 150 g for **1c** and 60 g for **1t**) using CH_2Cl_2–MeOH or $CHCl_3$–MeOH as the eluent. The fractions containing **1a**, **1g**, **1c**, or **1t** were collected and concentrated under reduced pressure to yield **1a**, **1g**, **1c** or **1t**.

***N*^6^-benzoyl-*O*-(*tert*-butyldiphenylsilyl)-Morpholino adenosine (1a).**

The crude mixture containing **1a** was purified by silica gel column chromatography using CH_2Cl_2–MeOH (99:1–95:5, v/v) as the eluent. **1a** was obtained as a colorless foam (0.499 g, 0.842 mmol, 84% yield).

^{1}H NMR (400 MHz, $CDCl_3$) δ 9.04 (s, 1H (-CONH)), 8.80 (s, 1H (H-2)), 8.12 (s, 1H (H-8)), 8.02 (d, *J* = 7.3 Hz, 2H (Ar)), 7.68–7.58 (m, 5H (Ar)), 7.52 (t, *J* = 7.6 Hz, 2H (Ar)), 7.45–7.32 (m, 6H (Ar)), 5.92 (dd, *J* = 10.1, 2.3 Hz, 1H (H-1′)), 4.01–3.97 (m, 1H (H-4′)), 3.79 (dd, *J* = 10.8, 4.8 Hz, 1H (H-5′)), 3.72 (dd, *J* = 10.5, 5.5 Hz, 1H (H-5′)), 3.37 (dd, *J* = 11.9, 2.3 Hz, 1H (H-2′)), 3.15 (dd, 12.6, 2.1 Hz, 1H (H-3′)), 3.03 (dd, *J* = 11.2, 10.1 Hz, 1H (H-2′)), 2.82 (dd, *J* = 11.7, 10.8 Hz, 1H (H-3′)), 1.9 (s, 1H (-NH)), 1.05 (s, 9H); ^{13}C {^{1}H} NMR (101 MHz, $CDCl_3$) δ 164.6 (-CONH-), 152.7 (C-2), 151.2 (C-4),

149.4 (C-6), 140.6 (C-8), 135.5, 135.5, 133.6, 133.1, 132.8, 129.8, 128.9, 127.8, 127.7 (Ar), 122.8 (C-5), 80.8 (C-1′), 78.8 (C-4′), 64.6 (C-5′), 50.5 (C-2′), 46.9 (C-3′), 26.7 (-C(C̲H3)3), 19.2 (-C̲(CH3)3)
ESI-MS *m/z* calcd for $C_{33}H_{37}N_6O_3Si$ $[M+H]^+$, 593.2691; found 593.2692.

***N*²-isobutyryl-*O*⁶-cyanoethyl-5′-*O*-(*tert*-butyldiphenylsilyl)-Morpholino guanosine (1g).**

The crude mixture containing **1g** was purified by silica gel column chromatography using $CHCl_3$–MeOH (99:1–96:4, v/v) as the eluent. **1g** was obtained as a colorless foam (0.764 g, 1.21 mmol, 88% yield).

^{1}H NMR (400 MHz, $CDCl_3$) δ 7.94 (s, 1H (H-8)), 7.85 (br, 1H (-CONH-)), 7.67–7.60 (m, 4H (Ar)), 7.44–7.33 (m, 6H (Ar)), 5.78 (dd, *J* = 10.1, 2.3 Hz, 1H (H-1′)), 4.82–4.76 (m, 2H (-OCH̲2-), 3.98–3.95 (m, 1H (H-4′)), 3.77 (dd, *J* = 10.8, 5.0 Hz, 1H (H-5′)), 3.69 (dd, *J* = 10.5, 5.5 Hz, 1H (H-5′)), 3.31 (dd, *J* = 12.1, 2.7 Hz, 1H (H-2′)), 3.13 (dd, *J* = 12.6, 2.3 Hz, 1H (H-3′)), 3.01 (t, *J* = 6.4 Hz, 3H (-CH̲2CN, -CH̲(CH3)2), 2.98 (d, *J* = 10.1 Hz, 1H (H-2′)), 2.78 (dd, *J* = 12.8, 10.5 Hz, 1H (H-3′)), 1.29 (dd, *J* = 6.9, 1.4 Hz, 6H (-CH(CH̲3)2), 1.04 (s, 9H (-C(CH̲3)3) ; ^{13}C {^{1}H} NMR (101 MHz, $CDCl_3$) δ 175.7 (-C̲ONH-), 159.6 (C-6), 152.5 (C-4), 151.7 (C-2), 139.8 (C-8), 135.5, 135.5, 133.1, 133.1, 129.8, 129.8, 127.7 (Ar), 117.6 (C-5), 116.8 (-CH2C̲N), 80.7 (C-1′), 78.6 (C-4′), 64.6 (C-5′), 61.6 (-OC̲H2-), 50.4 (C-2′), 46.9 (C-3′), 35.9 (-C̲H(CH3)2), 26.7 (-C(C̲H3)3), 19.3 (-CH(C̲H3)2, -C̲(CH3)3) , 18.1 (-C̲H2CN)
ESI-MS *m/z* calcd for $C_{33}H_{42}N_7O_4Si$ $[M+H]^+$, 628.3062; found 628.3061.

***N*⁴-benzoyl-5′-*O*-(*tert*-butyldiphenylsilyl)-Morpholino cytidine (1c).**

The crude mixture containing **1c** was purified by silica gel column chromatography using $CHCl_3$–MeOH (100:0–95:5, v/v) as the eluent. **1c** was obtained as a colorless foam (5.17g, 9.09 mmol, 91% yield).

^{1}H NMR (400 MHz, $CDCl_3$) δ 7.91 (d, *J*= 7.3 Hz, 2H (Ar)), 7.80 (d, *J*= 7.3 Hz, 1H (H-6)), 7.66 (td, *J*= 7.6, 0.9 Hz, 4H (Ar)), 7.61 (t, *J* = 7.6 Hz, 1H (Ar)), 7.55–7.35 (m, 9H (H-5, Ar), 5.72 (dd, *J* = 9.6 Hz, 2.3 Hz, 1H (H-1′)), 3.94–3.84 (m, 1H (H-4′)), 3.79 (dd, *J* = 11.0, 4.6 Hz, 1H (H-5′)), 3.73 (dd, *J* = 10.8, 5.3 Hz, 1H (H-5′)), 3.40 (dd, *J* = 12.4 Hz, 2.3 Hz, 1H (H-2′)), 3.07 (dd, *J* = 13.0 Hz, 2.1 Hz, 1H (H-3′)), 2.73 (dd, *J* = 13.0, 10.8 Hz, 1H (H-3′)), 2.42 (dd, *J* = 12.4, 9.6 Hz, 1H (H-2′)), 1.06 (s, 9H (-C(CH̲3)3). ^{13}C{^{1}H}NMR (101 MHz, $CDCl_3$) *δ* 166.6 (-CONH-), 162.1 (C-4), 154.3 (C-2), 144.3 (C-6), 135.5 133.2, 133.0, 133.0, 129.8, 129.0, 127.7, 127.7, 127.5 (Ar), 96.6 (C-5), 82.5 (C-1′), 79.2 (C-4′), 64.6 (C-5′), 49.9 (C-2′), 46.6 (C-3′), 26.7 (-C(C̲H3)3), 19.2 (-C̲(CH3)3). ESI-MS *m/z* calcd

for C32H37N4O4Si [M+H]$^+$, 569.2578; found 569.2570.

5′-*O*-(*tert*-butyldiphenylsilyl)-Morpholino thymidine (1t).

The crude mixture containing **1t** was purified by silica gel column chromatography using $CHCl_3$–MeOH (99.5:0.5–97:3, v/v) as the eluent. **1t** was obtained as a colorless foam (0.786 g, 1.64 mmol, 82% yield).

^{1}H NMR (400 MHz, $CDCl_3$) δ 7.65 (td, *J* = 7.8, 0.9 Hz, 4H (Ar)), 7.45–7.34 (m, 6H (Ar)), 7.22 (s, 1H (H-6)), 5.71 (dd, *J* = 10.1, 2.3 Hz, 1H (H-1′)), 3.93–3.83 (m, 1H (H-4′)), 3.74 (dd, *J* = 11.0, 4.6 Hz, 1H (H-5′)), 3.69 (dd, *J* = 10.8, 5.3 Hz, 1H (H-5′)), 3.13 (dd, *J* = 12.4, 2.3 Hz, 1H (H-2′)), 3.06 (dd, *J* = 12.8, 1.8 Hz, 1H (H-3′)), 2.73 (dd, *J* = 11.0, 12.4 Hz, 1H (H-3′)), 2.60 (dd, 12.4, 10.1 Hz, 1H (H-2′)), 1.88 (s, 3H (5-CH_3)), 1.06 (s, 9H (-C(C$\underline{H}_3$)$_3$)) ; ^{13}C {^{1}H} NMR (101 MHz, $CDCl_3$) δ 163.4 (C-4), 149.8 (C-2), 135.5 (C-6), 135.5, 133.1, 129.8, 127.7 (Ar), 110.7 (C-5), 80.5 (C-1′), 78.9 (C-4′), 64.6 (C-5′), 49.2 (C-2′), 46.6 (C-3′), 26.7 (-C($\underline{C}H_3$)$_3$), 19.3 (-$\underline{C}$(CH_3)$_3$), 12.5 (5-$\underline{C}H_3$)
ESI-MS *m/z* calcd for C26H34N3O4Si [M+H]$^+$, 480.2313; found 480.2298.

General procedure for the synthesis of triethylammonium or 1,8-diazabicyclo[5.4.0]undec-7-enium *N*′-trityl-morpholino nucleoside 5′-*H*-phosphonate (4a, 4g, 4c, or 4t).

Scheme S 2

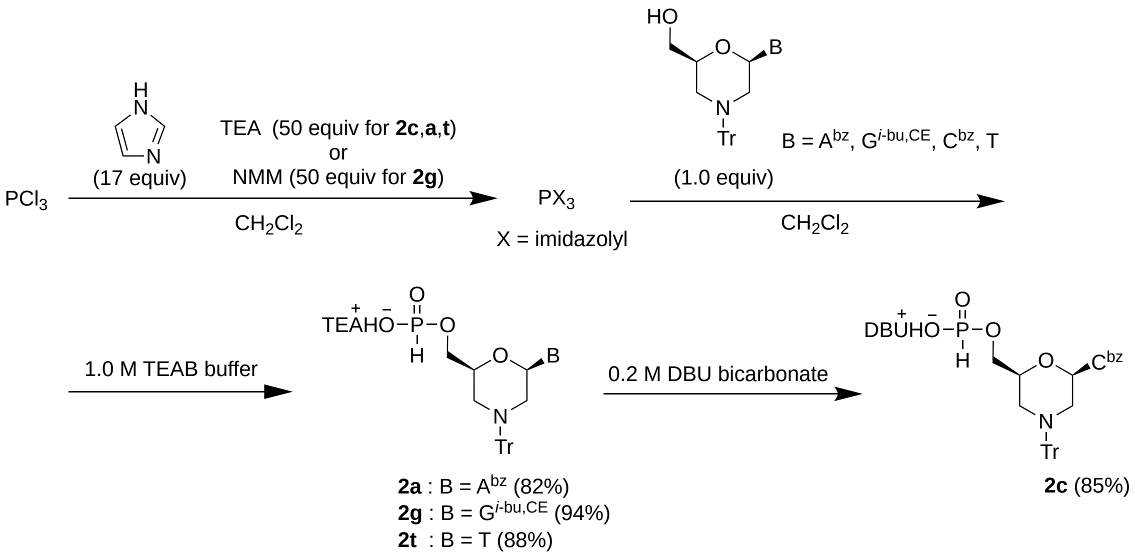

Imidazole (2.32 g, 34 mmol for **2a**, 0.232 g, 3.4 mmol for **2g**, 5.79 g, 85 mmol for **2c**, and 2.32 g, 34 mmol for **2t**) and TEA (14 mL, 100 mmol for **2a**, 35 mL, 250 mmol for **2c**, and 14 mL, 100 mmol for **2t**) or *N*-methylmorpholine (1.1 mL, 10 mmol for **2g**) were

dissolved in CH_2Cl_2 (100 mL for **2a**, 10 mL for **2g**, 250 mL for **2c**, and 100 mL for **2t**) under argon. PCl_3 (0.88 mL, 10 mmol for **2a**, 0.087 mL, 1.0 mmol for **2g**, 2.2 mL, 25 mmol for **2c**, and 0.88 mL, 10 mmol for **2t**) was added to the solution at rt, and the mixture was allowed to stir for 30 min. Then, the mixture was cooled to −78 °C (Solution A). N^6-benzoyl-*N*′-trityl-morpholino adenosine (1.19 g, 2.0 mmol), N^2-isobutyryl-O^6-cyanoethyl-*N*′-trityl-morpholino guanosine (0.126 g, 0.2 mmol), N^4-benzoyl-*N*′-trityl-morpholino cytidine (2.86 g, 5.0 mmol) or *N*′-trityl-morpholino thymidine (0.967 g, 2.0 mmol) was dried by coevaporation with dry pyridine and dissolved in dry CH_2Cl_2 (30 mL for **2a** and **2t**, 3.3 mL for **2g**, and 75 mL for **2c**) and added to the solution A at −78 °C dropwise over designated time (50 min for **2a**, 20 min for **2g**, 1.5 h for **2c**, 45 min for **2t**). The mixture was allowed to stir for 30 min at −78 °C and then designated time (1.5 h for **2a**, 30 min for **2g**, 2 h for **2c**, and 1 h for **2t**) at rt. To the mixture, 1.0 M TEAB buffer (150 mL for **2a**, 15 mL for **2g**, 375 mL for **2c**, and 100 mL for **2t**) was added and the mixture was allowed to stir for 5 min and then the organic layer was separated in a separating funnel. The organic layer was washed with 1.0 M TEAB buffer (3×20 mL for **2g**, and 2×100 mL for **2t**). The aqueous layers were combined and back-extracted with CH_2Cl_2 (3×400 mL for **2a**, 3×60 mL for **2g**, 2×500 mL for **2c**, and 2×200 mL for **2t**). The combined organic layers were dried over with Na_2SO_4, filtered and concentrated under reduced pressure. The residue was purified by silica gel chromatography (neutral silica gel, 90 g for **2a**, 10 g for **2g**, 150 g for **2c**, 100 g for **2t**) using CH_2Cl_2–MeOH–Et_3N or $CHCl_3$–MeOH–Et_3N as the eluent. The fractions containing **2a**, **2g**, **2c**, or **2t** were collected and concentrated under reduced pressure to yield **2a**, **2g**, or **2t** as trietylammonium salts. A solution of $CHCl_3$–pyridine (2:1, v/v, 100 mL) was added to the residue of **2c** and washed with 0.2 M DBU hydrogen carbonate aqueous solution (2×100 mL). The aqueous layers were combined and back-extracted with $CHCl_3$–pyridine (3×200 mL). The organic layers were combined, dried over with Na_2SO_4, filtered, and concentrated under reduced pressure to yield **2c** as a DBU salt.

Triethylammonium N^6-benzoyl-*N*′-trityl-morpholino adenosine 5′-*H*-phosphonate (2a).

The crude mixture containing **2a** was purified by silica gel column chromatography using CH_2Cl_2–MeOH–Et_3N (98:1:1–93:6:1, v/v/v) as the eluent. **2a** was obtained as a colorless foam with 1.0 equivalent of imidazole (1.36 g, 1.64 mmol, 82% yield).

^{1}H NMR (400 MHz, $CDCl_3$) δ 9.6 (br, 1H (-CONH-)), 8.77 (s, 1H (H-2)), 8.00 (s, 2H (Ar)), 7.98 (d, J = 1.4 Hz, 1H (H-8)), 7.58–7.39 (m, 8H (Ar)), 7.34–7.23 (m, 6H (Ar)), 7.18 (t, J = 7.1 Hz, 3H (Ar)), 6.84 (d, J = 623.0 Hz, 1H (P-H)), 6.39 (dd, J = 9.8, 2.1 Hz,

1H (H-1′)), 4.53–4.37 (m, 1H (H-4′)), 3.95–3.83 (m, 2H (H-5′)), 3.50 (d, *J* = 11.0 Hz, 1H (H-2′)), 3.26 (d, *J* = 11.9 Hz, 1H (H-3′)), 2.93 (q, *J* = 7.3 Hz, 5H (-NC<u>H</u>$_2$CH$_3$), 1.83 (t, *J* = 10.5 Hz, 1H (H-2′)), 1.62 (t, *J* = 11.2 Hz, 1H (H-3′)), 1.23 (t, *J* = 7.3 Hz, 7.5H (-NCH$_2$C<u>H</u>$_3$)); ^{13}C {^{1}H} NMR (101 MHz, CDCl$_3$) δ 164.9 (-NHCO-), 152.6 (C-2), 151.2 (C-4), 149.4 (C-6), 140.8 (C-8), 133.5, 132.7, 129.1, 128.7, 127.9, 127.9, 126.6 (Ar), 122.7 (C-5), 80.1 (C-1′), 76.9 (-<u>C</u>(Ar)$_3$), 76.0 (d, $^3J_{PC}$ = 7.7 Hz, C-4′), 64.2(d, $^2J_{PC}$ = 2.9 Hz, C-5′), 53.0 (C-2′), 49.3 (C-3′), 45.4 (-N<u>C</u>H$_2$CH$_3$), 8.5 (-CH$_2$<u>C</u>H$_3$); ^{31}P{^{1}H}NMR (162 MHz, CDCl$_3$) *δ* 4.9 ($^1J_{PH}$ = 630 Hz). ESI-MS *m/z* calcd for $C_{36}H_{32}N_6O_5P$ [M-H-TEA]$^-$, 659.2177; found 659.2153.

Triethylammonium *O*^{6}-cyanoethyl-*N*^{2}-*i*-butyryl-*N′*-trityl-morpholino guanosine 5′-*H*-phosphonate (2g).

The crude mixture containing **2g** was purified by silica gel column chromatography using CHCl$_3$–MeOH–Et$_3$N (96.5:3:0.5–93.5:6:0.5, v/v/v) as the eluent. **2g** was obtained as a colorless foam with 0.7 equivalents of imidazole (0.158 g, 0.187 mmol, 94% yield).

^{1}H NMR (400 MHz, CDCl$_3$) δ 8.7 (br, 1H (-NHCO-)), 7.78 (s, 1H (H-8)), 7.47 (br, 5H (Ar)), 7.28 (t, *J* = 7.8 Hz, 7H (Ar)), 7.17 (t, *J* = 6.9 Hz, 3H (Ar)), 6.87 (d, $^1J_{PH}$ = 625.3 Hz, PH), 6.25 (dd, *J* = 9.8, 2.1 Hz, 1H (H-1′)), 4.76 (t, *J* = 6.4 Hz, 2H (-OC<u>H</u>$_2$CH$_2$-)), 4.45–4.42 (m, 1H (H-4′)), 3.92-3.82 (m, 2H (H-5′)), 3.45(d, *J* = 11.4 Hz, 1H (H-2′)), 3.22 (d, 11.9 Hz, 1H (H-3′)), 3.02–2.95 (m, 6H (-N<u>C</u>H$_2$CH$_3$)), 1.81 (t, *J* = 10.5 Hz, 1H (H-2′)), 1.56 (t, *J* = 11.2 Hz, 1H (H-3′)), 1.34 (d, *J* = 6.9Hz, 3H (-CH(C<u>H</u>$_3$)$_2$), 1.32 (d, *J* = 6.9Hz, 3H (-CH(C<u>H</u>$_3$)$_2$)), 1.26 (t, *J* = 7.3 Hz, 9H (-CH$_2$C<u>H</u>$_3$); ^{13}C{^{1}H}NMR (101 MHz, CDCl$_3$) *δ* 175.8 (-NHCO-), 159.5 (C-6), 152.5 (C-4), 152.0 (C-2), 139.6 (C-8), 129.2, 127.9, 126.5 (Ar), 117.3 (C-5), 116.9 (-CH$_2$<u>C</u>N), 80.0 (C-1′), 75.8 (d, $^3J_{PC}$ = 6.7 Hz, C-4′), 64.4 (d, $^2J_{PC}$ = 3.9 Hz, C-5′), 61.6 (-O<u>C</u>H$_2$-), 52.6 (C-2′), 49.2 (C-3′), 45.5 (-N<u>C</u>H$_2$-), 35.9 (-<u>C</u>H(CH$_3$)$_2$), 19.4, 19.3 (-CH(CH$_3$)$_2$, rotamers), 18.1 (-<u>C</u>H$_2$CN), 8.5 (-NCH$_2$<u>C</u>H$_3$); ^{31}P{^{1}H}NMR (162 MHz, CDCl$_3$) *δ* 4.9 ($^1J_{PH}$ =626 Hz) ESI-MS *m/z* calcd for $C_{36}H_{37}N_7O_6P$ [M-H-TEA]$^-$, 694.2548; found 694.2544.

1,8-diazabicyclo[5.4.0]undec-7-enium *N*^{4}-benzoyl-*N′*-trityl-morpholino cytidine 5′-*H*-phosphonate (2c).

The crude mixture containing **2c** was purified by silica gel column chromatography using CHCl$_3$–MeOH–Et$_3$N (98:1.5:0.5–94.5:5:0.5, v/v/v) as the eluent for the first time and CHCl$_3$–MeOH–Et$_3$N (99:0:1–89:10:1, v/v/v) as the eluent for the second time. The fractions containing **2c** were collected and concentrated under reduced pressure. A solution of CHCl$_3$–pyridine (2:1, v/v, 100 mL) was added to the residue and washed with

0.2 M DBU hydrogen carbonate aqueous solution (2×100 mL). The aqueous layers were combined and back-extracted with $CHCl_3$–pyridine (3×200 mL). The organic layers were combined, dried over with Na_2SO_4, filtered, and concentrated under reduced pressure to yield **2c** as a colorless foam with 0.8 equivalents of imidazole (3.57 g, 4.24 mmol, 85% yield).

^{1}H NMR (400 MHz, $CDCl_3$) δ 7.90 (s, 1H (H$^+$DBU), 7.88 (s, 1H (Ar)), 7.73 (d, J = 7.3 Hz, 1H (H-6)), 7.57 (t, J = 7.1 Hz, 1H (Ar)), 7.53–7.39 (m, 7H (Ar)), 7.33–7.22 (m, 9H (Ar, H-5)), 7.16 (t, J = 7.1 Hz, 3H (Ar)), 6.85 (d, J_{PH} = 617.7 Hz, PH), 6.22 (dd, J =1.8, 9.1Hz, 1H (H-1′)), 4.41–4.32 (m, 1H (H-4′)), 3.93–3.78 (m, 2H (H-5′)), 3.53 (d, J = 11.4 Hz, 1H (H-2′)), 3.49–3.42 (m, 2H (DBU)), 3.40 (t, J = 5.9 Hz, 2H (DBU)), 3.33 (t, J = 5.7 Hz, 2H (DBU)), 3.17 (d, J = 11.9 Hz, 1H (H-3′)), 2.82–2.76(m, 2H (DBU)), 2.01–1.89 (m, 2H (DBU), 1.78–1.58 (m, 6H (DBU)), 1.51 (t, J = 11.2 Hz, 1H (H-3′)), 1.28 (t, J = 10.5 Hz, 1H (H-2′)). ^{13}C{^{1}H}NMR (101 MHz, $CDCl_3$) δ 166.1 (C-DBU), 162.2 (C-4), 154.2 (C-2), 144.7 (C-6), 133.1, 129.1, 128.9, 127.8, 127.7, 126.4 (Ar), 96.7 (C-5′), 82.0 (C-1′), 76.2 (d, $^3J_{PC}$ = 6.7 Hz, C-4′), 64.2 (d, $^2J_{PC}$ = 2.3 Hz, C-5′), 54.3 (-CH_2-(DBU)), 52.7 (C-2′), 49.3 (C-3′), 48.6 (-CH_2-(DBU)), 37.8 (-CH_2-(DBU)), 32.1 (-CH_2-(DBU)), 29.0 (-CH_2-(DBU)), 26.8 (-CH_2-(DBU)), 24.0 (-CH_2-(DBU)), 19.4 (-CH_2-(DBU)); ^{31}P{^{1}H}NMR (162 MHz, $CDCl_3$) δ 5.1 ($^1J_{PH}$ = 620 Hz). ESI-MS *m/z* calcd for $C_{35}H_{32}N_4O_6P$ [M-H-DBU]$^-$, 635.2065; found 635.2045.

Triethylammonium *N′*-trityl-morpholino thymidine 5′-*H*-phosphonate (2t).

The crude mixture containing **2t** was purified by silica gel column chromatography using $CHCl_3$–MeOH–Et_3N (98.5:1:0.5–91.5:8:0.5, v/v/v) as the eluent. **2t** was obtained as a colorless foam with 0.8 equivalents of imidazole (1.24 g, 1.76 mmol, 88% yield).

^{1}H NMR (400 MHz, $CDCl_3$) δ 10.5 (br, 1H (H-3), 7.57–7.33 (br, 5H (Ar)), 7.32–7.22 (m, 7H (Ar)), 7.19 (t, J = 6.6 Hz 3H (Ar)), 7.01 (d, J = 0.9 Hz, 1H (H-6)), 6.87 (d, J = 622.5 Hz, 1H (PH)), 6.12 (dd, J = 9.6, 2.3 Hz, 1H (H-1′)), 4.41–4.31 (m, 1H (H-4′)), 3.96–3.79 (m, 2H (H-5′)), 3.32 (d, J = 11.0 Hz, 1H (H-2′)), 3.17 (d, J = 11.9 Hz, 1H (H-3′)), 3.00 (q, J = 7.3 Hz, 6H (-NC$\underline{H}_2$$CH_3$)), 1.79 (d, J = 0.7 Hz, 3H (5-C$\underline{H}_3$)), 1.49–1.42 (m, 2H (H-2′, H-3′)), 1.29 (t, J = 7.3 Hz, 9H (-NCH_2C$\underline{H}_3$); ^{13}C{^{1}H}NMR (101 MHz, $CDCl_3$) δ 163.6 (C-4), 150.0 (C-2), 135.6 (C-6), 129.1, 127.8, 126.5 (Ar), 110.4 (C-5), 80.3 (C-1′), 77.2 (-$\underline{C}$(Ar)$_3$), 76.0 (d, $^3J_{PC}$ = 6.7 Hz, C-4′), 64.3 (d, $^2J_{PC}$ = 2.9 Hz, C-5′), 51.7 (C-2′), 49.2 (C-3′), 45.5 (-N$\underline{C}H_2CH_3$), 12.4 (5-$\underline{C}H_3$), 8.6 (-N$CH_2\underline{C}H_3$); ^{31}P{^{1}H}NMR (162 MHz, $CDCl_3$) δ 5.0 ($^1J_{PH}$ = 626 Hz). ESI-MS *m/z* calcd for $C_{29}H_{29}N_3O_6P$ [M-H-TEA]$^-$, 546.1799; found 546.1800.

^{31}P NMR study of the condensation reaction. (Table 1)

A morpholino nucleoside **1c** (0.0284 g, 0.050 mmol) and a *H*-phosphonate monomer **2c** (0.0473 g, 0.060 mmol) were dried by repeated coevaporation with dry pyridine and dissolved in a mixture of dry pyridine (0.5 mL) and acetonitrile-d_3 (0.5 mL). A condensing reagent (0.15 mmol) was added to the solution at 0 °C and the mixture was stirred for 15 min at 0 °C. The solution was transferred into an NMR sample tube (5 mm × 180 mm) and a spectrum was recorded. The formation of **3cc** was confirmed by ^{31}P NMR spectra (δ 14.2, 13.3 ppm, $^{1}J_{PH}$ = 655, 661 Hz) (Fig. S1–S6)

^{31}P NMR study of the condensation reaction and oxidative amination reaction as a one-pot reaction. (Table 2)

A morpholino nucleoside (**1a**, **1g**, **1c**, or **1t**, 0.050 mmol) and 5′-*H*-phosphonate (**2a**, **2g**, **2c**, or **2t,** 0.060 mmol) were dried by repeated coevaporation with dry pyridine and dissolved in a mixture of dry pyridine (0.5 mL) and acetonitrile-d_3 (0.5 mL). A condensing reagent (0.15 mmol) was added to the solution at 0 °C and the mixture was stirred for 20 min at 0 °C. To the reaction mixture, a halogenation reagent (I_2, CBr_4, or CCl_4) and dimethylamine were added at 25 °C or 0 °C and the mixture was stirred for designated time at 25 °C or 0 °C. The solution was transferred into an NMR sample tube (5 mm × 180 mm) and a spectrum was recorded. The formation of **4cc**, **4aa**, **4gg**, or **4tt** was confirmed by ^{31}P NMR spectra (**4cc**: δ 16.9, 16.5 ppm, **4aa**: 16.8 ppm, **4gg**: 16.9, 16.7 ppm, and **4tt**: 17.0, 16.8 ppm) (Fig. S7–S20)

General procedure for the synthesis of the 2-mer fragments bearing 3′-NH group (5cc or 5gt).

Scheme S 3

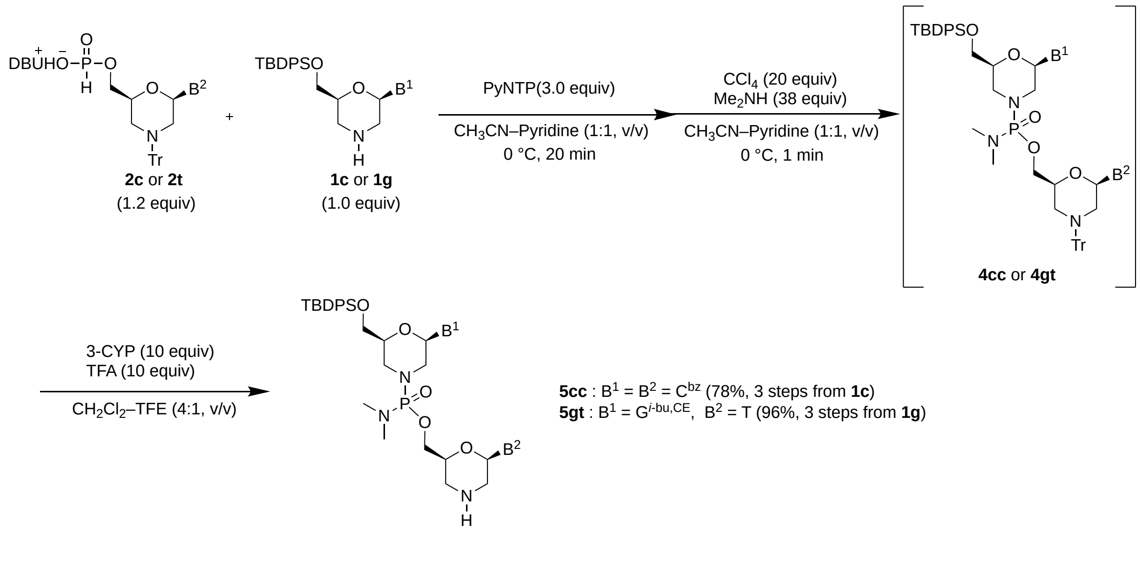

A morpholino nucleoside **1c** (0.455 g, 0.80 mmol) or **1g** (0.628 g, 1.0 mmol) and a 5′-*H*-phosphonate morpholino nucleoside **2c** (0.757 g, 0.96 mmol) or **2t** (0.778 g, 1.2 mmol) were dried by repeated coevaporation with dry pyridine and dissolved in a mixture of dry pyridine (8 mL for **5cc**, 10 mL for **5gt**) and dry acetonitrile (8 mL for **5cc**, 10 mL for **5gt**). PyNTP (1.19 g, 2.4 mmol for **5cc**, 1.50 g, 3.0 mmol for **5gt**) was added to the solution at 0 °C and mixture was stirred for 20 min at 0 °C. To the mixture, CCl_4 (1.55 mL, 16 mmol for **5cc**, 1.93 mL, 20 mmol for **5gt**) and a 9.5 M dimethylamine aqueous solution (3.2 mL, 30.4 mmol for **5cc**, 4.0 mL, 38 mmol for **5gt**) were added at 0 °C and the mixture was stirred for 1 min at 0 °C. Then, the mixture was diluted with $CHCl_3$ (100 mL for **5cc**, 50 mL for **5gt**) and washed with saturated aqueous solutions of $NaHCO_3$ (3×100 mL for **5cc**, 3×50 mL for **5gt**). The aqueous layers were combined and back-extracted with $CHCl_3$ (3×300 mL, for **5cc**, 3×150 mL for **5gt**). The organic layers were combined, dried over with Na_2SO_4, filtered, and concentrated under reduced pressure. The residue was dissolved in CH_2Cl_2 (16 mL for **5cc**, 20 mL for **5gt**) (solution A). 3-cyanopyridine (0.833 g, 8 mmol, for **5cc**, 1.04 g, 10 mmol for **5gt**) and CF_3COOH (0.61 mL, 8 mmol for **5cc**, 0.76 mL, 10 mmol for **5gt**) were dissolved in a mixture of CH_2Cl_2–CF_3CH_2OH (10:6, v/v) (16 mL for **5cc**, 20 mL for **5gt**) (solution B). To the solution A, the solution B was added at rt and allowed to stir for designated time (45 min for **5cc**, 30 min for **5gt**). The mixture was diluted with CH_2Cl_2 (40 mL) and washed with a saturated aqueous solution of $NaHCO_3$ (3×50 mL). The aqueous layers were combined and back-extracted with CH_2Cl_2 (3×150 mL). The combined organic layers were dried over with Na_2SO_4, filtered and concentrated under reduced pressure. The residue was purified by silica gel chromatography (neutral silica gel, 20 g for **5cc** and 25 g for **5gt**) using $CHCl_3$–MeOH as the eluent. The fractions containing **5cc** or **5gt** were collected and concentrated under reduced pressure to yield **5cc** or **5gt**.

C-C 2-mer fragment bearing 3′-NH group (5cc).

The crude mixture containing **5cc** was purified by silica gel column chromatography using $CHCl_3$–MeOH (97:3–95:5, v/v) as the eluent. **5cc** was obtained as a colorless foam (0.615 g, 0.622 mmol, 78 % yield from **1c**, 3 steps).

^{1}H NMR (400 MHz, $CDCl_3$) δ 8.11 (d, *J* = 7.3 Hz, 0.5H, H-6 (3′-downstream), one of diastereomers), 8.03 (d, *J* = 7.8 Hz, 0.5 H (3′-downstream), one of diastereomers), 7.96–7.83 (m, 4H (Ar)), 7.76 (t, *J* = 8.2 Hz, 1H, H-6 (5′-upstream)), 7.70–7.35 (m, 18H, H-5, Ar), 5.82 (dd, *J* = 9.4, 2.1 Hz, 1H, H-1′ (3′-downstream)), 5.69 (dd, *J* = 9.6, 2.3 Hz, 1H, H-1′ (5′-upstream)), 4.15–3.70 (m, 7H, H-2′ (5′-upstream), H-4′, H-5′, 5′′), 3.61–3.29 (m, 2H, H-2′ (3′-downstream), H-3′ (3′-downstream)), 3.07–2.65 (m, 9H, H-3′, -N(C<u>H</u>$_3$)$_2$),

2.59–2.40 (m , 2H, H-2′ ′), 1.07 (s, 9H (-C(C$\underline{H}_3$)$_3$); $^{13}C\{^1H\}$NMR (101 MHz, $CDCl_3$) δ 166.8 (-NHCO-), 162.4 (C-4 (5′-upstream)), 162.2 (C-4 (3′-downstream)), 154.1 (C-2 (3′-downstream)), 153.9 (C-2 (5′-upstream)), 144.7, 144.5 (C-6 (3′-downstream), diastereomers), 143,6 (C-6 (5′-upstream)), 135.3, 133.0, 132.9, 132.8, 132.8, 132.7, 132.7, 129.8, 128.7, 127.9, 127.6, 127.6, 127.3 (Ar), 96.9 (C-5 (3′-downstream)), 96.7 (C-5 (5′-upstream)), 82.3, 82.1 (C-1′ (3′-downstream), diastereomers), 81.5, 81.4 (d, $^3J_{PC}$ = 8.7 Hz, C-1′ (5′-upstream), diastereomers), 77.8, 77.7 (C-4′, diastereomers), 77.2 (C-4′), 65.2 (d, $^2J_{PC}$ = 4.8 Hz, C-5′ (3′-downstream), one of diastereomers), 65.0 (d, $^2J_{PC}$ = 3.9 Hz, C-5′ (3′-downstream), one of diastereomers), 64.1, 64.0 (C-4′ (5′-upstream), diastereomers), 49.4 (C-2′), 48.0, 47.9 (C-2′, diastereomers), 45.7, 45.6 (C-3′, diastereomers), 45.0 (C-3′), 36.6, 36.5 (d, $^2J_{PC}$ = 3.9 Hz, -N($\underline{C}H_3$)$_2$, diastereomers), 26.6 (-C($\underline{C}H_3$)$_3$), 19.0 (-$\underline{C}$(CH_3)$_3$). $^{31}P\{^1H\}$NMR (162 MHz, $CDCl_3$) δ 17.0, 16.4. ESI-MS *m/z* calcd for $C_{50}H_{59}N_9O_9PSi$ $[M+H]^+$, 988.3937; found 988.3930.

G-T 2-mer fragment bearing 3′-NH group (5gt).

The crude mixture containing **5gt** was purified by silica gel column chromatography using $CHCl_3$–MeOH (99:1–90:10, v/v) as the eluent. **5gt** was obtained as a colorless foam (0.919 g, 0.959 mmol, 96% yield from **1g**, 3 steps).

^{1}H NMR (400 MHz, $CDCl_3$) δ 9.23 (s, 0.5H, -N$\underline{H}$CO- (guanosine), one of diastereomers), 9.1 (br, 1H, H-3 (thymidine)), 8.94 (s, 0.5H, -N$\underline{H}$CO- (guanosine), one of diastereomers), 7.94, 7.91 (s, 1H, H-8 (guanosine), diastereomers), 7.68–7.59 (m, 4H, Ar), 7.46–7.32 (m, 6H, Ar), 7.24, 7.21 (d, *J* = 0.9 Hz, 1H, H-6 (thymidine), diastereomers), 5.83 (dt, *J* = 10.1, 2.3 Hz, 1H, H-1′ (guanosine)), 5.74 (dt, *J* = 8.9, 2.3 Hz, 1H, H-1′ (thymidine)), 4.87–4.74 (m, 2H, -OC$\underline{H}_2$$CH_2$-), 4.20–3.91 (m, 4H, H-4′, H-5′ (thymidine)), 3.83–3.60 (m, 3.5H, H-2′, H-3′, H-5′ (guanosine), one of diastereomers), 3.43 (t, *J* = 10.3 Hz, 0.5H, H-3′, one of diastereomers), 3.21–2.81 (m, 8.5H, H-2′, H-3′, -CH_2C$\underline{H}_2$CN, -C$\underline{H}$(CH_3)$_2$, one of diastereomers), 2.79–2.62 (m, 6.5H, -N(C$\underline{H}_3$)$_2$, H-2′ (thymidine), one of diastereomers), 1.84, 1.80 (s, 3H, 5-C$\underline{H}_3$, diastereomers), 1.27 (d, *J* = 6.9 Hz, 6H, -CH(C$\underline{H}_3$)$_2$), 1.05 (s, 9H, -C(C$\underline{H}_3$)$_3$); $^{13}C\{^1H\}$NMR (101 MHz, $CDCl_3$) δ 176.3, 175.9 (-NHCO-, diastereomers), 163.3, 163.3 (C-4 (thymidine), diastereomers), 159.6, 159.6 (C-6 (guanosine), diastereomers), 152.4, 152.4 (C-4 (guanosine), diastereomers), 152.3, 152.2 (C-2 (guanosine), diastereomers), 150.0, 149.9 (C-2 (thymidine), diastereomers), 139.2 (C-8 (guanosine)), 135.5 (Ar), 135.0 (C-6 (thymidine), 132.8, 132.8, 132.8, 132.7, 130.0, 129.9, 129.9, 129.8, 127.8 (Ar), 117.4, 117.4 (C-5 (guanosine), diastereomers), 116.9, 116.9 (-$\underline{C}$N, diastereomers), 111.0, 111.0 (C-5 (thymidine), diastereomers), 80.4, 80.3 (C-1′ (thymidine), diastereomers), 79.6, 79.6 (d, $^3J_{PC}$ = 5.8 Hz, C-1′ (guanosine),

diastereomers), 77.9, 77.8 (C-4′, diastereomers), 77.4, 77.3 (C-4′, diastereomers), 65.4 (d, $^2J_{PC}$ = 5.8 Hz, C-5′ (thymidine) , one of diastereomers), 65.4 (d, $^2J_{PC}$ = 3.9 Hz, C-5′ (thymidine), one of diastereomers), 64.1, 64.0 (C-5′ (guanosine), diastereomers), 61.7, 61.6 (-O<u>C</u>H_2CH_2-, diastereomers), 48.9, 48.7 (C-2′, diastereomers), 48.4, 48.3 (C-2′, diastereomers), 46.1 (C-3′), 45.7, 45.4 (C-3′, diastereomers), 36.8, 36.6 (d, $^2J_{PC}$ = 3.9 Hz, -N(<u>C</u>$H_3)_2$, diastereomers), 35.8, 35.7 (-<u>C</u>H($CH_3)_2$, diastereomers), 26.7 (-C(<u>C</u>$H_3)_3$), 19.4 (-CH(<u>C</u>$H_3)_2$), 19.2 (-<u>C</u>($CH_3)_3$), 18.1 (-CH_2<u>C</u>H_2CN), 12.5, 12.5 (5-<u>C</u>H_3, diastereomers); $^{31}P\{^1H\}$NMR (162 MHz, $CDCl_3$) δ 17.5, 17.1. ESI-MS *m/z* calcd for $C_{45}H_{61}N_{11}O_9PSi$ $[M+H]^+$, 958.4155; found 958.4157.

General procedure for the synthesis of the 2-mer fragments bearing *H*-phosphonate monoester on 5′-OH (7cc or 7ca).

Scheme S 4

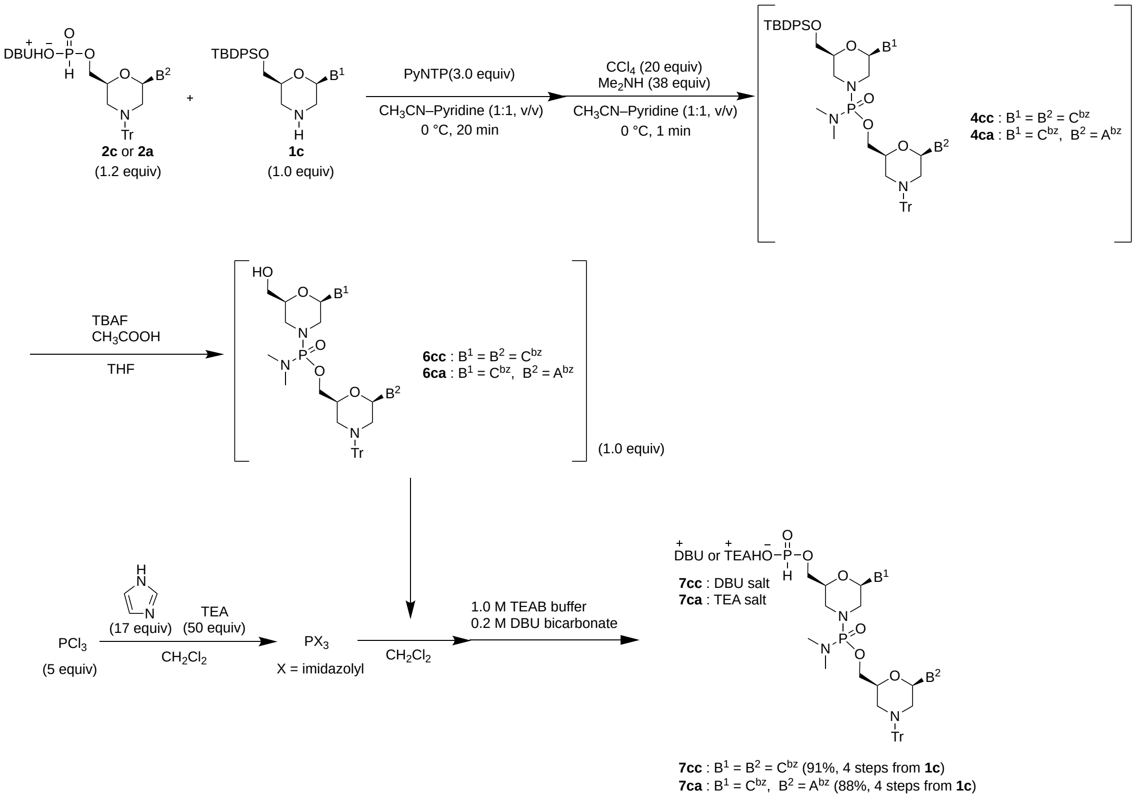

A morpholino nucleoside **1c** (0.455 g, 0.80 mmol for **7cc**, or 0.569 g, 1.0 mmol for **7ca**) and an 5′-*H*-phosphonate **2c** (0.757 g, 0.96 mmol for **7cc**) or **2a** (0.914 g, 1.2 mmol for **7ca**) were dried by repeated coevaporation with dry pyridine and dissolved in a mixture of dry pyridine (8 mL for **7cc**, and 10 mL for **7ca**) and dry acetonitrile (8 mL for **7cc**, and 10 mL for **7ca**). PyNTP (1.19 g, 2.4 mmol for **7cc**, and 1.5 g, 3.0 mmol for **7ca**) was added to the solution at 0 °C and mixture was stirred for 20 min at 0 °C. To the mixture, CCl_4

(1.55 mL, 16 mmol for **7cc**, and 1.93 mL, 20 mmol for **7ca**) and a 9.5 M dimethylamine aqueous solution (3.2 mL, 30.4 mmol for **7cc**, and 4 mL, 38 mmol for **7ca**) were added at 0 °C and the mixture was stirred for 1 min at 0 °C. Then, the mixture was diluted with $CHCl_3$ (100 mL for **7cc**, and 50 mL for **7ca**) and washed with saturated aqueous solutions of $NaHCO_3$ (3×100 mL for **7cc**, and 3×50 mL for **7ca**). The aqueous layers were combined and back-extracted with $CHCl_3$ (3×300 mL for **7cc**, and 3×150 mL for **7ca**). The organic layers were combined, dried over with Na_2SO_4, filtered, and concentrated under reduced pressure. The crude product was used in the next step without further purification. The residue was dried by repeated coevaporation with dry toluene and dissolved in THF (8 mL for **7cc** and 10mL for **7ca**). A mixture of a 1 M tetrabutylammonium fluoride (TBAF) THF solution (2.4 mL, 2.4 mmol for **7cc**, and 3.0 mL, 3.0 mmol for **7ca**) and acetic acid (0.137 mL, 2.4 mmol for **7cc**, and 0.17 mL, 3.0 mmol for **7ca**) was added to the solution at 0 °C and the mixture was stirred for designated time (2.5 h at 0 °C for **7cc**, 45 min at 0 °C then 30 min at rt for **7ca**). Then, the mixture was diluted with $CHCl_3$ (100 mL for **7cc**, and 50 mL for **7ca**) and washed with saturated aqueous solutions of $NaHCO_3$ (3×100 mL for **7cc**, and 3×50 mL for **7ca**). The aqueous layers were combined and back-extracted with $CHCl_3$ (3×300 mL for **7cc**, and 3×150 mL for **7ca**). The organic layers were combined, dried over with Na_2SO_4, filtered, and concentrated under reduced pressure. The crude product was used in the next step without further purification. Imidazole (0.924 g, 13.6 mmol for **7cc**, and 1.157 g, 17 mmol for **7ca**) and TEA (5.6 mL, 40 mmol for **7cc**, and 7.0 mL, 50 mmol for **7ca**) were dissolved in dry CH_2Cl_2 (40 mL for **7cc**, and 50 mL for **7ca**) under argon. PCl_3 (0.35 mL, 4 mmol for **7cc**, and 0.44 mL, 5 mmol for **7ca**) was added to the solution at rt, and the mixture was allowed to stir for 30 min. Then, the mixture was cooled to −78 °C (solution C). The crude mixture (**6cc** or **6ca**) was dried by repeated coevaporation and dissolved in dry CH_2Cl_2 (13.3 mL for **7cc**, and 16.6 mL for **7ca**) and added to the solution C at −78 °C dropwise over designated time (35 min for **7cc**, 30 min for **7ca**). The mixture was allowed to stir for 30 min at −78 °C and then designated time (1.5 h for **7cc** and 1 h for **7ca**) at rt. To the mixture, a 1.0 M TEAB buffer (60 mL for **7cc**, and 70 mL for **7ca**) was added and the mixture was allowed to stir for 5 min and then separated in a separating funnel. The aqueous layer was extracted with $CHCl_3$ (6×60 mL for **7cc**, and 3×70 mL for **7ca**). The combined organic layers were dried over with Na_2SO_4, filtered and concentrated under reduced pressure. The residue was purified by silica gel chromatography (neutral silica gel, 20 g for **7cc** and 25 g for **7ca**) using $CHCl_3$–MeOH–Et_3N as the eluent. The fractions containing **7cc** or **7ca** were collected and concentrated under reduced pressure to yield **7ca** as a TEA salt. A mixture of $CHCl_3$–pyridine (2:1, v/v, 70 mL) was added to the residue

of **7cc** and washed with 0.2 M DBU hydrogen carbonate aqueous solution (1×70 mL). The aqueous layers were combined and back-extracted with $CHCl_3$–pyridine (6×70 mL). The organic layers were combined, dried over with Na_2SO_4, filtered, and concentrated under reduced pressure to yield **7cc** as a DBU salt.

C-C 2-mer fragment bearing *H*-phosphonate monoester on 5′-OH group (7cc).

The crude mixture containing **7cc** was purified by silica gel column chromatography using $CHCl_3$–MeOH–Et_3N (98:1:1–92:7:1, v/v/v) as the eluent. After following the procedure for the salt exchange described above, **7cc** was obtained as a colorless foam (0.877 g, 0.73 mmol, 91% yield from **1c**, 4 steps).

^{1}H NMR (400 MHz, $CDCl_3$) δ 11.9–11.8 (br, 0.5H), 8.01–7.66 (m, 7H, H-6, Ar), 7.62–7.36 (m, 12H, H-5, Ar), 7.31–7.27 (m, 3H, Ar), 7.21–7.00 (m, 7H, Ar), 6.92 (d, J_{PH} = 610 Hz, 1H, P-H), 6.27 (t, J = 7.1 Hz, 1H, H-1′), 5.70–5.62 (m, 1H, H-1′), 4.51–4.33 (m, 1H, H-4′), 4.10–3.78 (m, 5H, H-4′, H-5′), 3.73–3.31 (m, 9H, H-2′, H-3′, DBU), 3.10 (t, J = 13.5 Hz, 1H, H-3′), 2.88–2.84 (m, 2H, DBU), 2.77–2.27 (m, 8H, H-2′, H-3′, -N(CH_3)$_2$), 2.01–1.94 (m, 2H, DBU), 1.77–1.64 (m, 6H, DBU), 1.55–1.24 (m, 2H, H-2′, H-3′); ^{13}C{^{1}H}NMR (101 MHz, $CDCl_3$) δ 167.1, 167.0 (-CONH-), 165.8 (C-DBU), 162.6, 162.5, 162.4 (C-4, diastereomers), 154.3, 154.1, 154.0 (C-2, diastereomers), 144.6, 144.2, 144.0 (C-6, diastereomers), 135.2, 133.0, 132.9, 132.8, 132.7, 128.9, 128.6, 128.5, 128.5, 127.8, 127.7, 126.3, 121.3 (Ar), 97.3, 97.0, 96.9 (C-5, diastereomers), 82.0, 81.6 (C-1′ (3′-downstream), diastereomers), 81.4 (d, $^3J_{PC}$ = 6.7 Hz, C-1′ (5′-upstream), one of diastereomers), 81.1 (d, $^3J_{PC}$ = 7.7 Hz, C-1′ (5′-upstream), one of diastereomers), 76.4, 75.3, 75.2, 75.1 (C-4′, diastereomers), 65.4, 63.3 (C-5′), 54.1 (C-DBU), 52.2 (d, $^2J_{PC}$ = 7.7 Hz, C-2′ (5′-upstream)), 48.6 (C-3′ (3′-downstream)), 48.4 (C-DBU), 47.7, 47.5 (C-2′ (3′-downstream), diastereomers), 44.7 (d, $^2J_{PC}$ = 4.8 Hz, C-3′ (5′-upstream)), 37.7 (C-DBU), 36.5, 36.4 (d, $^2J_{PC}$ = 3.9 Hz, -N(CH$_3$)$_2$, diastereomers), 32.0 (C-DBU), 28.7 (C-DBU), 26.5 (C-DBU), 23.8 (C-DBU), 19.2 (C-DBU); ^{31}P{^{1}H}NMR (162 MHz, $CDCl_3$) δ 16.9, 16.7 (phosphorodiamidate), 5.2 ($^1J_{PH}$ = 615 Hz), 5.1 ($^1J_{PH}$ = 615 Hz) (*H*-phosphonate monoester). ESI-MS *m/z* calcd for $C_{53}H_{54}N_9O_{11}P_2$ [M-H-DBU]$^-$, 1054.3424; found 1054.3413.

C-A 2-mer fragment bearing *H*-phosphonate monoester on 5′-OH group (7ca).

The crude mixture containing **7ca** was purified by silica gel column chromatography using $CHCl_3$–MeOH–Et_3N (98:1:1–94:5:1, v/v/v) as the eluent. **7ca** was obtained as a colorless foam (1.034 g, 0.88 mmol, 88% yield from **1c**, 4 steps).

^{1}H NMR (400 MHz, $CDCl_3$) δ 8.79, 8.74 (s, 1H, H-2 (adenosine), diastereomers), 8.18

(s, 0.5H, H-8 (adenosine), one of diastereomers), 8.14 (s, 0.5H, H-8 (adenosine), one of diastereomers), 8.10–7.97 (m, 2H, -N$\underline{\text{H}}$CO-, Ar), 7.96–7.85 (m, 2.5H, -NHCO-, Ar, H-6 (cytidine), one of diastreomers), 7.79 (d, J = 7.3 Hz, 0.5H, H-6 (cytidine), one of diastereomers), 7.61–7.14 (m, 23H, Ar, H-5 (cytidine)), 6.88 (d, J_{PH} = 620 Hz, 0.5H, P-$\underline{\text{H}}$, one of diastereomers), 6.85 (d, J_{PH} = 629 Hz, 0.5H, P-$\underline{\text{H}}$, one of diastereomers), 6.41 (t, J = 8.7 Hz, 1H, H-1′ (adenosine)), 5.65 (d, J = 9.6 Hz, 0.5H, H-1′ (cytidine), one of diastereomers), 5.56 (d, J = 9.2 Hz, 0.5H, H-1′ (cytidine), one of diastereomers), 4.57–4.38 (m, 1H, H-4′), 4.05–3.13 (m, 9H, H-2′, H-3′, H-4′, H-5′), 3.00 (q, J = 6.9 Hz, 6H, -$NC\underline{H}_2CH_3$)), 2.82–2.23 (m, 8H, -$N(C\underline{H}_3)_2$, H-2′, H-3′), 1.89 (t, J = 10.5 Hz, 1H, H-2′ (adenosine)), 1.63 (q, J = 10.5 Hz, 1H, H-3′), 1.29 (t, J = 7.3 Hz, 9H, -$NCH_2C\underline{H}_3$); $^{13}C\{^1H\}$NMR (101 MHz, $CDCl_3$) δ 167.1, 166.9 (-CONH-, diastereomers), 165.2, 165.0 (-CONH-, diastereomers), 162.4, 162.3 (C-4 (cytidine), diastereomers), 153.9 (C-2 (cytidine)), 152.6, 152.5 (C-2 (adenosine), diastereomers), 151.3, 151.3 (C-4 (adenosine), diastereomers), 149.8, 149.4 (C-6 (adenosine), diastereomers), 144.1 (C-6 (cytidine)), 141.2, 141.0 (C-8 (adenosine), diastereomers), 133.6, 133.1, 133.0, 132.6, 132.5, 129.5, 129.1, 128.9, 128.6, 128.5, 128.1, 127.9, 127.7, 126.5 (Ar), 123.3, 122.9 (C-5 (adenosine), diastereomers), 96.8 (C-5 (cytidine)), 81.4 (d, $^3J_{PC}$ = 7.7 Hz, C-1′ (cytidine), one of diastereomers), 81.3 (d, $^3J_{PC}$ = 8.7 Hz, C-1′ (cytidine), one of diastereomers), 80.2, 79.7 (C-1′ (adenosine), diastereomers), 76.8, 76.8 (C-4′, diastereomers), 75.3, 75.2 (C-4′, diastereomers), 65.4 (d, $^2J_{PC}$ = 3.9 Hz, C-5′), 63.5, 63.3 (d, $^2J_{PC}$ = 2.9 Hz, C-5′, diastereomers), 52.7, 52.6 (C-2′ (adenosine)), 48.8, 48.7 (C-3′, diastereomers), 47.8, 47.6 (C-2′ (cytidine), diastereomers), 45.4 (-$N\underline{C}H_2CH_3$), 45.0, 44.8 (C-3′, diastereomers), 36.6, 36.5 (d, $^2J_{PC}$ = 3.9 Hz, -$N(\underline{C}H_3)_2$, diastereomers), 8.5 (-$NCH_2\underline{C}H_3$); $^{31}P\{^1H\}$NMR (162 MHz, $CDCl_3$) δ 17.0, 16.6 (phosphorodiamidate), 5.2 ($^1J_{PH}$ = 620 Hz), 5.0 ($^1J_{PH}$ = 620 Hz) (*H*-phosphonate monoester). ESI-MS *m/z* calcd for $C_{54}H_{54}N_{11}O_{10}P_2$ [M-H-TEA]$^-$, 1078.3536; found 1078.3527.

HPLC analysis of the synthesis of 4-mer (9cccc) using the fragment condensation. (Scheme 3)

Scheme S 5

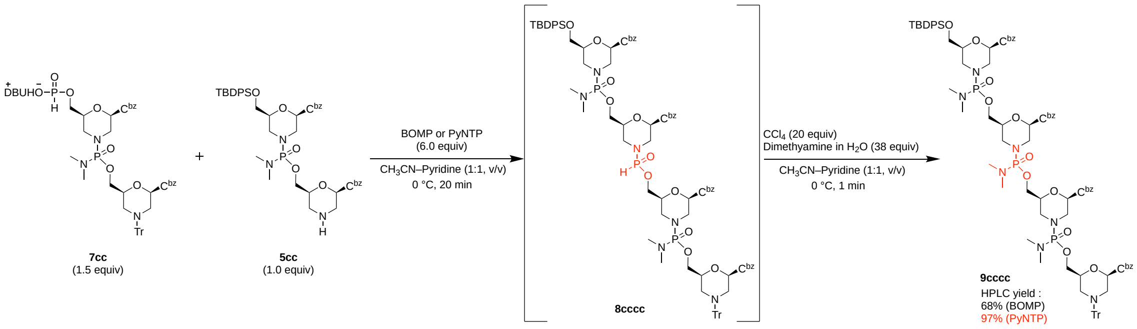

A 2-mer fragment bearing the 3′-NH group **5cc** (4.94 mg, 5 µmol) and a 2-mer fragment bearing an *H*-phosphonate monoester on 5′-OH group **7cc** (9.06 mg, 7.5 µmol) was dried by repeated coevaporation with dry pyridine and dissolved in a mixture of dry pyridine (50 µL) and acetonitrile (50 µL). A condensing reagent (30 µmol) was added to the solution at 0 °C and the mixture was stirred for 20 min at 0 °C. To the mixture, CCl_4 (10 µL, 100 µmol) and a 9.5 M dimethylamine aqueous solution (20 µL, 190 µmol) was added at 0 °C and the mixture was stirred for 1 min at 0 °C. Then, the mixture was diluted with $CHCl_3$ (3 mL) and coevaporated with $CHCl_3$ (3×3 mL), toluene (2×3 mL). The residue was analyzed by RP-HPLC. RP-HPLC was performed with a linear gradient of 0%–60% MeCN for 60 min in a 0.1 M triethylammonium acetate buffer (pH 7.0) at 50 °C at a flow rate of 0.5 mL/min using a C18 column (100 Å, 3.9 mm×150 mm). The condensation yields were estimated by the area rations of the 4-mer **9cccc** to unreacted 2-mer **5cc**.

Synthesis of 4-mer fragments (10cccc, 10gtca, 12cccc, 12gtca)

Scheme S 6

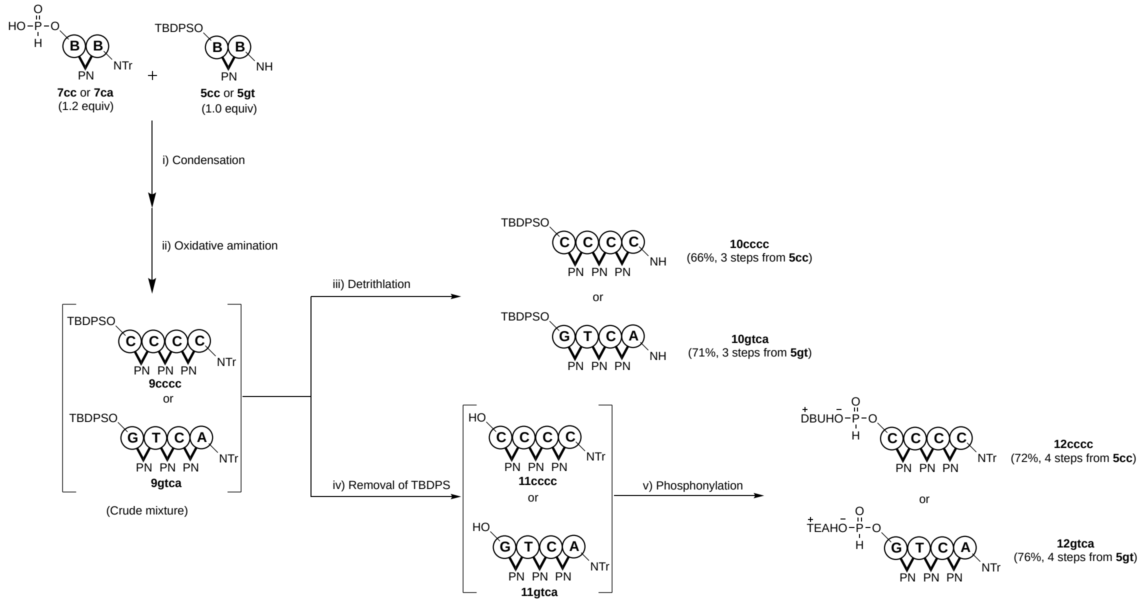

Four kinds of 4-mer fragments were synthesized using same procedures as for the synthesis of 2-mer fragments described above.

General procedure for the synthesis of the 4-mer fragments bearing 3′-NH group (10cccc or 10gtca).

The 2-mer fragment bearing the 3′-NH group **5cc** (0.198 g, 0.20 mmol) or **5gt** (0.192 g, 0.20 mmol) and the 2-mer fragment bearing an *H*-phosphonate monoester on the 5′-OH group **7cc** (0.289 g, 0.24 mmol) or **7ca** (0.283 g, 0.24 mmol) were dried by repeated coevaporation with dry pyridine and dissolved in a mixture of dry pyridine (2 mL) and dry acetonitrile (2 mL). PyNTP (0.299 g, 0.60 mmol) was added to the solution at 0 °C and mixture was stirred for 20 min at 0 °C. To the mixture, CCl_4 (0.39 mL, 4.0 mmol) and a 9.5 M dimethylamine aqueous solution (0.8 mL, 7.6 mmol) were added at 0 °C and the mixture was stirred for the designated time (20 min for **10cccc**, 1 min for **10gtca**) at 0 °C. Then, the mixture was diluted with $CHCl_3$ (30 mL) and washed with saturated aqueous solutions of $NaHCO_3$ (3×30 mL). The aqueous layers were combined and back-extracted with $CHCl_3$ (3×90 mL). The organic layers were combined, dried over with Na_2SO_4, filtered, and concentrated under reduced pressure. The residue was dissolved in CH_2Cl_2 (4 mL) (solution A). 3-cyanopyridine (0.208 g, 2.0 mmol) and CF_3COOH (0.15 mL, 2.0 mmol) were dissolved in a mixture of CH_2Cl_2–CF_3CH_2OH (6:4, v/v, 4 mL) (solution B). To the solution A, the solution B was added at rt and allowed to stir for designated time (1 h for **10cccc**, 30 min for **10gtca**). The mixture was diluted with $CHCl_3$ (30 mL) and

washed with saturated aqueous solutions of $NaHCO_3$ (3×30 mL). The aqueous layers were combined and back-extracted with $CHCl_3$ (3×90 mL). The combined organic layers were dried over with Na_2SO_4, filtered and concentrated under reduced pressure. The residue was purified by silica gel chromatography (neutral silica gel, 16 g) using $CHCl_3$–MeOH as the eluent. The fractions containing **10cccc** or **10gtca** were collected and concentrated under reduced pressure to yield **10cccc** or **10gtca**.

C-C-C-C 4-mer fragments bearing 3′-NH group (10cccc)

The crude mixture containing **10cccc** was purified by silica gel column chromatography using $CHCl_3$–MeOH (97:3–84:16, v/v) as the eluent. **10cccc** was obtained as a colorless foam (0.241 g, 0.13 mmol, 66 % yield from **5cc**, 3 steps).

^{1}H NMR (400 MHz, $CDCl_3$) δ 8.26–7.34 (m, 38H, Ar, H-5, H-6), 5.96–5.63 (m, 4H, H-1′), 4.33–3.71 (m, 13H, H-2′, H-4′, H-5′), 3.62–3.26 (m, 3H, H-2′, H-3′), 3.13–2.30 (m, 30H, H-2′, H-3′, -N(C$\underline{H}_3$)$_2$), 1.06 (s, 9H, -C(C$\underline{H}_3$)$_3$); ^{13}C{^{1}H}NMR (101 MHz, $CDCl_3$) δ 167.3, 166.7 (m, -CONH-, diastereomers), 162.9, 162.8, 162.6, 162.5, 162.2 (C-4, diastereomers), 154.3, 154.2 (C-2, diastereomers), 144.9, 144.7, 144.6, 144.2, 143.5 (C-6, diastereomers), 135.5, 135.5, 133.0, 132.9, 132.8, 132.6, 129.9, 128.8, 128.7, 128.1, 128.0, 128.0, 127.8, 127.8, 127.7, 127.6 (Ar), 97.1 (C-5), 82.4, 82.1, 81.7 (C-1′, diastereomers), 77.9, 76.1 (C-4′, diastereomers), 65.4, 65.0, 64.6, 64.2, 64.1 (C-5′, diastereomers), 49.5, 49.2, 48.3, 48.1, 47.9, 47.8, 47.6, 47.5 (C-2′, diastereomers), 45.8, 45.7, 45.6, 45.1, 44.6, 44.4, 44.2 (C-3′, diastereomers), 36.7, 36.4 (-N($\underline{C}$H$_3$)$_2$, diastereomers), 26.7 (-C($\underline{C}$H$_3$)$_3$), 19.2, 19.2 (-$\underline{C}$(CH$_3$)$_3$, diastereomers); ^{31}P{^{1}H}NMR (162 MHz, $CDCl_3$) δ 17.2–16.2. ESI-MS *m/z* calcd for $C_{86}H_{104}N_{19}O_{19}P_3Si$ [M + 2 H]$^{2+}$, 913.8364; found 913.8364.

G-T-C-A 4-mer fragment bearing 3′-NH group (10gtca)

The crude mixture containing **10gtca** was purified by silica gel column chromatography using $CHCl_3$–MeOH (97:3–82:18, v/v) as the eluent. **10gtca** was obtained as a colorless foam (0.252 g, 0.14 mmol, 71% yield from **5gt**, 3 steps).

^{1}H NMR (400 MHz, $CDCl_3$) δ 8.82–8.74 (m, 1H), 8.82–8.31 (m, 4H), 8.14–7.85 (m, 6H), 7.66–7.32 (m, 15H), 7.27–7.17 (m, 1H, H-6 (thymidine)), 6.09 (t, J = 9.2 Hz, 1H, H-1′), 5.91–5.60 (m, 3H, H-1′), 4.83–4.70 (m, 2H, -OC$\underline{H}_2$CH$_2$CN), 4.45–3.66 (m, 14H, H-2′, H-4′, H-5′), 3.62–2.95 (m, 10H, H-2′, H-3′, -C$\underline{H}$(CH$_3$)$_2$, -CH$_2$C$\underline{H}_2$CN), 2.92–2,52 (m, 25H, -N(C$\underline{H}_3$)$_2$, H-2′, H-3′), 1.77–1.61 (m, 3H, 5-CH$_3$ (thymidine)), 1.28–1.23 (m, 6H, -CH(C$\underline{H}_3$)$_2$), 1.03 (s, 9H, -C(C$\underline{H}_3$)$_3$); ^{13}C{^{1}H}NMR (101 MHz, $CDCl_3$) δ 175.7, 175.6 (-NHCO-, diastereomers), 167.6, 166.9, 166.8, 166.7, 166.6, 166.3, 165.4, 165.3, 165.0,

164.9, 164.6, 164.5, 164.1, 164.0 (C-4 (thymidine), diastereomers), 162.7, 162.6, 162.6, 162.5, 162.5, 162.4 (C-4 (cytidine), diastereomers), 159.6 (C-6 (guanosine)), 154.1 (C-2 (cytidine)), 152.6, 152.4, 151.9 (C-2 (thymidine)), 151.5, 151.4, 151.1, 150.2, 149.9, 150.2, 150.2, 149.9, 149.8, 145.1, 145.0, 144.6, 144.5, 144.4, 144.3, 144.1, 144.0, 143.9, 141.6, 141.4, 139.5, 135.4, 134.7, 134.6 (C-6 (thymidine), diastereomers), 133.7, 133.1, 132.8, 132.7, 132.5, 132.4, 132.2, 130.8, 129.8, 128.9, 128.8, 128.7, 128.6, 128.5, 128.3, 128.1, 128.0, 127.7, 127.4 (Ar), 123.0, 117.4, 116.9, 116.9 (-C̲N, diastereomers), 110.9 (C-5 (thymidine)), 97.0, 97.0, 96.9, 96.7 (C-5 (cytidine), diastereomers), 81.4, 81.3, 81.2, 80.4, 80.1, 79.9, 79.7, 79.5 (C-1′, diastereomers), 76.4, 76.2, 76.0, 75.9, 75.8 (C-4′, diastereomers), 65.5, 65.2, 65.0, 64.7, 64.0, 64.0 (C-5′, diastereomers), 61.6 (-OC̲H_2CH_2-), 49.6, 49.5, 49.4, 49.3, 49.0, 49.0, 48.3, 48.1, 48.0, 47.7, 47.5, 47.3, 47.1 (C-2′, diastereomers), 45.7, 45.5, 45.3, 44.9, 44.6, 44.6, 44.3, 44.0 (C-3′, diastereomers), 36.9, 36.8, 36.7, 36.6, 36.6 (-N(C̲H_3)$_2$, diastereomers), 35.7, 35.6 (-C̲H(CH_3)$_2$, diastereomers), 26.7 (-C(C̲H_3)$_3$), 19.3 (-CH(C̲H_3)$_2$), 19.2 (-C̲(CH_3)$_3$), 18.0 (-CH_2C̲H_2CN), 12.2, 12.2, 12.0 (5-C̲H_3 (thymidine), diastereomers); $^{31}P\{^1H\}$NMR (162 MHz, $CDCl_3$) δ 17.4–15.6. ESI-MS *m/z* calcd for $C_{82}H_{106}N_{23}O_{18}P_3Si$ $[M+2H]^{2+}$, 910.8529; found 910.8528.

General procedure for the synthesis of the 4-mer fragments bearing *H*-phosphonate monoester on 5′-OH (12cccc or 12gtca).

The 2-mer fragment bearing the 3′-NH group **5cc** (0.198 g, 0.20 mmol) or **5gt** (0.192 g, 0.2 mmol) and the 2-mer fragment bearing an *H*-phosphonate monoester on the 5′-OH group **7cc** (0.289 g, 0.24 mmol) or **7ca** (0.283 g, 0.24 mmol) were dried by repeated coevaporation with dry pyridine and dissolved in a mixture of dry pyridine (2 mL) and dry acetonitrile (2 mL). PyNTP (0.299 g, 0.60 mmol) was added to the solution at 0 °C and mixture was stirred for 20 min at 0 °C. To the mixture, CCl_4 (0.39 mL, 4.0 mmol) and a 9.5 M dimethylamine aqueous solution (0.8 mL, 7.6 mmol) were added at 0 °C and the mixture was stirred for the designated time (20 min for **12cccc**, 1 min for **12gtca**) at 0 °C. Then, the mixture was diluted with $CHCl_3$ (30 mL) and washed with saturated aqueous solutions of $NaHCO_3$ (3×30 mL). The aqueous layers were combined and back-extracted with $CHCl_3$ (3×90 mL). The organic layers were combined, dried over with Na_2SO_4, filtered, and concentrated under reduced pressure. The crude product was used in the next step without further purification. The residue was dried by repeated coevaporation with dry toluene and dissolved in dry THF (2 mL). A mixture of a 1 M tetrabutylammonium fluoride (TBAF) THF solution (0.6 mL, 0.60 mmol) and acetic acid (0.034 mL, 0.60 mmol) were added to the solution at 0 °C and the mixture was stirred for designated time (1 h at 0 °C for **12cccc**, 30 min at 0 °C then 1 h at rt for **12gtca**). For the synthesis of

12cccc, a mixture of a 1 M tetrabutylammonium fluoride (TBAF) THF solution (0.4 mL, 0.40 mmol) and acetic acid (0.023 mL, 0.40 mmol) were added to the solution at 0 °C and the mixture was stirred for 20 min. Then, the mixture was diluted with $CHCl_3$ (30 mL) and washed with saturated aqueous solutions of $NaHCO_3$ (3×30 mL). The aqueous layers were combined and back-extracted with $CHCl_3$ (3×90 mL). The organic layers were combined, dried over with Na_2SO_4, filtered, and concentrated under reduced pressure. The crude product was used in the next step without further purification. Imidazole (0.231 g, 3.4 mmol) and TEA (1.4 mL, 10 mmol for **12cccc**) or *N*-methylmorpholine (1.1 mL, 10 mmol for **12gtca**) were dissolved in dry CH_2Cl_2 (10 mL) under argon. PCl_3 (0.087 mL, 1.0 mmol) was added to the solution at rt, and the mixture was allowed to stir for 30 min. Then, the mixture was cooled to −78 °C (solution C). The crude mixture (**11cccc** or **11gtca**) was dried by repeated coevaporation and dissolved in dry CH_2Cl_2 (3.3 mL) and added to the solution C at −78 °C dropwise over designated time (23 min for **12cccc**, 25 min for **12gtca**). The mixture was allowed to stir for 30 min at −78 °C and then designated time (1 h for **12cccc** and 30 min for **12gtca**) at rt. To the mixture, a 1.0 M TEAB buffer (15 mL) was added, and the mixture was allowed to stir for 5 min and then separated in a separating funnel. The aqueous layer was extracted with a mixture of $CHCl_3$–pyridine (2:1, v/v) (3×20 mL for **12cccc**) or $CHCl_3$ (3×50 mL for **12gtca**). The combined organic layers were dried over with Na_2SO_4, filtered and concentrated under reduced pressure. The residue was purified by silica gel chromatography (neutral silica gel, 16 g) using $CHCl_3$–MeOH–Et_3N as the eluent. The fractions containing **12cccc** or **12gtca** were collected and concentrated under reduced pressure to yield **12gtca** as a TEA salt. A mixture of $CHCl_3$–pyridine (2:1, v/v, 30 mL) was added to the residue of **12cccc** and washed with a 0.2 M DBU hydrogen carbonate aqueous solution (1×30 mL). The aqueous layers were combined and back-extracted with $CHCl_3$–pyridine (3×30 mL). The organic layers were combined, dried over with Na_2SO_4, filtered, and concentrated under reduced pressure to yield **12cccc** as a DBU salt.

C-C-C-C 4-mer fragment bearing *H*-phosphonate monoester on 5′-OH group (12cccc)

The crude mixture containing **12cccc** was purified by silica gel column chromatography using $CHCl_3$–MeOH–Et_3N (96:3:1–89:10:1, v/v/v) as the eluent. **12cccc** was obtained as a colorless foam (0.293 g, 0.143 mmol, 72% yield from **5cc**, 4 steps).

^{1}H NMR (400 MHz, $CDCl_3$) δ 11.9 (s, 1H, $HDBU^+$), 8.18–7.63 (m, 14H, Ar, H-6), 7.63–7.29 (m, 23H, Ar, H-5), 7.26–7.07 (m, 6H, Ar), 6.88 (d, J_{PH} = 614 Hz, 1H, P-H), 6.27 (t, *J* = 10.5 Hz, 1H, H-1′), 5.87–5.60 (m, 3H, H-1′), 4.50–3.68 (m, 10H, H-2′, H-4′, H-5′), 3.61–3.19 (m, 13H, H-2′, H-3′, DBU), 3.13–3.00 (m, 1H, H-3′), 2.97–2.37 (m, 28H,

H-2′, H-3′, -N(C$\underline{H}_3$)$_2$, DBU), 2.04–1.97 (m, 2H, DBU), 1.77–1.65 (m, 6H, DBU), 1.58–1.32 (m, 2H, H-2′, H-3′); ^{13}C{^{1}H}NMR (101 MHz, $CDCl_3$) *δ* 167.0, 166.9 (-NHCO-), 166.2 (C-DBU), 163.2, 162.9, 162.7, 162.6 (C-4), 154.5, 154.3, 154.3 (C-2), 144.8, 144.7, 144.5, 144.4, 144.3, 144.2, 144.1 (C-6, diastereomers), 133.0, 132.9, 129.1, 129.0, 128.7, 128.5, 128.3, 128.2, 128.1, 127.9, 126.5 (Ar), 97.2, 97.1, 97.0, 96.9 (C-5), 82.0, 81.7, 81.4, 81.2 (C-1′), 77.8, 76.4, 76.1, 76.0, 75.4, 75.4, 75.3 (C-4′, diastereomers), 65.7, 65.6, 65.1, 65.0, 65.0, 64.9, 64.8, 63.4, 63.3 (C-5′, diastereomers), 54.3 (C-DBU), 52.4, 52.3, 52.3 (C-2′, diastereomers), 48.9, 48.8, 48.8, 48.7 (C-3′, diastereomers), 48.6 (C-DBU), 47.9, 47.5, 47.4, 47.0 (C-2′, diastereomers), 45.4, 45.1, 44.9, 44.9, 44.7, 44.6, 44.2 (C-3′, diastereomers), 42.8, 42.5 (C-2′, diastereomers), 37.9 (C-DBU), 36.6 (-N($\underline{C}H_3$)$_2$), 32.1 (C-DBU), 29.0 (C-DBU), 26.8 (C-DBU), 24.0 (C-DBU), 19.5 (C-DBU); ^{31}P{^{1}H}NMR (162 MHz, $CDCl_3$) *δ* 17.1–16.5 (phosphorodiamidate), 5.3, 5.1 ($^1J_{PH}$ = 616, 611 Hz) (*H*-phosphonate monoester). ESI-MS *m/z* calcd for $C_{89}H_{97}N_{19}O_{21}P_4$ [M-2H-DBU]$^{2-}$, 945.8034; found 945.8037.

G-T-C-A 4mer fragment bearing *H*-phosphonate monoester on 5′-OH group (12gtca)

The crude mixture containing **12gtca** was purified by silica gel column chromatography using $CHCl_3$–MeOH–Et_3N (96.5:3:0.5–87.5:12:0.5, v/v/v) as the eluent. **12gtca** was obtained as a colorless foam 0.303 g, 0.152 mmol, 76% yield from **5gt**, 4 steps).

^{1}H NMR (400 MHz, $CDCl_3$) δ 10.4–9.65 (m, 0.5H, -CONH-, one of diastereomers), 8.92–8.34 (m, 2H), 8.32–7.74 (m, 7H, H-6 (cytidine)), 7.70–7.34 (m, 16H, Ar), 7.27–7.11 (m, 6H, H-6 (thymidine)), 6.85 (d, J_{PH} = 620 Hz, 1H, P-$\underline{H}$), 6.46 (d, *J* = 9.2 Hz, 1H, H-1′), 5.85–5.56 (m, 3H, H-1′), 4.77 (s, 2H, -OC$\underline{H}_2$CH$_2$-), 4.58–4.43 (m, 1H, H-4′), 4.12–3.72 (m, 10H, H-4′, H-5′), 3.55–3.05 (m, 12H, H-2′, H-3′, -C$\underline{H}$(CH$_3$)$_2$), 3.05–2.74 (m, 9H, H-2′, -CH$_2$C$\underline{H}_2$CN, -NC$\underline{H}_2$CH$_3$), 2.73–2.42 (m, 21H, H-2′, H-3′, -N(C$\underline{H}_3$)$_2$), 2.00–1.53 (m, 5H, H-2′, H-3′, 5-C$\underline{H}_3$ (thymidine)), 1.30–1.25 (m, 15H, -NCH$_2$C$\underline{H}_3$, -CH(C$\underline{H}_3$)$_2$); ^{13}C{^{1}H}NMR (101 MHz, $CDCl_3$) *δ* 175.9, 175.8, 175.7, 175.6, 175.5, 175.4, 175.3 (-CONH-), 168.4, 168.3, 167.1, 167.0, 166.7, 166.4, 166.1, 166.0, 165.7, 165.5, 165.4, 165.3, 165.2, 165.0, 164.3, 163.9 (C-4 (thymidine), diastereomers), 162.5, 162.4, 162.3, 162.2 (C-4 (cytidine), diastereomers), 159.5 (C-6 (guanosine)), 154.1, 153.9, 153.9, 153.7 (C-2 (cytidine), diastereomers), 152.6, 152.4, 152.0, 151.9, 151.3, 151.2, 151.1, 150.2, 150.1, 150.0, 149.8, 149.7 (C-2 (thymidine), diastereomers), 144.2, 144.1, 144.0, 143.9, 143.5, 143.5, 141.7, 141.5, 141.3, 141.1, 141.0, 141.0, 140.9, 140.7, 140.6, 140.4, 140.2, 140.0, 139.9, 139.8, 139.7, 138.9, 138.8, 135.1, 134.9, 134.7 (C-6 (thymidine),

diastereomers), 133.9, 133.6, 133.5, 133.1, 133.0, 133.0, 132.8, 132.5, 132.4, 132.1, 129.6, 129.4, 129.0, 128.8, 128.7, 128.6, 128.6, 128.3, 128.2, 128.1, 127.9, 127.4, 126.5, 126.3, 123.2, 123.1, 123.0, 122.9, 122.7, 122.5, 117.4, 116.9 (-CN), 111.0, 110.9, 110.8, 110.8 (C-5 (thymidine), diastereomers), 97.5, 97.4, 97.2, 97.2, 97.0, 97.0, 96.8 (C-5 (cytidine), diastereomers), 81.3, 81.1, 81.1, 80.9, 80.8, 80.8, 80.7, 80.4, 80.2, 80.1, 79.8, 79.6, 79.5, 79.4, 79.2 (C-1′, diastereomers), 76.4, 76.2, 76.0, 75.8, 75.7, 75.4, 75.3, 75.2, 75.1, 75.0, 74.7, 74.7 (C-4′, diastereomers), 65.5, 65.4, 65.3, 65.1, 65.1, 64.9, 64.8, 63.3, 63.2 (C-5′, diastereomers), 61.5 ($-O\underline{C}H_2CH_2-$), 52.8, 52.7, 52.5, 52.4, 52.4 (C-2′, diastereomers), 48.7, 48.6, 48.5, 48.2, 48.0, 47.9, 47.7, 47.5, 47.3, 47.2, 47.1, 46.7 (C-2′, C-3′, diastereomers), 45.3 ($-N\underline{C}H_2CH_3$), 45.2, 45.0, 44.7, 44.5, 44.4, 44.3, 44.1, 43.7 (C-2′, C-3′, diastereomers), 40.2 (C-2′), 36.7, 36.5, 36.2 ($-N(CH_3)_2$, diastereomers), 35.7, 35.6 ($-\underline{C}H(CH_3)_2$, diastereomers), 19.3 ($-CH(\underline{C}H_3)_2$), 18.0 ($-CH_2\underline{C}H_2CN$), 12.4, 12.2, 12.1, 12.1 (5-CH_3 (thymidine), diastereomers), 8.4 ($-NCH_2\underline{C}H_3$); $^{31}P\{^1H\}$NMR (162 MHz, $CDCl_3$) δ 17.3–15.6 (phosphorodiamidate), 5.4 ($^1J_{PH}$ = 620 Hz) (*H*-phosphonate monoester). ESI-MS *m/z* calcd for $C_{85}H_{100}N_{23}O_{20}P_4$ $[M-H-TEA]^{1-}$, 1886.6471; found 1886.6472.

General procedure for the synthesis of 6-mer and 8-mer (15, 16, 17)

Scheme S 7

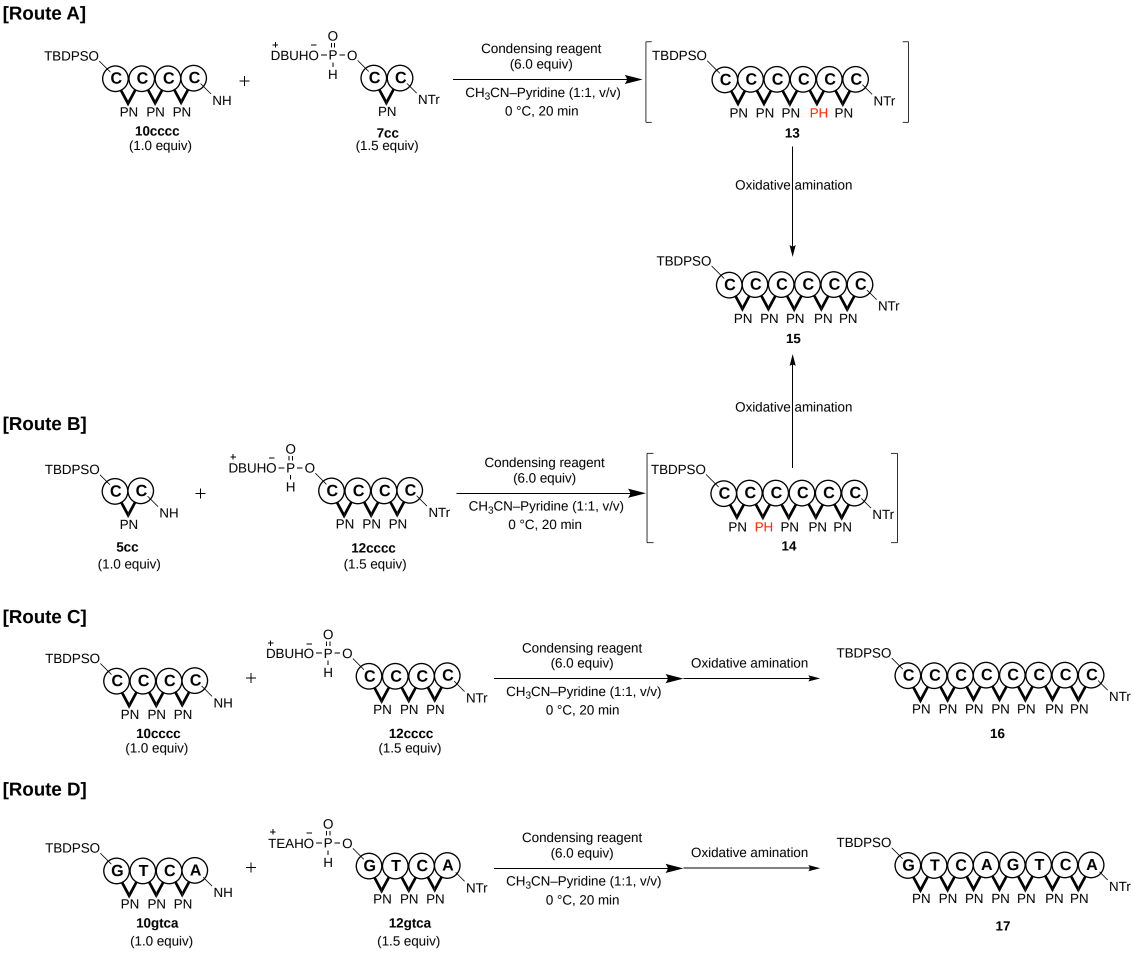

A 2-mer or 4mer fragment bearing the 3′-NH group (**5cc**, **10cccc**, or **10gtca**, 5 μmol**)** and a 2-mer or 4-mer fragment bearing an *H*-phosphonate monoester on the 5′-OH group (**7cc**, **12cccc**, or **12gtca**, 7.5 μmol) were dried by repeated coevaporation with dry pyridine and dissolved in a mixture of dry pyridine (50 μL) and acetonitrile(50 μL). A condensing reagent (30 μmol) was added to the solution at 0 °C and the mixture was stirred for 20 min at 0 °C. To the mixture, CCl_4 (10 μL, 100 μmol) and a 9.5 M dimethylamine aqueous solution (20 μL, 190 μmol) were added at 0 °C and the mixture was stirred for 1 min at 0 °C. Then, the mixture was diluted with $CHCl_3$ (3 mL) and coevaporated with $CHCl_3$ (3×3mL), toluene (2×3 mL). The one tenth of residue were analyzed by RP-HPLC. RP-HPLC was performed with a linear gradient of 0%–60% MeCN for 60 min in a 0.1 M triethylammonium acetate buffer (pH 7.0) at 50 °C at a flow rate of 0.5 mL/min using a C18 column (100 Å, 3.9 mm×150 mm). The condensation yields were estimated by the area ratios of the 6-mer or 8-mer to the unreacted 2-mer or 4-mer fragments bearing an amino group.

C-C-C-C-C-C 6-mer (**15**)

ESI-MS *m/z* calcd for $C_{141}H_{160}N_{29}O_{29}P_5Si$ $[M+2H]^{2+}$, 1454.020; found 1454.352.

C-C-C-C-C-C-C-C 8-mer (**16**)

ESI-MS *m/z* calcd for $C_{177}H_{204}N_{39}O_{39}P_7Si$ $[M+2H]^{2+}$, 1873.156; found 1874.634.

G-T-C-A-G-T-C-A 8-mer (**17**)

ESI-MS *m/z* calcd for $C_{169}H_{211}N_{47}O_{37}P_7Si$ $[M+3H]^{3+}$, 1245.1330; found 1245.1357.

General procedure for the removal of protecting group of 8-mer

Scheme S 8

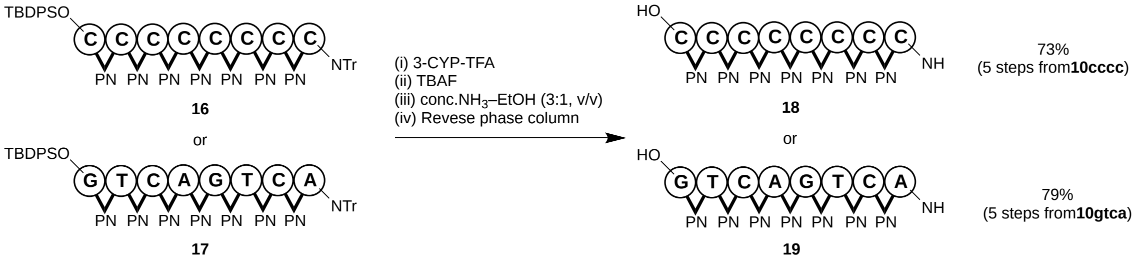

The nine tenths of the crude mixture of **16** or **17** (5 μmol) was dissolved in CH_2Cl_2 (0.5 mL) (solution D). 3-Cyanopyridine (0.021 g, 200 μmol) and CF_3COOH (15 μL, 200 μmol) were dissolved in a mixture of CH_2Cl_2–CF_3CH_2OH (3:2, v/v) (0.5 mL) (solution E). To the solution D, the solution E was added at rt and allowed to stir for 1 h. The mixture was concentrated under reduced pressure. Then, the residue was dissolved in THF (0.2 mL). A 1 M tetrabutylammonium fluoride (TBAF) THF solution (0.2 mL, 200 μmol) was added to the solution at rt and the mixture was stirred for 2 h at rt. The mixture was concentrated under reduced pressure. The residue was treated with concentrated aqueous NH_3–EtOH (3:1, v/v, 2 mL) at 55 °C for 16 h. The mixture was diluted with H_2O (10 mL) and washed with Et_2O (1×10 mL). The organic layer was back-extracted with H_2O (5 mL). The aqueous layers were combined and concentrated under reduced pressure. The residue was analyzed by RP-HPLC. RP-HPLC was performed with a linear gradient of 0%–60% MeCN for 60 min in a 0.1 M triethylammonium acetate buffer (pH 7.0) at 50 °C at a flow rate of 0.5 mL/min using a C18 column (100 Å, 3.9 mm×150 mm). The crude 8-mer (**18** and **19**) was purified by silica gel column chromatography. Column chromatography was carried out on the Yamazen UNIVERSAL Premium ODS column (M size) using an automated flash chromatography system W-prep 2XY (Yamazen Corporation).

8-mer (CCCCCCCC) (18)

The chromatography was performed with a linear gradient of 0%–30% MeCN in a 0.1 M TEAA buffer (pH 7.0). The fractions containing **18** were collected and concentrated under reduced pressure. **18** was obtained as a colorless foam (7.2 mg, 3.0 μmol, 73% yield from **10cccc**, 5 steps).

^{1}H NMR (600 MHz, D_2O) δ 7.60–7.75 (m, 8H, C-6), 6.01–5.62 (m, 16H, H-5, H-1′), 4.36–3.24 (m, 24H, H-4′, H-5′), 3.53–3.25 (m, 16H, H-2′, H-3′), 3.08–2.74 (m, 16H, H-2′, H-3′), 2.71–2.63 (m, 42H, -N(C<u>H</u>3)2); ^{13}C {^{1}H} NMR (151 MHz, D_2O) δ 166.4, 166.3 (C-4), 157.1, 156.8 (C-2), 142.0, 141.9, 141.7 (C-6), 97.1, 96.7 (C-5), 81.2, 81.0, 80.0, 79.8 (C-1′), 78.2, 77.9, 76.4, 76.0 (C-4′), 66.3, 66.0 (C-5′), 47.3, 47.1, 45.9, 44.6, 44.2, 43.3, 43.3 (C-2′, C-3′), 36.4 (-N($\underline{C}H_3$)$_2$); ^{31}P {^{1}H}NMR (243 MHz, D_2O) δ 19.2–18.8.

HRMS (ESI-TOF) *m/z* calcd for $C_{86}H_{143}N_{39}O_{31}P_7$ $[M+3H]^{3+}$, 811.6320; found 811.6319.

8-mer (GTCAGTCA) (19)

The chromatography was performed with a linear gradient of 0%–30% MeCN in 0.1 M TEAA buffer (pH 7.0). The fractions containing **19** were collected and concentrated under reduced pressure. **19** was obtained as a colorless foam (8.3 mg, 3.2 μmol, 79% yield from **10gtca**, 5 steps).

^{1}H NMR (600 MHz, D_2O) δ 8.01–7.30 (m, 10H, H-2 (adenine), H-8 (adenine), H-8 (guanine), H-6 (thymine), H-6 (cytosine)), 5.95–5.01 (m, 10H, H-5 (cytosine), H-1′), 4.29–3.94 (16H, H-4′, H-5′), 3.70–2.91 (m, 31H, H-2′, H-3′, H-4′, H-5′), 2.78–2.53 (m, 51H, H-2′, H-3′, -N(CH_3)$_2$), 1.76–1.36 (m, 6H (5-C$\underline{H}_3$)); ^{31}P {^{1}H}NMR (243 MHz, D_2O) δ 20.0–18.5; ^{13}C {^{1}H} NMR: Due to the low solubility of **19** in water, the measurement of ^{13}C NMR was difficult.

HRMS (ESI-TOF) *m/z* calcd for $C_{92}H_{145}N_{45}O_{31}P_7$ $[M+3H]^{3+}$, 864.3100; found 864.3100.

2. ^{31}P NMR analysis of the formation of *H*-phosphonamidate linkages (Table 1)

*The NMR yields were determined by the integral ratios of the **3cc** to the other byproducts, except for derivatives that were derived from condensing reagents.

Table 1, entry 1 (PivCl)

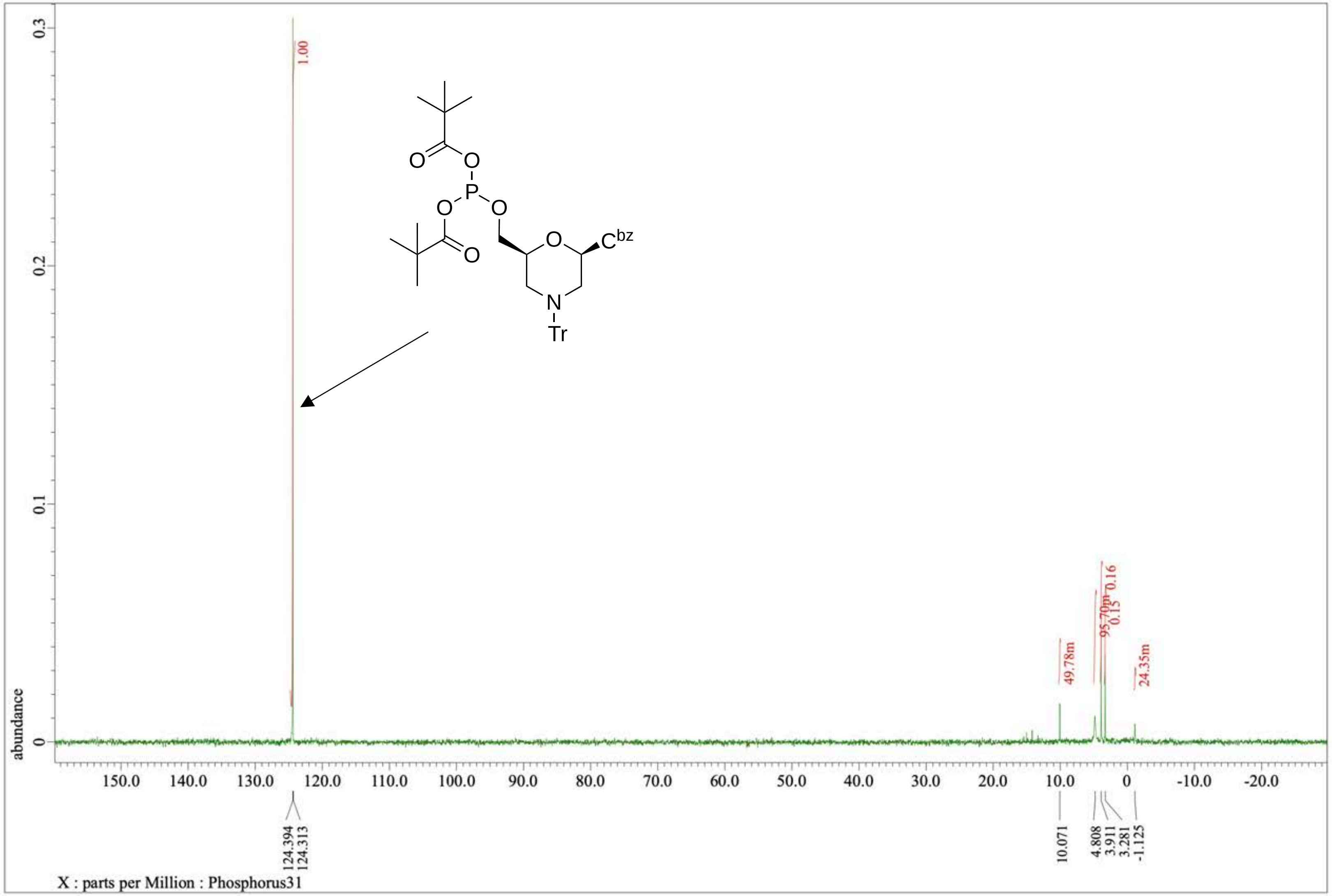

Fig. S 1 ^{31}P NMR spectrum (CD_3CN, 162 MHz) of the reaction mixture (condensing reagent: PivCl).

Table 1, entry 2 (BOPCl)

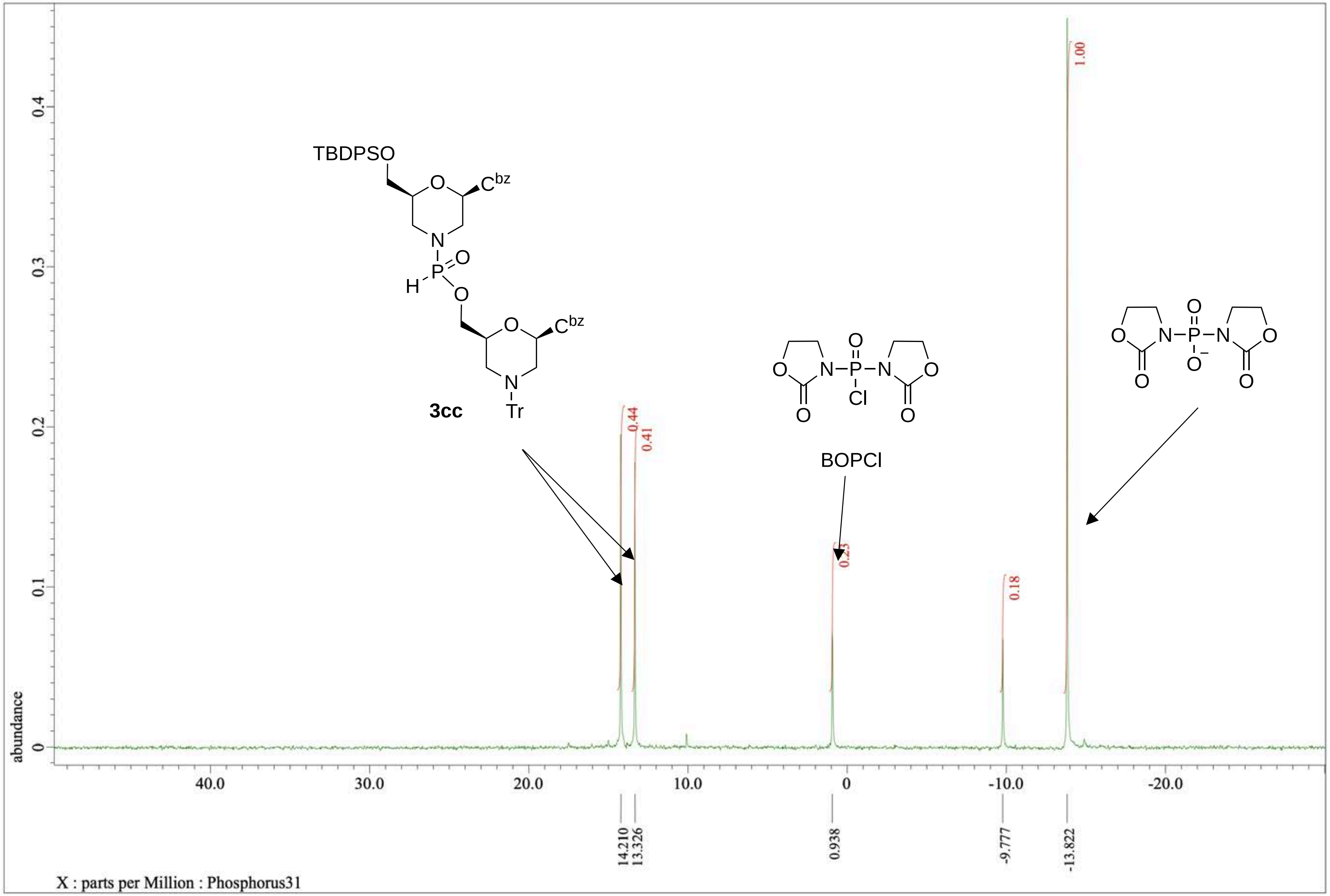

Fig. S 2 ^{31}P NMR spectrum (CD_3CN, 162 MHz) of the reaction mixture (condensing reagent: BOPCl).

Table 1, entry 3 (PyBOP)

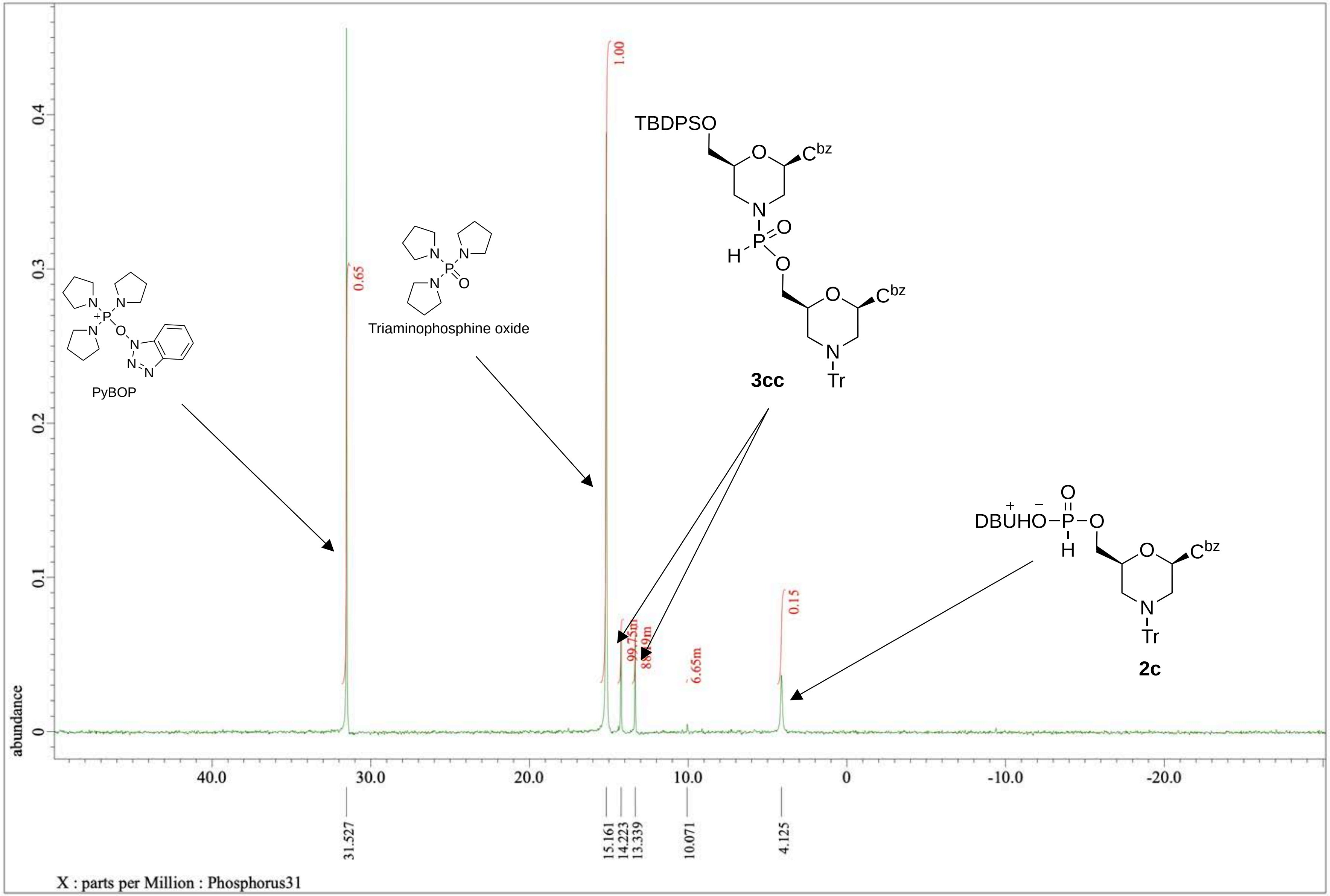

Fig. S 3 ^{31}P NMR spectrum (CD_3CN, 162 MHz) of the reaction mixture (condensing reagent: PyBOP).

Table 1, entry 4 (BOMP)

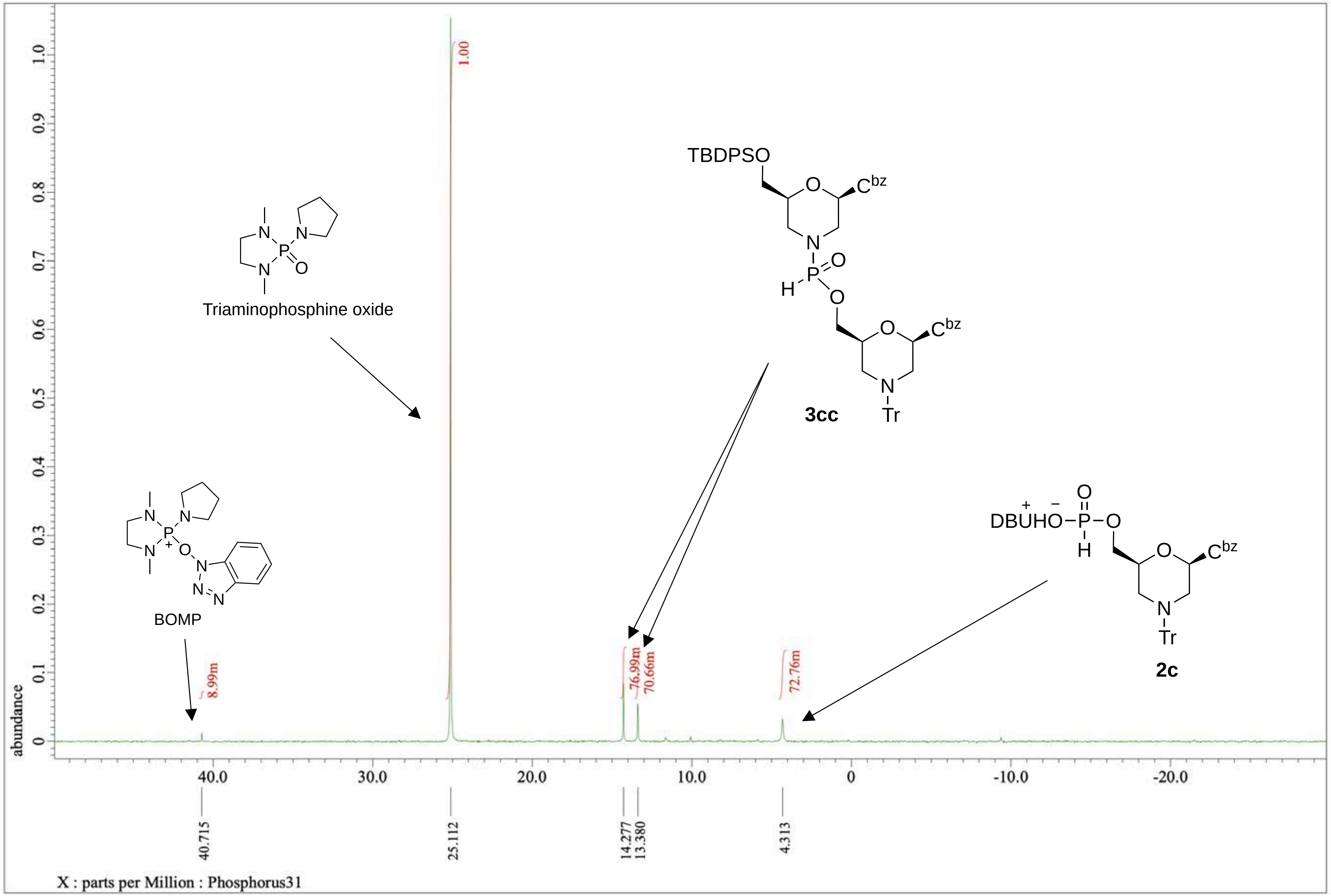

Fig. S 4 ^{31}P NMR spectrum (CD_3CN, 162 MHz) of the reaction mixture (condensing reagent: BOMP).

Table 1, entry 5 (PyNTP)

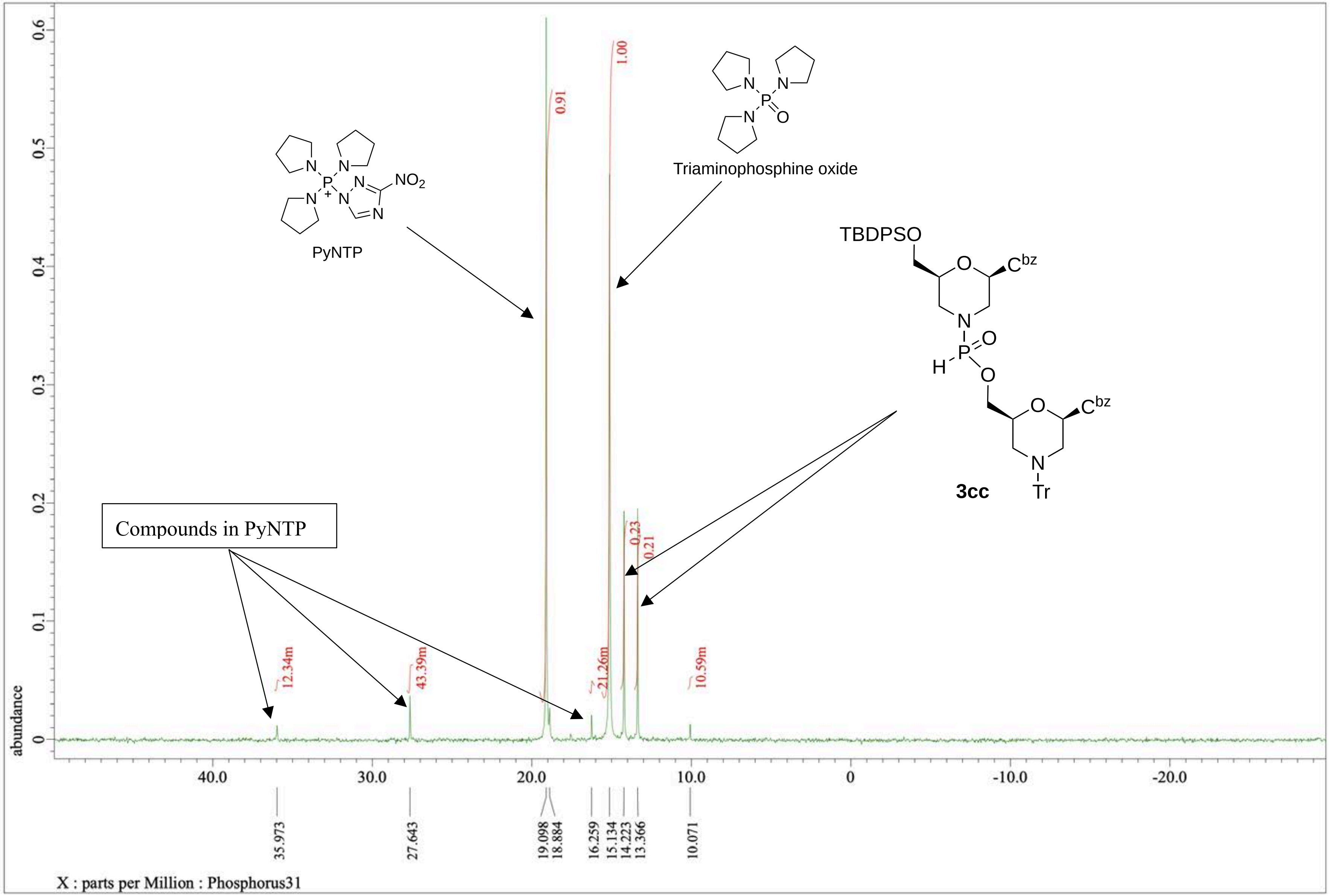

Fig. S 5 ^{31}P NMR spectrum (CD_3CN, 162 MHz) of the reaction mixture (condensing reagent: PyNTP).

Table 1, entry 6 (MNTP)

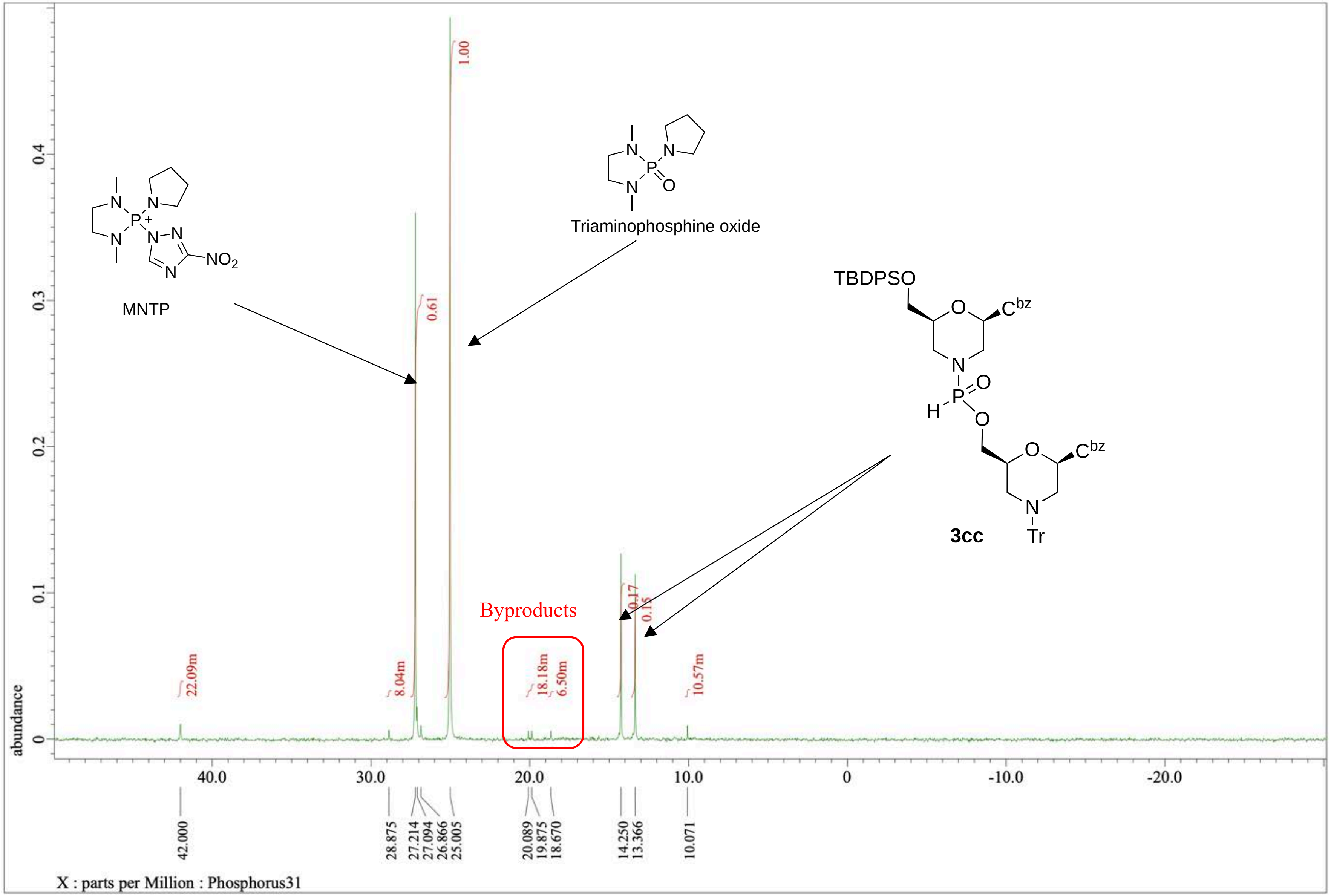

Fig. S 6 ^{31}P NMR spectrum (CD_3CN, 162 MHz) of the reaction mixture (condensing reagent: MNTP).

3. ^{31}P NMR analysis of the formation of phosphorodiamidate linkages (Table 2)

Table S1 Synthesis of phosphprodiamidate dimers 4 ($N_{PN}N$)

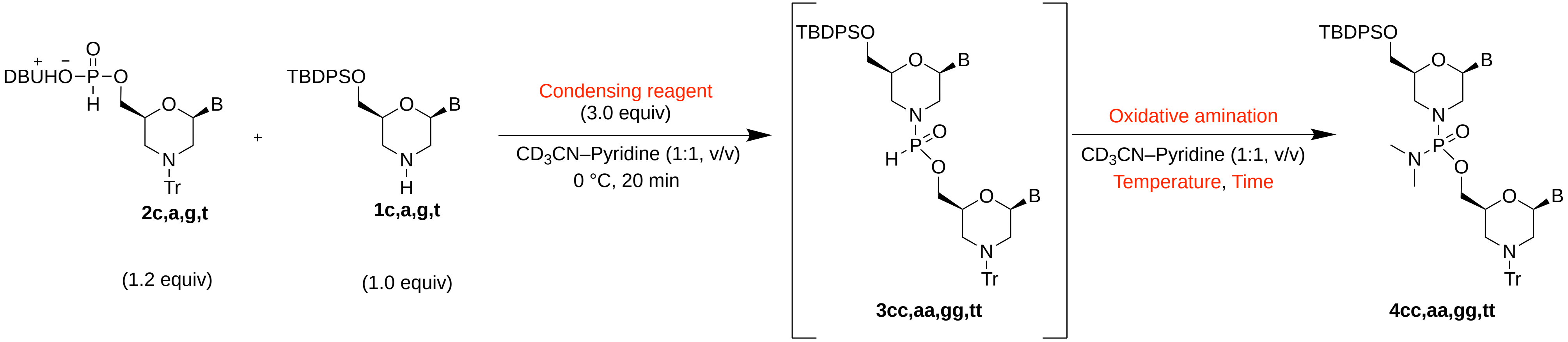

*The NMR yields were determined by the integral ratios of the **4cc** to the other byproducts, except for derivatives that were derived from condensing reagents.

Entry	Monomer **4**	Nucleoside **3**	Condensing reagent	Oxidative amination conditions	Temp. (°C)	Time (min)	Product	NMR yield (%)
1	**4c**	**3c**	BOPCl	I_2 (5.0 equiv)<br>Me_2NH in THF (2.2 equiv)<br>DIPEA (4.0 equiv)	25	30	$C_{PN}C$<br>(**4cc**)	0
2	**4c**	**3c**	BOPCl	CBr_4 (20 equiv)<br>Me_2NH in THF (2.2 equiv)<br>DIPEA (4.0 equiv)	25	30	$C_{PN}C$<br>(**4cc**)	34
3	**4c**	**3c**	BOPCl	CCl_4 (20 equiv)<br>Me_2NH in THF (2.2 equiv)<br>DIPEA (4.0 equiv)	25	30	$C_{PN}C$<br>(**4cc**)	48
4	**4c**	**3c**	BOPCl	CCl_4 (20 equiv)<br>Me_2NH in THF (9.6 equiv)	0	30	$C_{PN}C$<br>(**4cc**)	47
5	**4c**	**3c**	PyNTP	CCl_4 (20 equiv)<br>Me_2NH in THF (9.6 equiv)	0	30	$C_{PN}C$<br>(**4cc**)	91
6	**4c**	**3c**	PyNTP	CCl_4 (20 equiv)<br>Me_2NH in H_2O (9.6 equiv)	0	30	$C_{PN}C$<br>(**4cc**)	98
7	**4c**	**3c**	PyNTP	CCl_4 (20 equiv)<br>Me_2NH in H_2O (38 equiv)	0	30	$C_{PN}C$<br>(**4cc**)	98
8	**4c**	**3c**	PyNTP	CCl_4 (20 equiv)<br>Me_2NH in H_2O (38 equiv)	0	1	$C_{PN}C$<br>(**4cc**)	>99
9	**4a**	**3a**	PyNTP	CCl_4 (20 equiv)<br>Me_2NH in H_2O (38 equiv)	0	1	$A_{PN}A$<br>(**4aa**)	>99
10	**4g**	**3g**	PyNTP	CCl_4 (20 equiv)<br>Me_2NH in H_2O (38 equiv)	0	1	$G_{PN}G$<br>(**4gg**)	>99
11	**4t**	**3t**	PyNTP	CCl_4 (20 equiv)<br>Me_2NH in H_2O (38 equiv)	0	1	$T_{PN}T$<br>(**4tt**)	>99

Table S1, entry 1

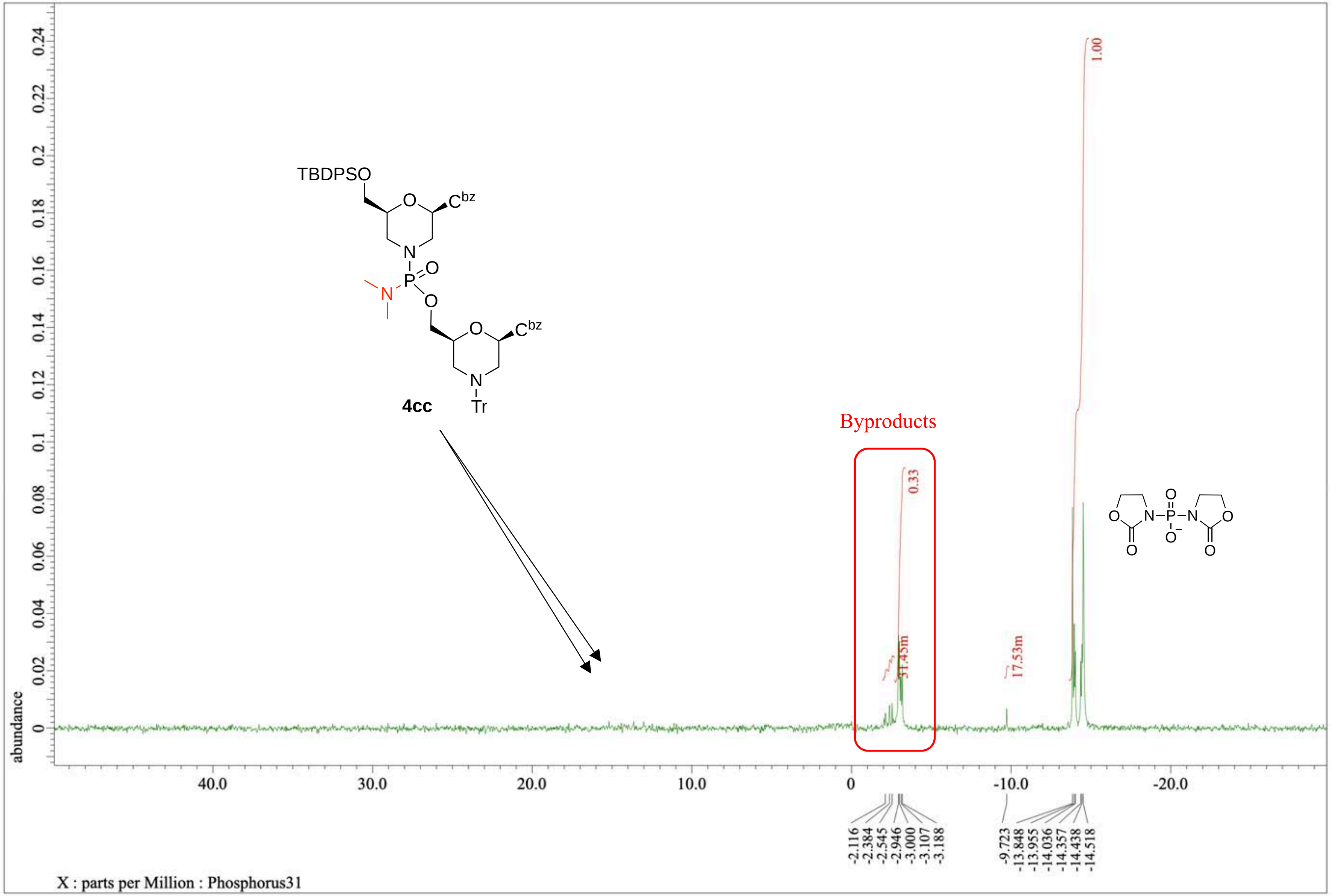

Fig. S 7 ^{31}P NMR spectrum (CD_3CN, 162 MHz) of the reaction mixture (halogenating reagent: I_2).

Table S1, entry 2

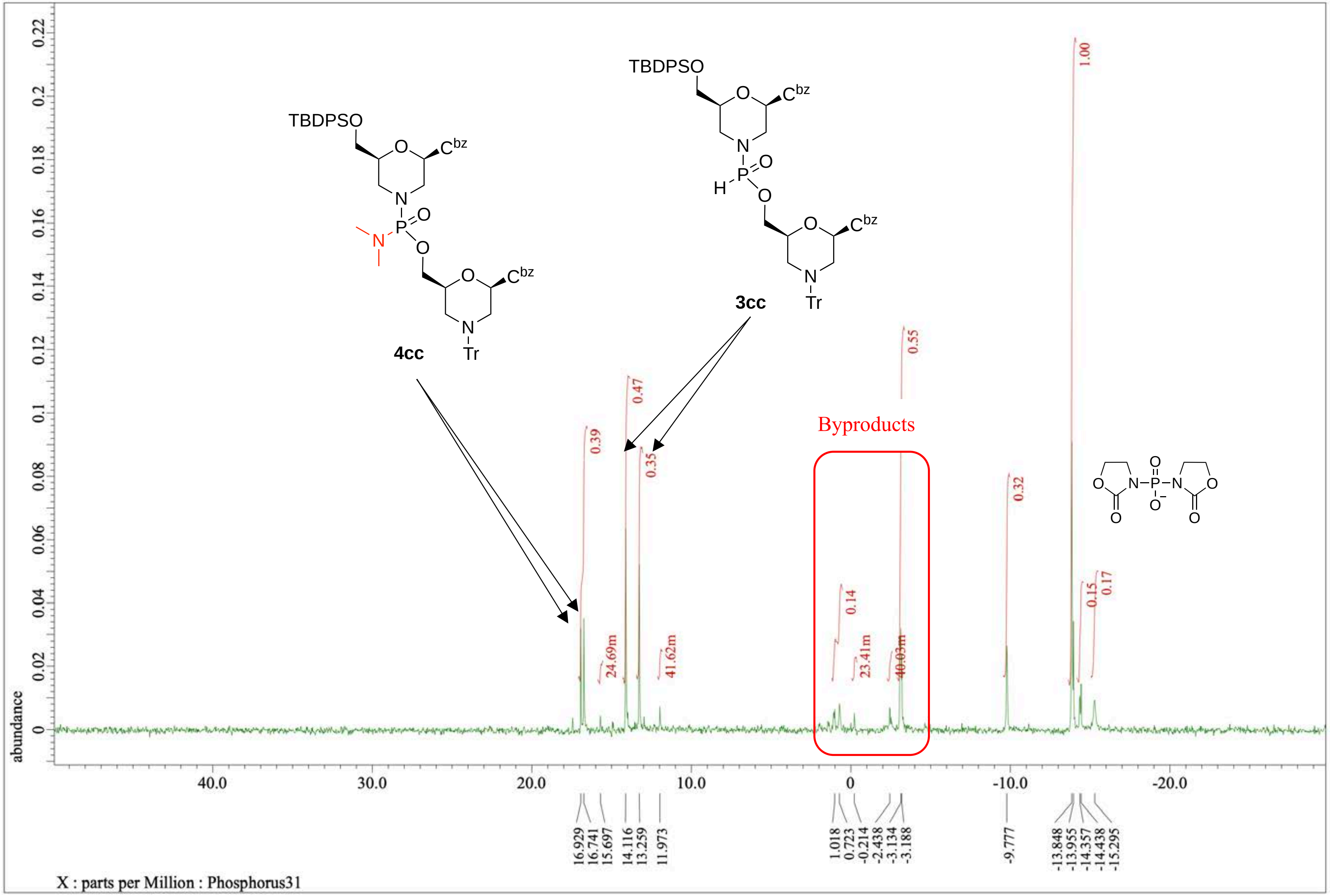

Fig. S 8 ^{31}P NMR spectrum (CD_3CN, 162 MHz) of the reaction mixture (halogenating reagent: CBr_4).

Table S1, entry 3

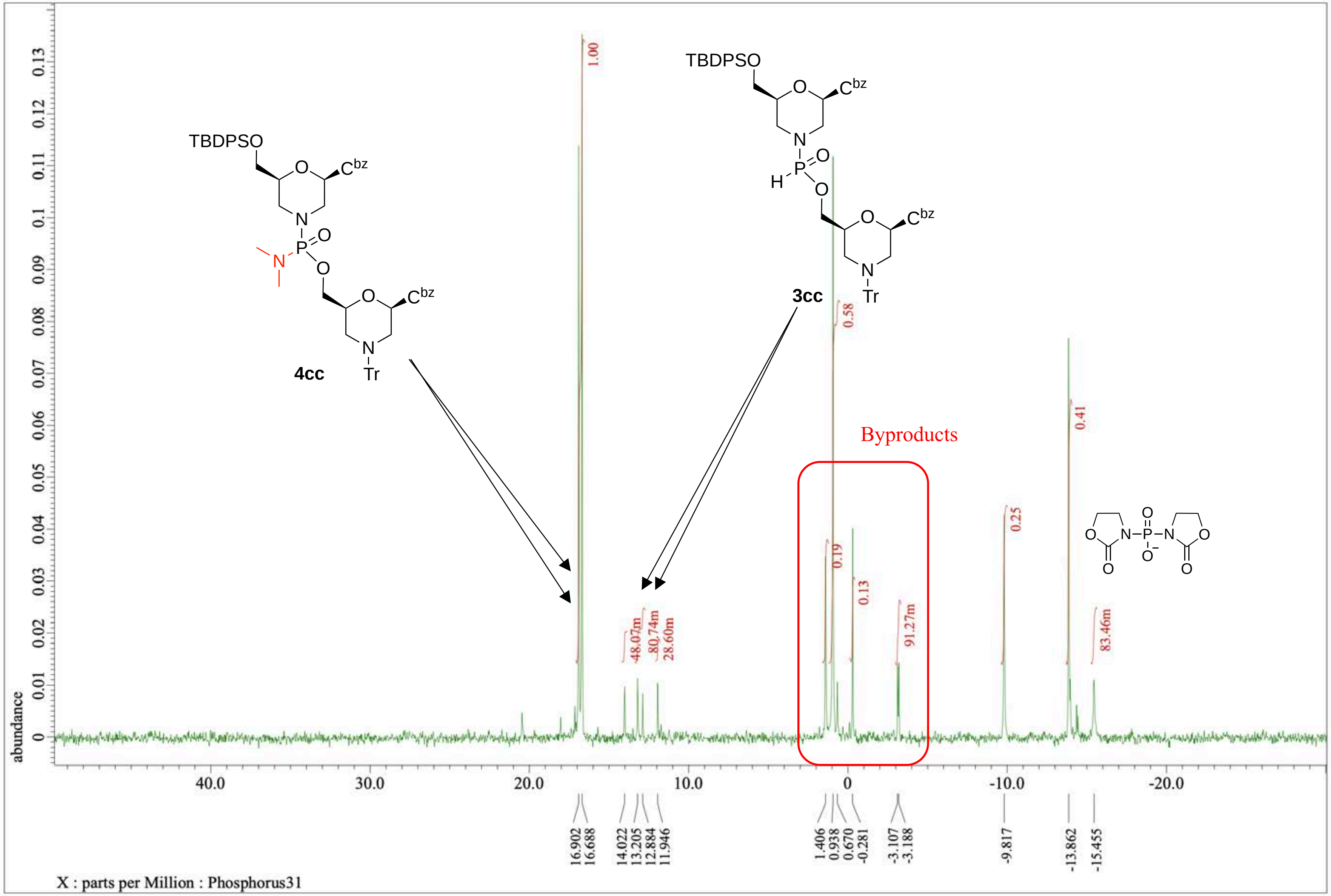

Fig. S 9 ^{31}P NMR spectrum (CD_3CN, 162 MHz) of the reaction mixture (halogenating reagent: CCl_4).

Table S1, entry 4

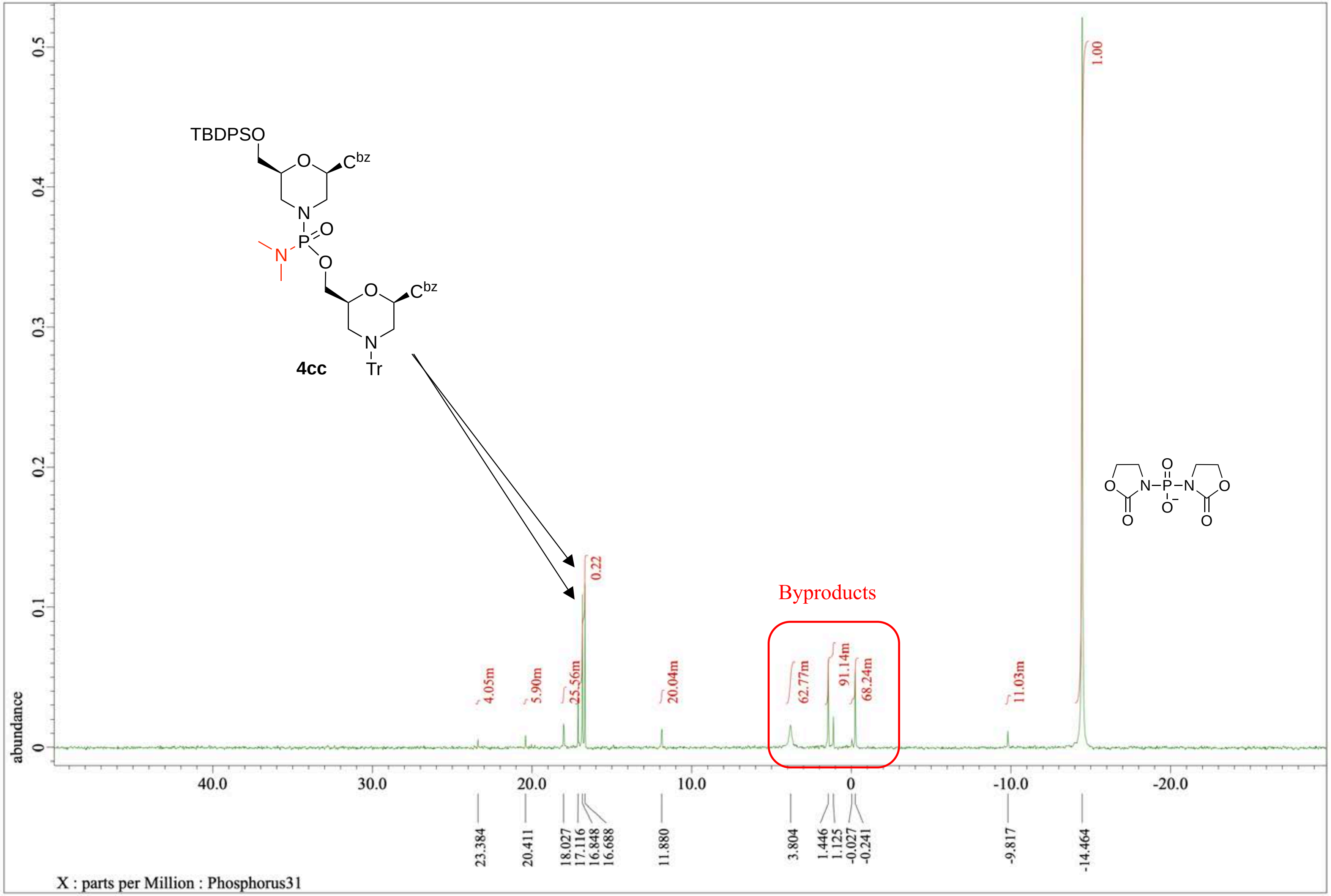

Fig. S 10 ^{31}P NMR spectrum (CD_3CN, 162 MHz) of the reaction mixture (equivalent of Me_2NH: 9.6 equiv in THF).

Table S1, entry 5

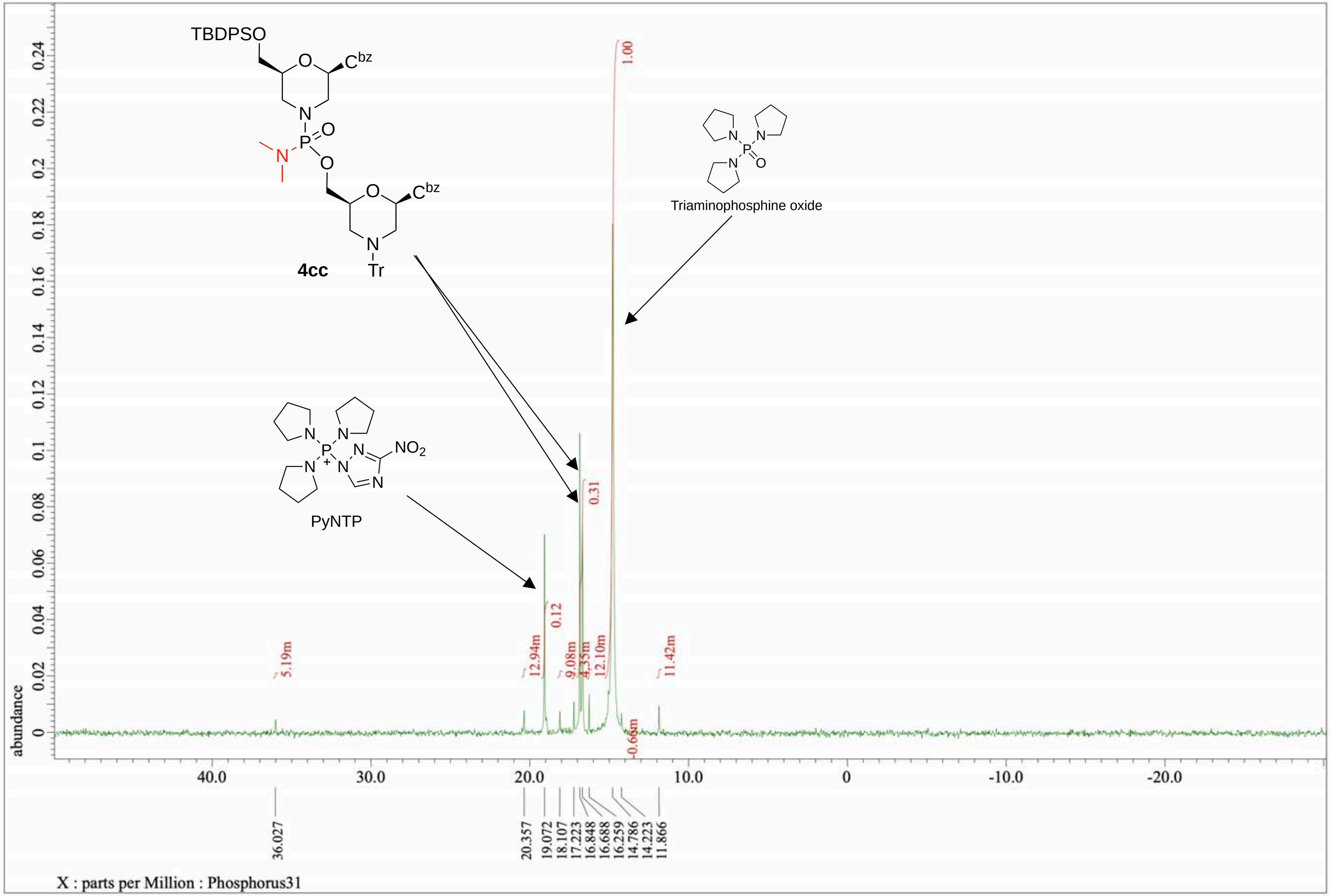

Fig. S 11 ^{31}P NMR spectrum (CD_3CN, 162 MHz) of the reaction mixture (condensing reagent: PyNTP).

Table S1, entry 6

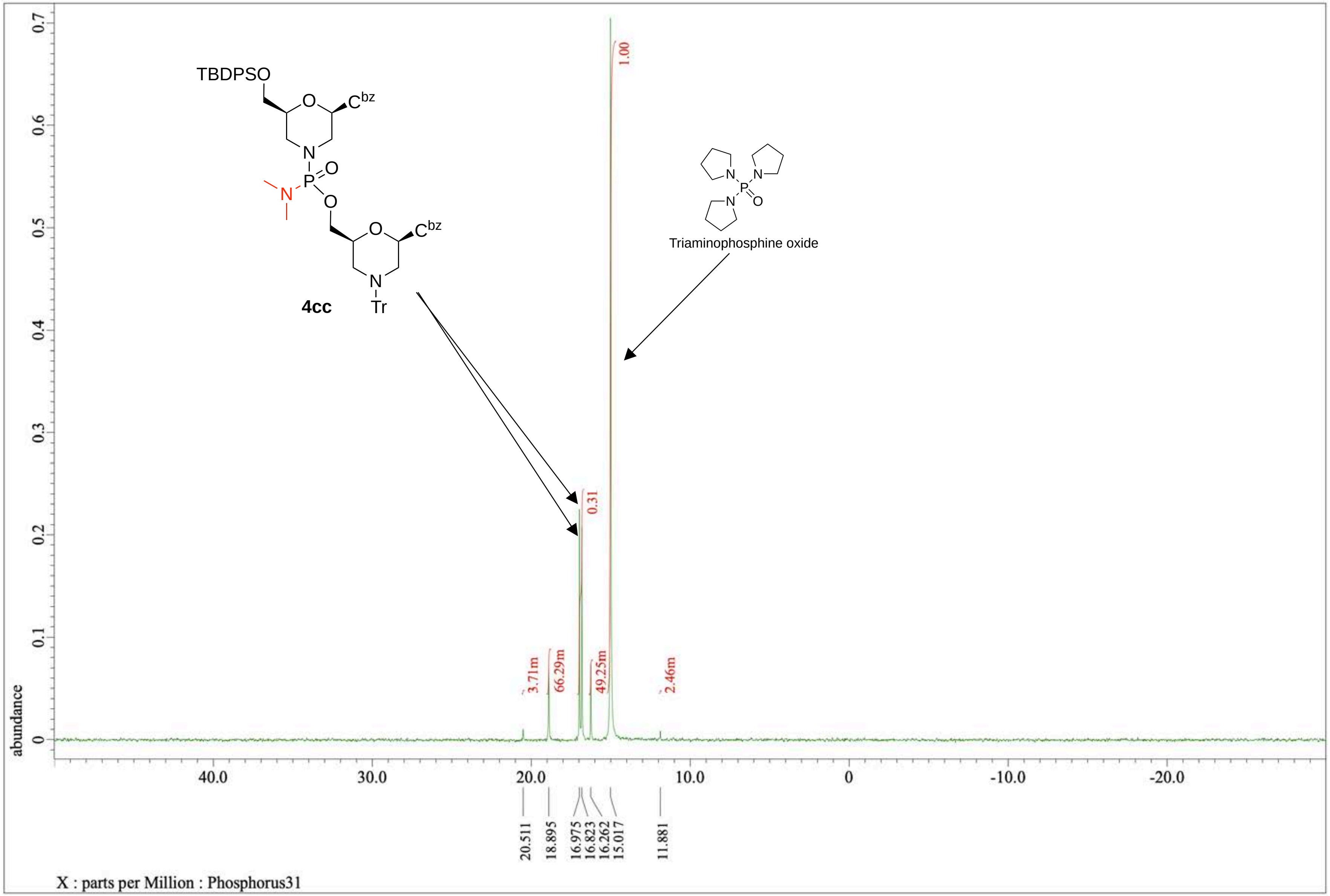

Fig. S 12 ^{31}P NMR spectrum (CD_3CN, 162 MHz) of the reaction mixture (reaction conditions: Me_2NH: 9.6 equiv in H_2O).

Table S1, entry 7

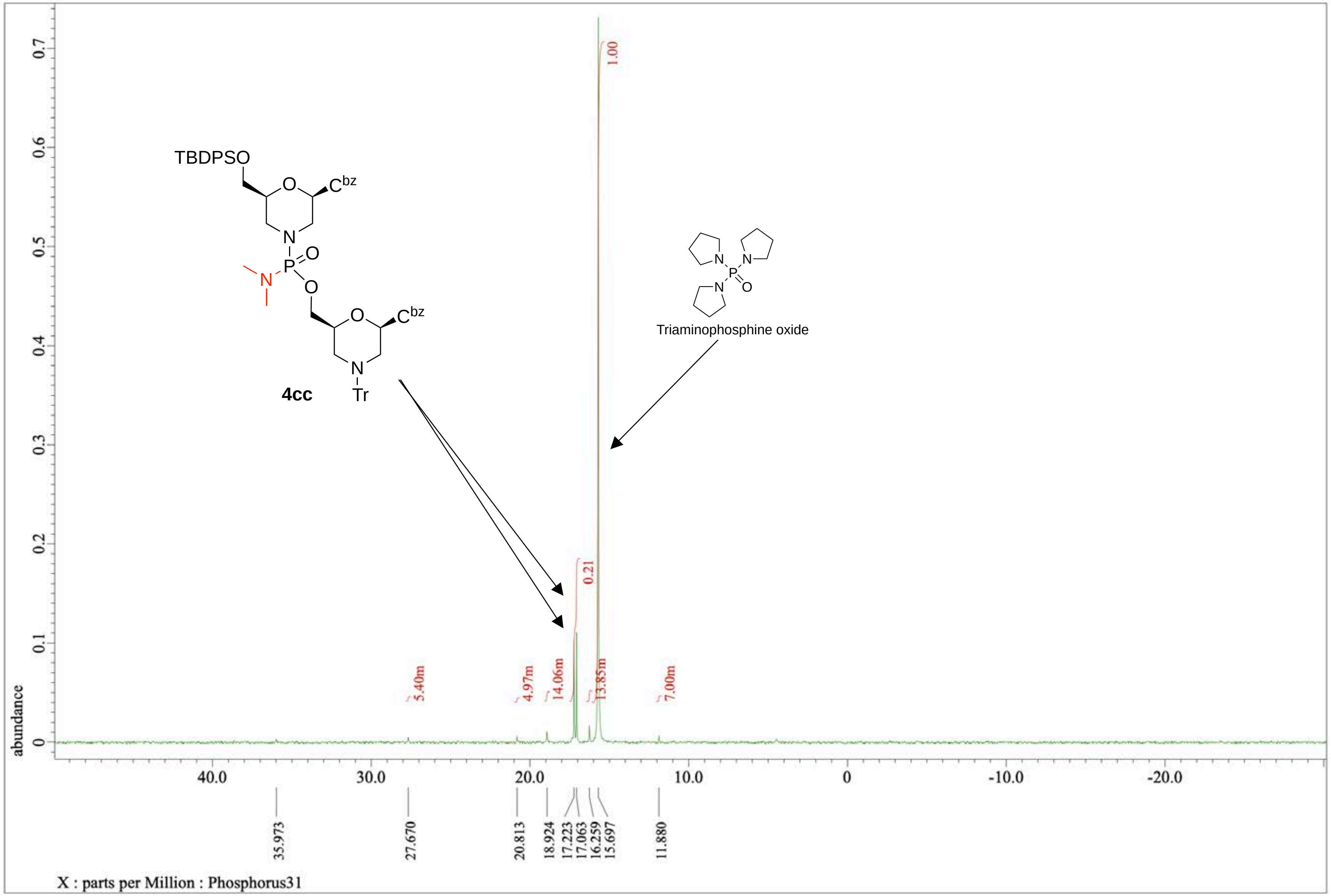

Fig. S 13 ^{31}P NMR spectrum (CD_3CN, 162 MHz) of the reaction mixture (reaction conditions: Me_2NH: 38 equiv in H_2O).

Table S1, entry 8

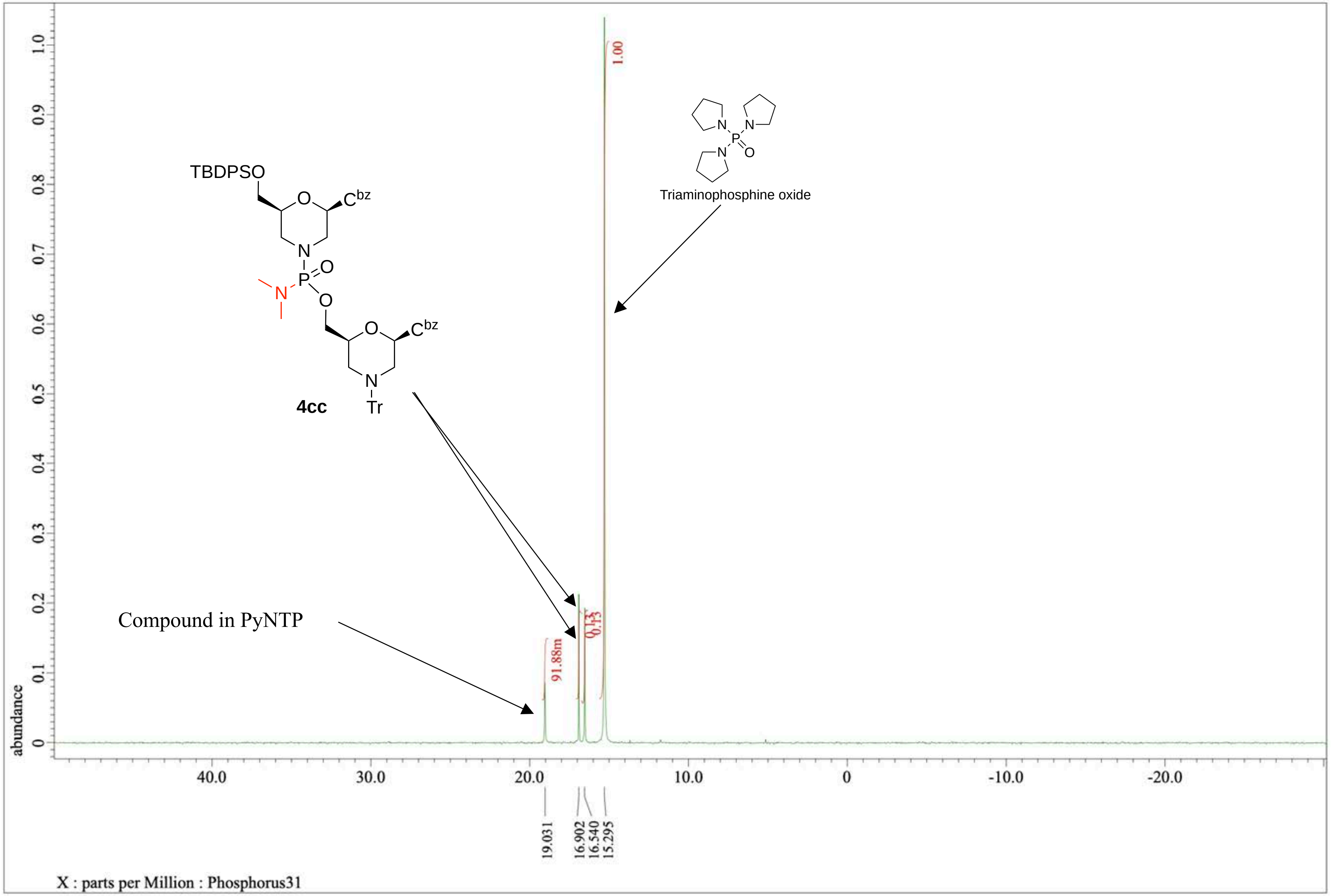

Fig. S 14 ^{31}P NMR spectrum (CD_3CN, 162 MHz) of the reaction mixture (reaction time: 1 min, $C_{PN}C$ (**4cc**)).

Table S1, entry 8

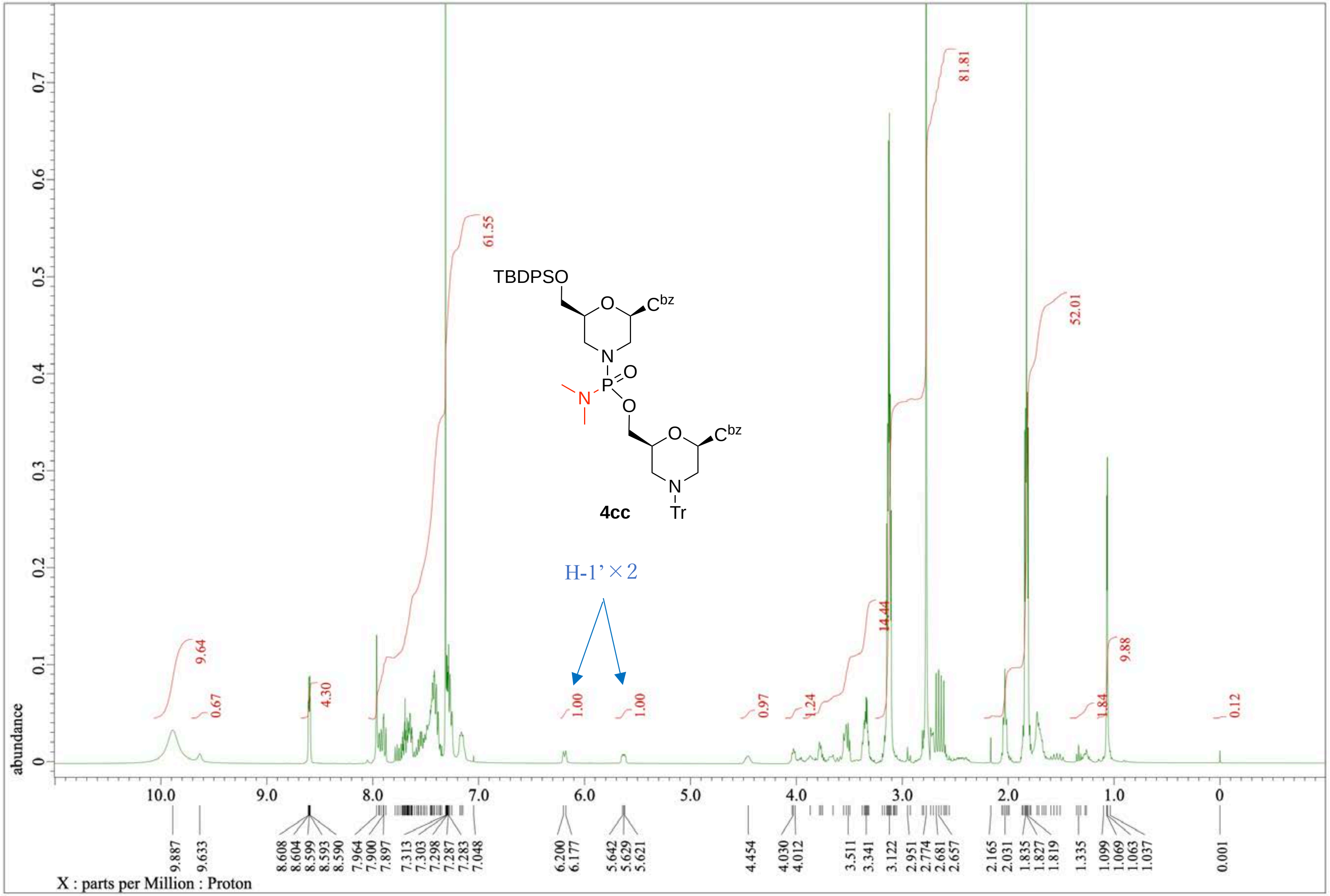

Fig. S 15 ^{1}H NMR spectrum (CD_3CN, 400 MHz) of the reaction mixture (reaction time: 1 min, $C_{PN}C$ (**4cc**)).

Table S1, entry 9

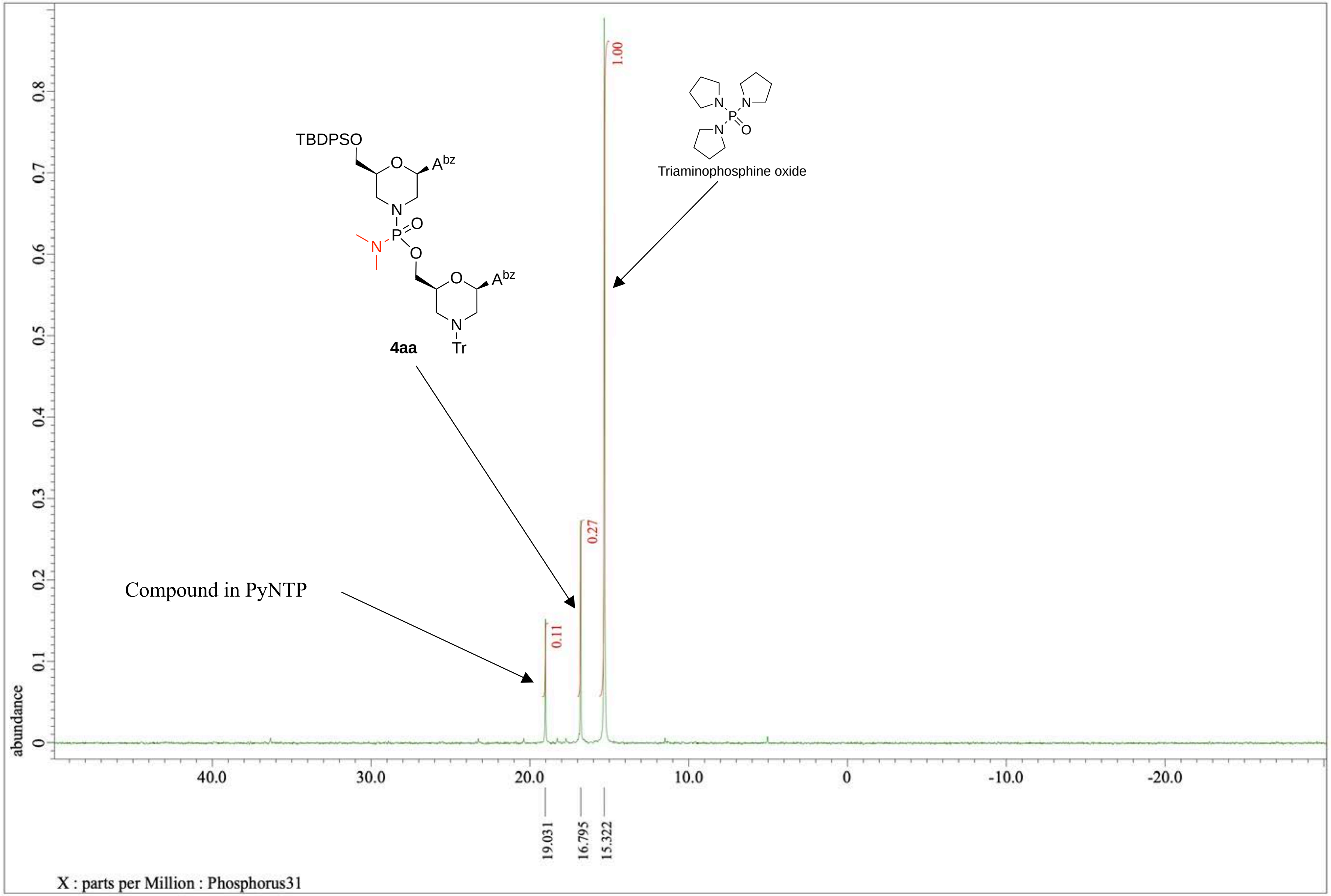

Fig. S 16 ^{31}P NMR spectrum (CD_3CN, 162 MHz) of the reaction mixture (reaction time: 1 min, A$_{PN}$A (**4aa**)).

Table S1, entry 9

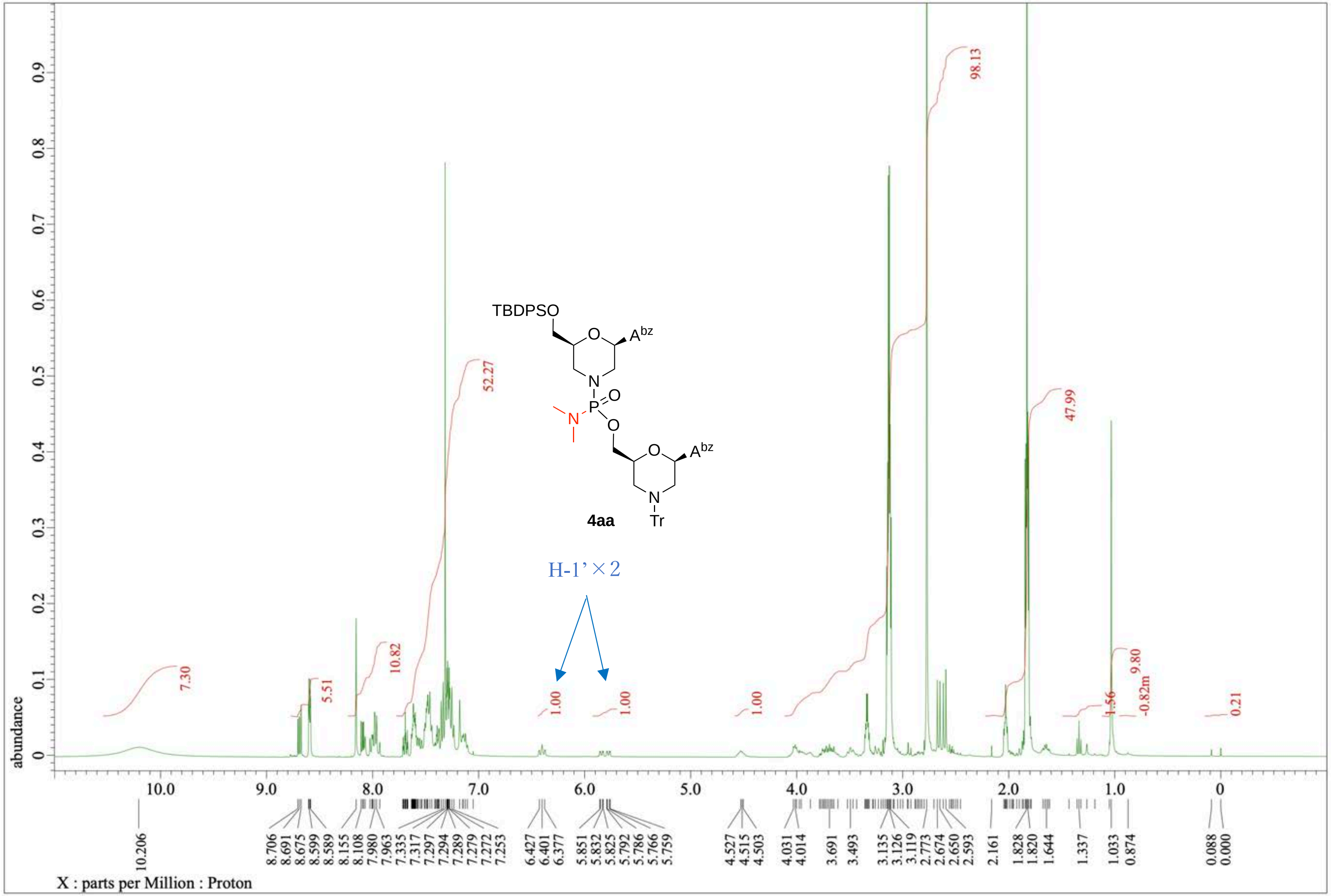

Fig. S 17 ^{1}H NMR spectrum (CD_3CN, 400 MHz) of the reaction mixture (reaction time: 1 min, A$_{PN}$A (**4aa**)).

Table S1, entry 10

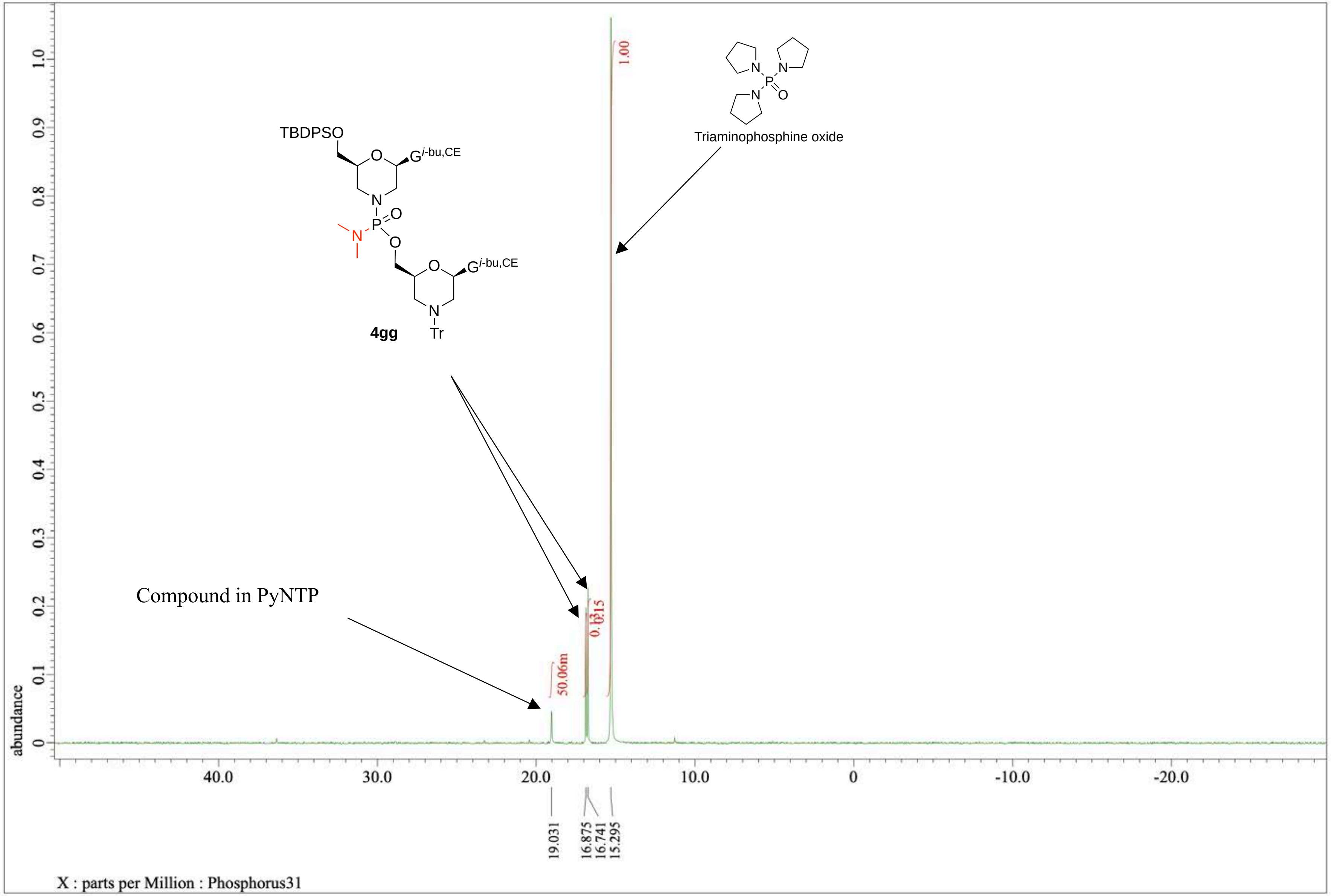

Fig. S 18 ^{31}P NMR spectrum (CD_3CN, 162 MHz) of the reaction mixture (reaction time: 1 min, $G_{PN}G$ (**4gg**)).

Table S1, entry 10

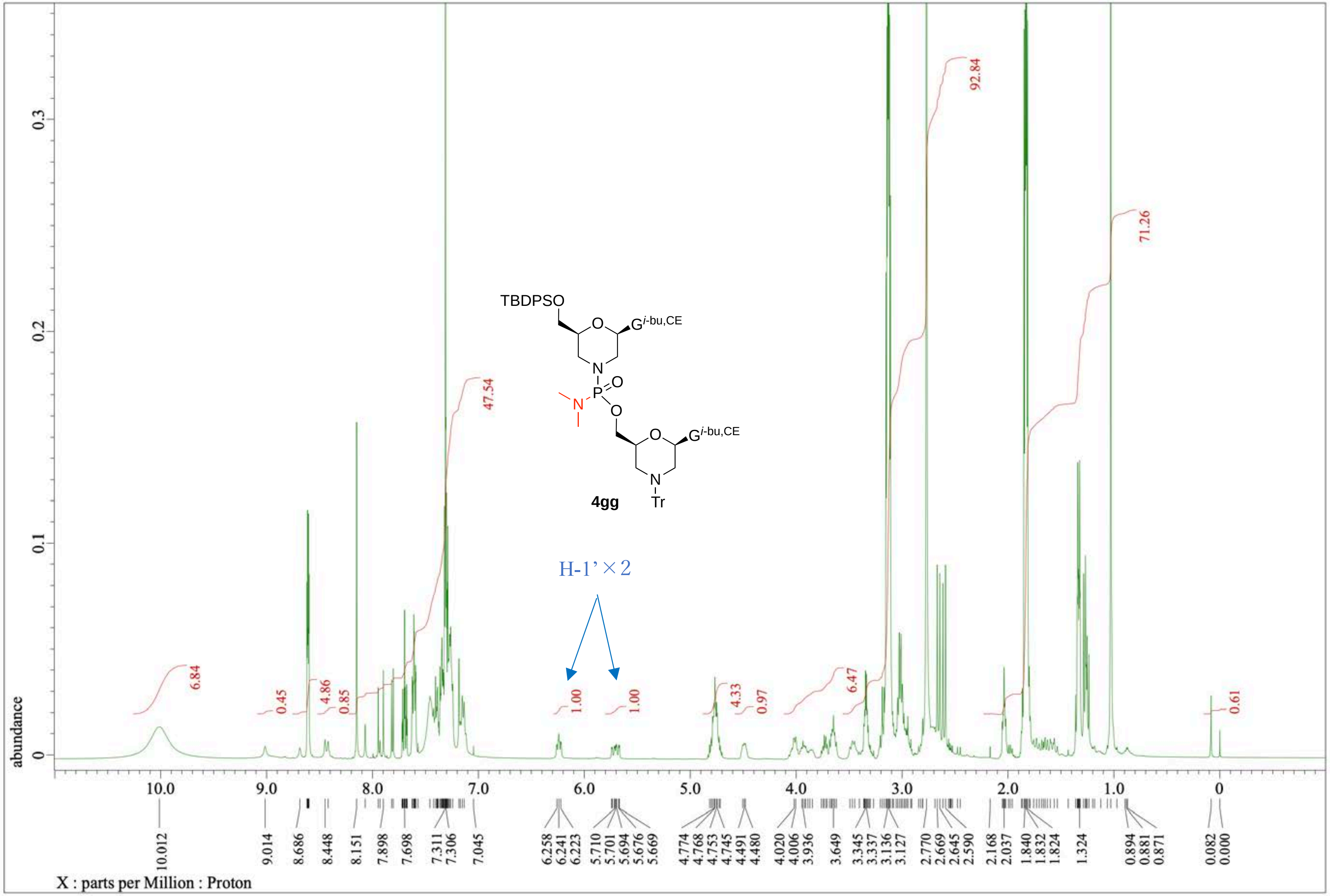

Fig. S 19 ^{1}H NMR spectrum (CD_3CN, 400 MHz) of the reaction mixture (reaction time: 1 min, G$_{PN}$G (**4gg**)).

Table S1, entry 11

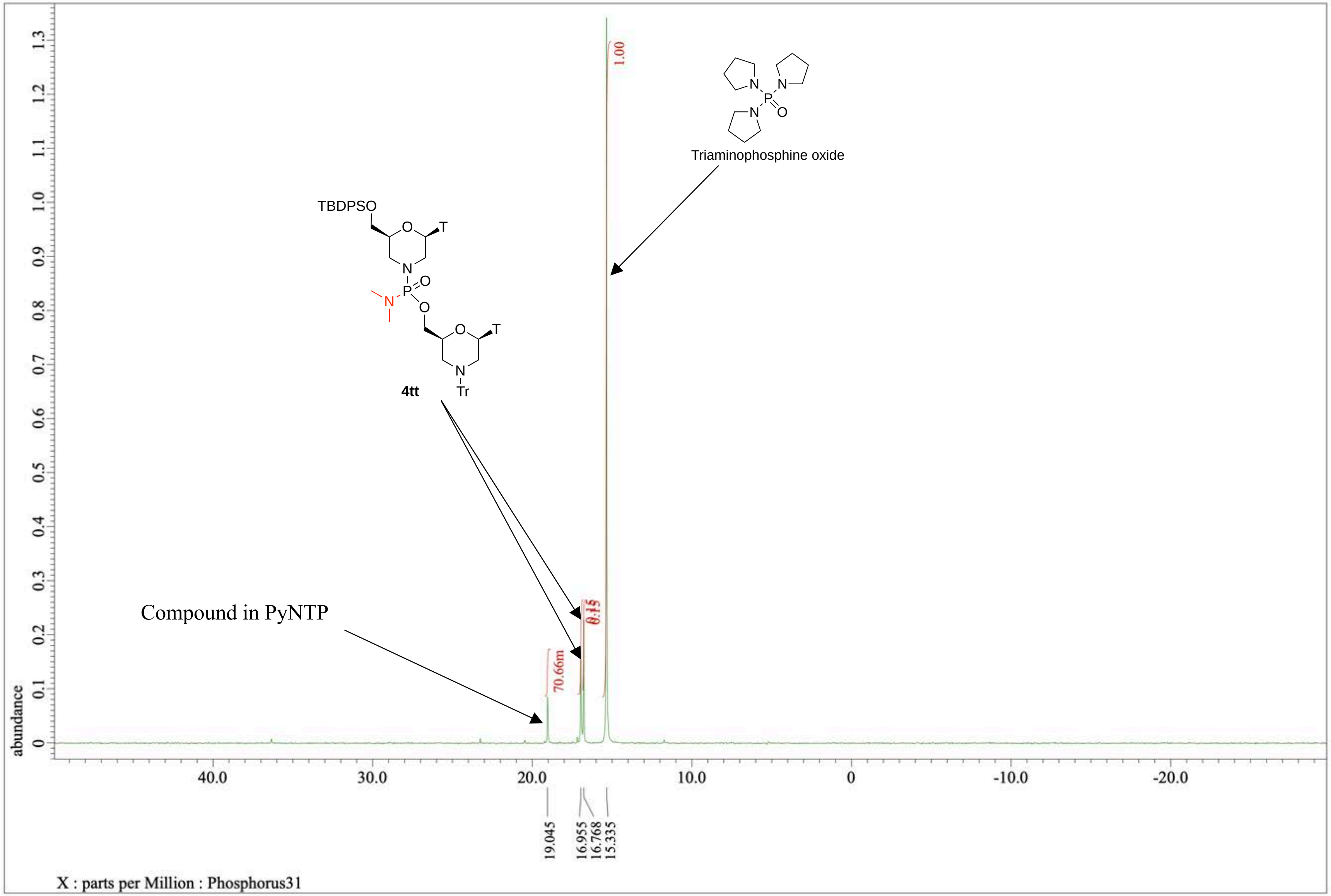

Fig. S 20 ^{31}P NMR spectrum (CD_3CN, 162 MHz) of the reaction mixture (reaction time: 1 min, $T_{PN}T$ (**4tt**)).

Table S1, entry 11

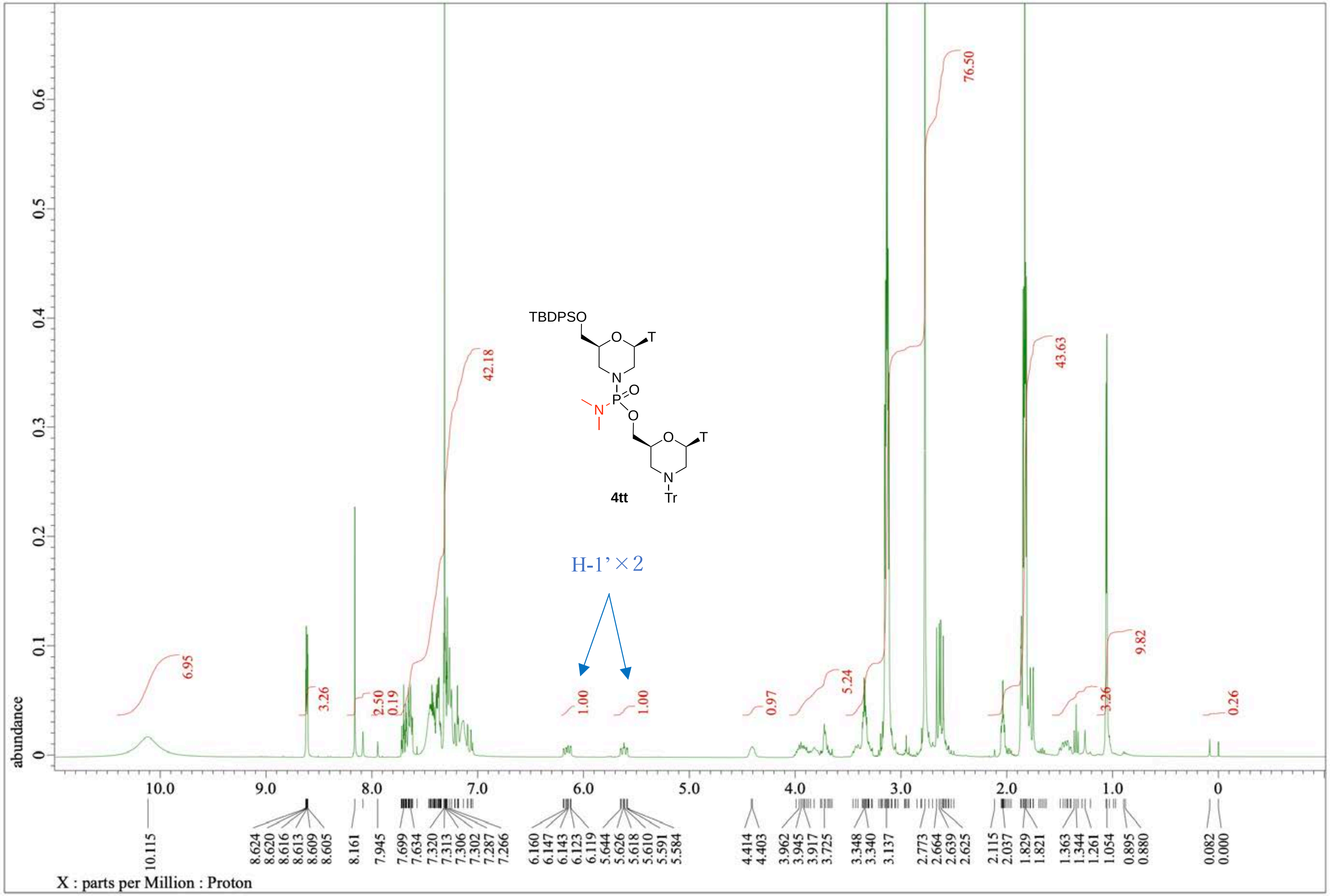

Fig. S 21 ^{1}H NMR spectrum (CD_3CN, 400 MHz) of the reaction mixture (reaction time: 1 min, $T_{PN}T$ (**4tt**)).

Scheme S 9. Plausible mechanism of a side reaction when using BOPCl as a condensing reagent

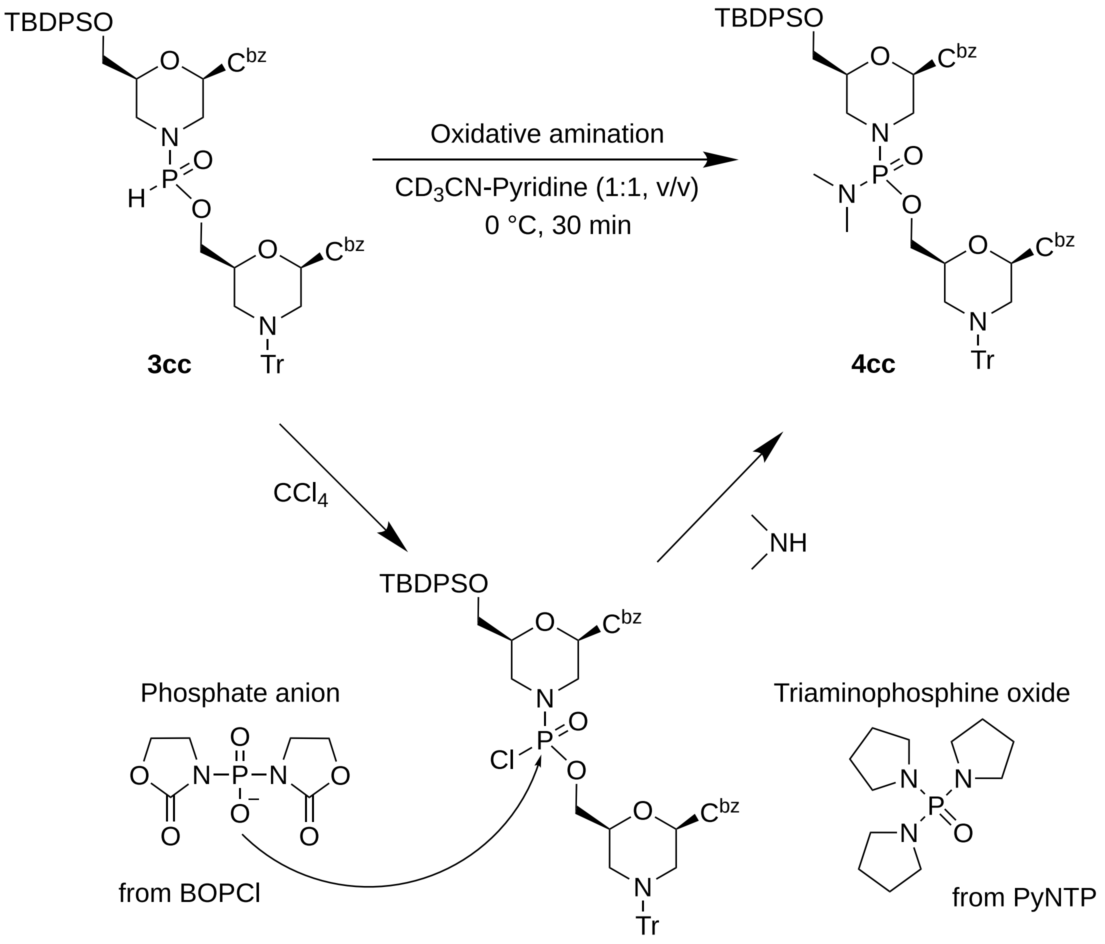

From the results of Table S1, it was revealed that compared with BOPCl (47%, entry 4), PyNTP gave the better result (91%, entry 5) for the condensation and oxidative amination reaction as a one-pot reaction. We reasoned that residues of condensing reagent affected the oxidative amination reaction. A residue of BOPCl was a phosphate anion and reacted with a chlorophosphoramidate intermediate. On the other hand, a residue of PyNTP was a nonactive triaminophosphine oxide derivative and would not inhibit the oxidative amination reaction.

4. HPLC analysis of the fragment condensation reactions.

Scheme 3 (2-mer + 2-mer, condensing reagent: BOMP)

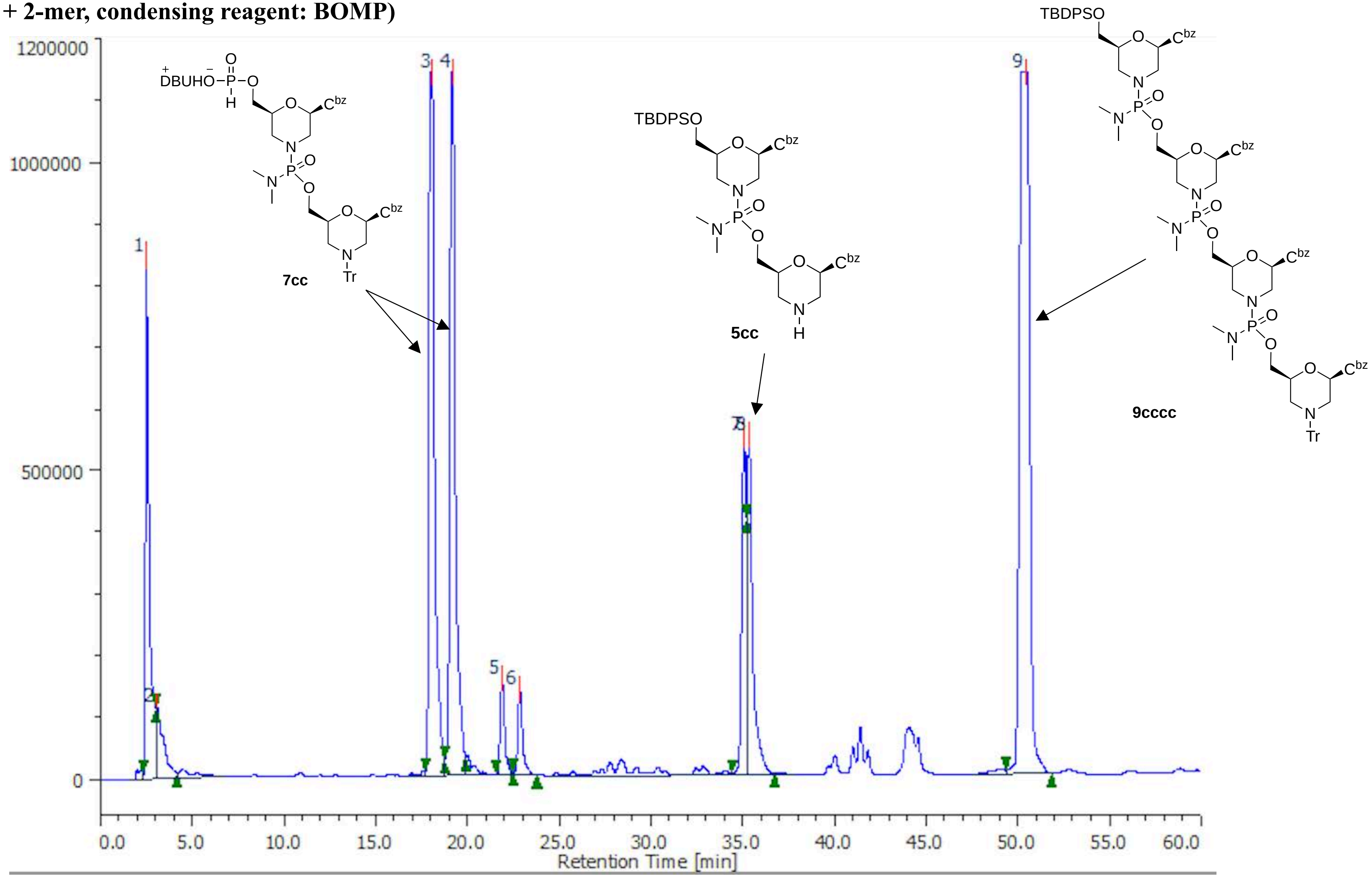

Fig. S 22 HPLC profile of the crude 4-mer. RP-HPLC was performed with a linear gradient of 40%–100% CH_3CN in 0.1 M TEAA buffer (pH 7.0) over 60 min at 50 °C at a rate of 0.5 mL/min.

Scheme 3 (2-mer + 2-mer, condensing reagent: PyNTP)

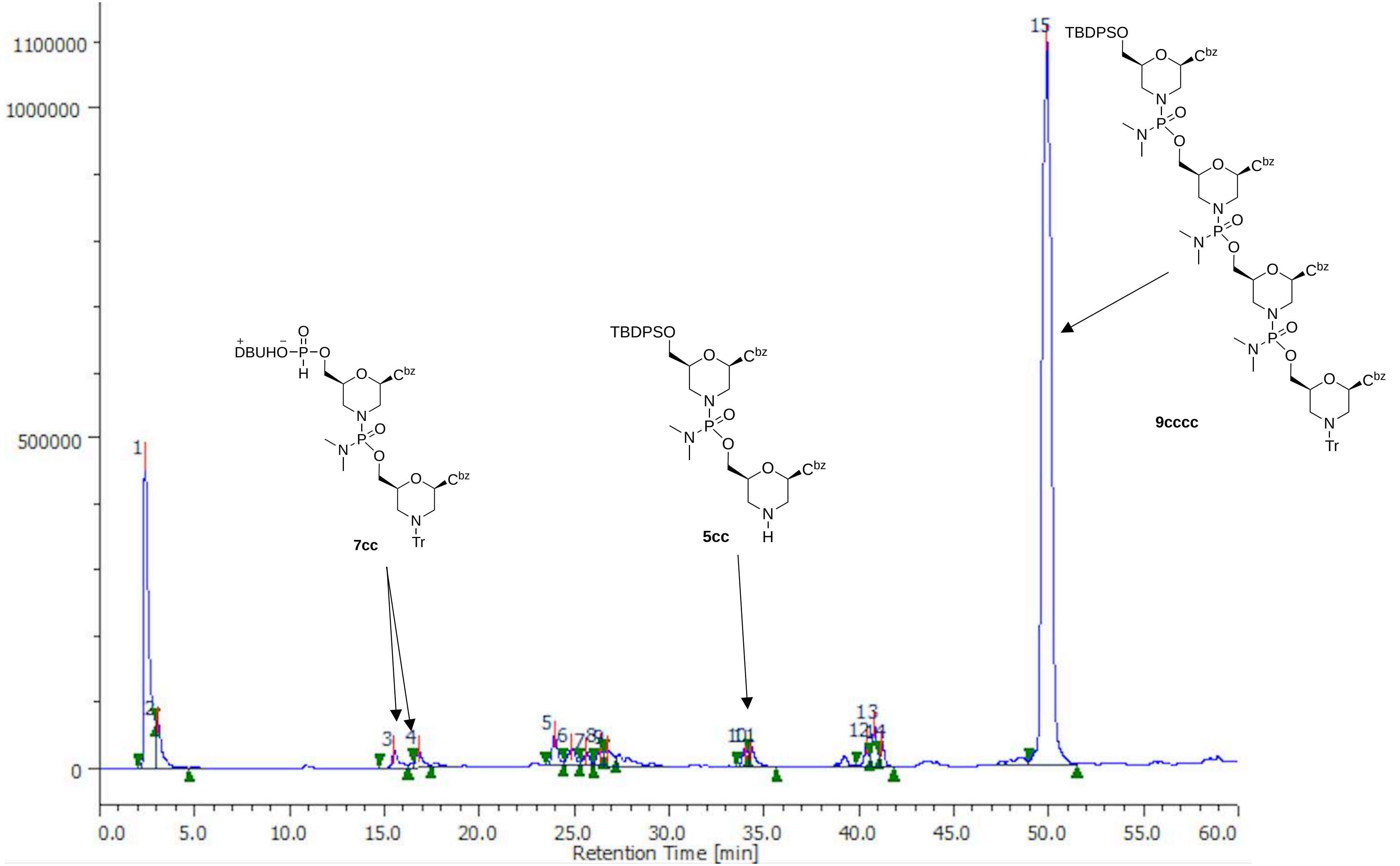

Fig. S 23 HPLC profile of the crude 4-mer. RP-HPLC was performed with a linear gradient of 40%–100% CH_3CN in 0.1 M TEAA buffer (pH 7.0) over 60 min at 50 °C at a rate of 0.5 mL/min.

Table 4, entry 1 (4-mer + 2-mer, condensing reagent: PyNTP)

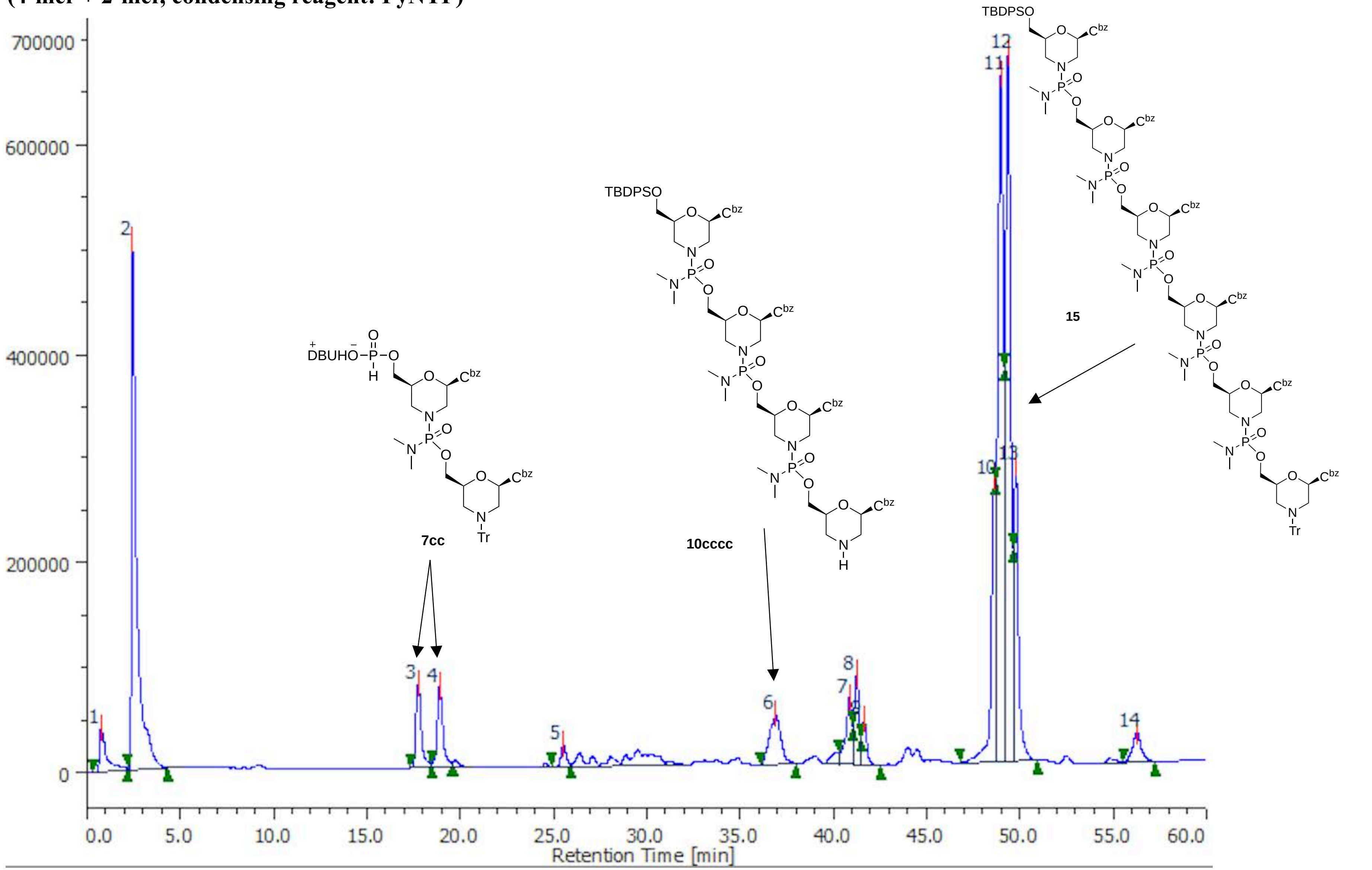

Fig. S 24 HPLC profile of the crude 6-mer. RP-HPLC was performed with a linear gradient of 40%–100% CH_3CN in 0.1 M TEAA buffer (pH 7.0) over 60 min at 50 °C at a rate of 0.5 mL/min.

Table 4, entry 2 (2-mer + 4-mer, condensing reagent: PyNTP)

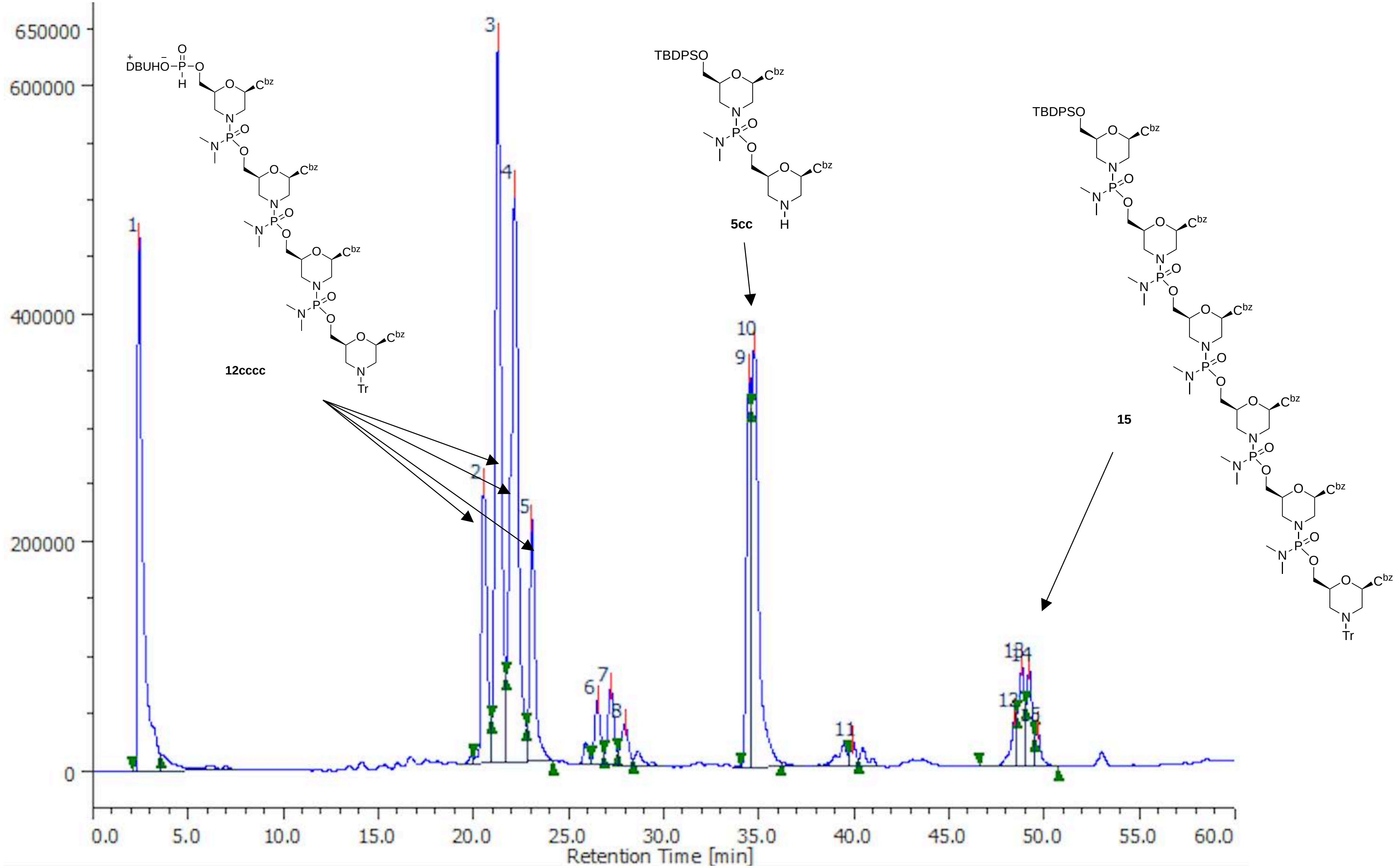

Fig. S 25 HPLC profile of the crude 6-mer. RP-HPLC was performed with a linear gradient of 40%–100% CH_3CN in 0.1 M TEAA buffer (pH 7.0) over 60 min at 50 °C at a rate of 0.5 mL/min.

Table 4, entry 3 (2-mer + 4-mer, condensing reagent: MNTP)

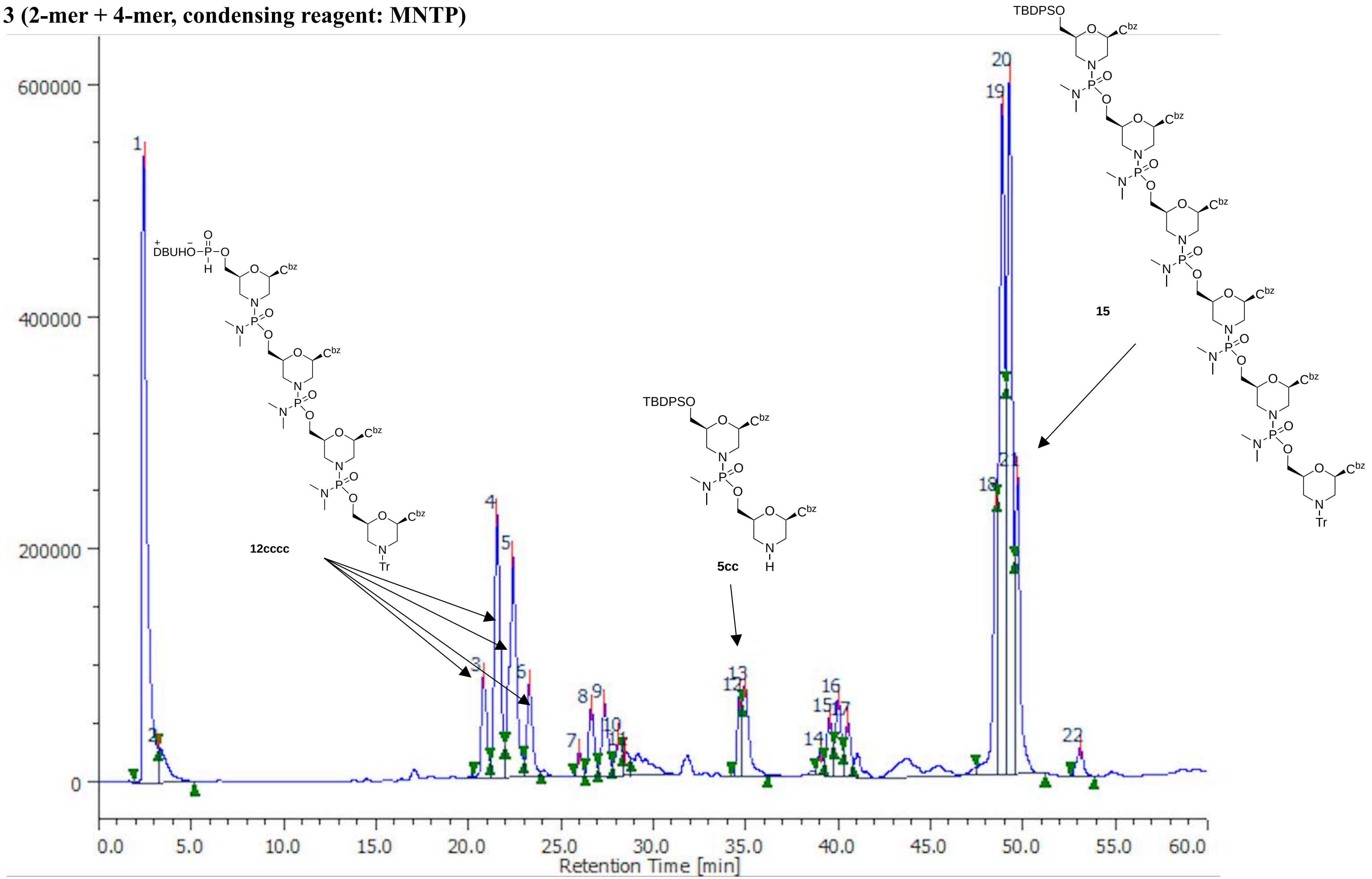

Fig. S 26 HPLC profile of the crude 6-mer. RP-HPLC was performed with a linear gradient of 40%–100% CH_3CN in 0.1 M TEAA buffer (pH 7.0) over 60 min at 50 °C at a rate of 0.5 mL/min.

Table 4, entry 4 (4-mer + 4-mer, condensing reagent: PyNTP)

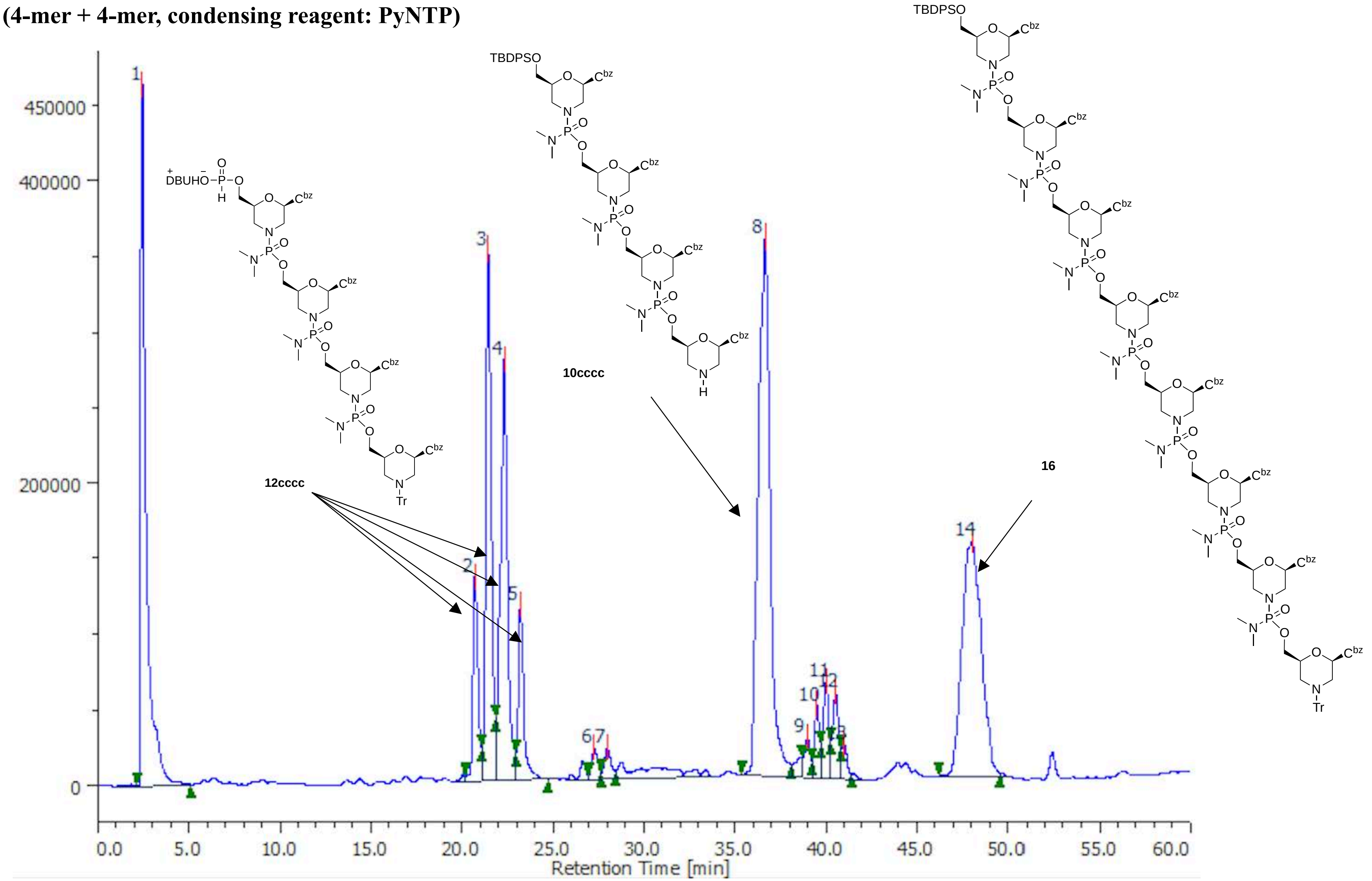

Fig. S 27 HPLC profile of the crude 8-mer. RP-HPLC was performed with a linear gradient of 40%–100% CH_3CN in 0.1 M TEAA buffer (pH 7.0) over 60 min at 50 °C at a rate of 0.5 mL/min.

Table 4, entry 5 (4-mer + 4-mer, condensing reagent: MNTP)

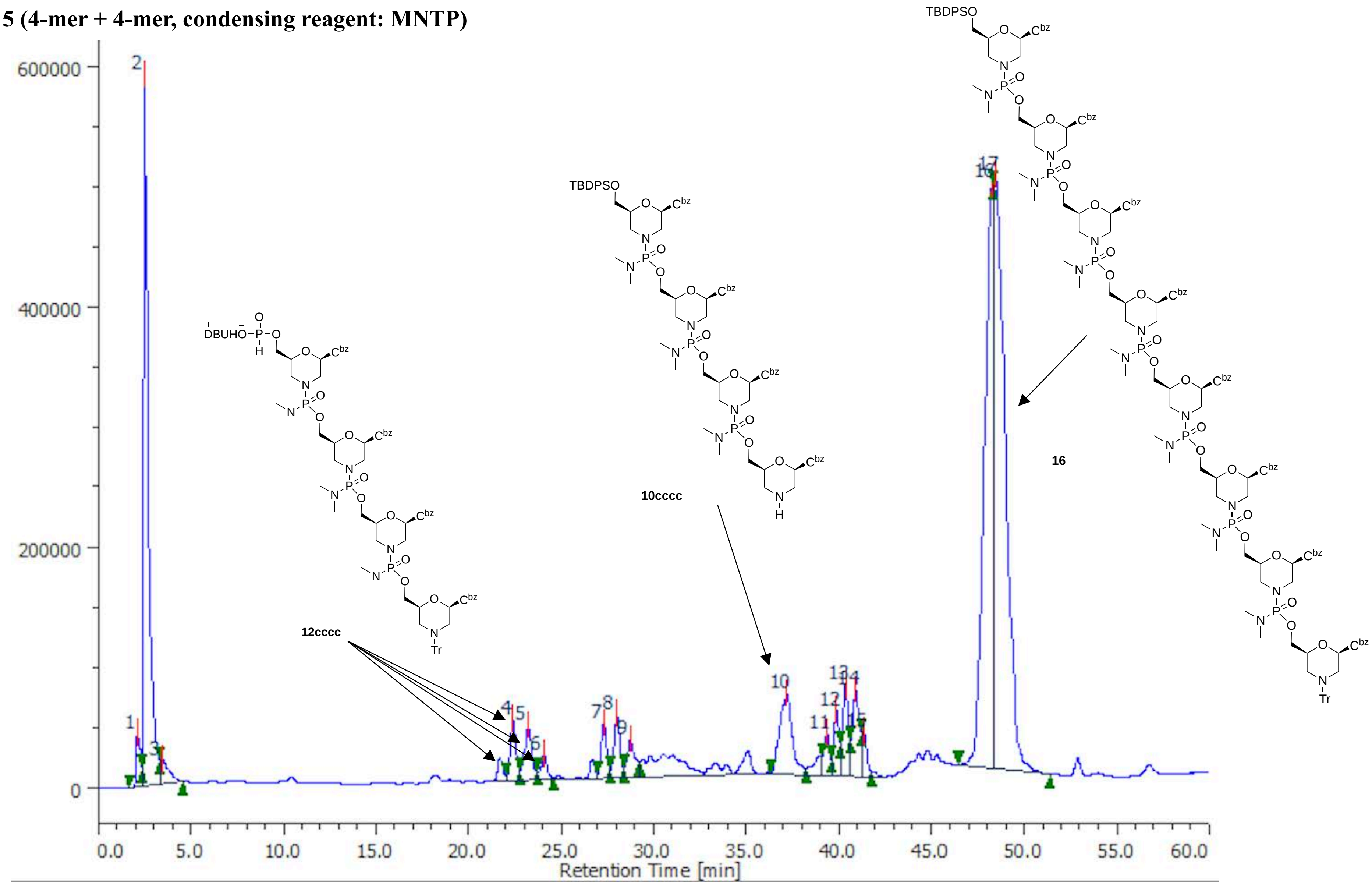

Fig. S 28 HPLC profile of the crude 8-mer. RP-HPLC was performed with a linear gradient of 40%–100% CH_3CN in 0.1 M TEAA buffer (pH 7.0) over 60 min at 50 °C at a rate of 0.5 mL/min.

Table 4, entry 6 (4-mer + 4-mer, GTCAGTCA, condensing reagent: MNTP)

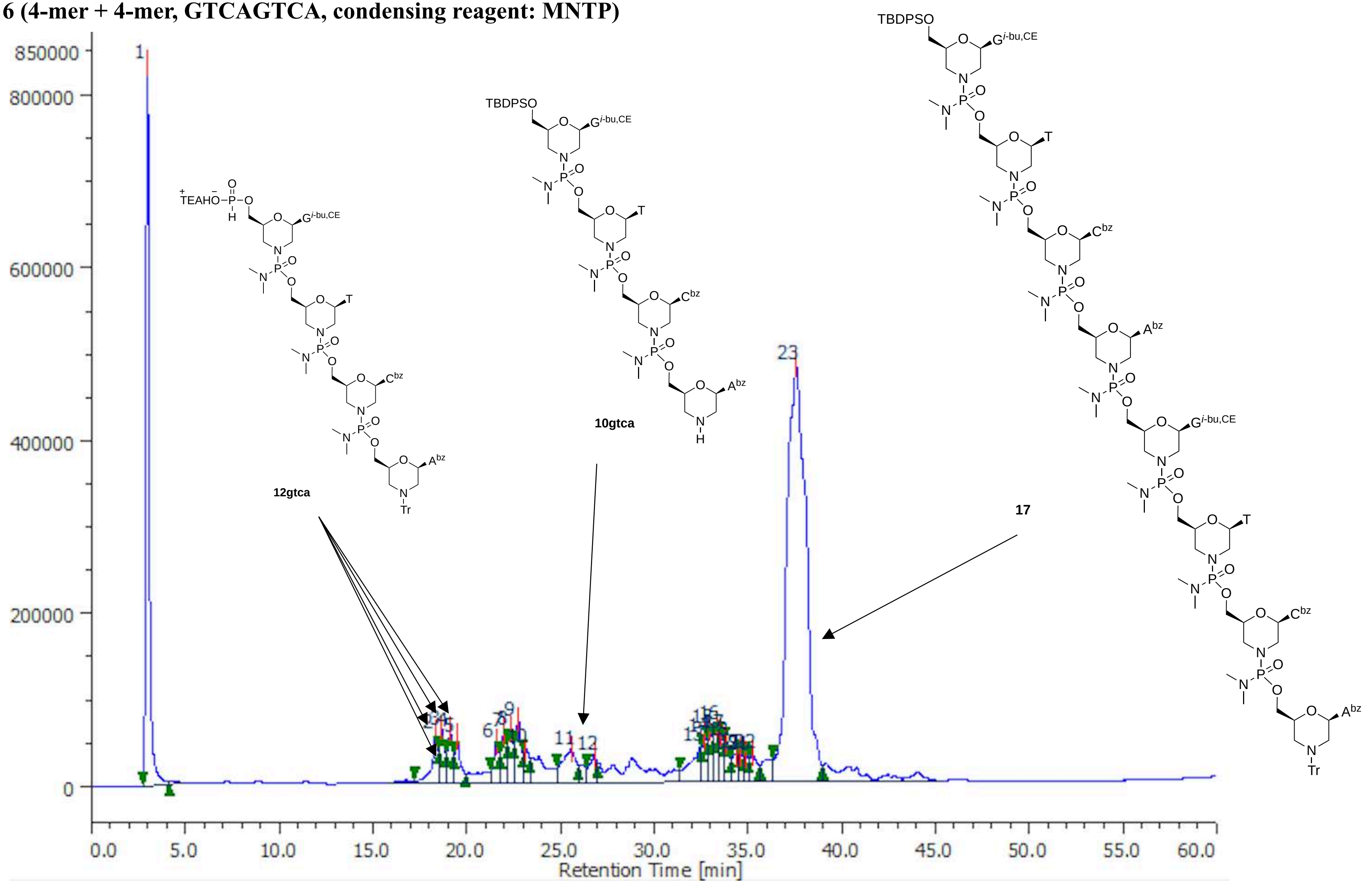

Fig. S 29 HPLC profile of the crude 8-mer. RP-HPLC was performed with a linear gradient of 40%–100% CH_3CN in 0.1 M TEAA buffer (pH 7.0) over 60 min at 50 °C at a rate of 0.5 mL/min.

5. HPLC analysis of isolated compounds

- **2-mer fragment bearing 3′-NH group (5cc)**

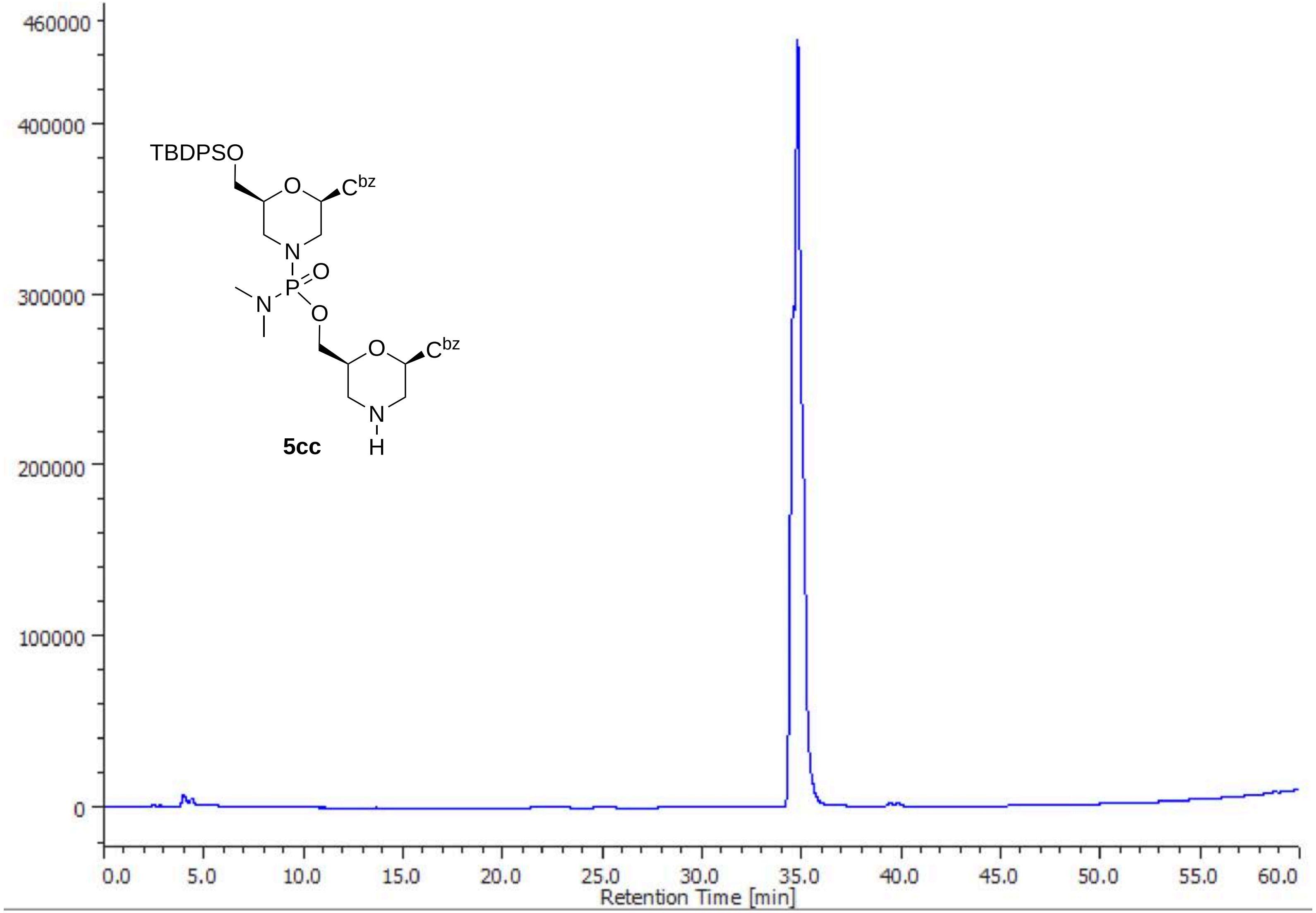

Fig. S 30 HPLC profile of **5cc**. RP-HPLC was performed with a linear gradient of 40%–100% CH_3CN in 0.1 M TEAA buffer (pH 7.0) over 60 min at 50 °C at a rate of 0.5 mL/min.

- **2-mer fragment bearing 3′-NH group (5gt)**

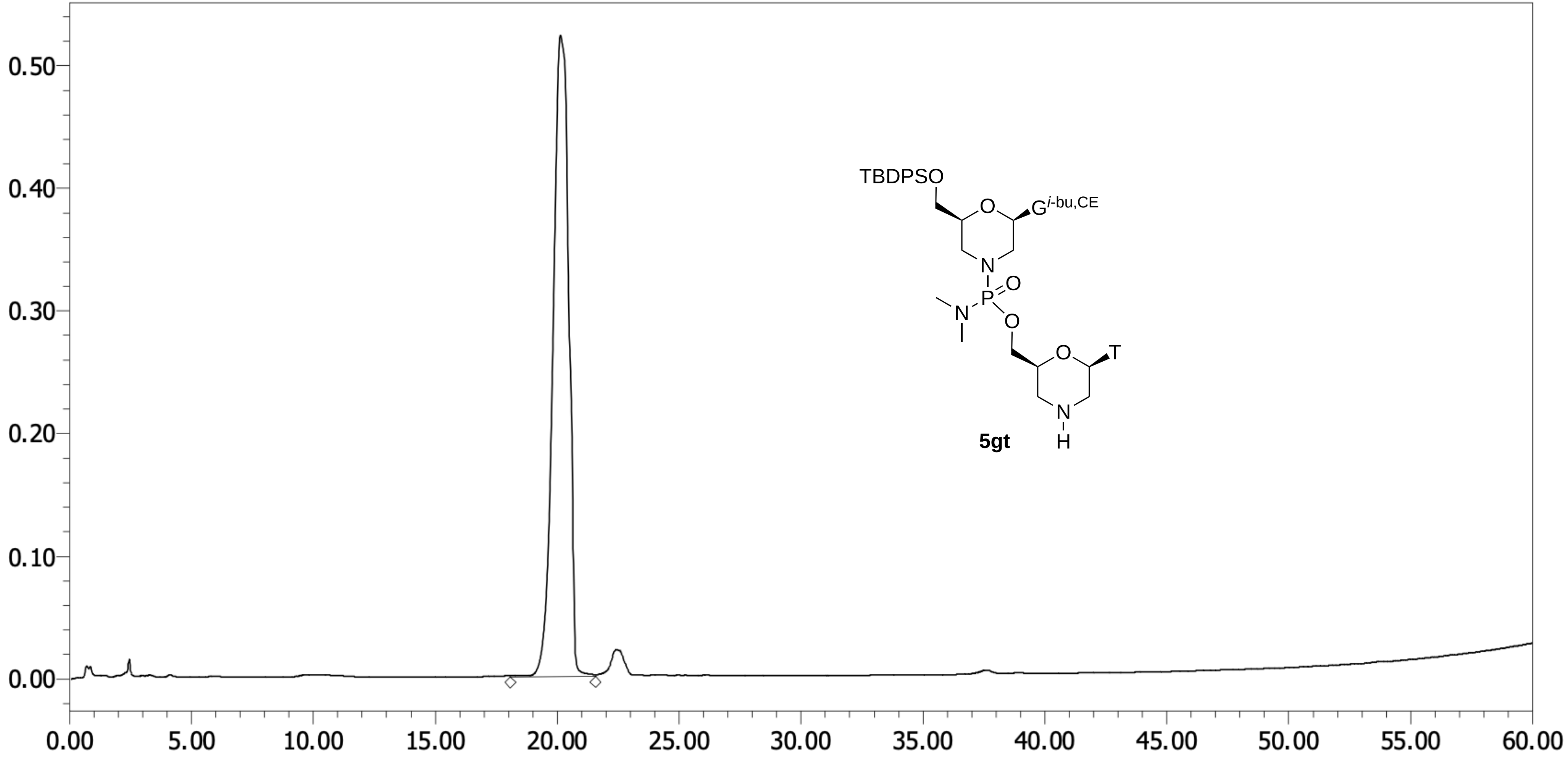

Fig. S 31 HPLC profile of **5gt**. RP-HPLC was performed with a linear gradient of 40%–100% CH_3CN in 0.1 M TEAA buffer (pH 7.0) over 60 min at 50 °C at a rate of 0.5 mL/min.

- **2-mer fragment bearing *H*-phosphonate monoester on 5′-OH group (7cc)**

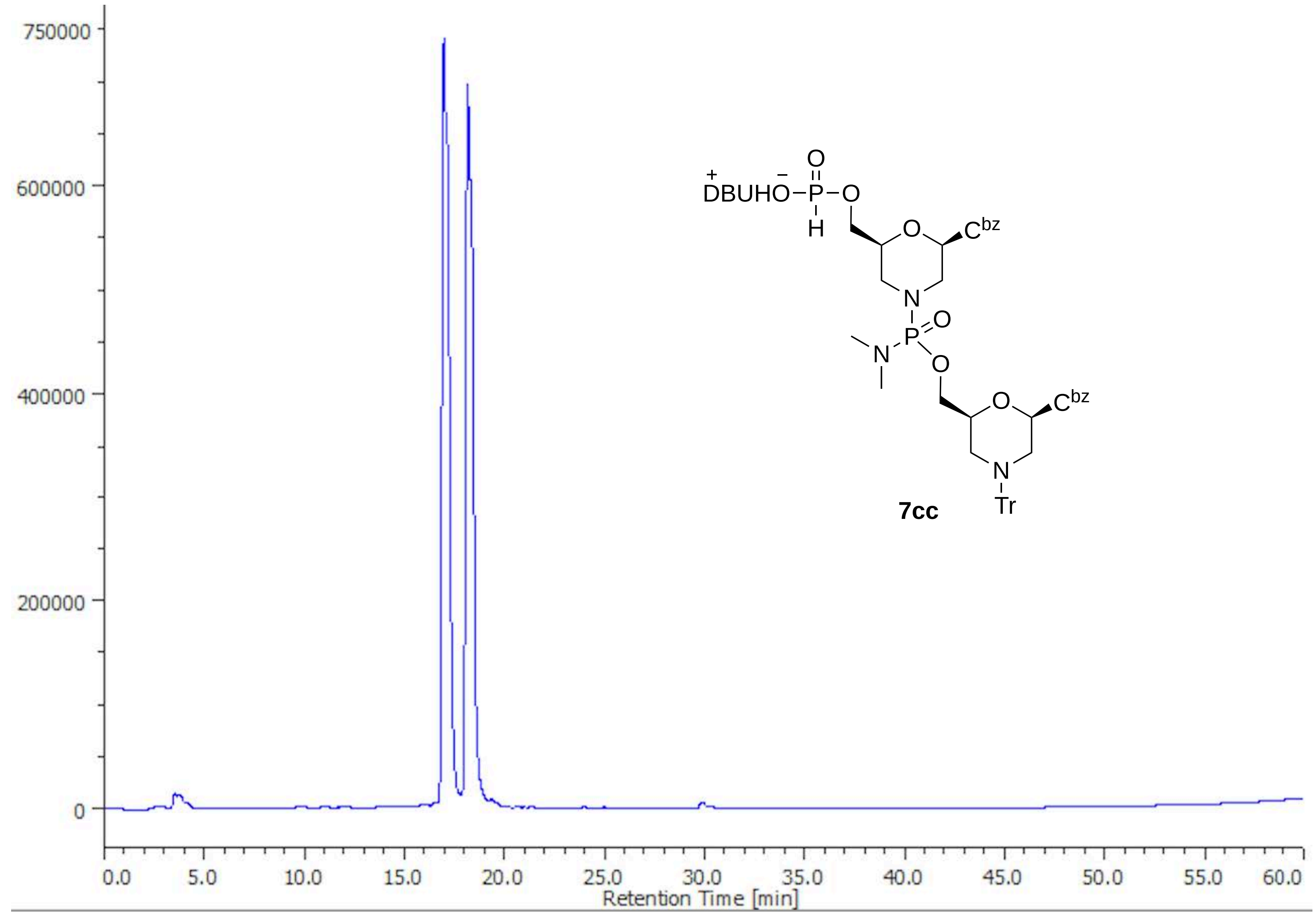

Fig. S 32 HPLC profile of **7cc**. RP-HPLC was performed with a linear gradient of 40%–100% CH_3CN in 0.1 M TEAA buffer (pH 7.0) over 60 min at 50 °C at a rate of 0.5 mL/min.

- **2-mer fragment bearing *H*-phosphonate monoester on 5′-OH group (7ca)**

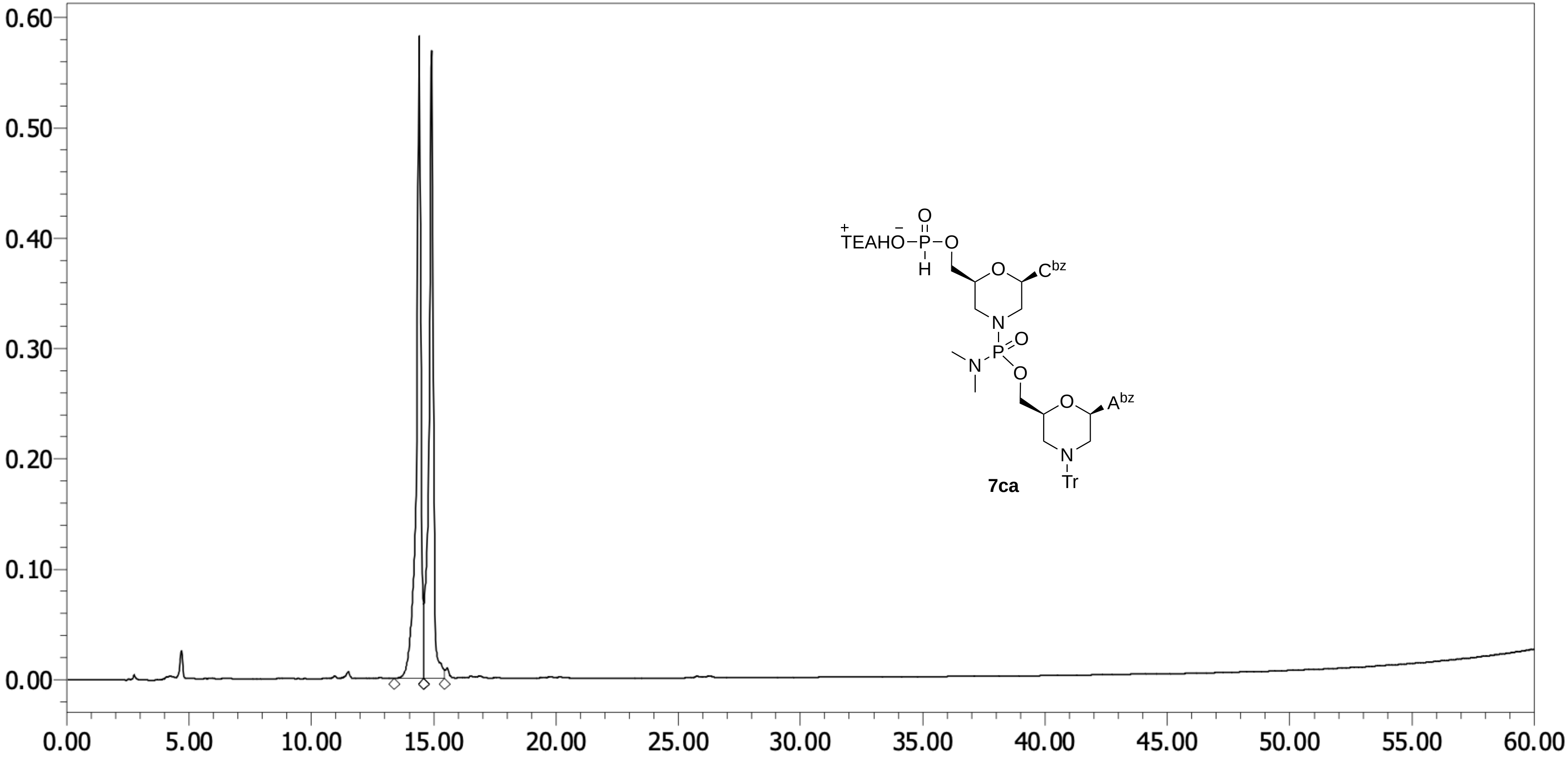

Fig. S 33 HPLC profile of **7ca**. RP-HPLC was performed with a linear gradient of 40%–100% CH_3CN in 0.1 M TEAA buffer (pH 7.0) over 60 min at 50 °C at a rate of 0.5 mL/min.

- **4-mer fragment bearing 3′-NH group (10cccc)**

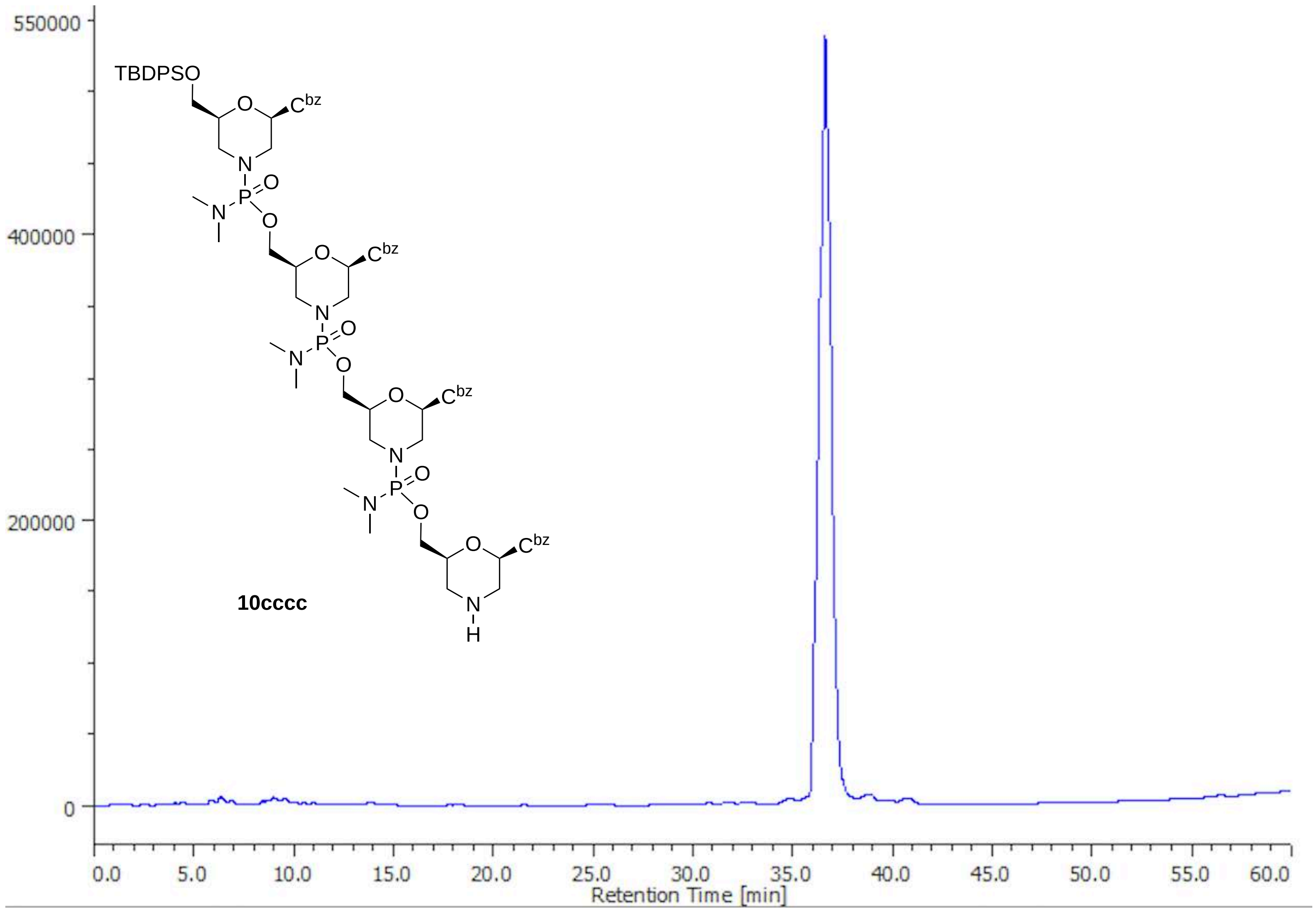

Fig. S 34 HPLC profile of **10cccc**. RP-HPLC was performed with a linear gradient of 40%–100% CH_3CN in 0.1 M TEAA buffer (pH 7.0) over 60 min at 50 °C at a rate of 0.5 mL/min.

- **4-mer fragment bearing 3′-NH group (10gtca)**

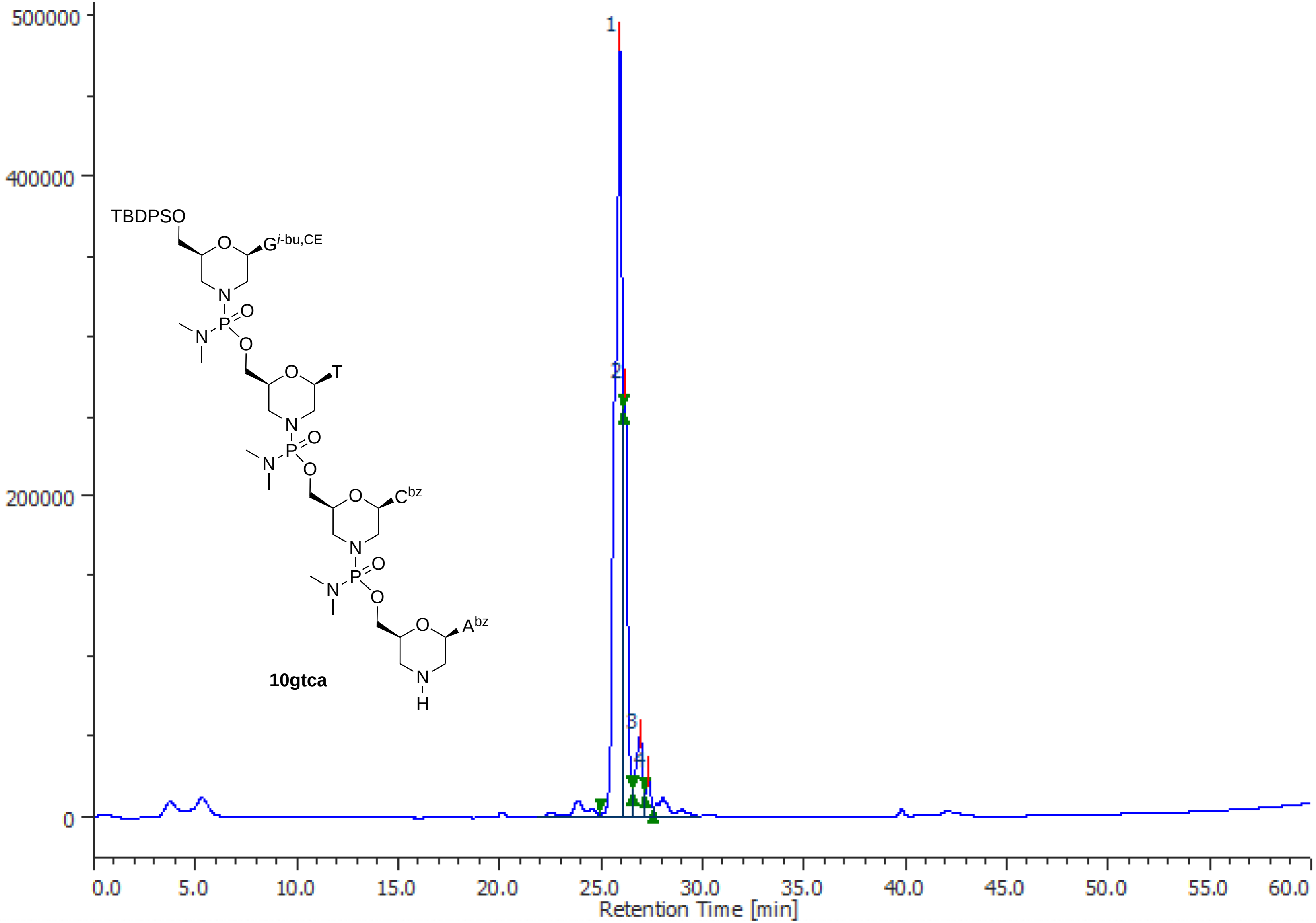

Fig. S 35 HPLC profile of **10gtca**. RP-HPLC was performed with a linear gradient of 40%–100% CH_3CN in 0.1 M TEAA buffer (pH 7.0) over 60 min at 50 °C at a rate of 0.5 mL/min.

- **4-mer fragment bearing *H*-phosphonate monoester on 5′-OH group (12cccc)**

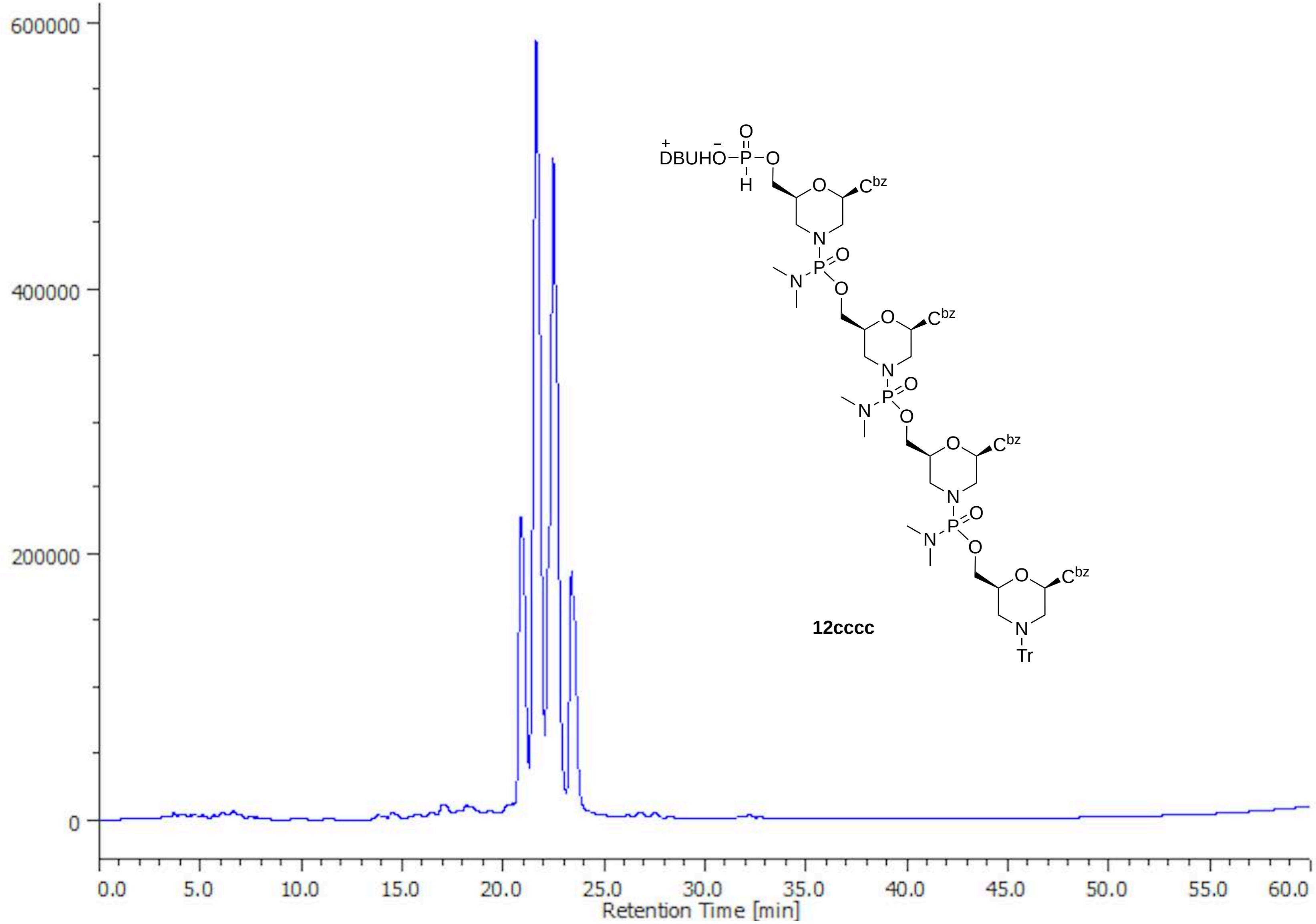

Fig. S 36 HPLC profile of **12cccc**. RP-HPLC was performed with a linear gradient of 40%–100% CH_3CN in 0.1 M TEAA buffer (pH 7.0) over 60 min at 50 °C at a rate of 0.5 mL/min.

- **4-mer fragment bearing *H*-phosphonate monoester on 5′-OH group (12gtca)**

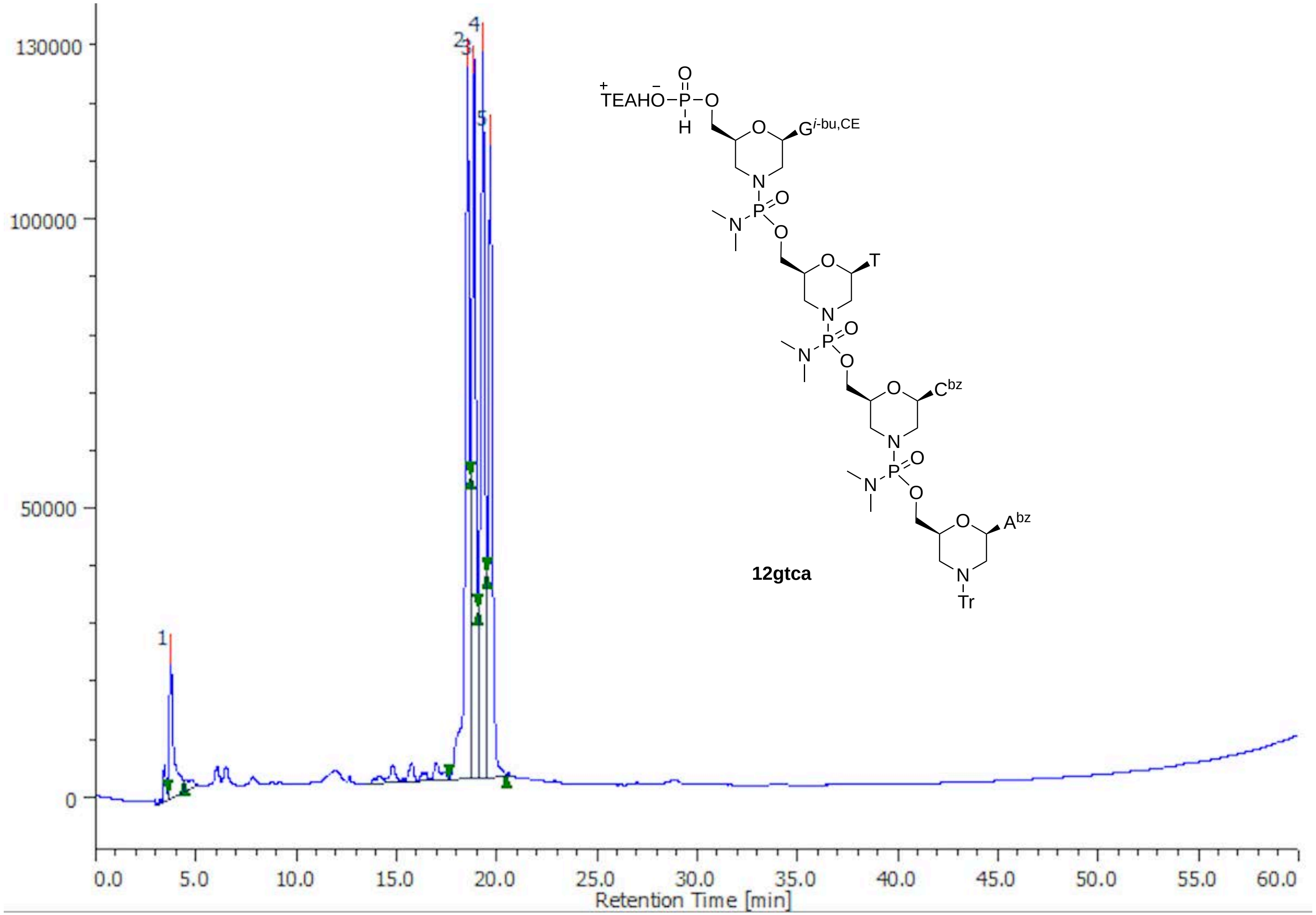

Fig. S 37 HPLC profile of **12gtca**. RP-HPLC was performed with a linear gradient of 40%–100% CH_3CN in 0.1 M TEAA buffer (pH 7.0) over 60 min at 50 °C at a rate of 0.5 mL/min.

- **8-mer (18)**

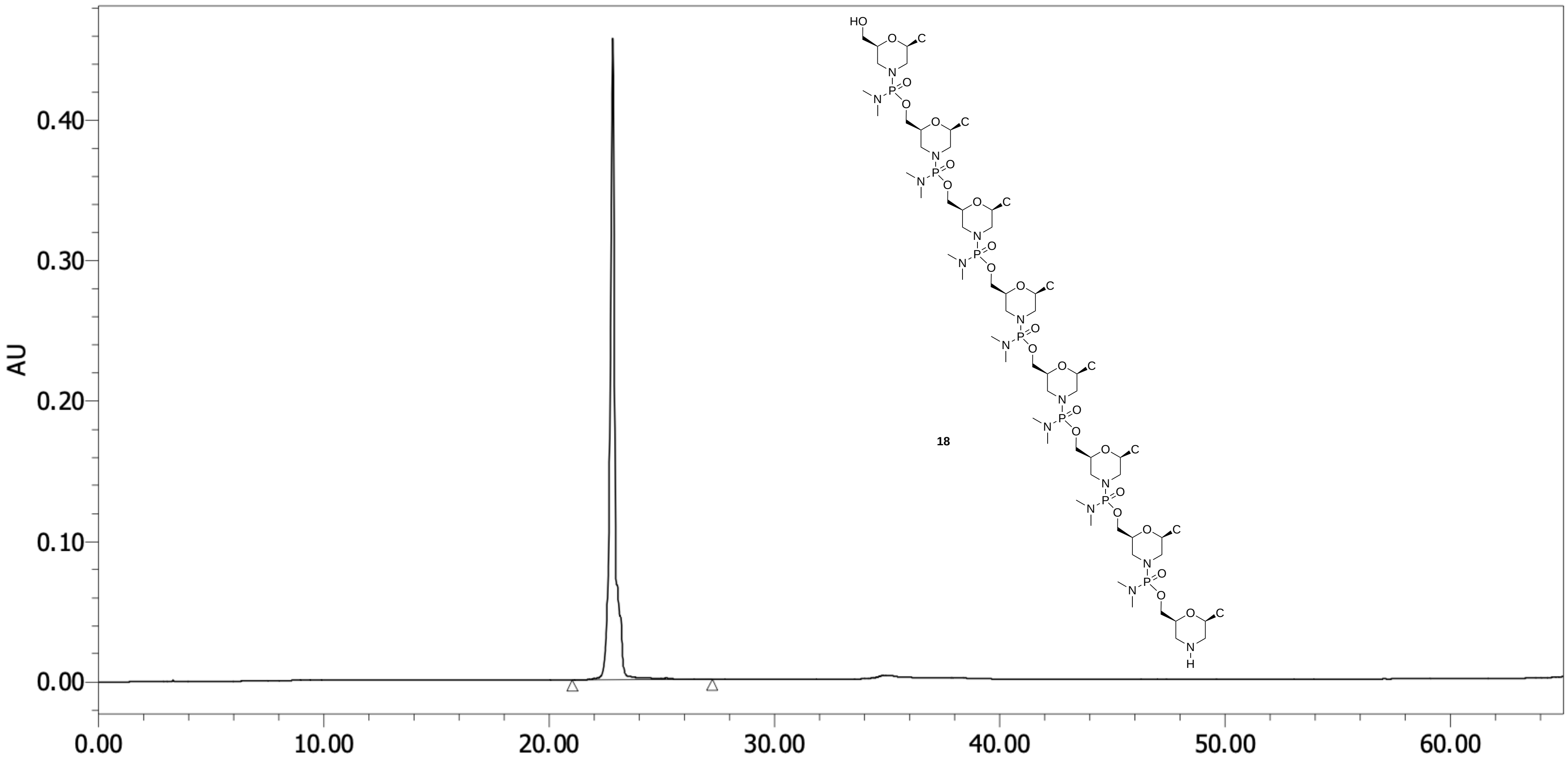

Fig. S 38 HPLC profile of **18**. RP-HPLC was performed with a linear gradient of 0%–60% CH_3CN in 0.1 M TEAA buffer (pH 7.0) over 60 min at 50 °C at a rate of 0.5 mL/min.

- **8-mer (19)**

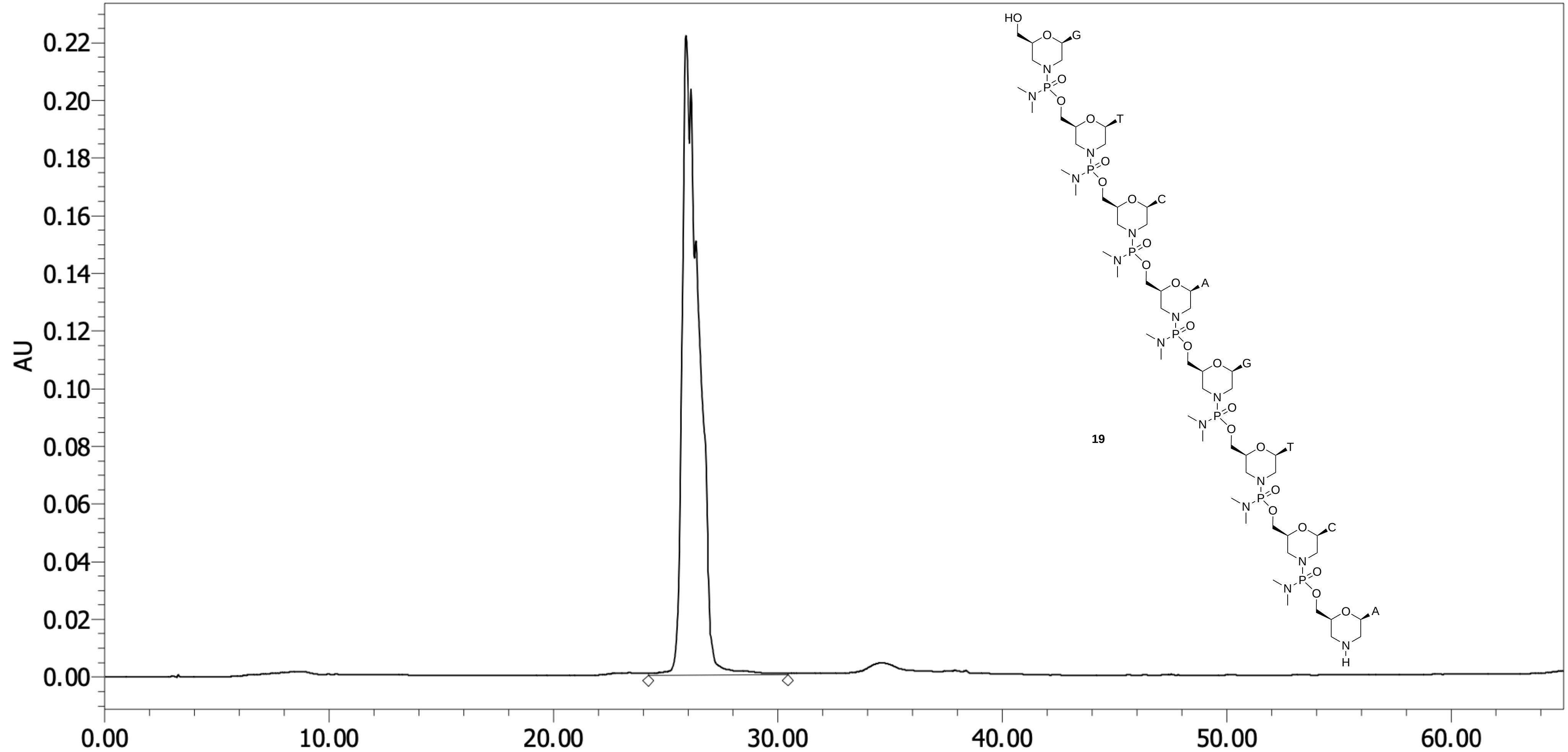

Fig. S 39 HPLC profile of **19**. RP-HPLC was performed with a linear gradient of 0%–60% CH_3CN in 0.1 M TEAA buffer (pH 7.0) over 60 min at 50 °C at a rate of 0.5 mL/min.

6. ^{1}H, ^{13}C, ^{31}P NMR spectra of isolated compounds

^{1}H-NMR (400 MHz, $CDCl_3$)

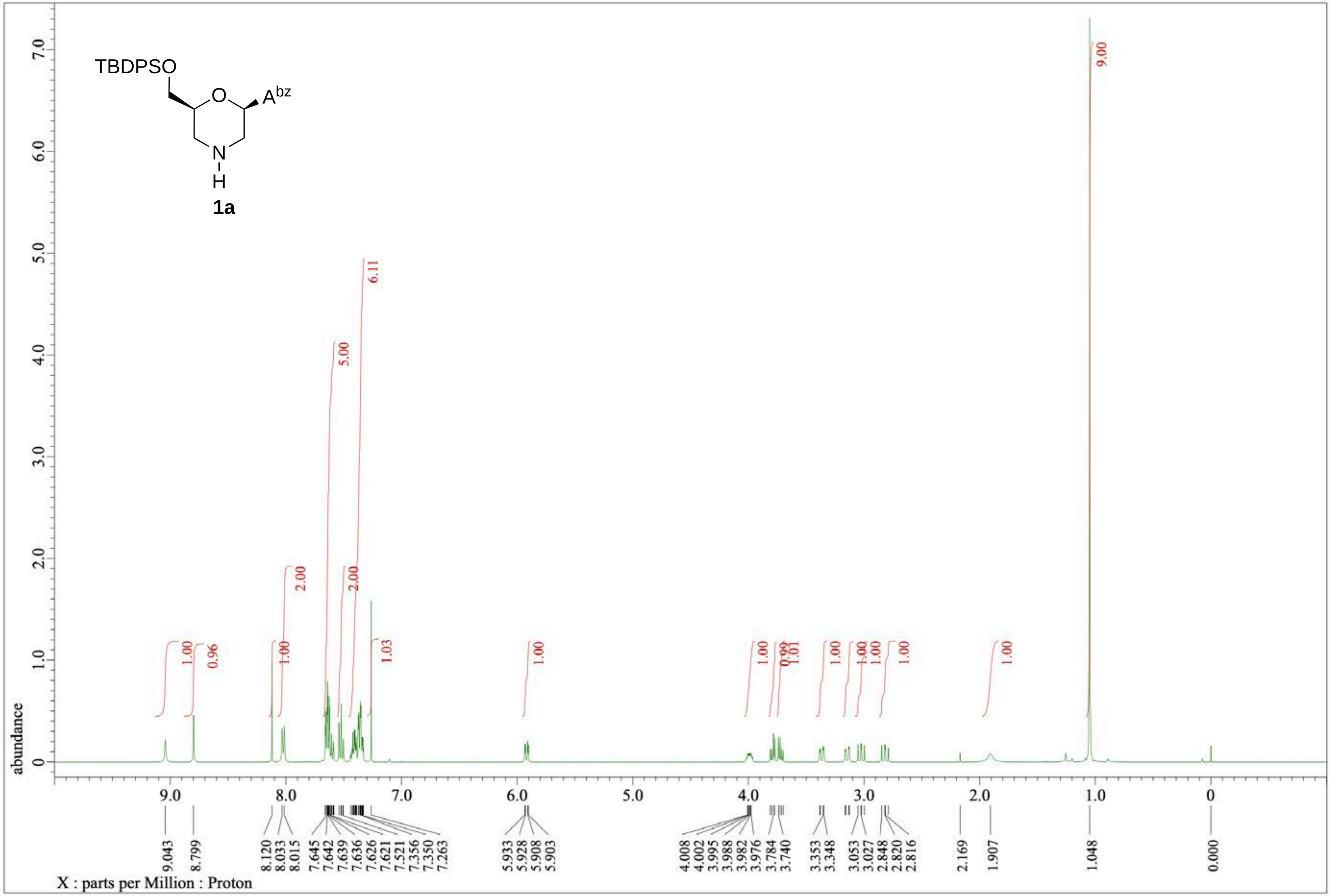

$^{13}C\{H\}$-NMR (101 MHz, $CDCl_3$)

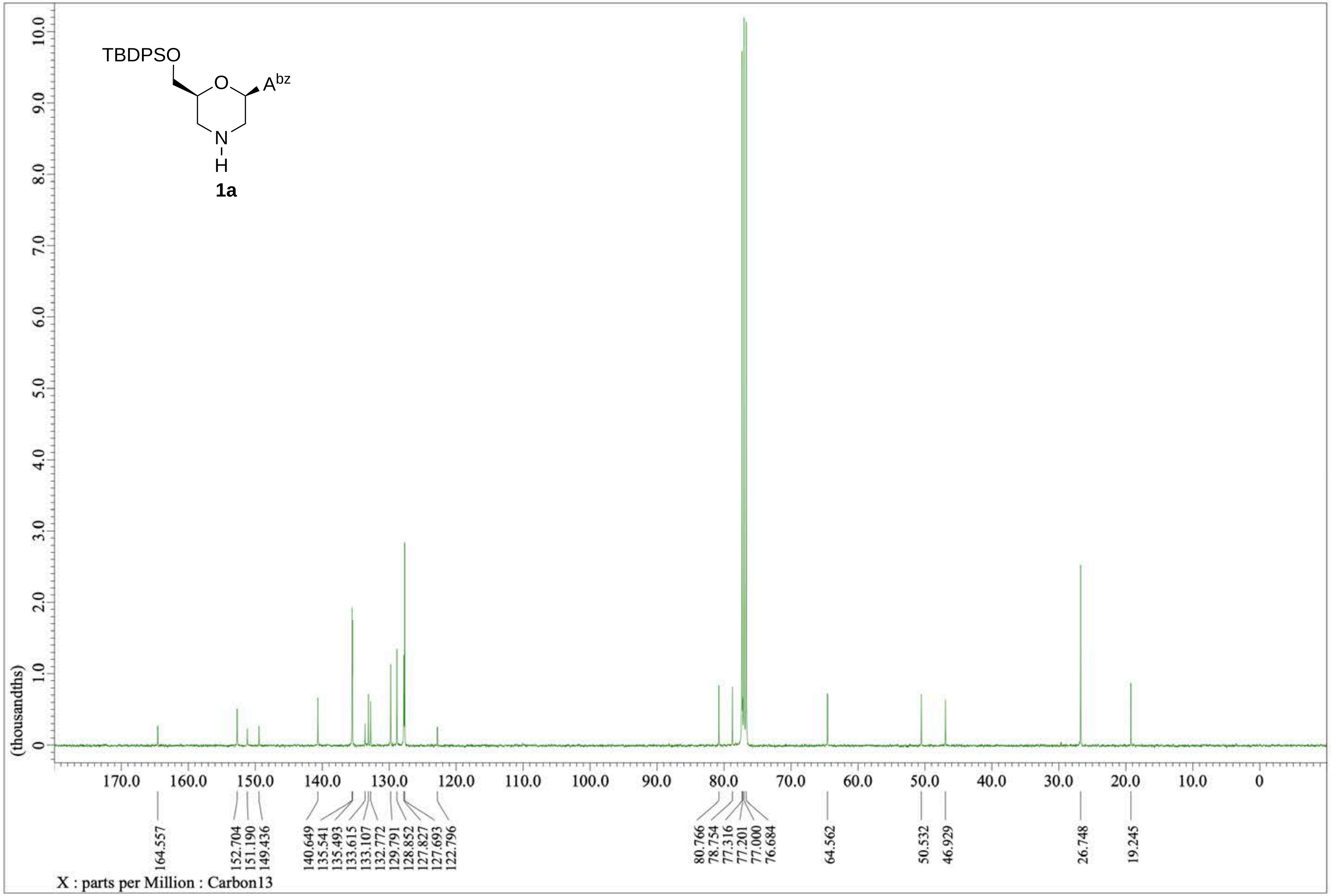

HMQC ($CDCl_3$)

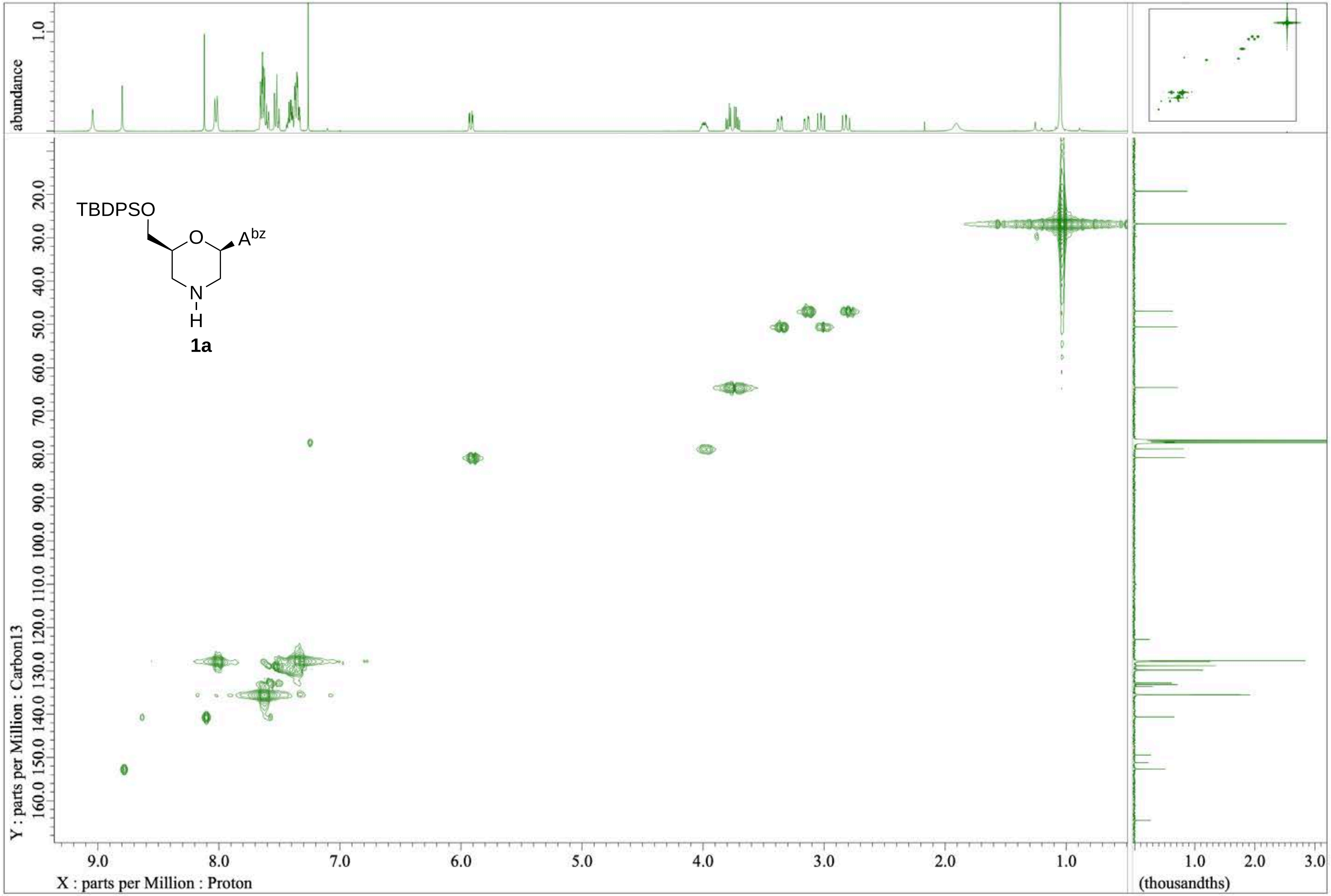
abundance
1.0
Y : parts per Million : Carbon13
20.0
30.0
40.0
50.0
60.0
70.0
80.0
90.0
100.0
110.0
120.0
130.0
140.0
150.0
160.0
9.0
8.0
7.0
6.0
5.0
4.0
3.0
2.0
1.0
X : parts per Million : Proton
1.0
2.0
3.0
(thousandths)
TBDPSO
O
A^bz
N
H
1a

HMBC ($CDCl_3$)

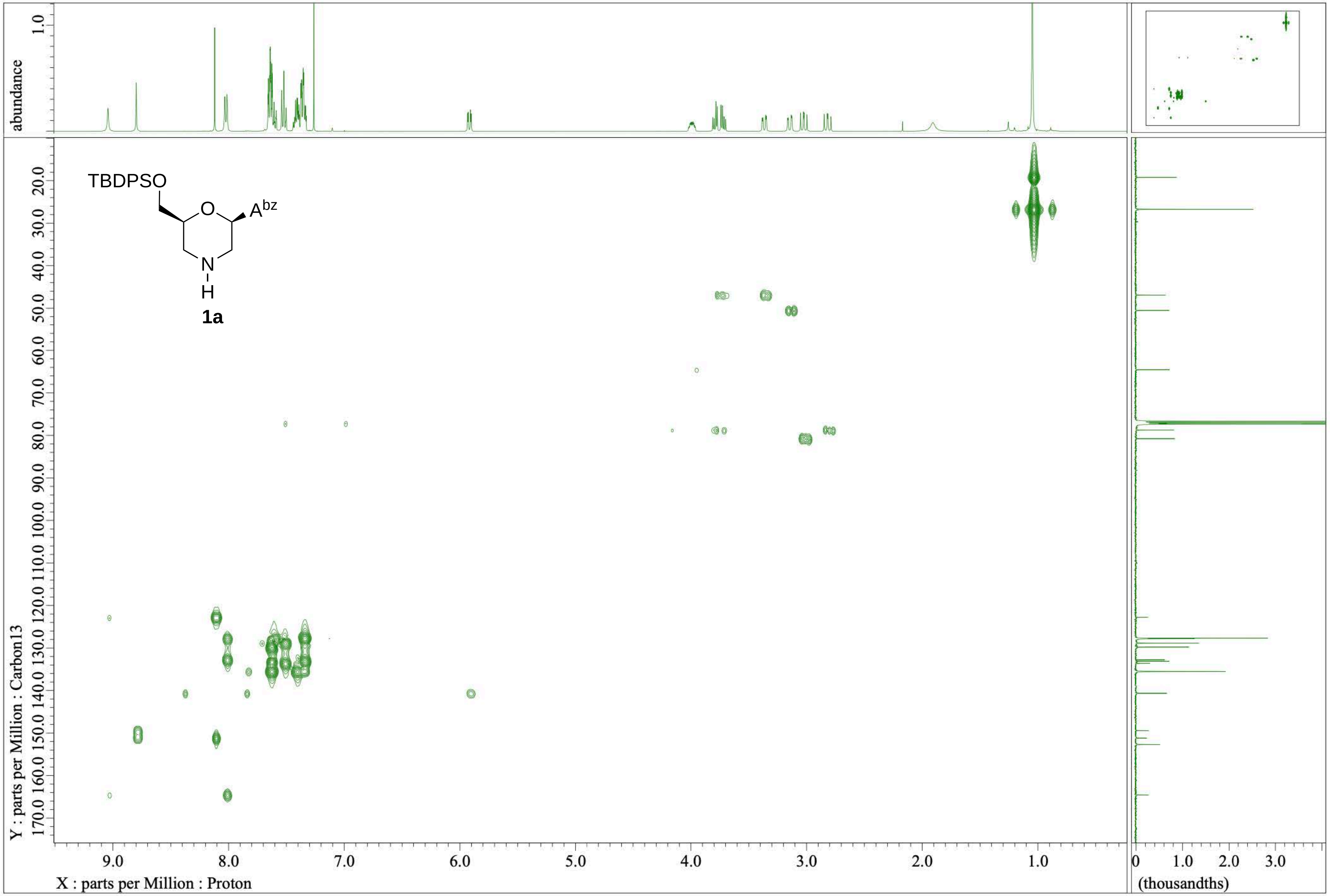
abundance
1.0
TBDPSO
O
A^bz
N
H
1a
Y : parts per Million : Carbon13
20.0
30.0
40.0
50.0
60.0
70.0
80.0
90.0
100.0
110.0
120.0
130.0
140.0
150.0
160.0
170.0
9.0
8.0
7.0
6.0
5.0
4.0
3.0
2.0
1.0
X : parts per Million : Proton
0
1.0
2.0
3.0
(thousandths)

Mass spectra

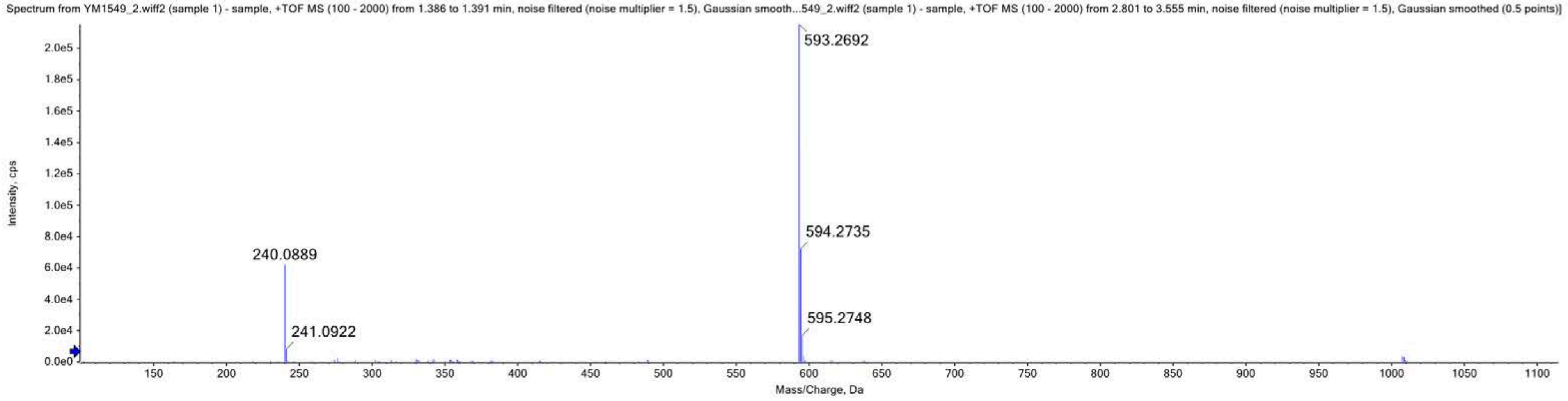

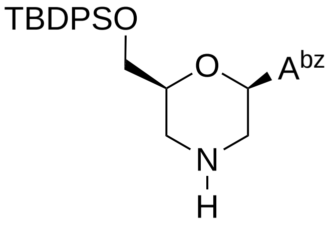

1a

ESI-MS *m/z* calcd for $C_{33}H_{37}N_6O_3Si$ $[M+H]^+$, 593.2691; found 593.2692.

^{1}H-NMR (400 MHz, $CDCl_3$)

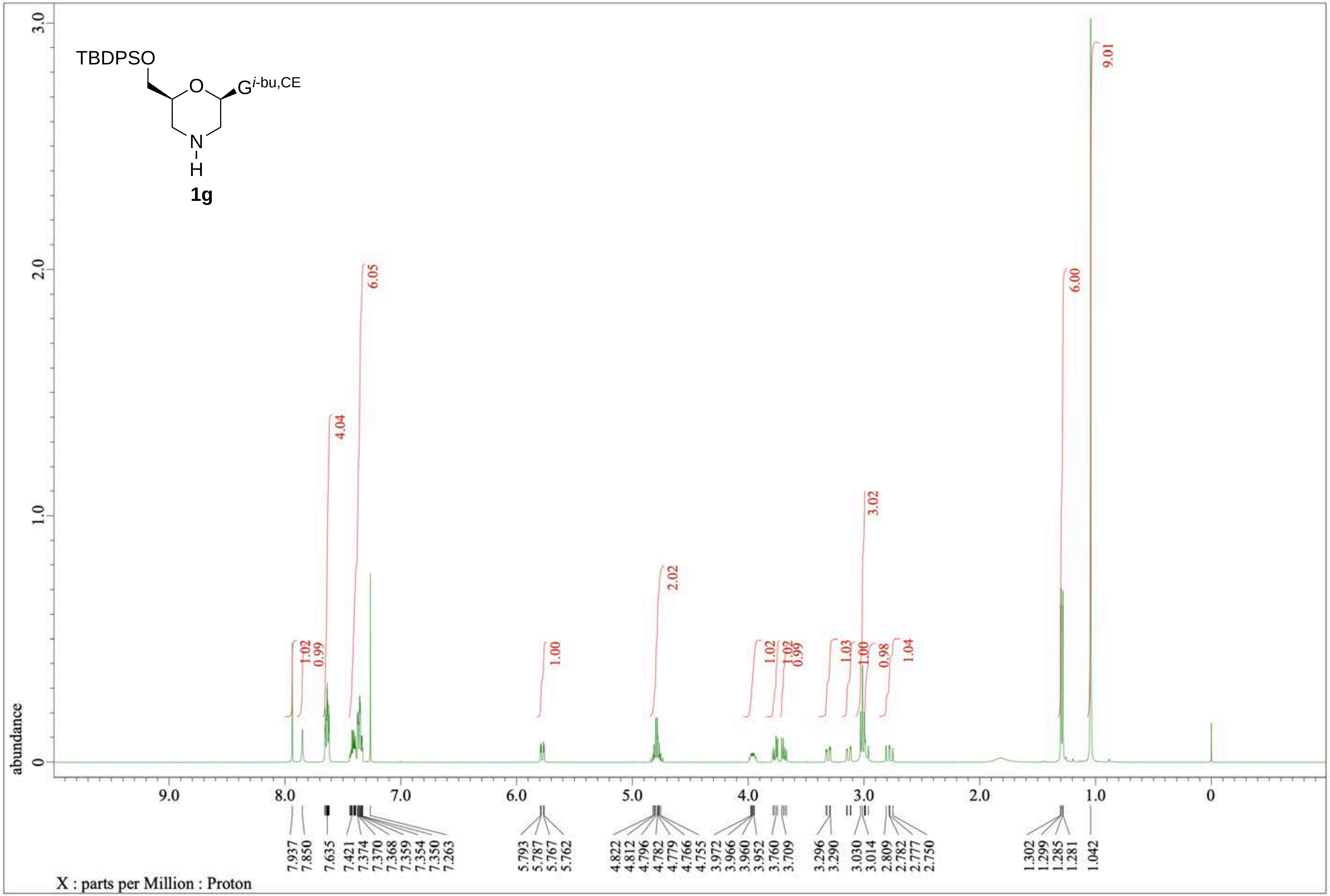

$^{13}C\{H\}$-NMR (101 MHz, $CDCl_3$)

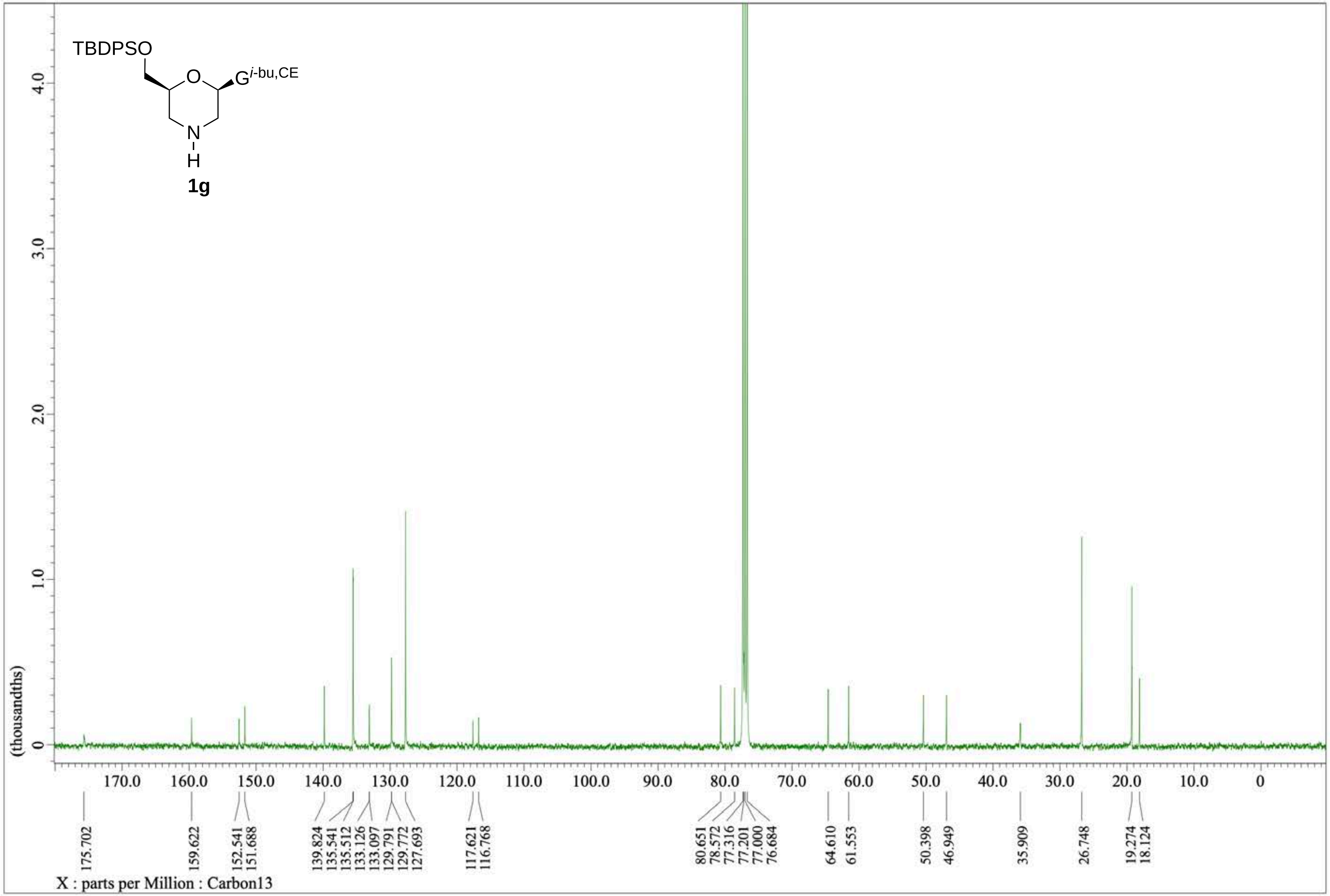

HMQC ($CDCl_3$)

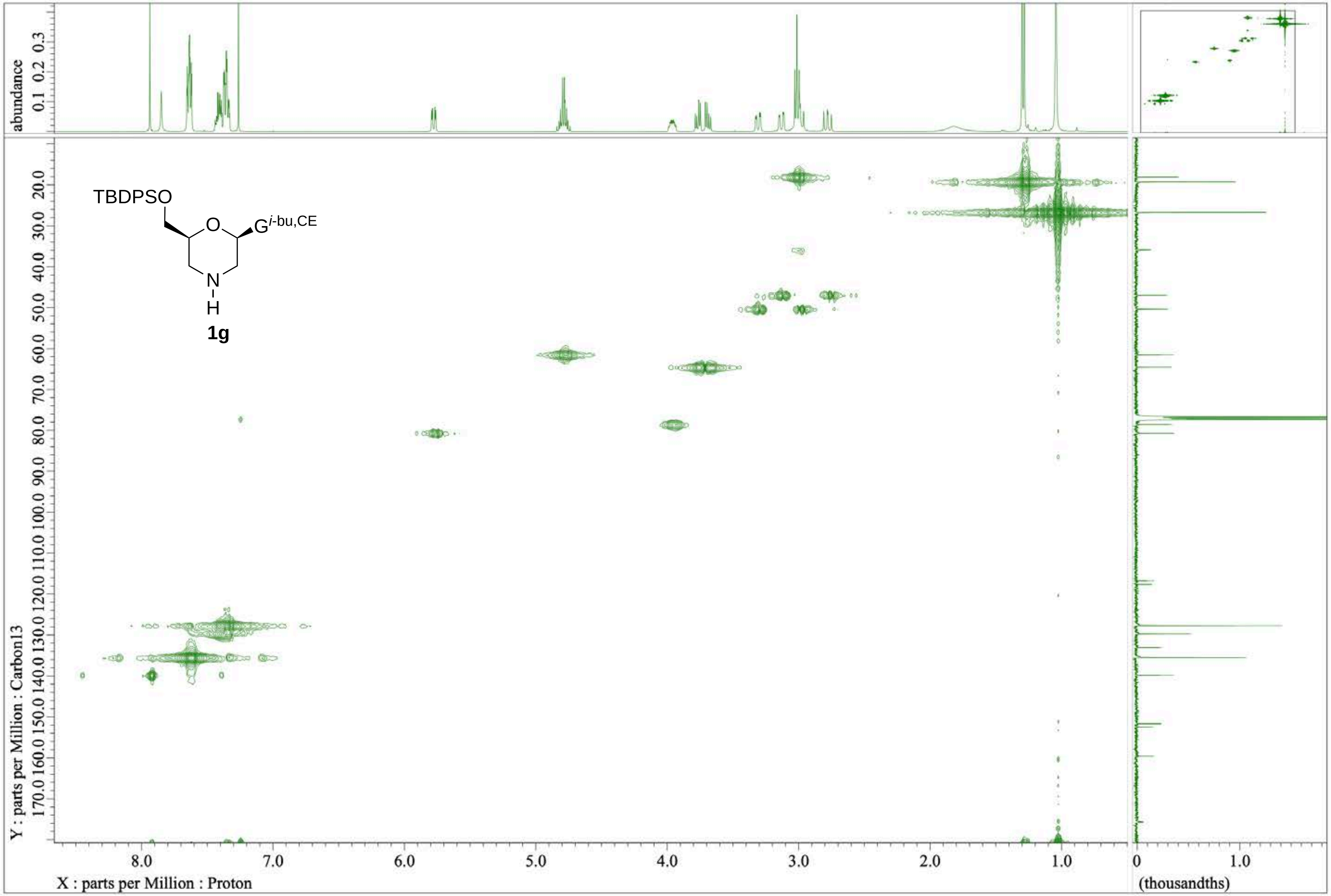

abundance
TBDPSO
O
$G^{i\text{-bu,CE}}$
N
H
1g
Y : parts per Million : Carbon13
X : parts per Million : Proton
(thousandths)

HMBC ($CDCl_3$)

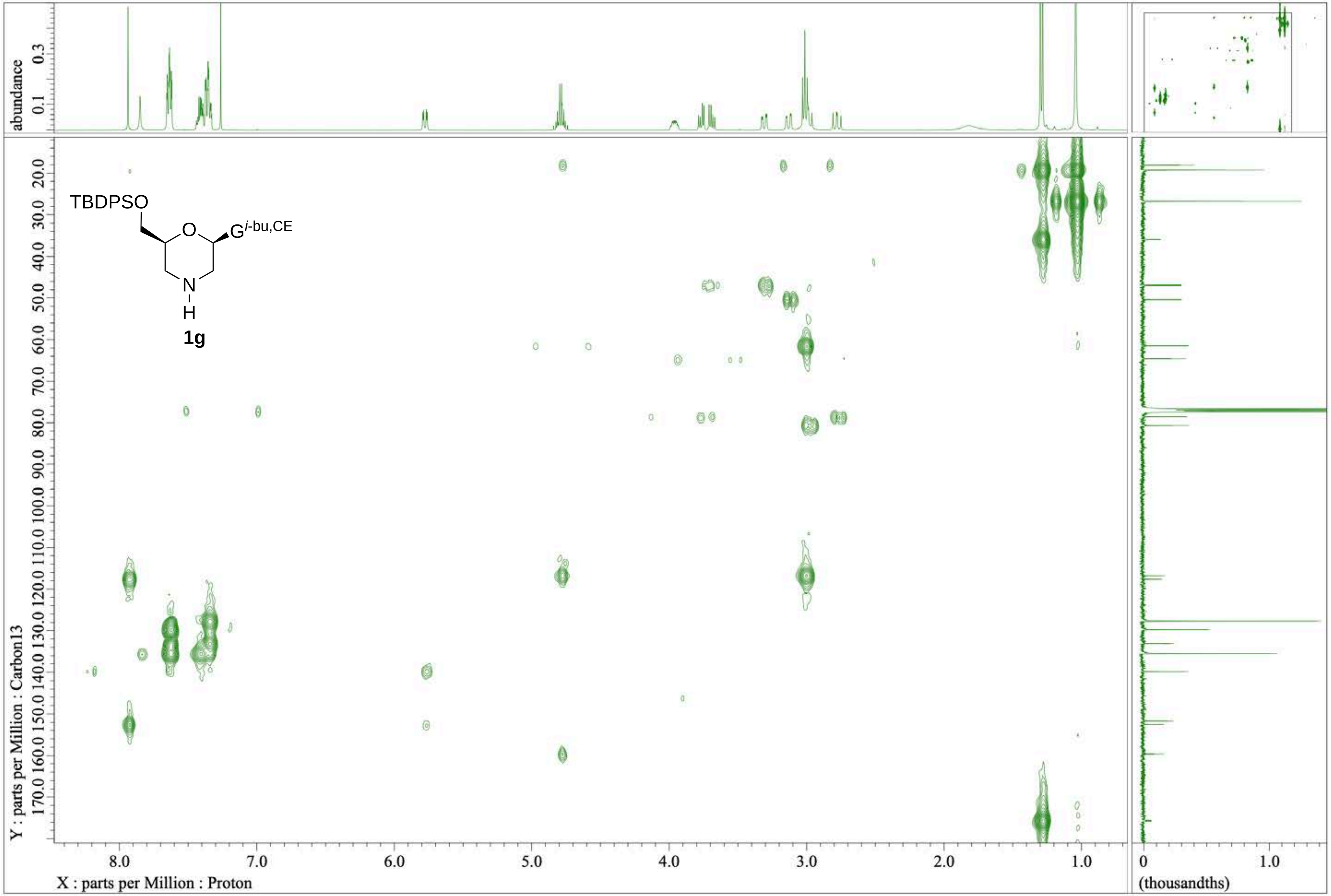

abundance
0.1
0.3
Y : parts per Million : Carbon13
X : parts per Million : Proton
(thousandths)
TBDPSO
O
$G^{i\text{-bu,CE}}$
N
H
1g

Mass spectra

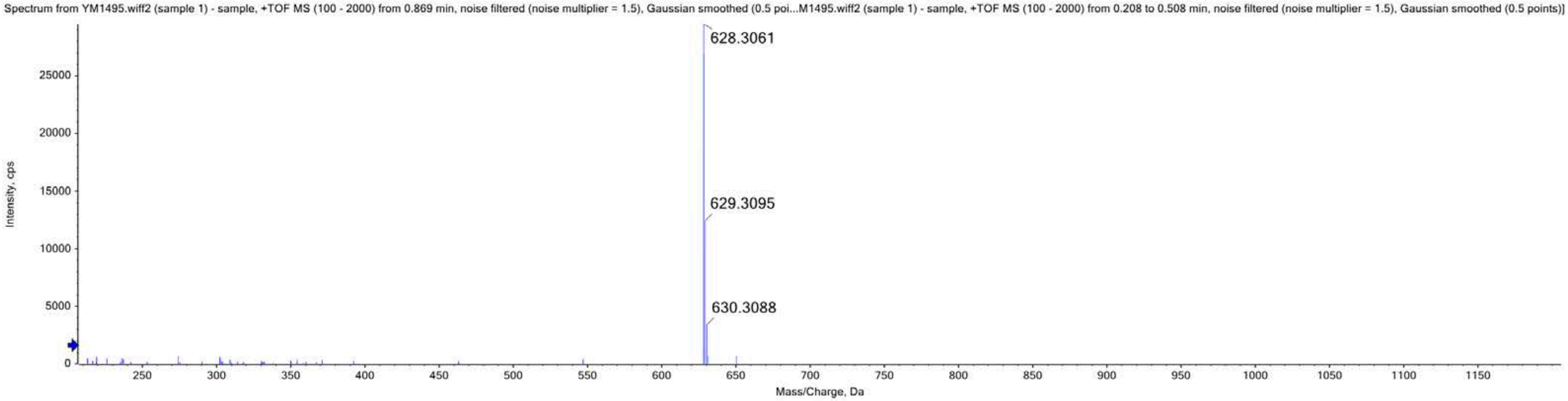

ESI-MS *m/z* calcd for $C_{33}H_{42}N_7O_4Si$ $[M+H]^+$, 628.3062; found 628.3061.

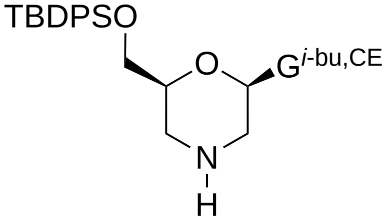

1g

^{1}H-NMR (400 MHz, $CDCl_3$)

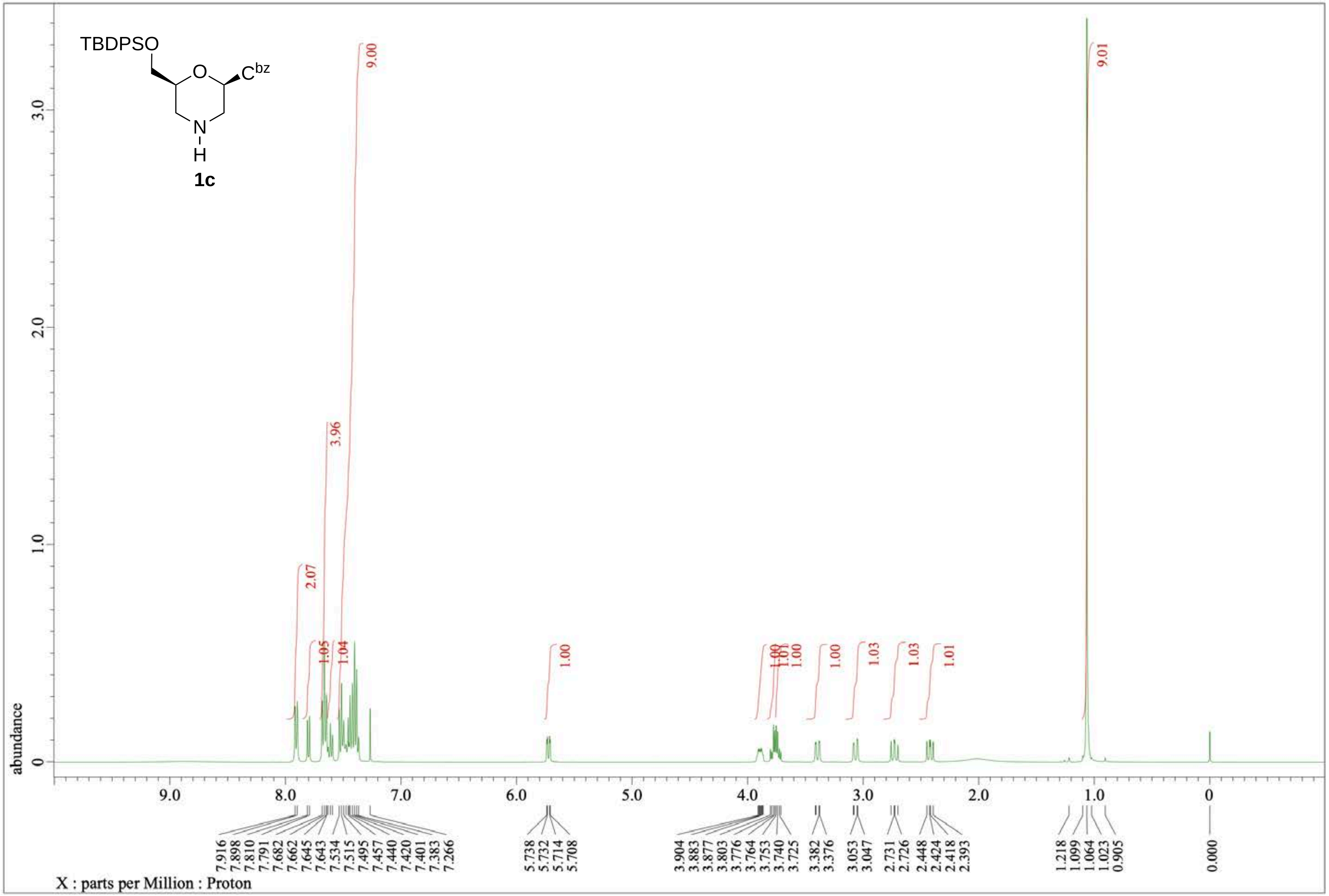

$^{13}C\{H\}$-NMR (101 MHz, $CDCl_3$)

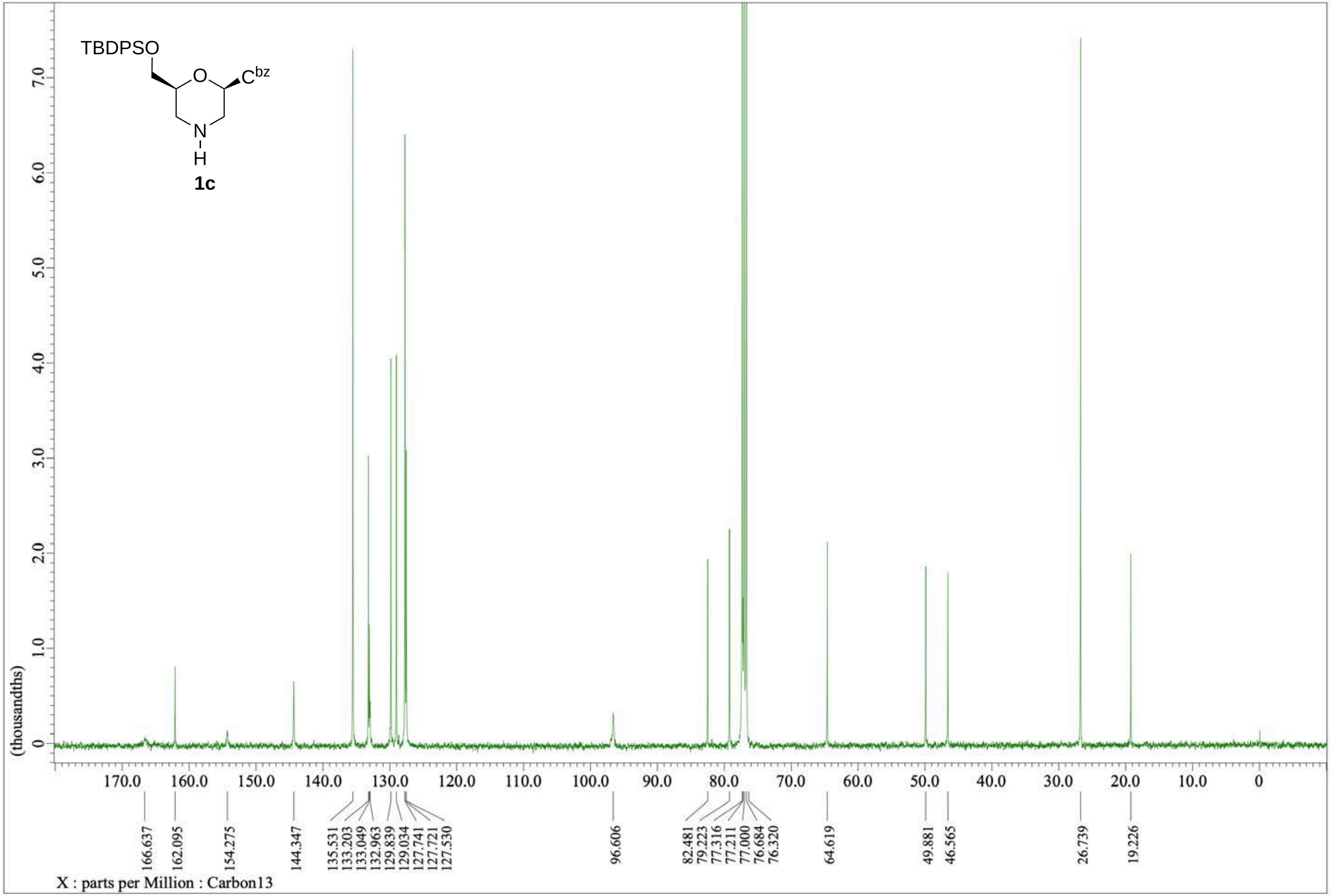

HMQC ($CDCl_3$)

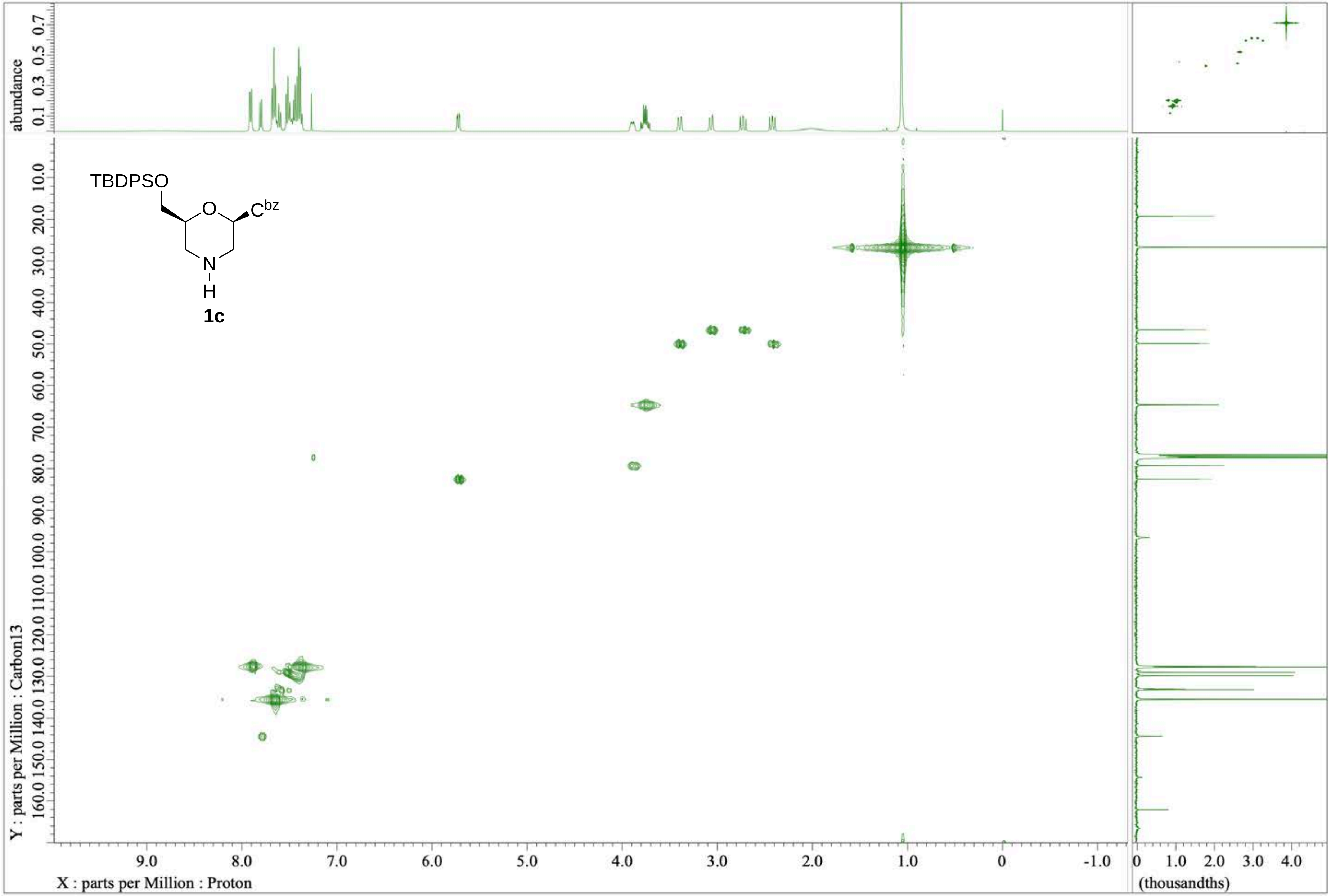

TBDPSO
O
C^bz
N
H
1c
abundance
Y : parts per Million : Carbon13
X : parts per Million : Proton
(thousandths)

HMBC ($CDCl_3$)

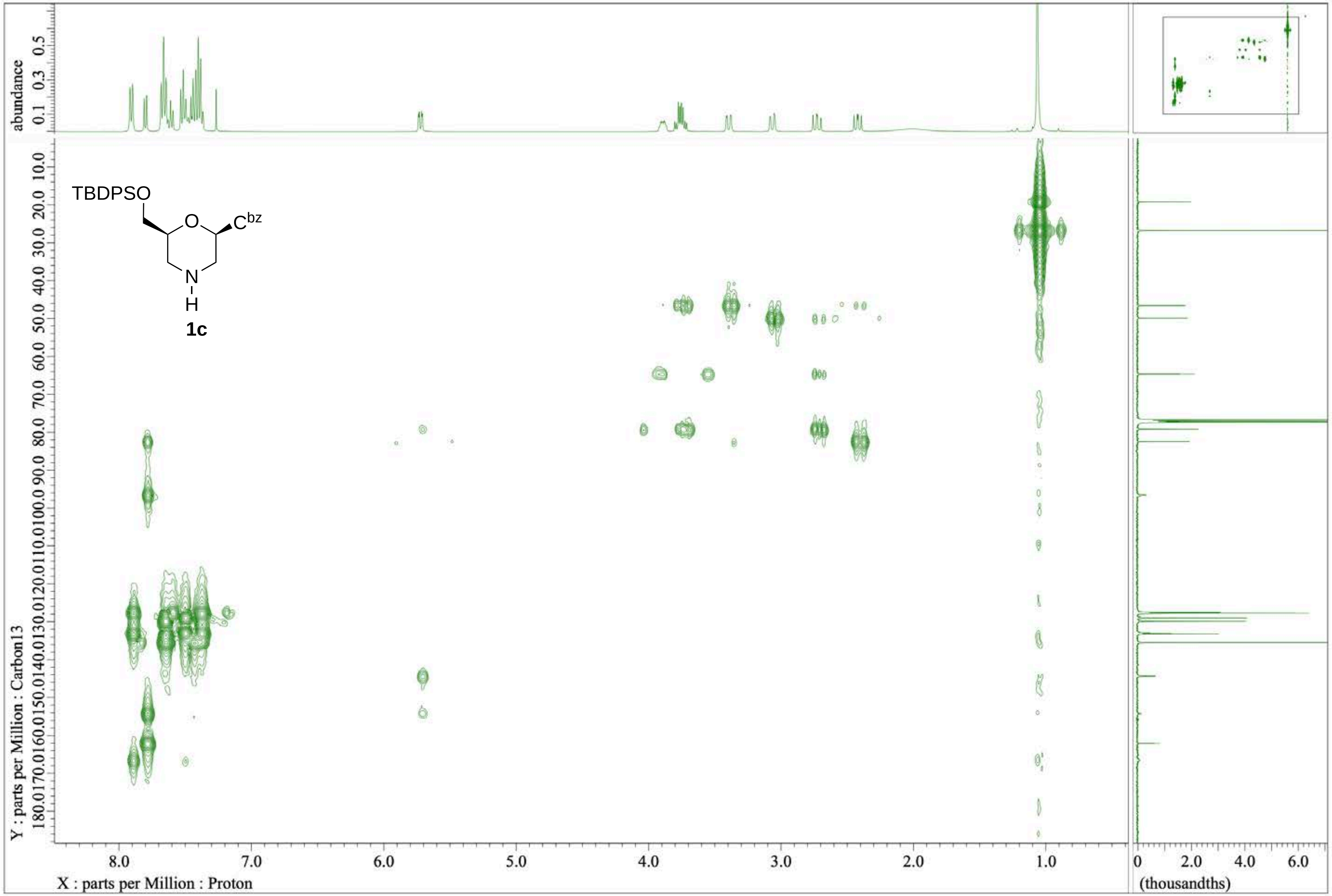

abundance
0.1
0.3
0.5
TBDPSO
O
C^bz
N
H
1c
Y : parts per Million : Carbon13
X : parts per Million : Proton
8.0
7.0
6.0
5.0
4.0
3.0
2.0
1.0
0
2.0
4.0
6.0
(thousandths)

Mass spectra

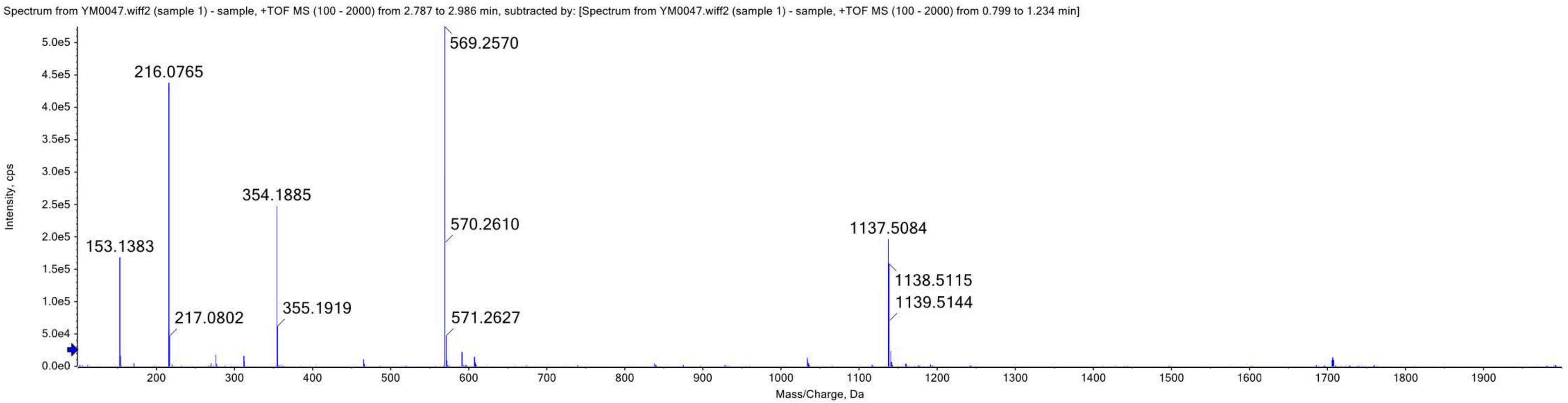

ESI-MS *m/z* calcd for C32H37N4O4Si $[M+H]^+$, 569.2578; found 569.2570.

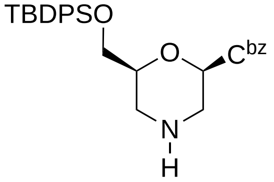

1c

^{1}H-NMR (400 MHz, $CDCl_3$)

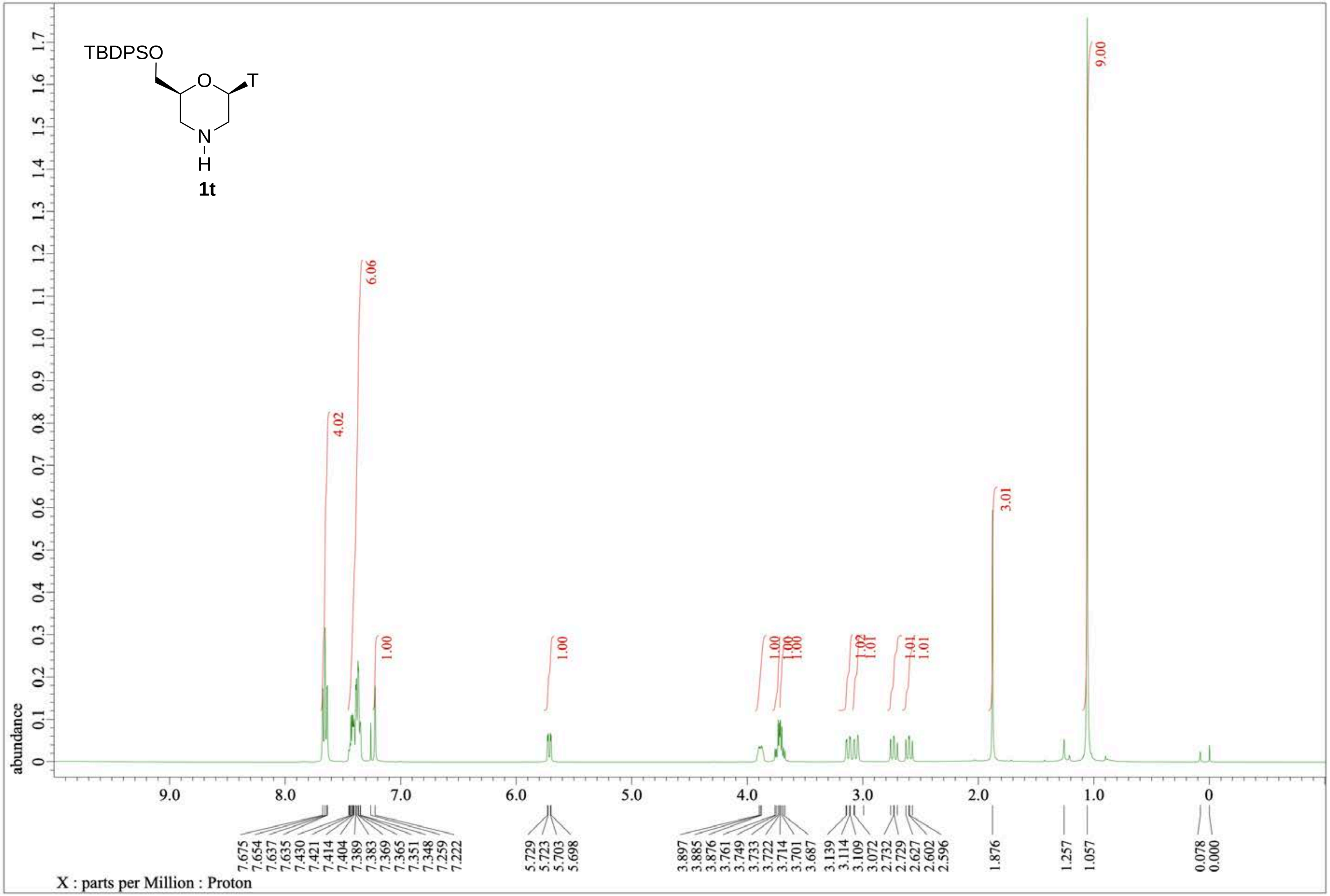

$^{13}C\{H\}$-NMR (101 MHz, $CDCl_3$)

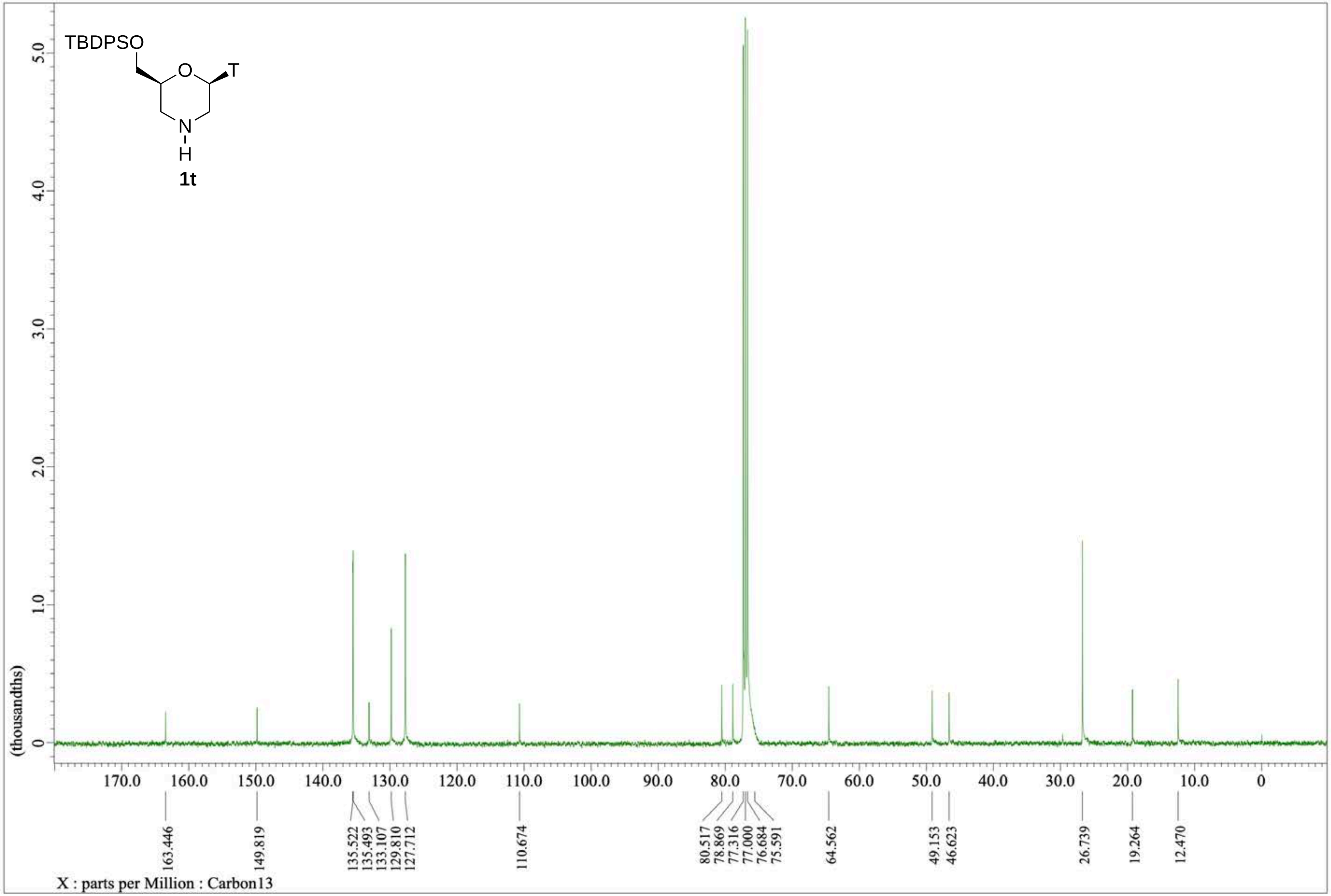

HMQC ($CDCl_3$)

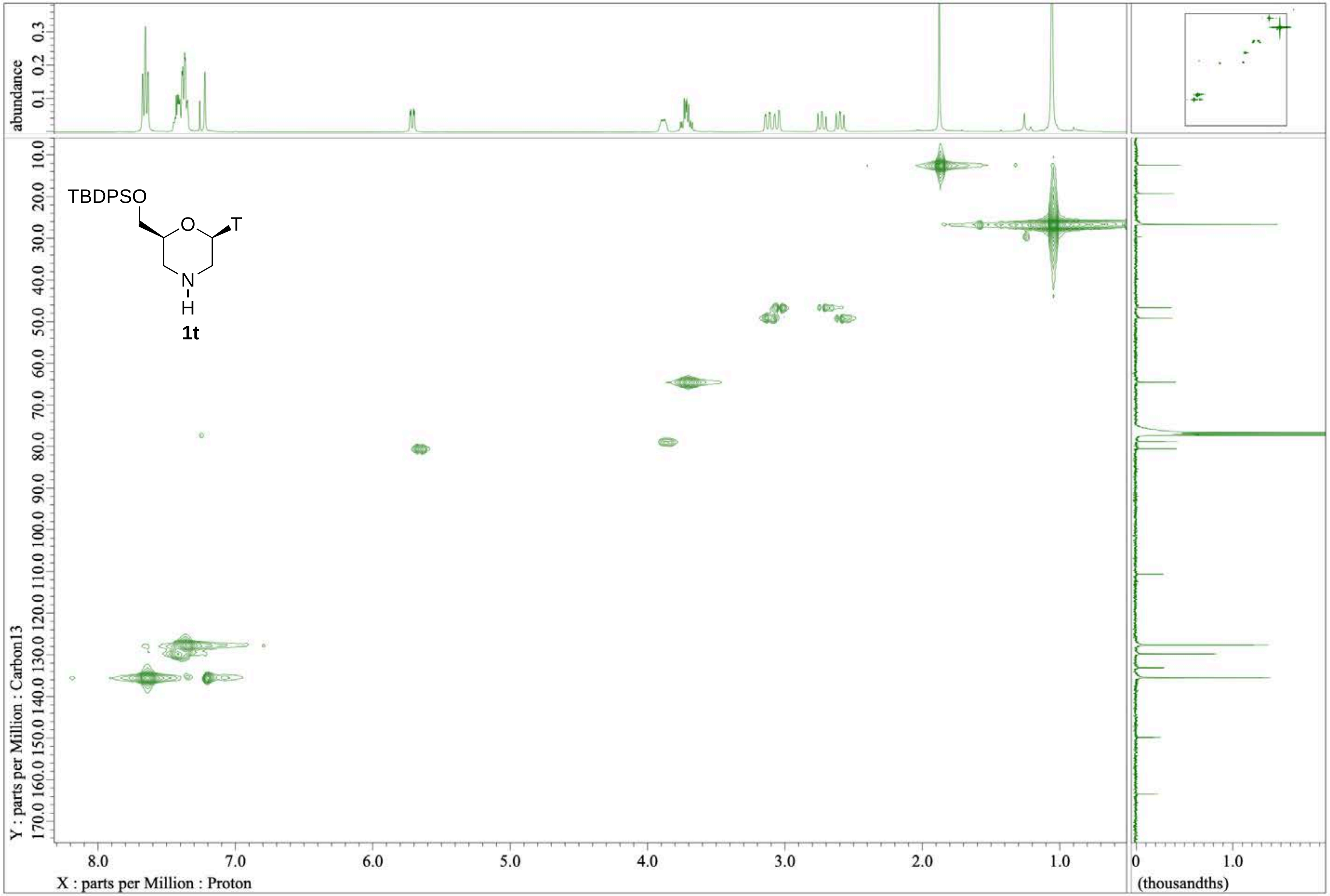

abundance
TBDPSO
O
T
N
H
1t
Y : parts per Million : Carbon13
X : parts per Million : Proton
(thousandths)

HMBC ($CDCl_3$)

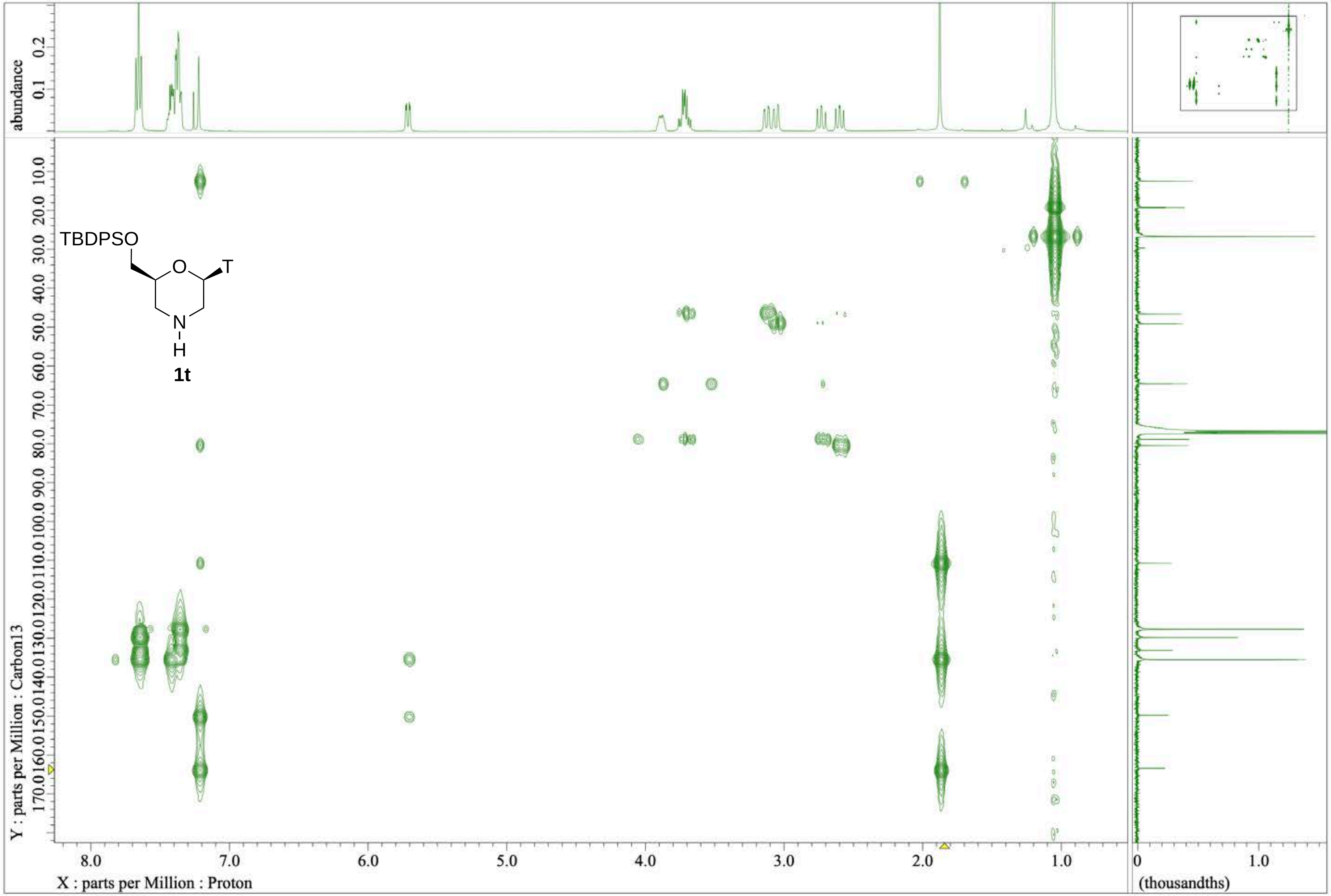

TBDPSO
O
T
N
H
1t
abundance
0.1
0.2
Y : parts per Million : Carbon13
X : parts per Million : Proton
8.0
7.0
6.0
5.0
4.0
3.0
2.0
1.0
(thousandths)

Mass spectra

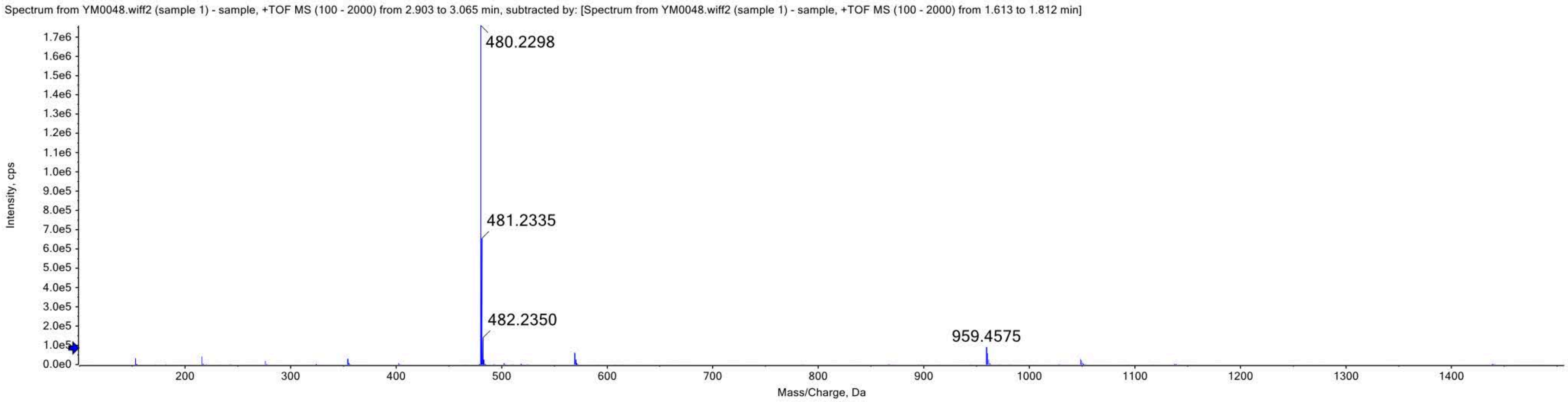

ESI-MS *m/z* calcd for C26H34N3O4Si $[M+H]^+$, 480.2313; found 480.2298.

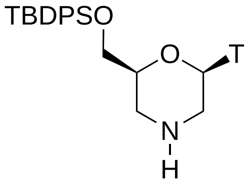

1t

^{1}H-NMR (400 MHz, $CDCl_3$)

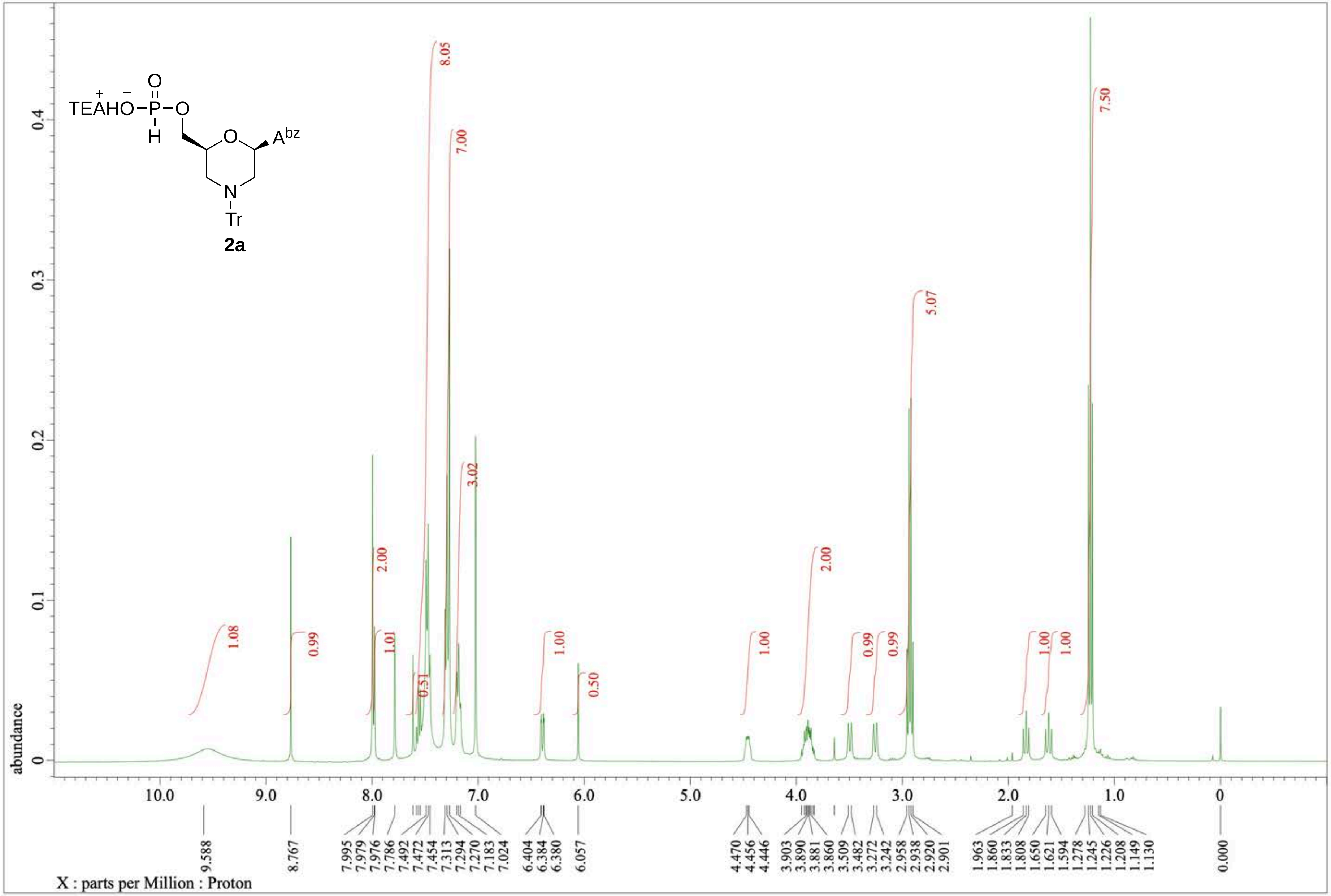

$^{13}C\{H\}$-NMR (101 MHz, $CDCl_3$)

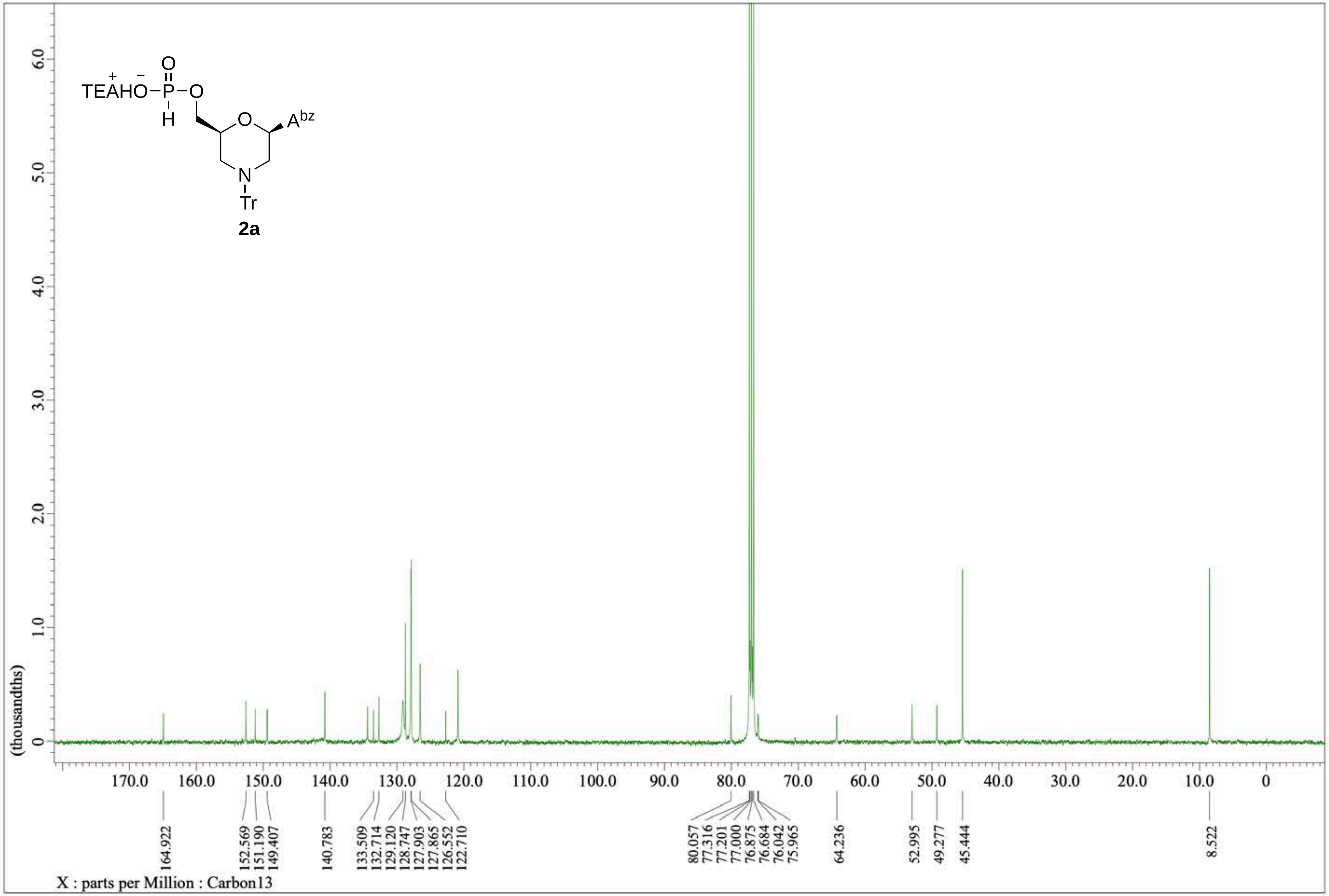

HMQC ($CDCl_3$)

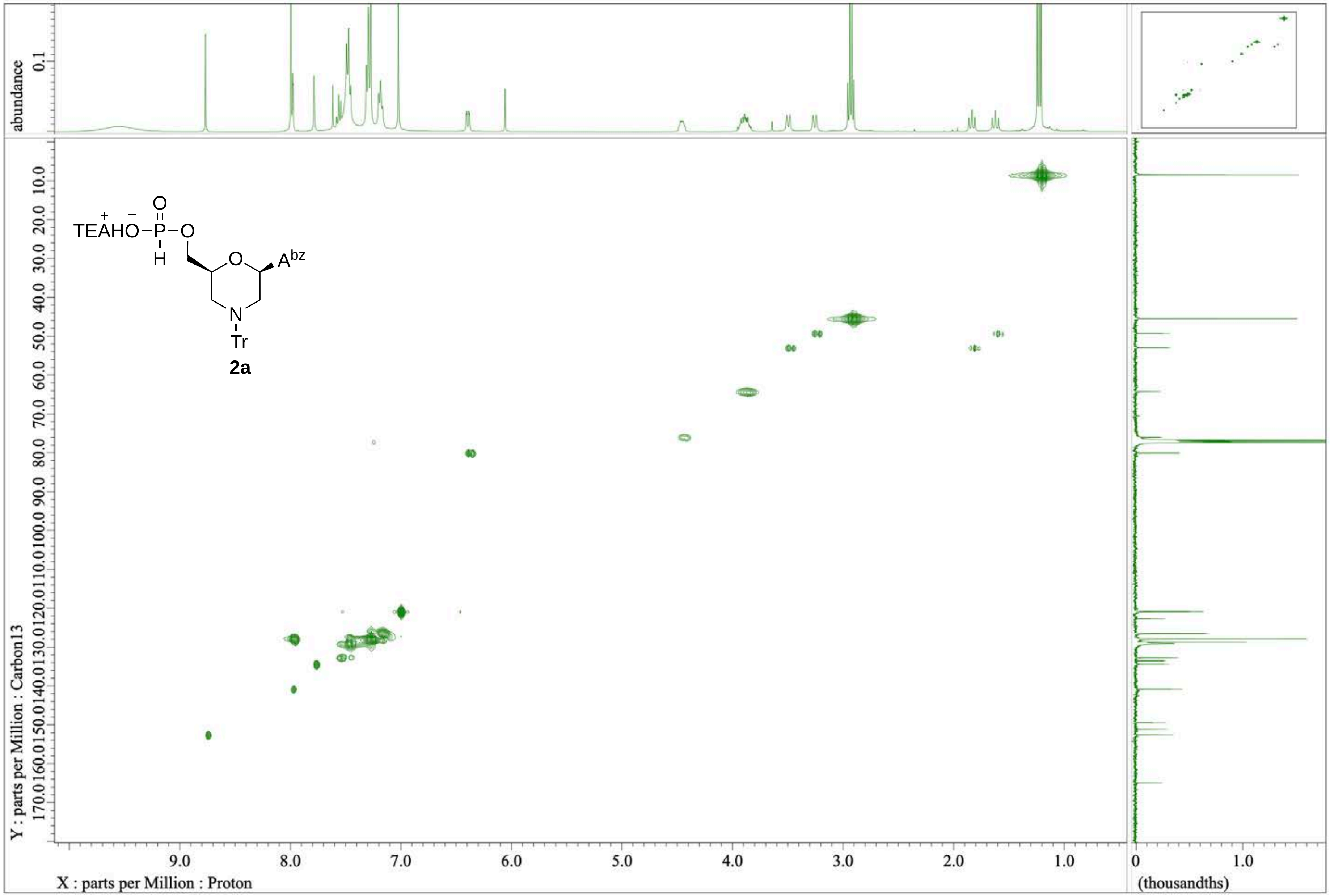
abundance
0.1
TEAHO−P−O
O
H
N
Tr
A^bz
2a
Y : parts per Million : Carbon13
10.0
20.0
30.0
40.0
50.0
60.0
70.0
80.0
90.0
100.0
110.0
120.0
130.0
140.0
150.0
160.0
170.0
9.0
8.0
7.0
6.0
5.0
4.0
3.0
2.0
1.0
X : parts per Million : Proton
0
1.0
(thousandths)

HMBC ($CDCl_3$)

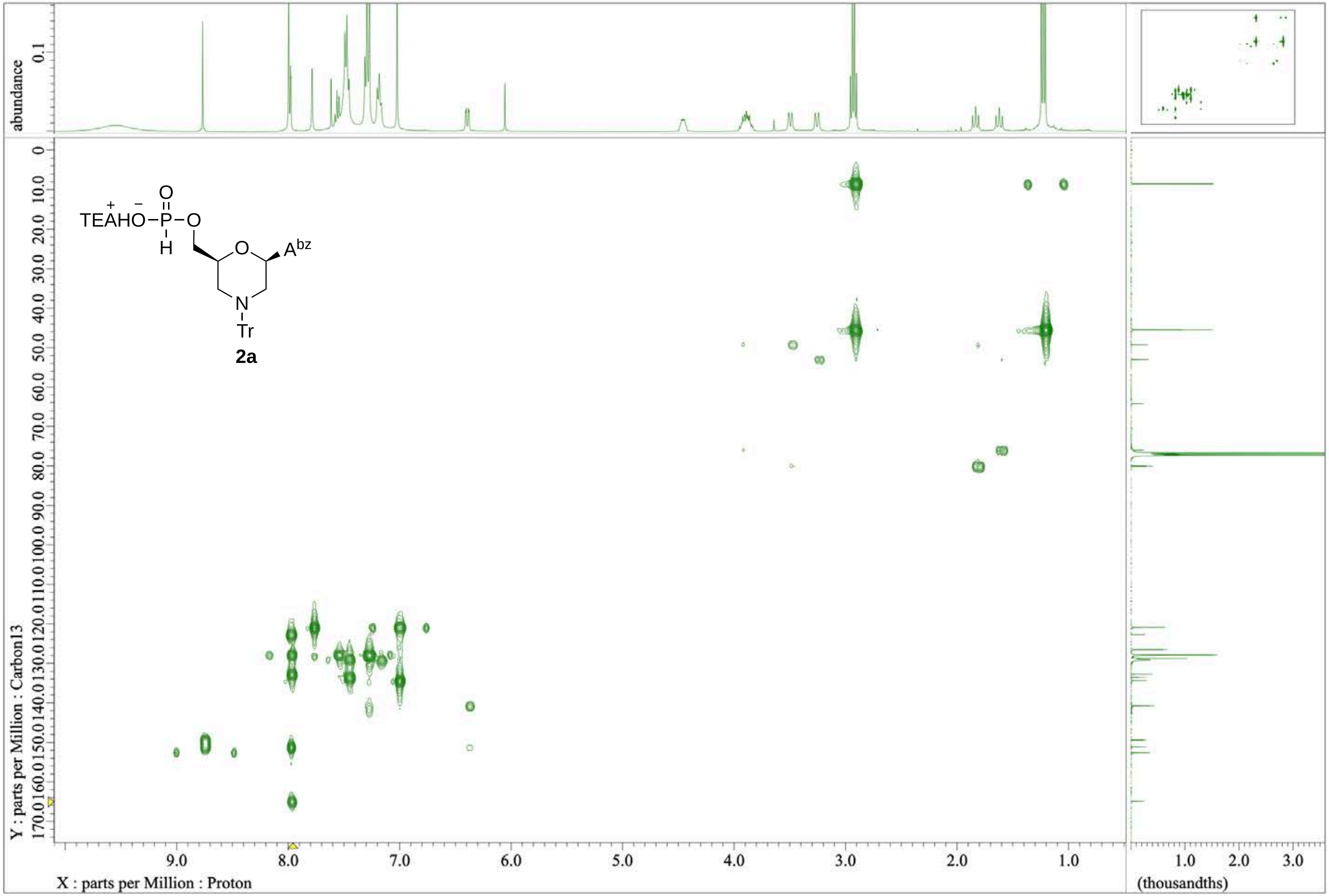
abundance
0.1
TEAHO−P−O
O
H
O
A^bz
N
Tr
2a
Y : parts per Million : Carbon13
X : parts per Million : Proton
9.0
8.0
7.0
6.0
5.0
4.0
3.0
2.0
1.0
(thousandths)

$^{31}P\{^{1}H\}$ NMR (162 MHz, $CDCl_3$)

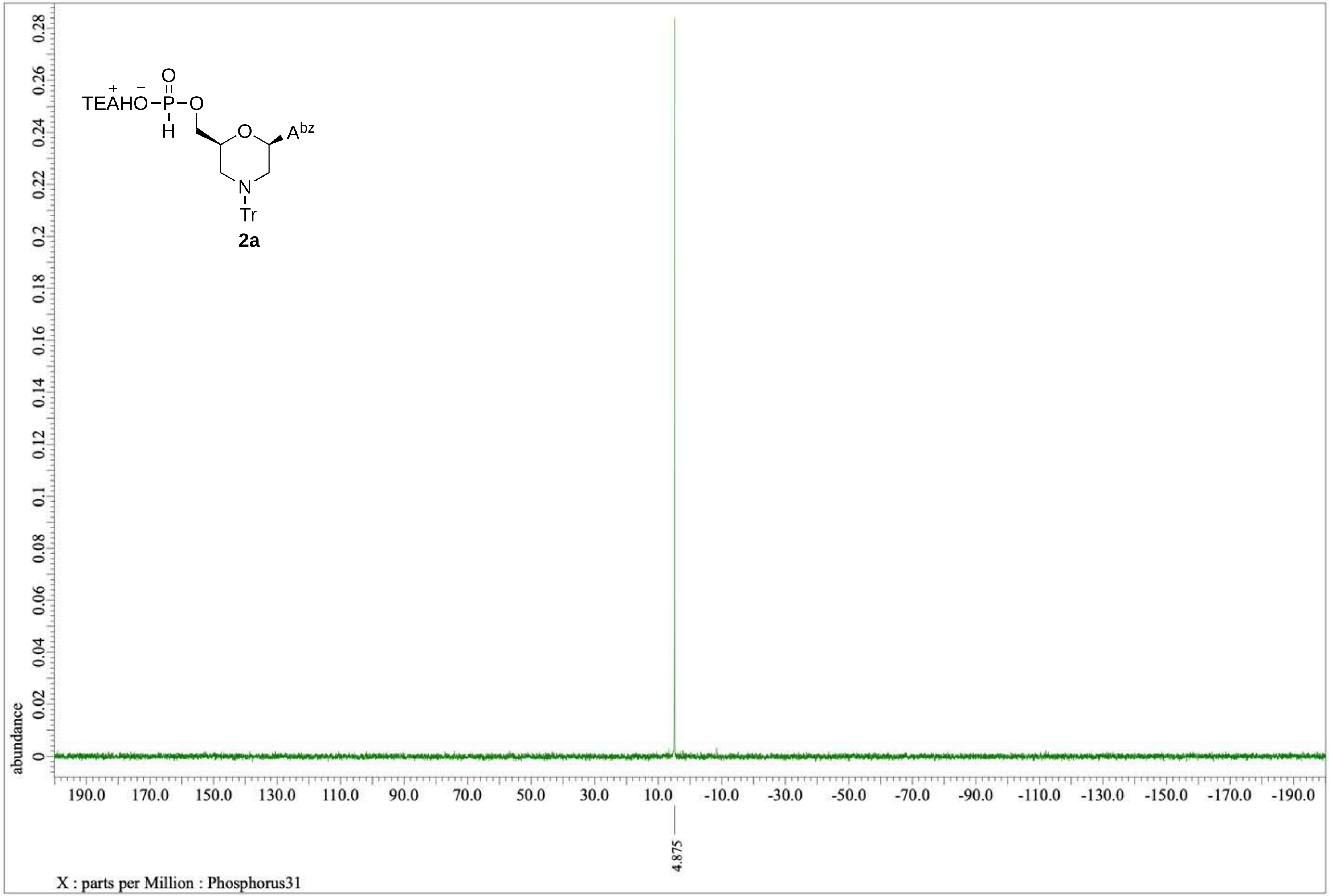

Mass spectra

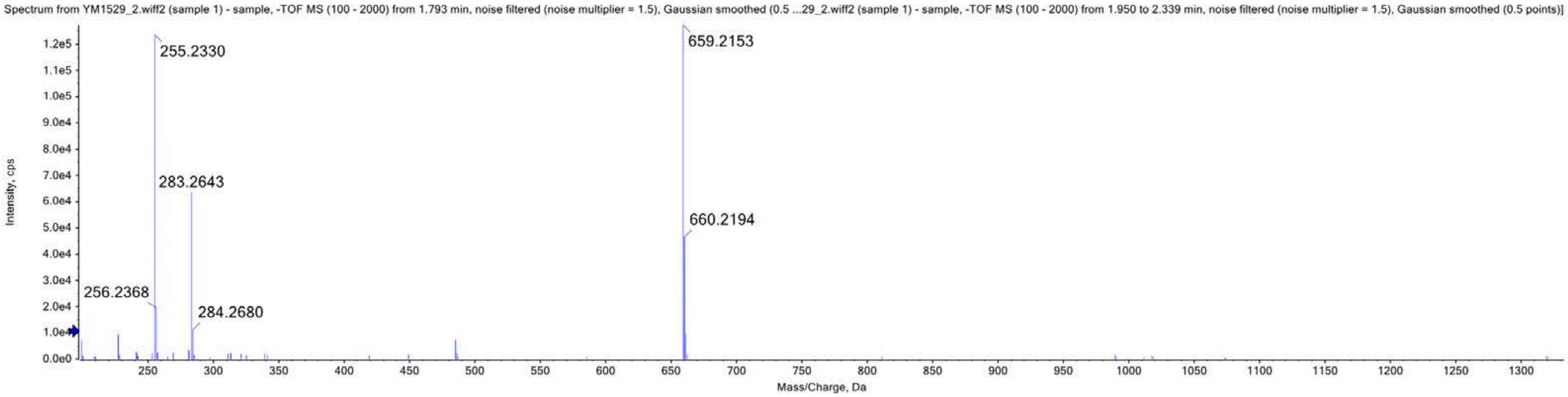

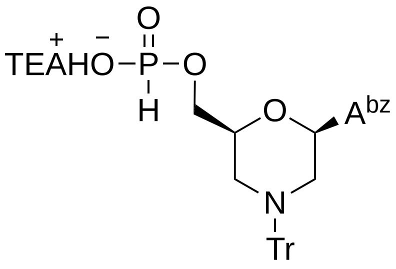

2a

ESI-MS *m/z* calcd for $C_{36}H_{32}N_6O_5P$ [M-H-TEA]$^-$, 659.2177; found 659.2153.

^{1}H-NMR (400 MHz, $CDCl_3$)

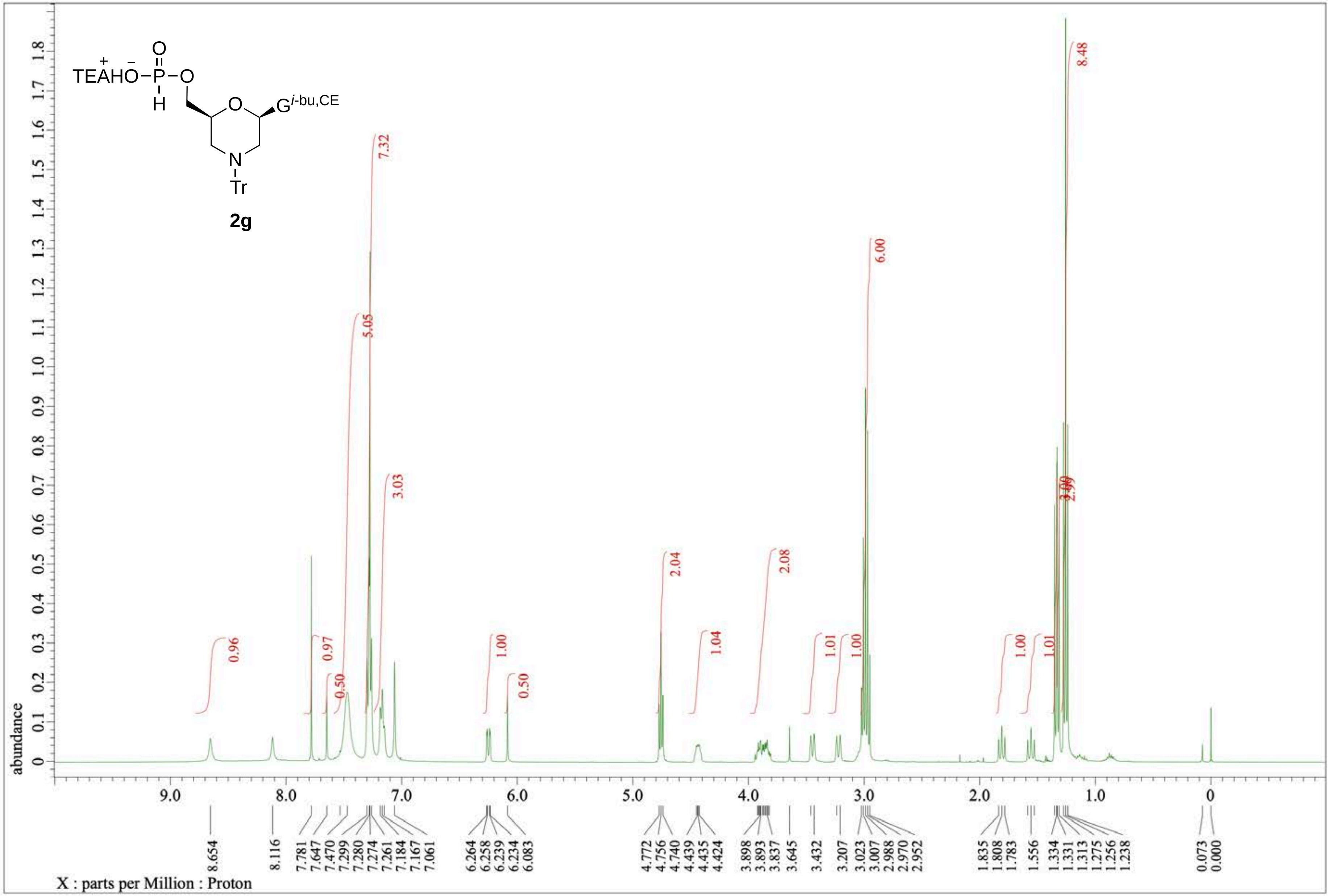

$^{13}C\{H\}$-NMR (101 MHz, $CDCl_3$)

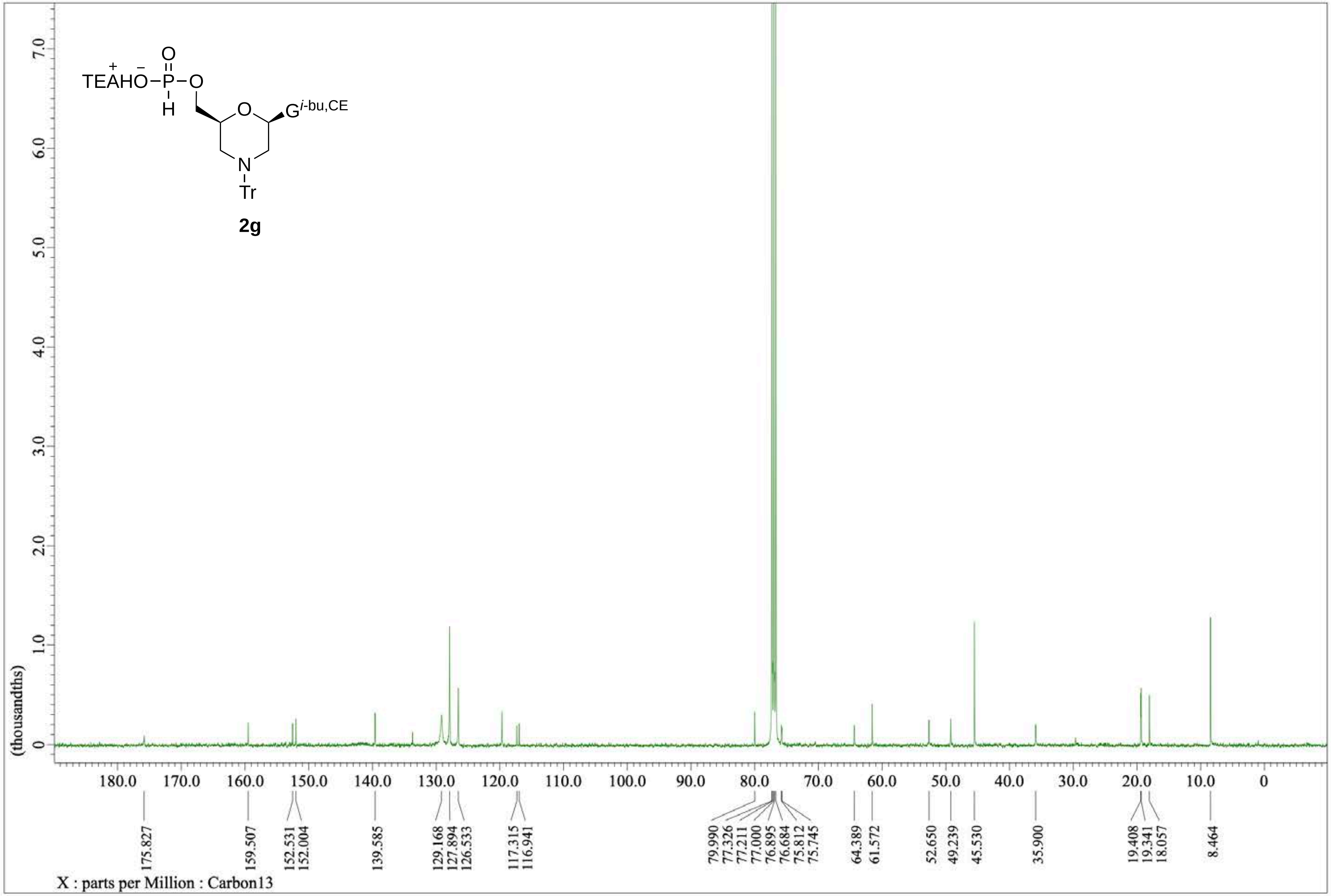

HMQC ($CDCl_3$)

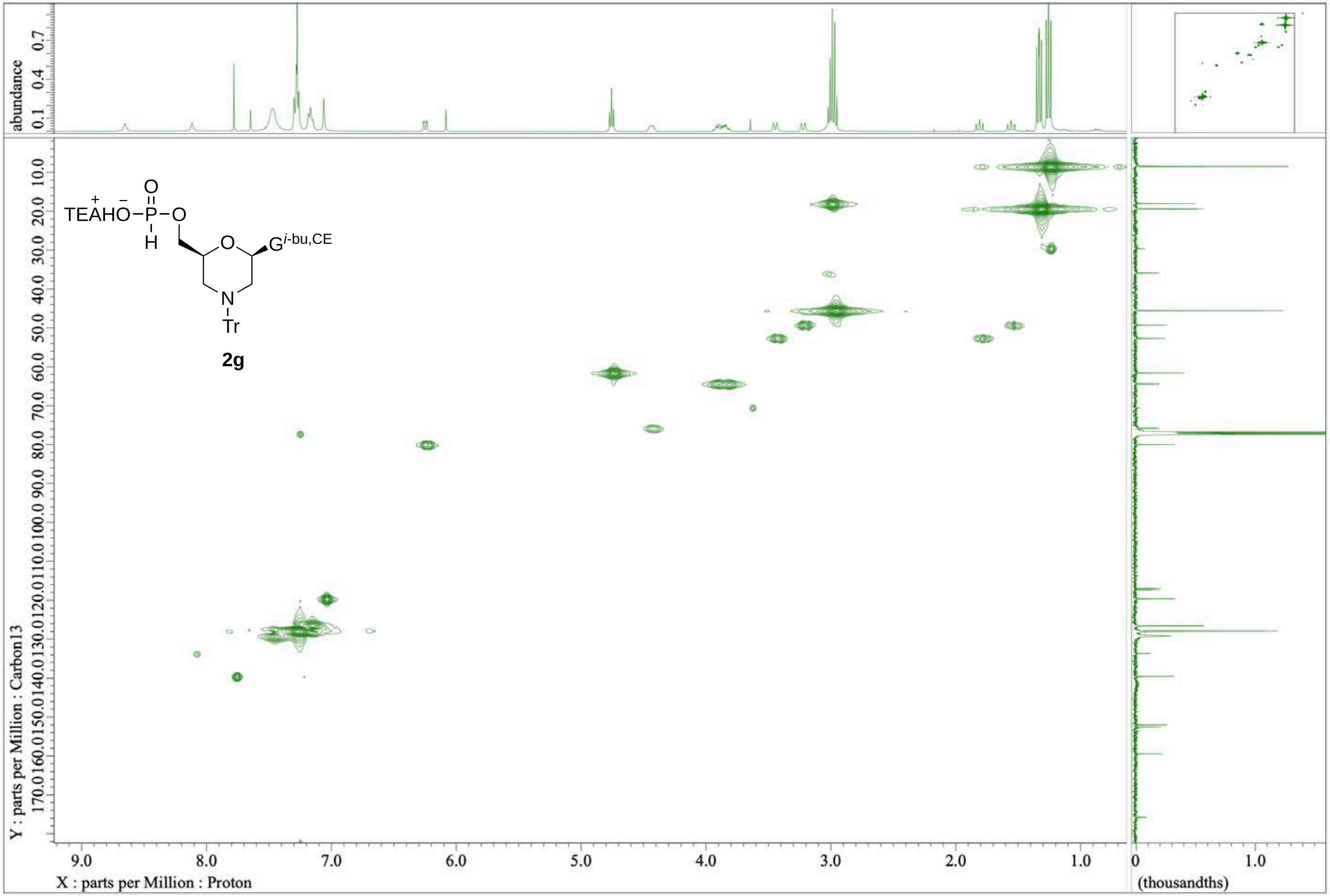

abundance
0.1
0.4
0.7
TEAH O−P−O
O
H
O
G^{i-bu,CE}
N
Tr
2g
Y : parts per Million : Carbon13
10.0
20.0
30.0
40.0
50.0
60.0
70.0
80.0
90.0
100.0
110.0
120.0
130.0
140.0
150.0
160.0
170.0
9.0
8.0
7.0
6.0
5.0
4.0
3.0
2.0
1.0
X : parts per Million : Proton
0
1.0
(thousandths)

HMBC ($CDCl_3$)

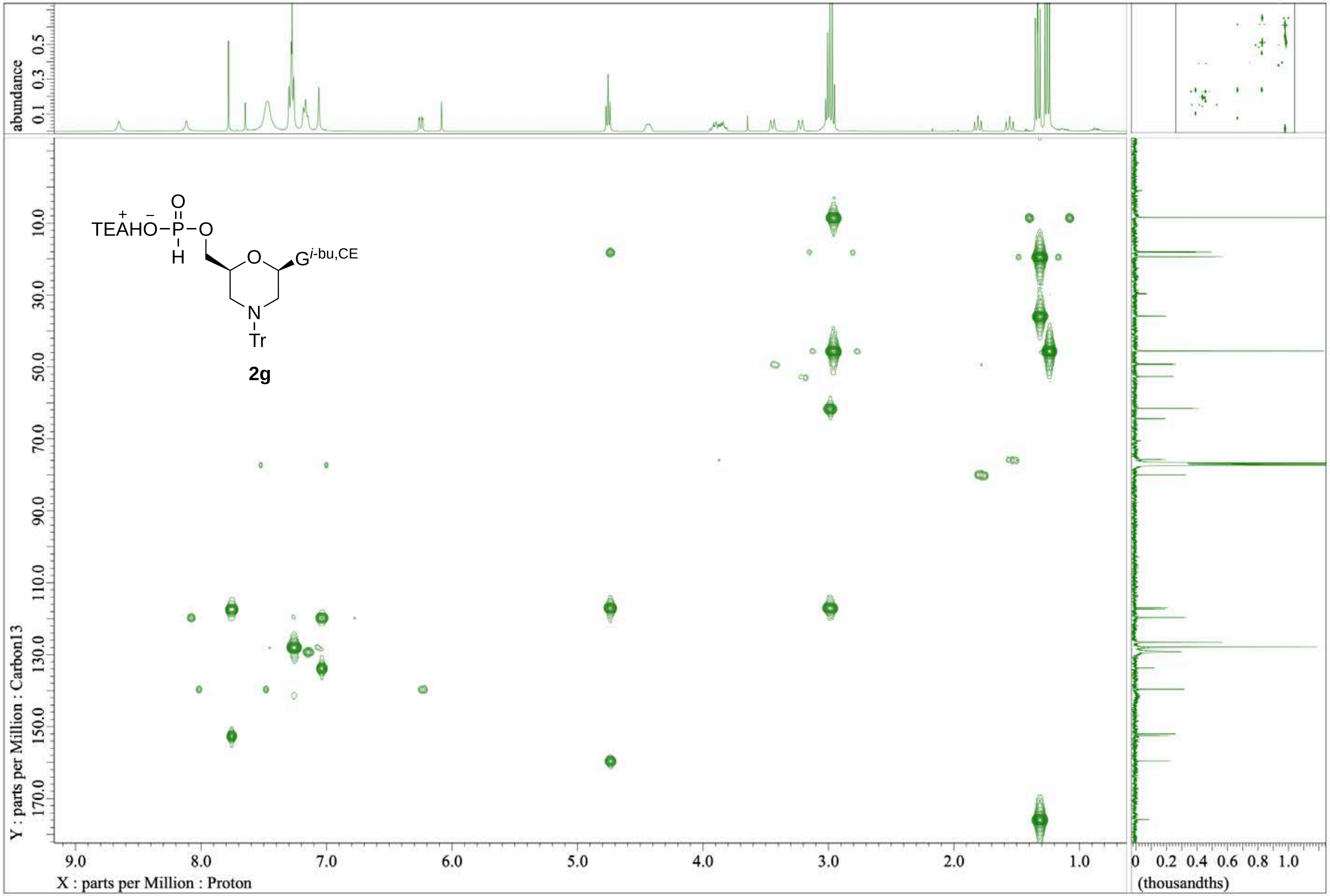
TEAHO
O
P
O
H
O
N
Tr
G i-bu,CE
2g
abundance
0.1 0.3 0.5
Y : parts per Million : Carbon13
10.0 30.0 50.0 70.0 90.0 110.0 130.0 150.0 170.0
9.0 8.0 7.0 6.0 5.0 4.0 3.0 2.0 1.0
X : parts per Million : Proton
0 0.2 0.4 0.6 0.8 1.0
(thousandths)

$^{31}P\{^{1}H\}$ NMR (162 MHz, $CDCl_3$)

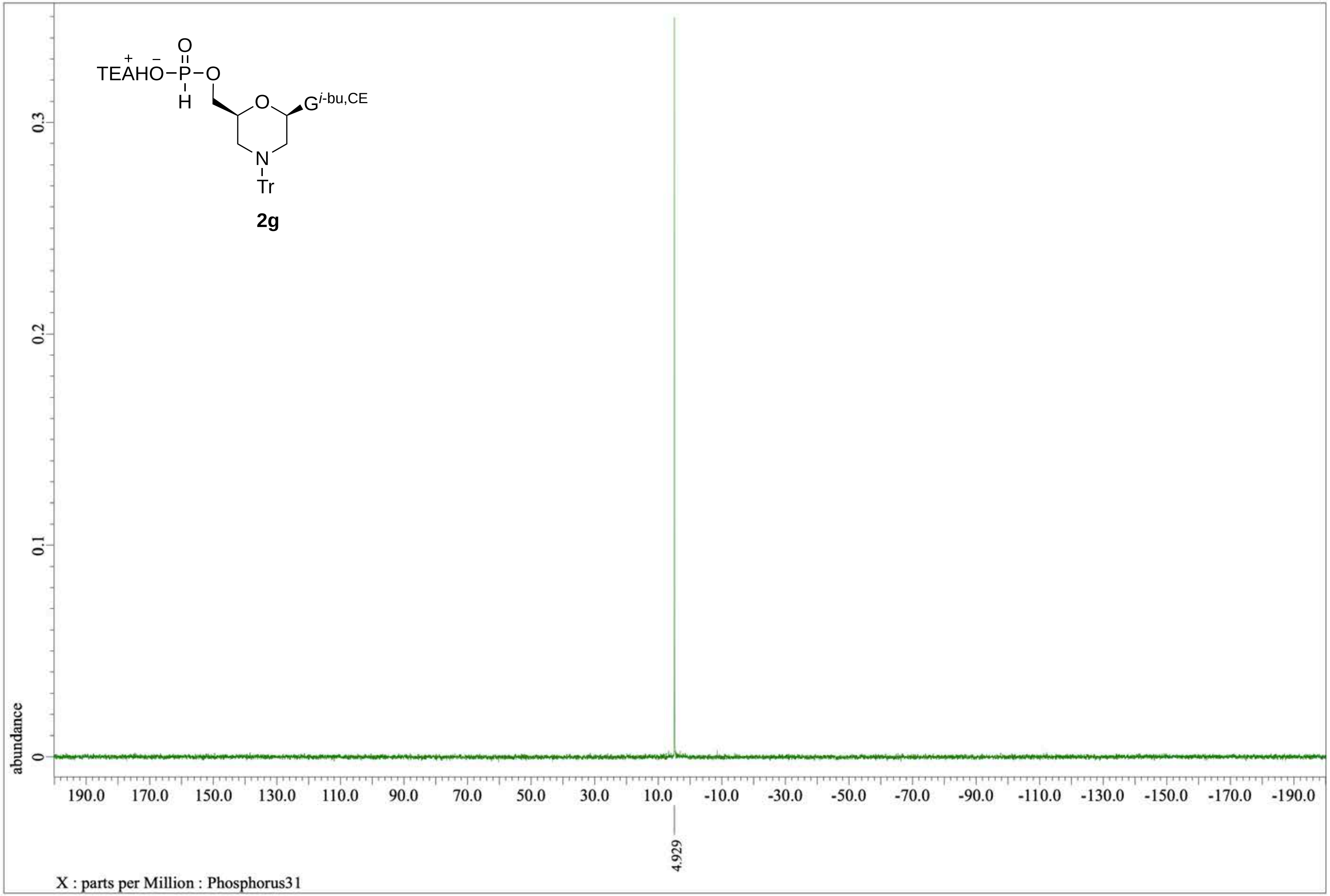

Mass spectra

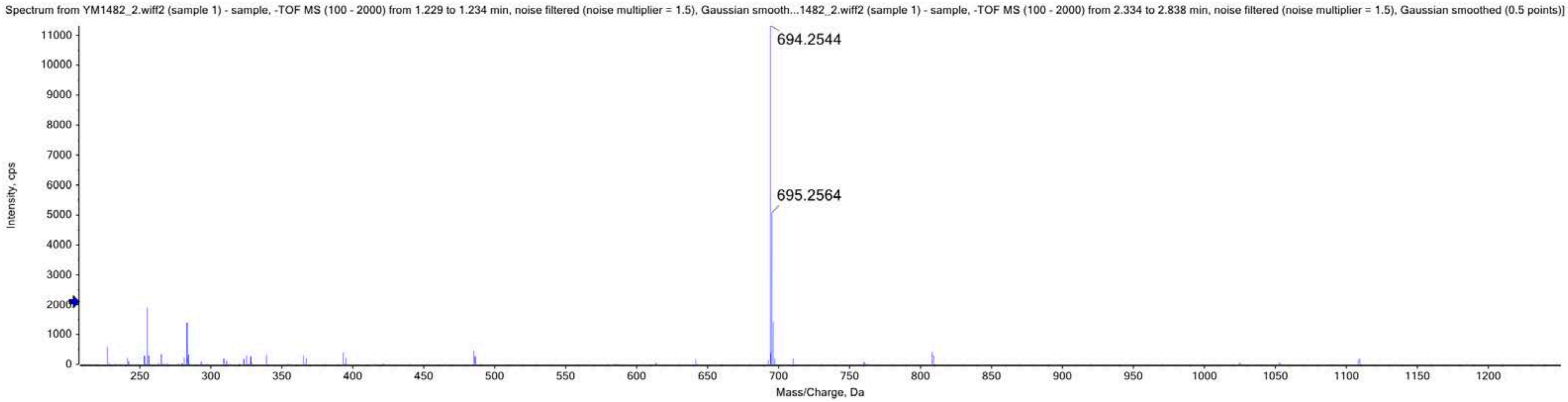

ESI-MS *m/z* calcd for $C_{36}H_{37}N_7O_6P$ [M-H-TEA]$^-$, 694.2548; found 694.2544.

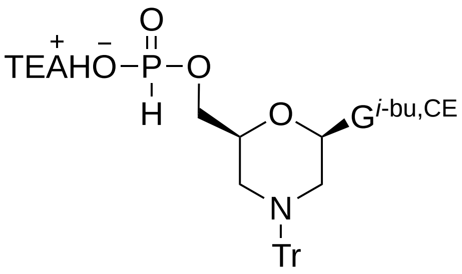

2g

^{1}H-NMR (400 MHz, $CDCl_3$)

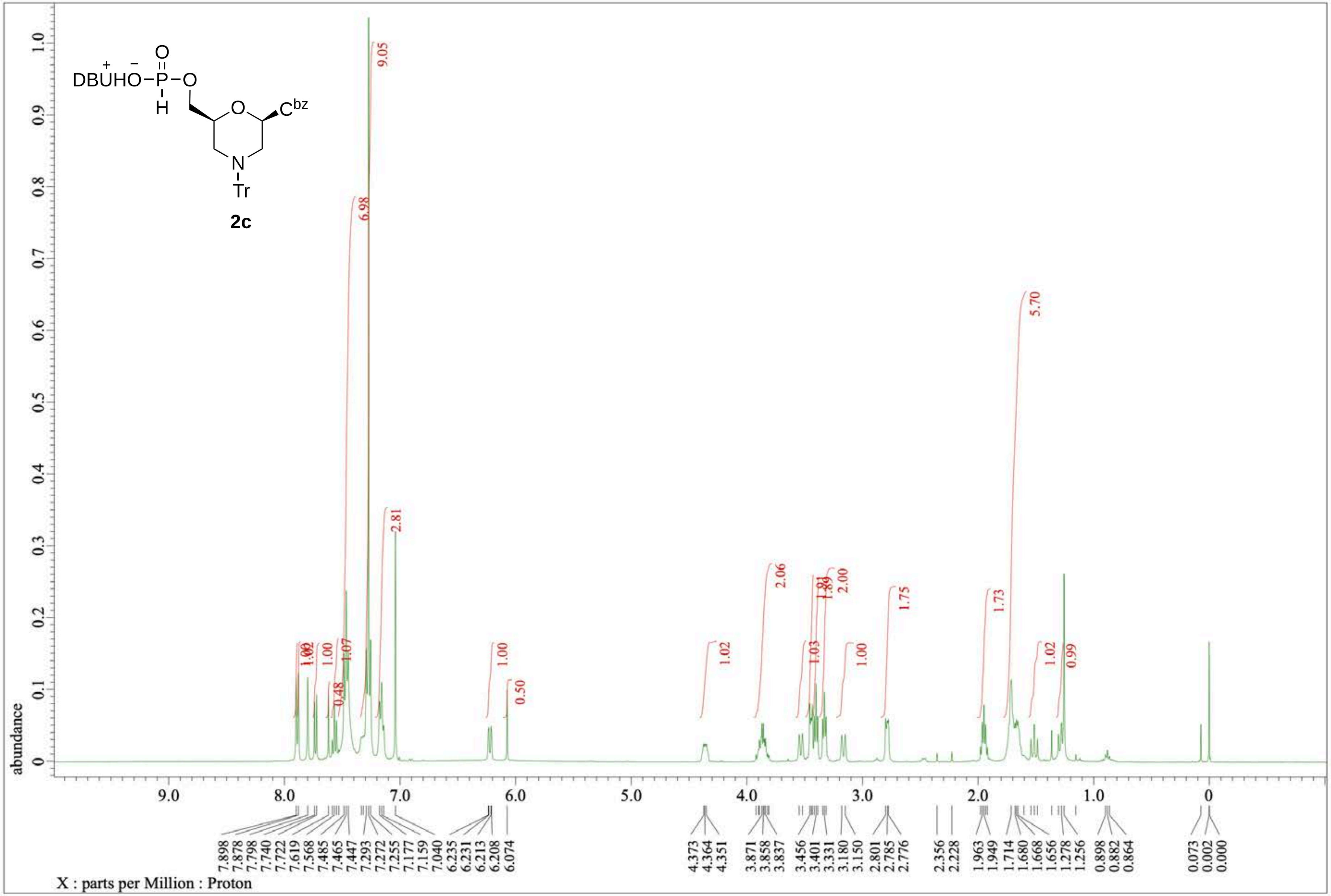

$^{13}C\{H\}$-NMR (101 MHz, $CDCl_3$)

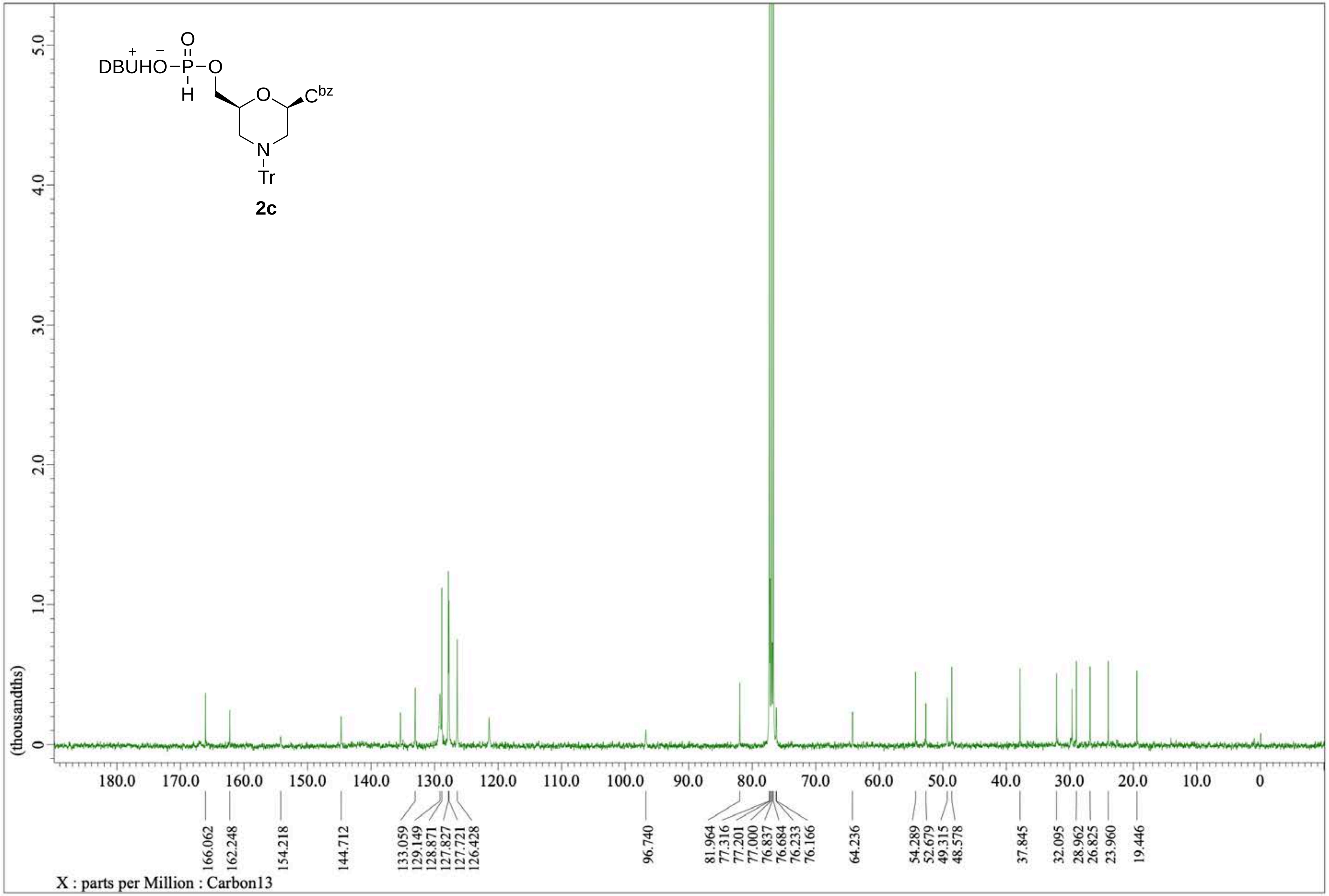

HMQC ($CDCl_3$)

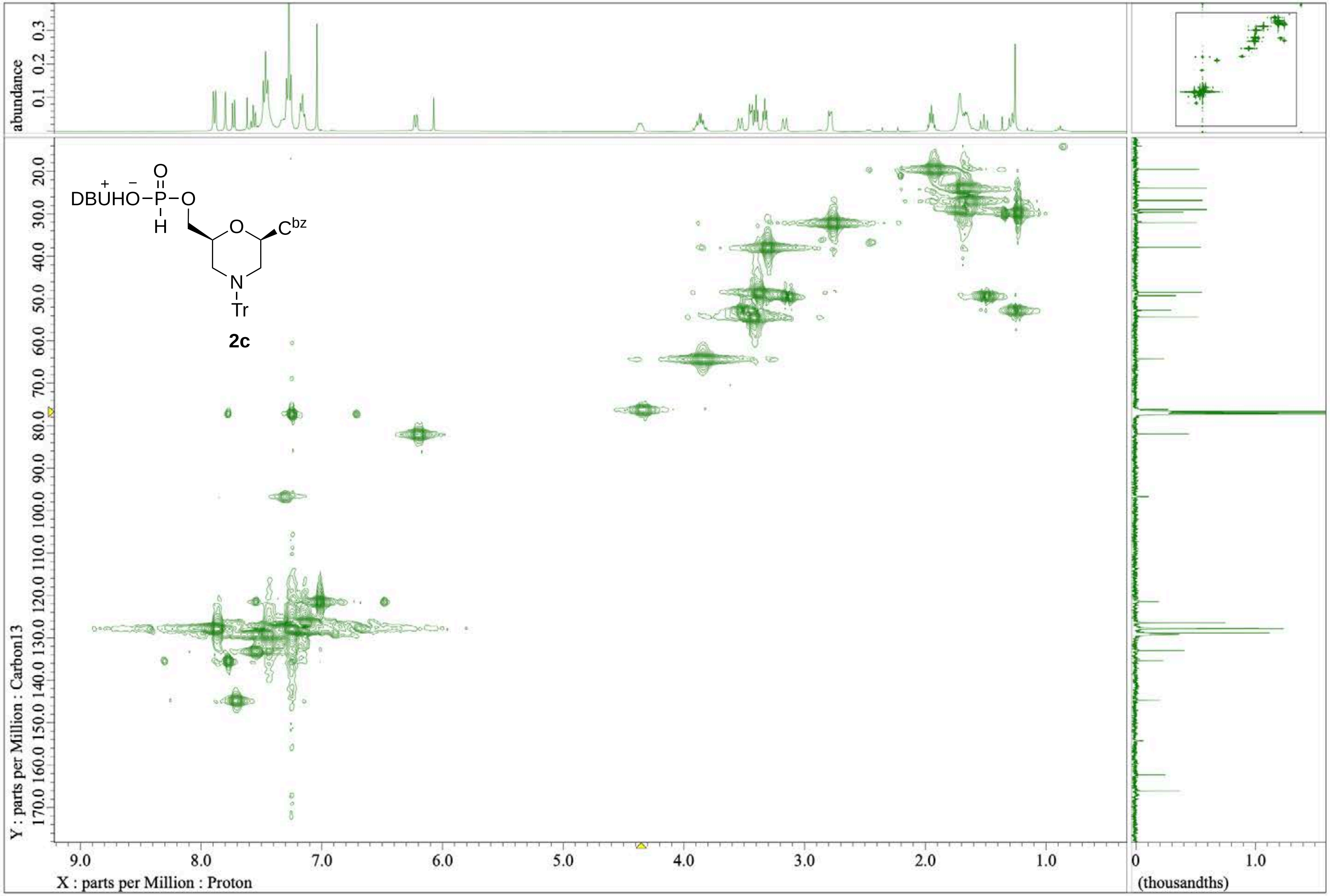

abundance
0.1
0.2
0.3
DBUHO–P–O
O
H
O
C^bz
N
Tr
2c
Y : parts per Million : Carbon13
X : parts per Million : Proton
9.0
8.0
7.0
6.0
5.0
4.0
3.0
2.0
1.0
20.0
30.0
40.0
50.0
60.0
70.0
80.0
90.0
100.0
110.0
120.0
130.0
140.0
150.0
160.0
170.0
0
1.0
(thousandths)

HMBC ($CDCl_3$)

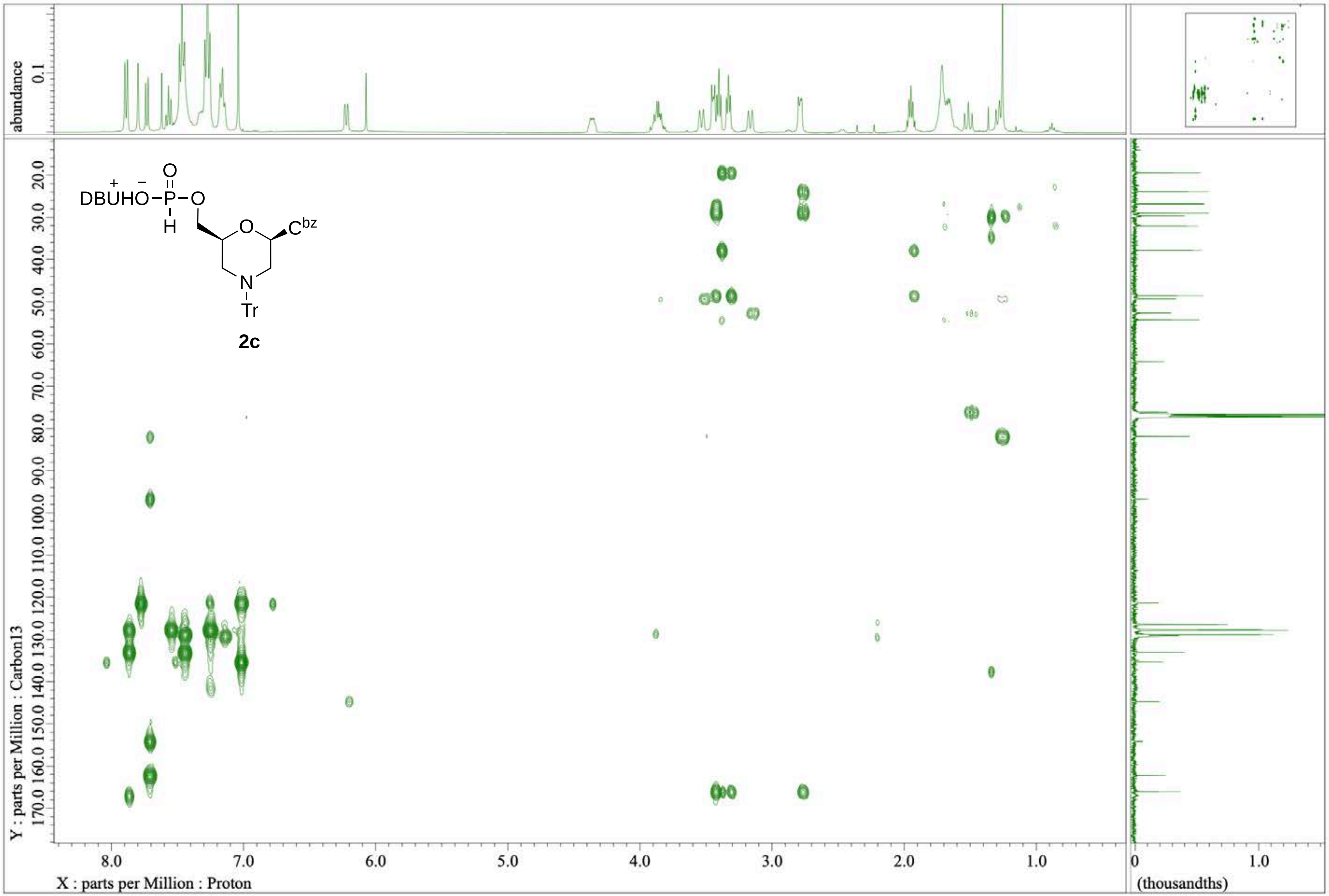

abundance
0.1
DBUHO–P–O
O
H
O
C^bz
N
Tr
2c
Y : parts per Million : Carbon13
X : parts per Million : Proton
(thousandths)

$^{31}P\{^{1}H\}$ NMR (162 MHz, $CDCl_3$)

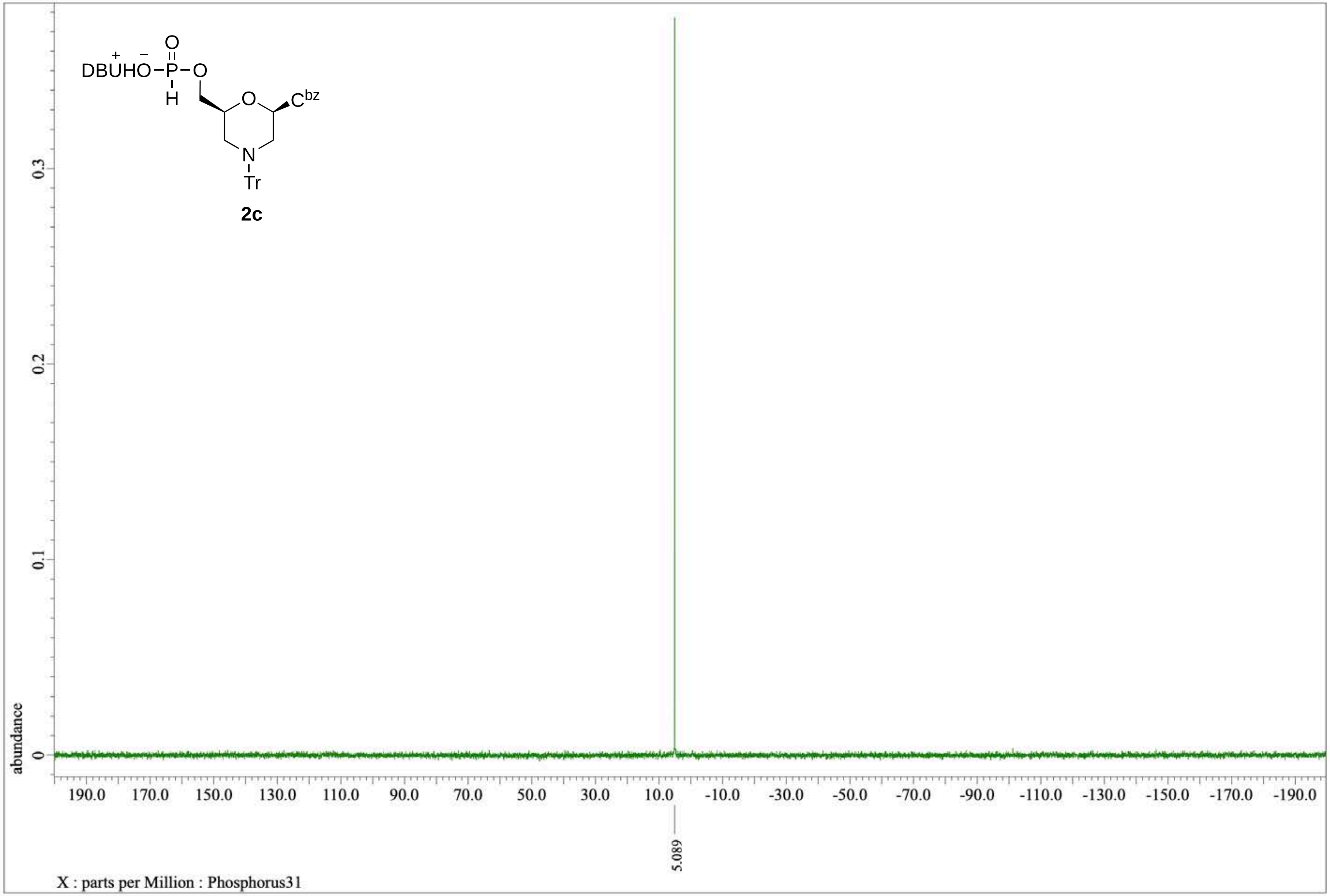

Mass spectra

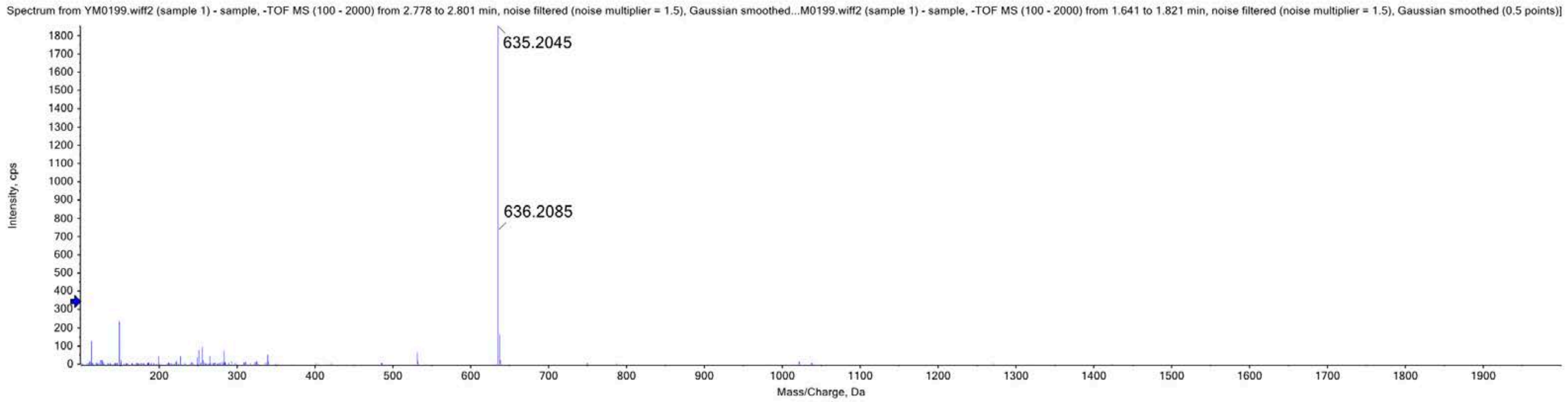

ESI-MS *m/z* calcd for $C_{35}H_{32}N_4O_6P$ [M-H-DBU]$^-$, 635.2065; found 635.2045.

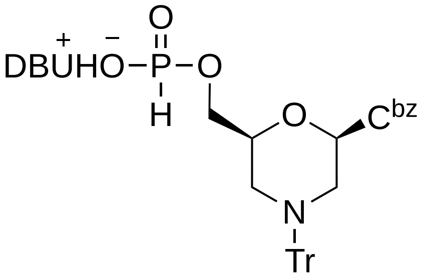

2c

^{1}H-NMR (400 MHz, $CDCl_3$)

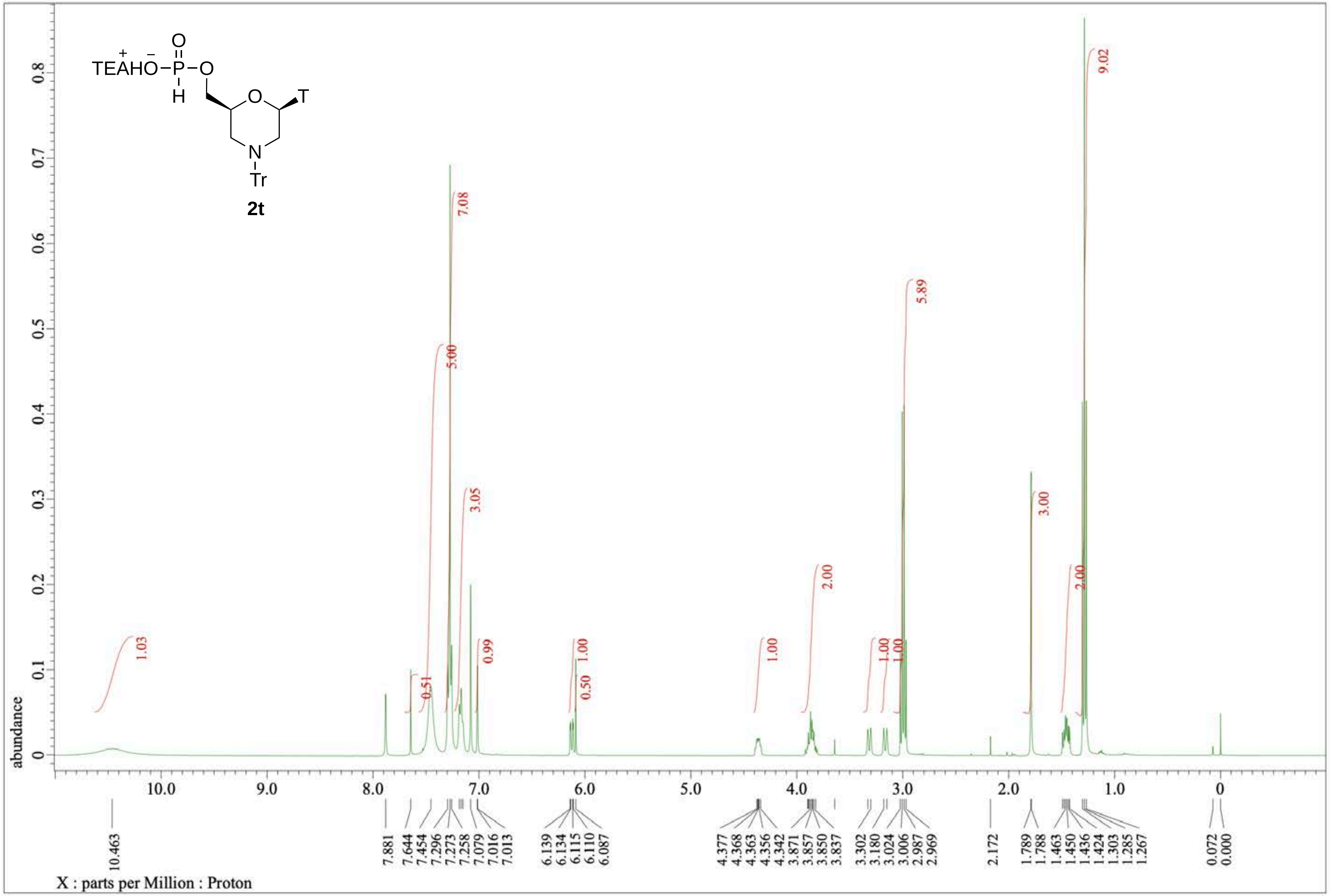

$^{13}C\{H\}$-NMR (101 MHz, $CDCl_3$)

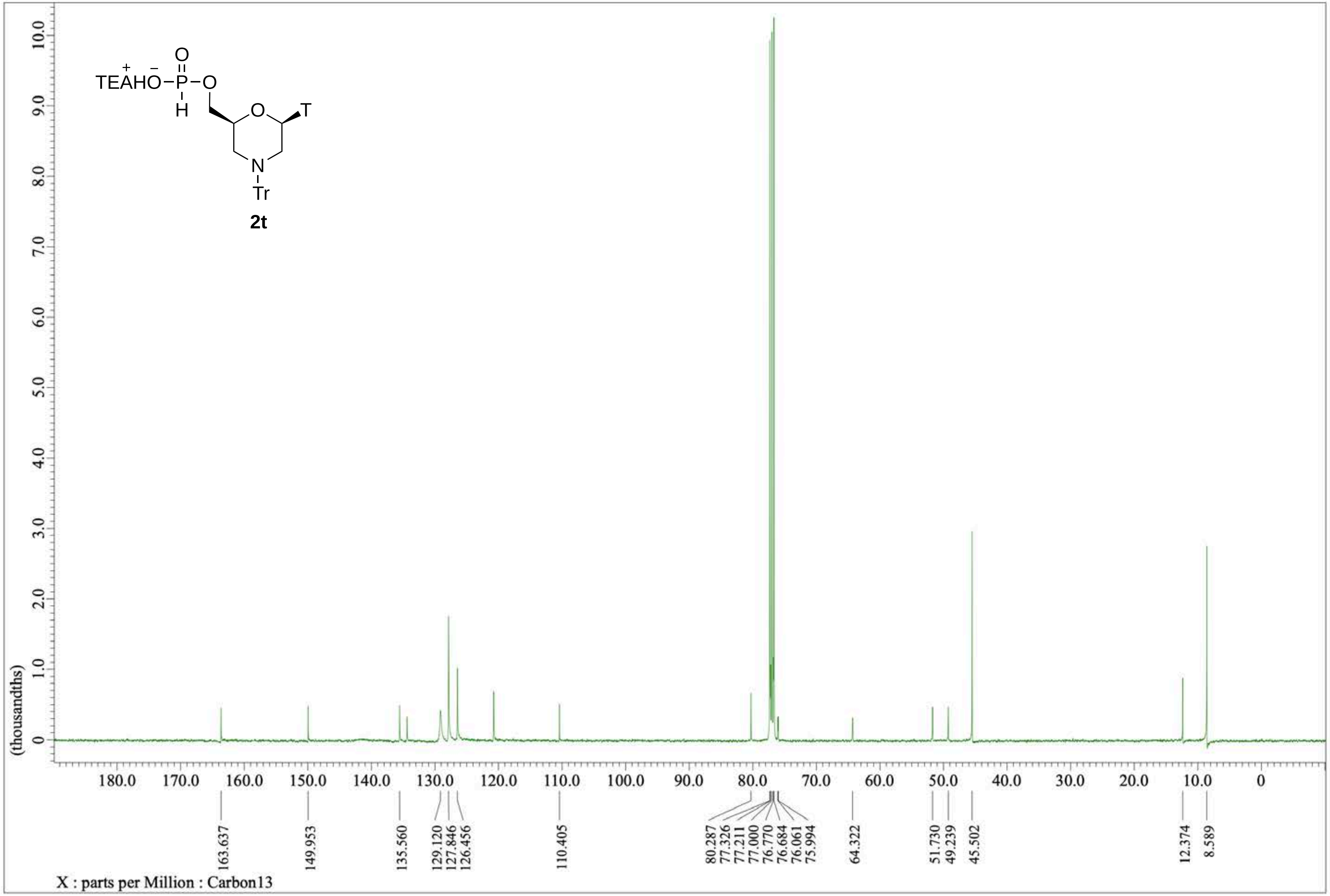

HMQC ($CDCl_3$)

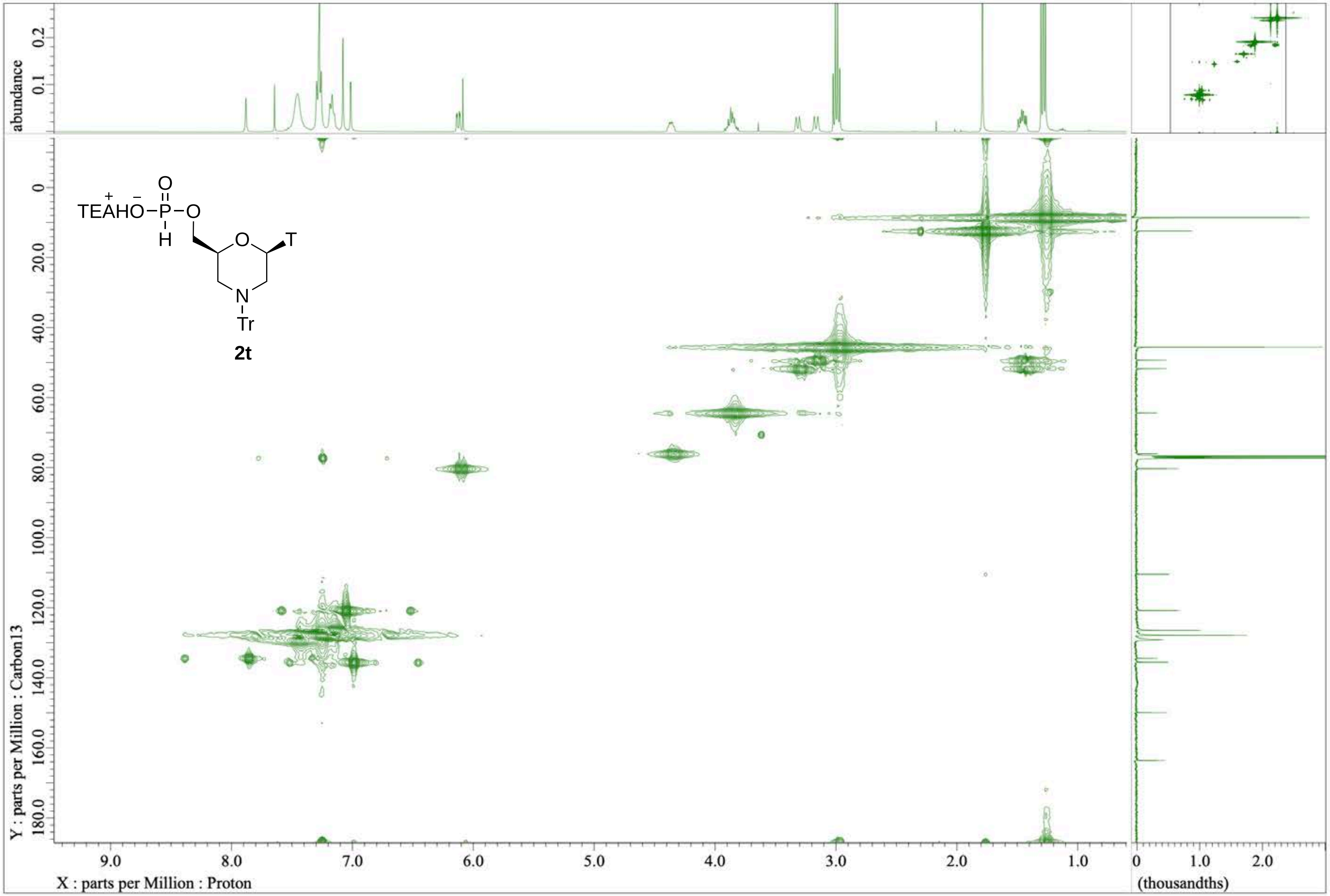

abundance
0.1
0.2
Y : parts per Million : Carbon13
0
20.0
40.0
60.0
80.0
100.0
120.0
140.0
160.0
180.0
X : parts per Million : Proton
9.0
8.0
7.0
6.0
5.0
4.0
3.0
2.0
1.0
(thousandths)
TEAHO–P–O
O
H
O
T
N
Tr
2t

HMBC ($CDCl_3$)

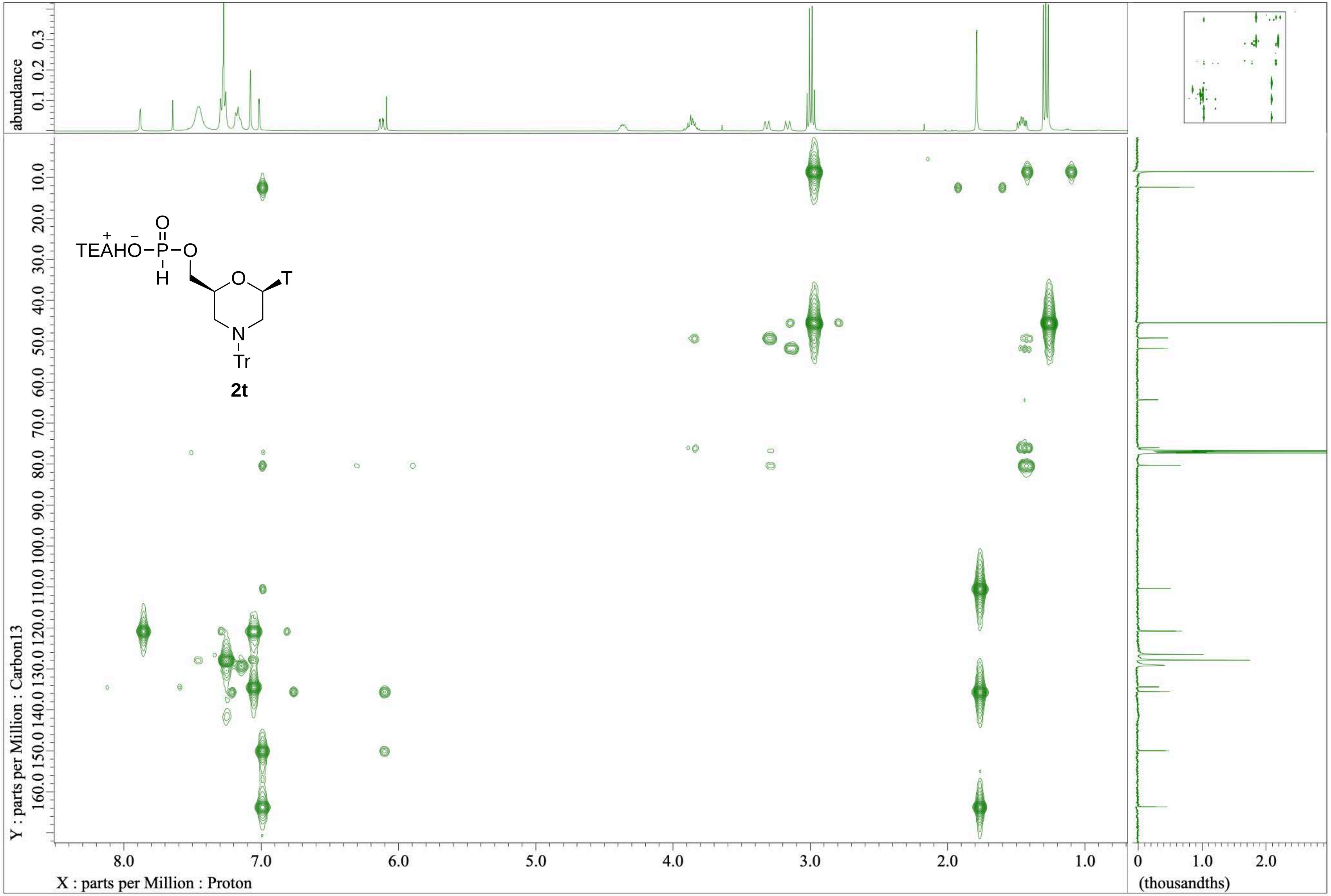
abundance
0.1
0.2
0.3
TEAHO–P–O
O
H
O
T
N
Tr
2t
Y : parts per Million : Carbon13
X : parts per Million : Proton
(thousandths)

$^{31}P\{^{1}H\}$ NMR (162 MHz, $CDCl_3$)

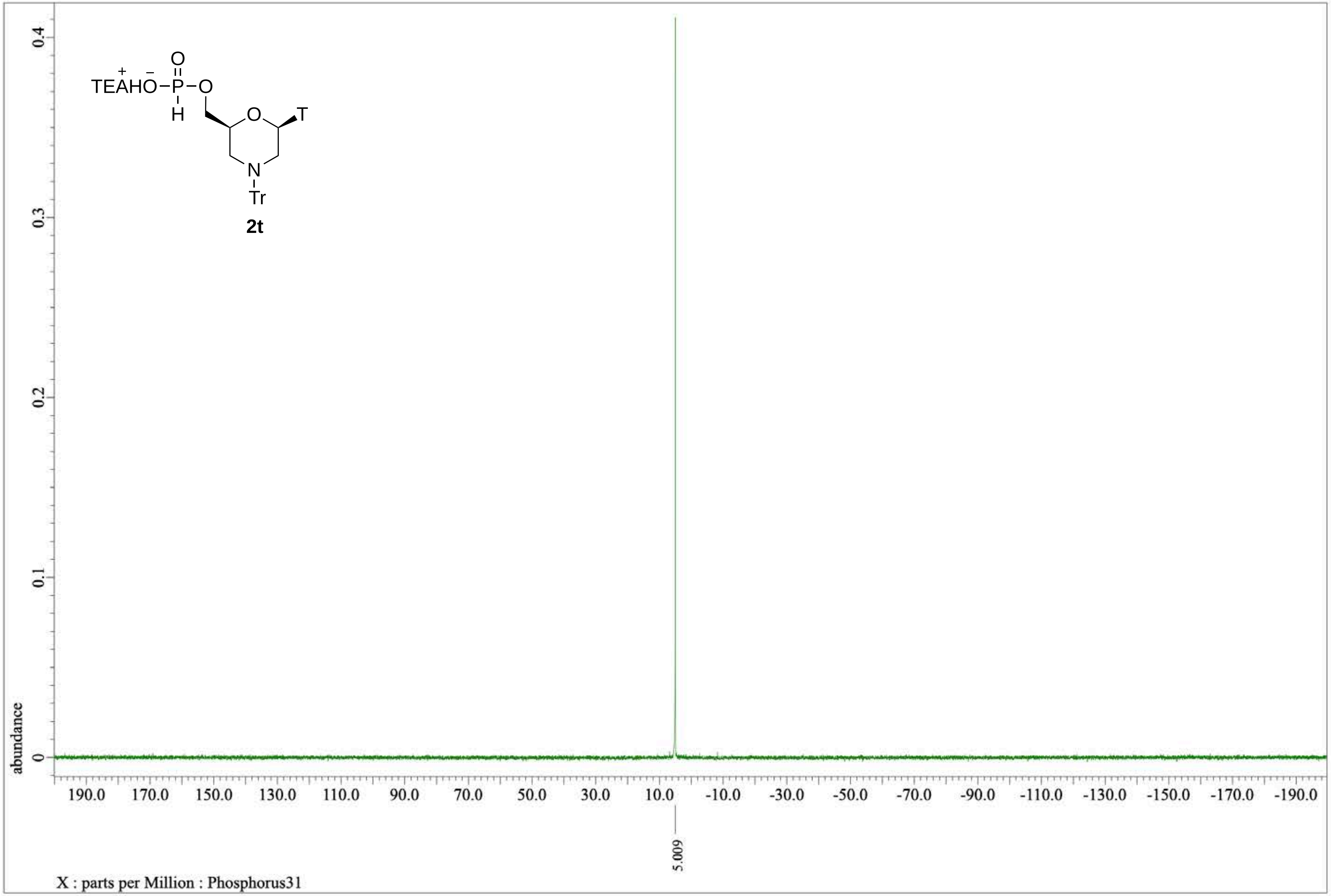

Mass spectra

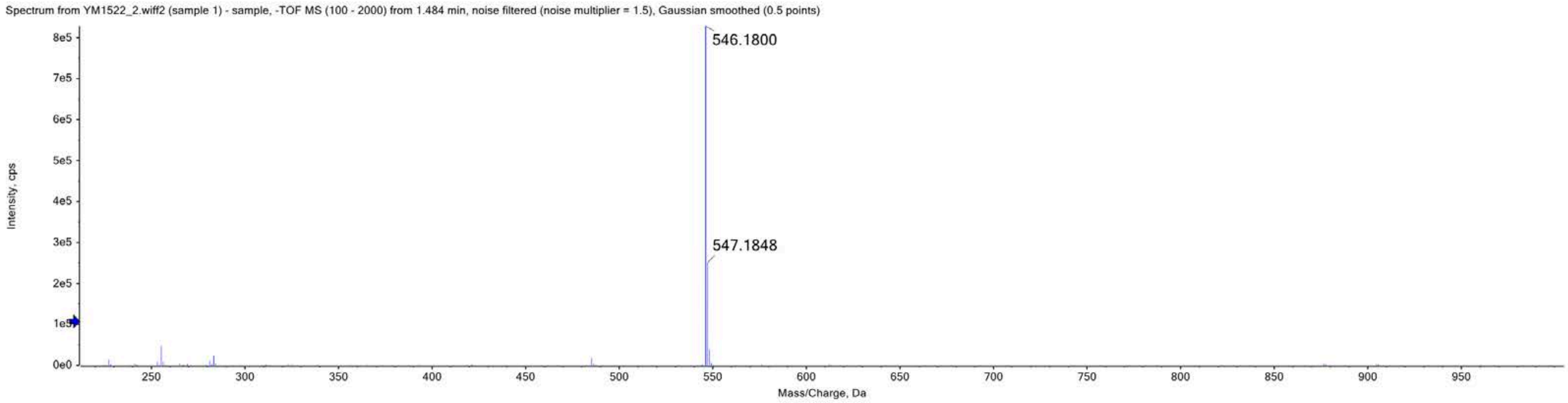

ESI-MS *m/z* calcd for $C_{29}H_{29}N_3O_6P$ [M-H-TEA]$^-$, 546.1799; found 546.1800.

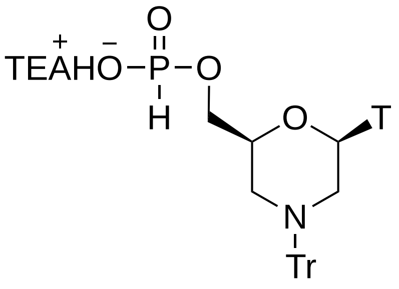

2t

^{1}H-NMR (400 MHz, $CDCl_3$)

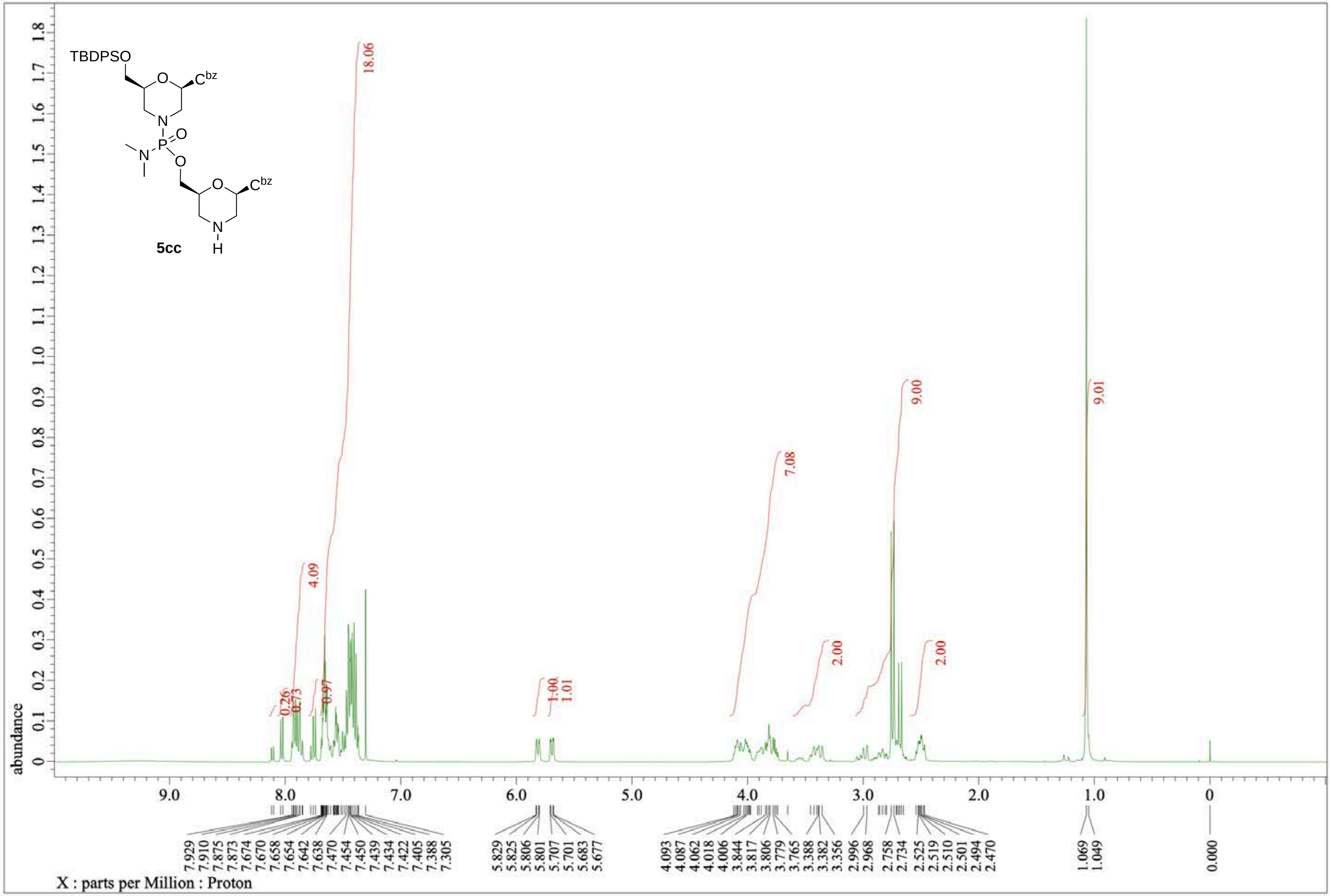

$^{13}C\{H\}$-NMR (101 MHz, $CDCl_3$)

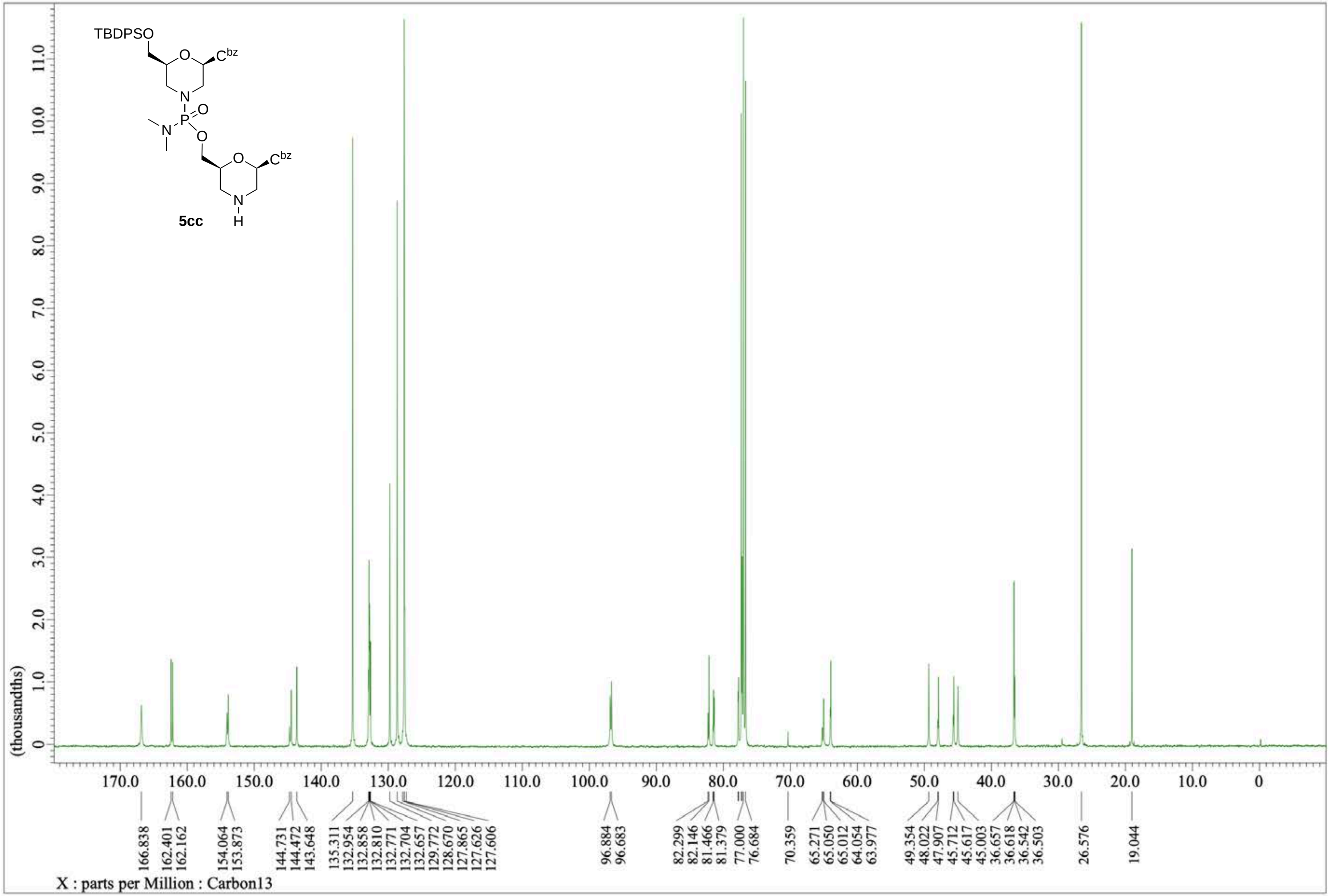

HMQC ($CDCl_3$)

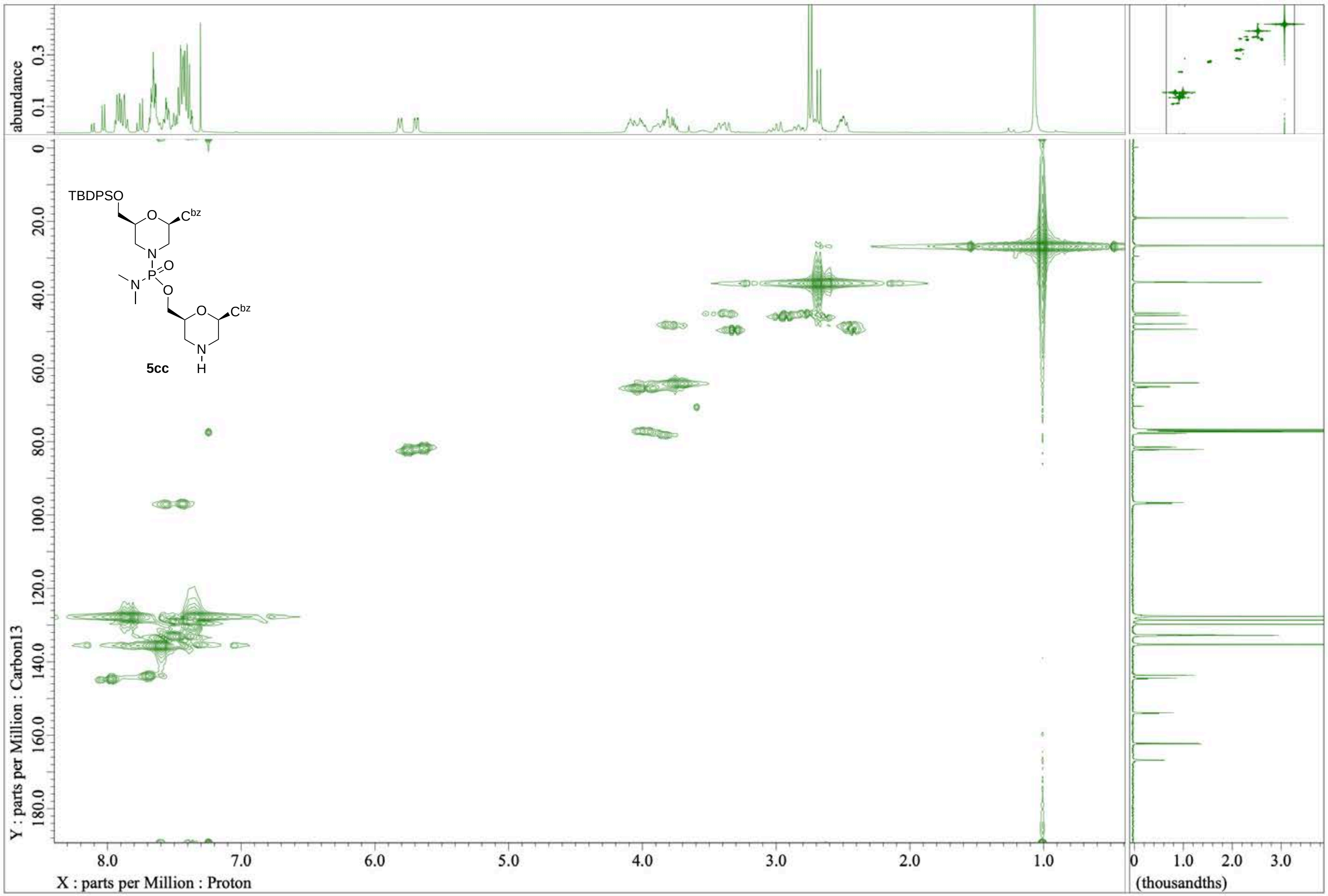
abundance
0.1
0.3
TBDPSO
O
C^{bz}
N
P
O
N
O
O
C^{bz}
N
H
5cc
Y : parts per Million : Carbon13
0
20.0
40.0
60.0
80.0
100.0
120.0
140.0
160.0
180.0
X : parts per Million : Proton
8.0
7.0
6.0
5.0
4.0
3.0
2.0
1.0
0
1.0
2.0
3.0
(thousandths)

HMBC ($CDCl_3$)

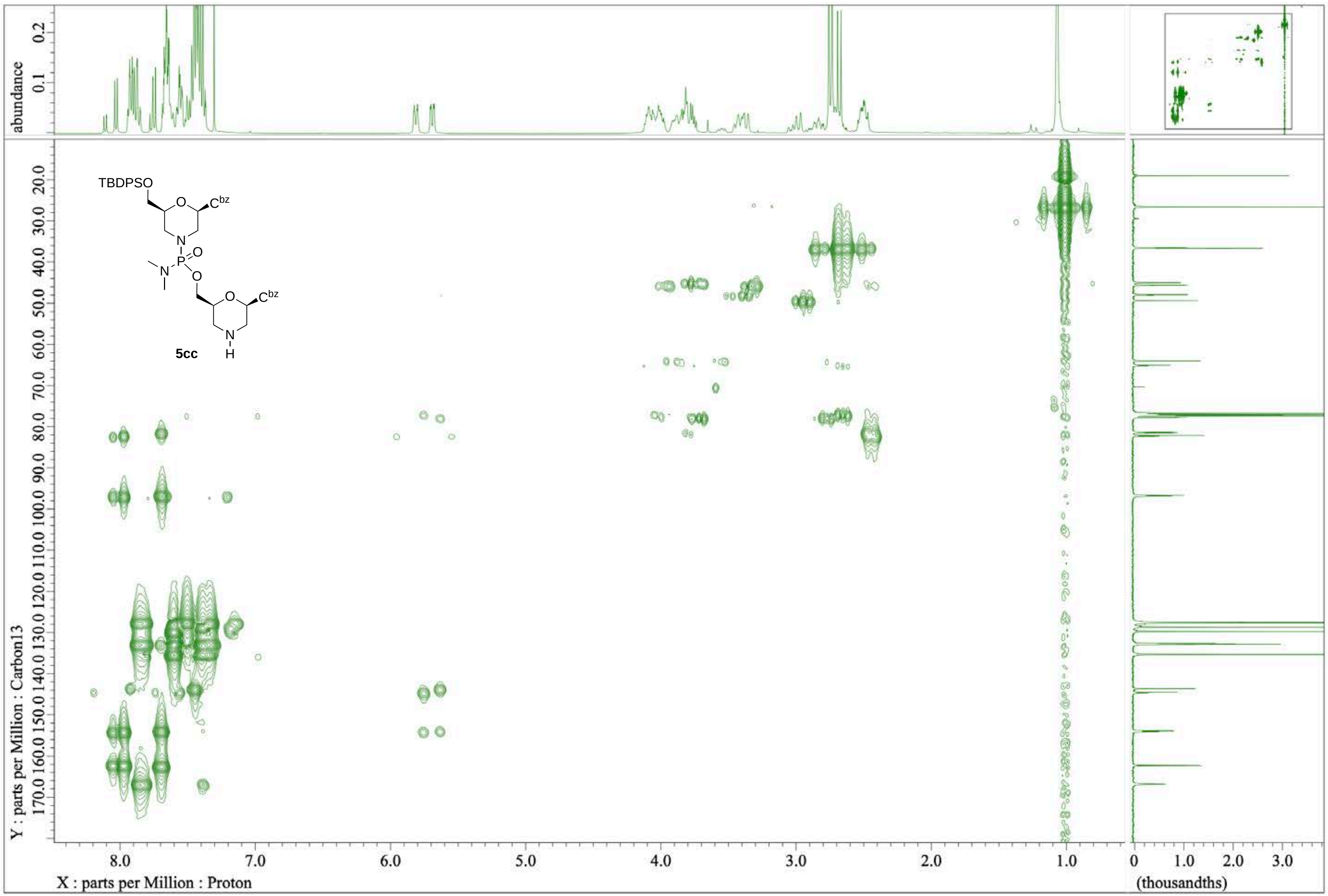
abundance
0.2
0.1
TBDPSO
O
C^{bz}
N
P
O
N
O
O
C^{bz}
N
H
5cc
Y : parts per Million : Carbon13
20.0
30.0
40.0
50.0
60.0
70.0
80.0
90.0
100.0
110.0
120.0
130.0
140.0
150.0
160.0
170.0
8.0
7.0
6.0
5.0
4.0
3.0
2.0
1.0
X : parts per Million : Proton
0
1.0
2.0
3.0
(thousandths)

$^{31}P\{^{1}H\}$ NMR (162 MHz, $CDCl_3$)

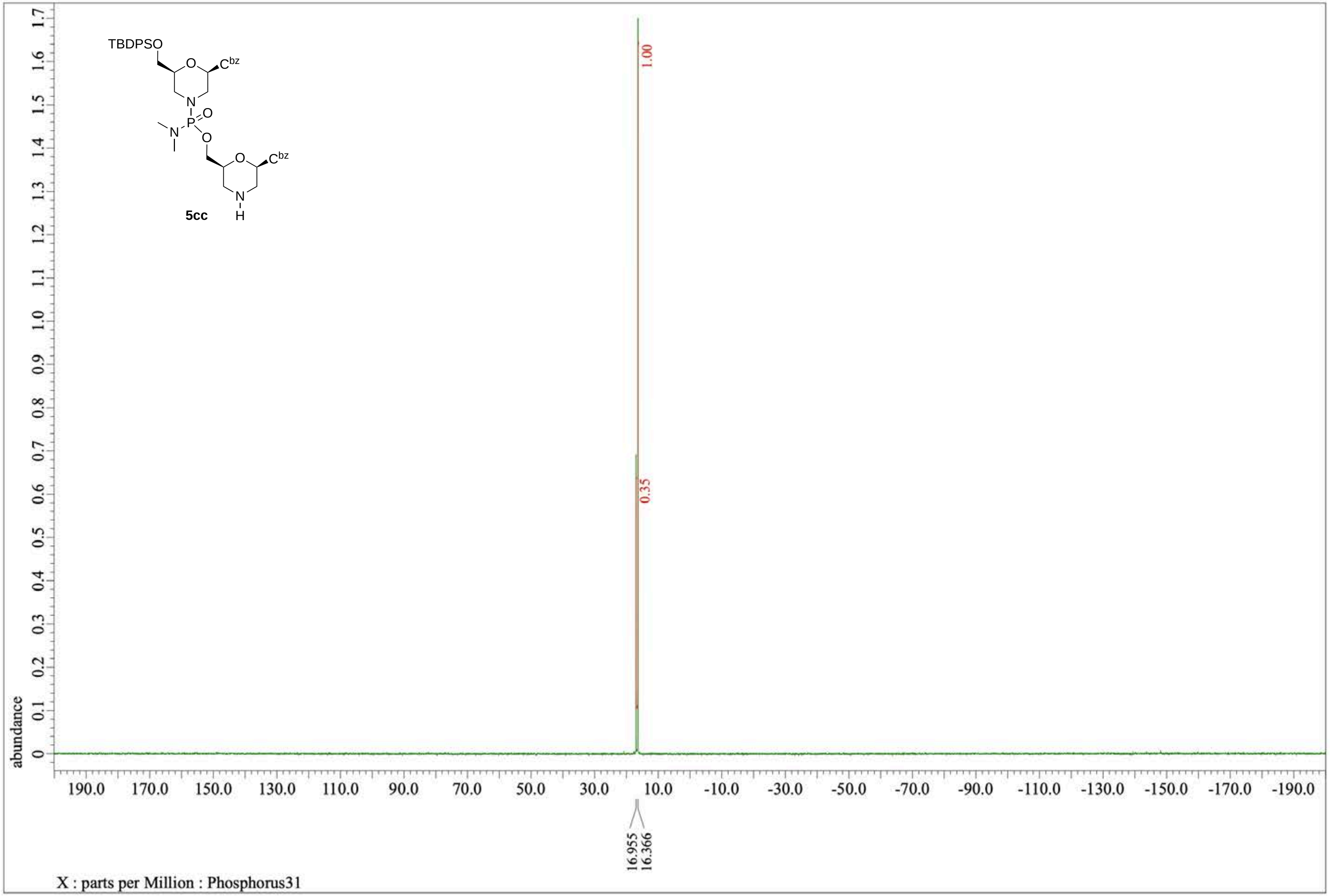

Mass spectra

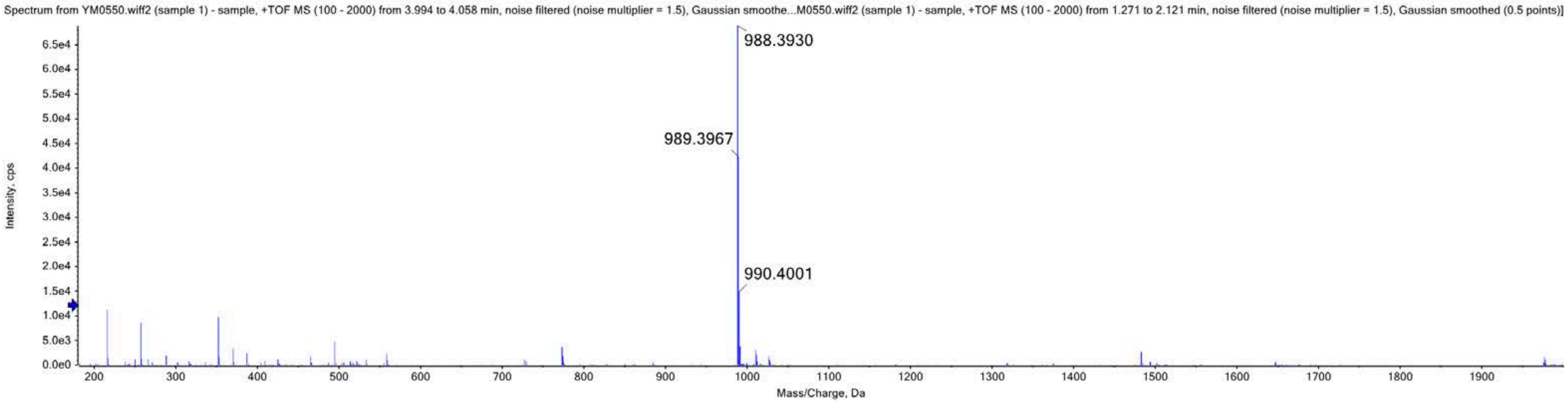

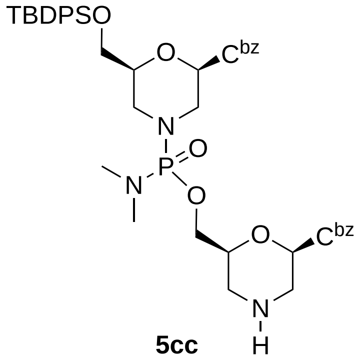

5cc

ESI-MS *m/z* calcd for $C_{50}H_{59}N_9O_9PSi$ $[M+H]^+$, 988.3937; found 988.3930.

^{1}H-NMR (400 MHz, $CDCl_3$)

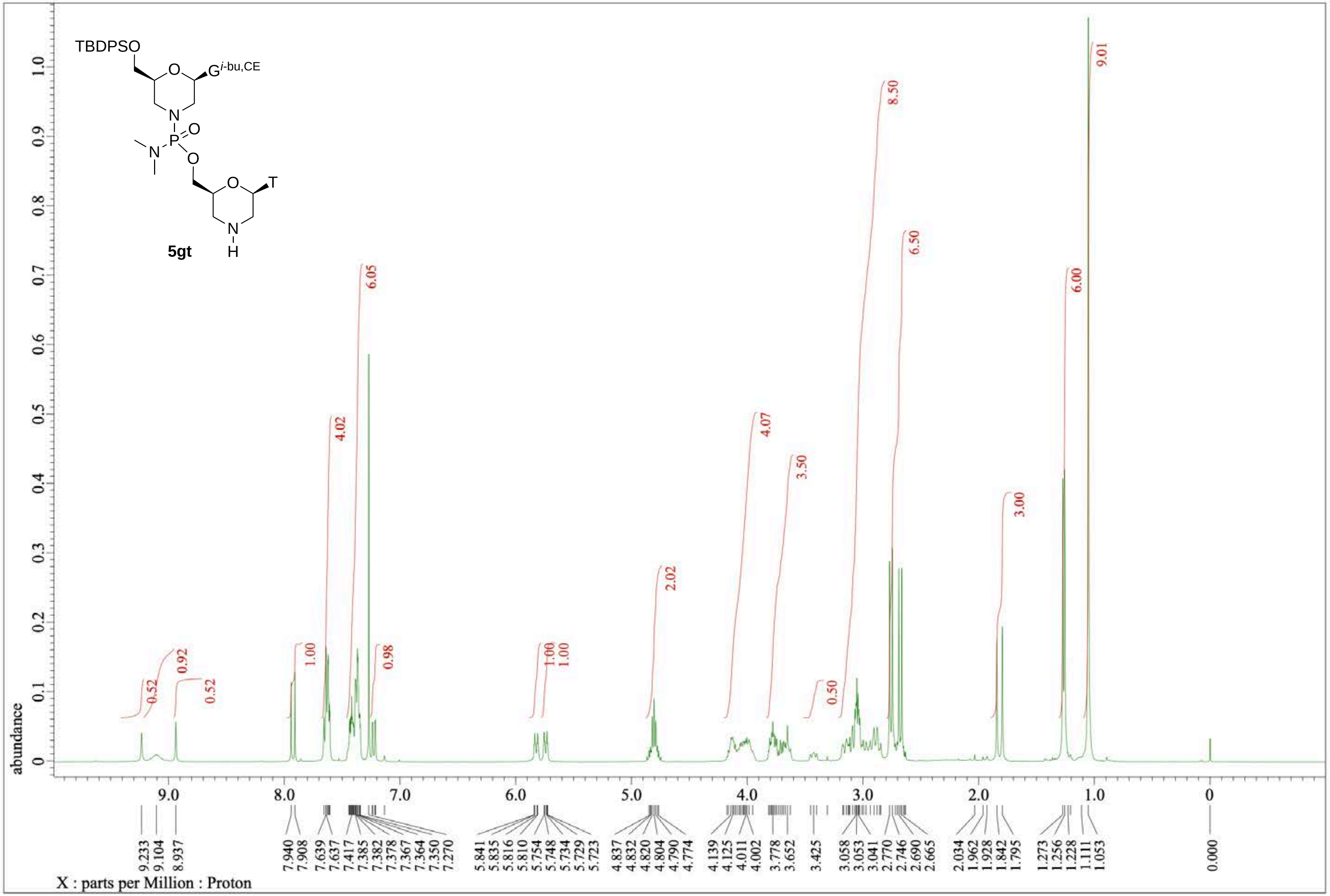

$^{13}C\{H\}$-NMR (101 MHz, $CDCl_3$)

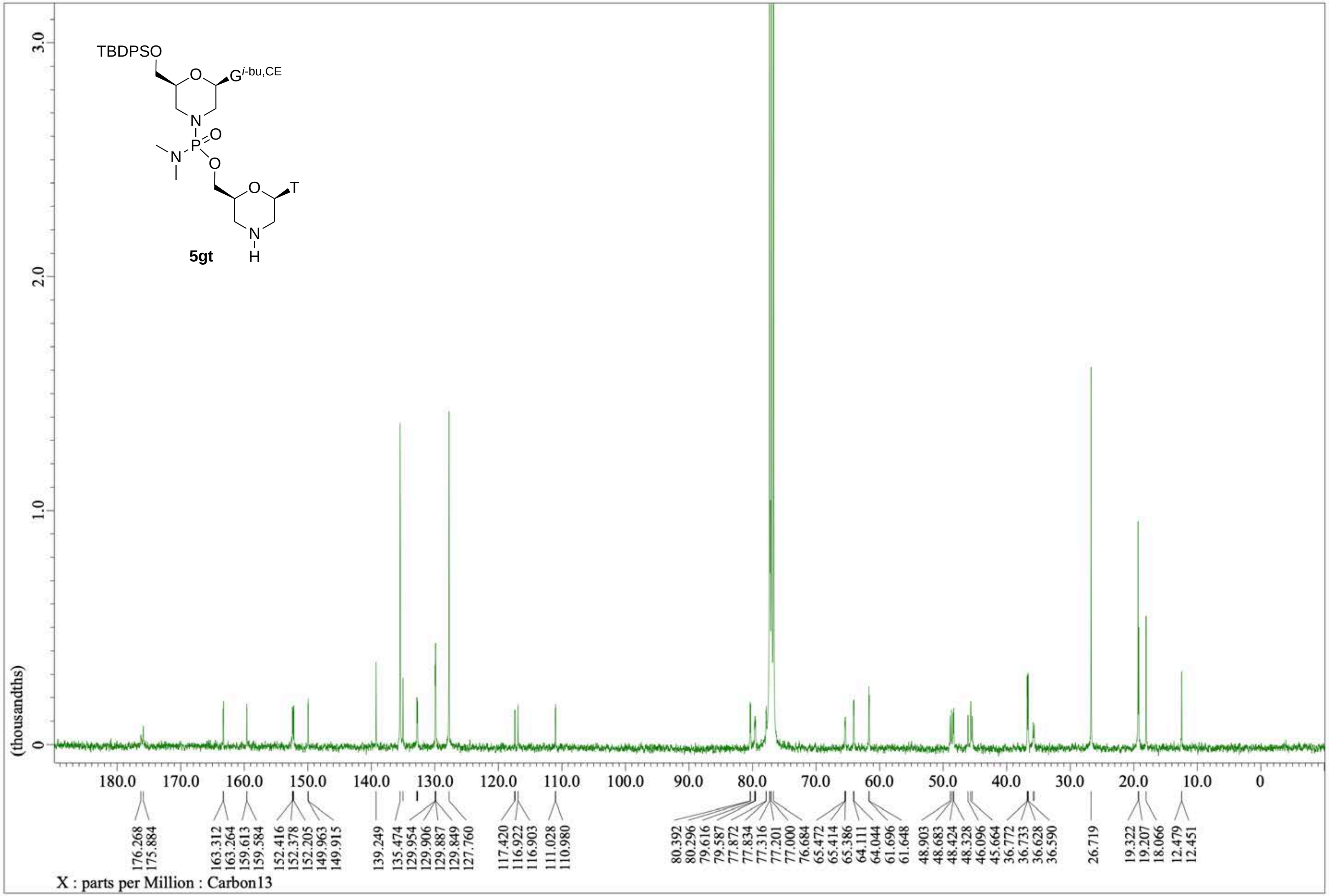

HMQC ($CDCl_3$)

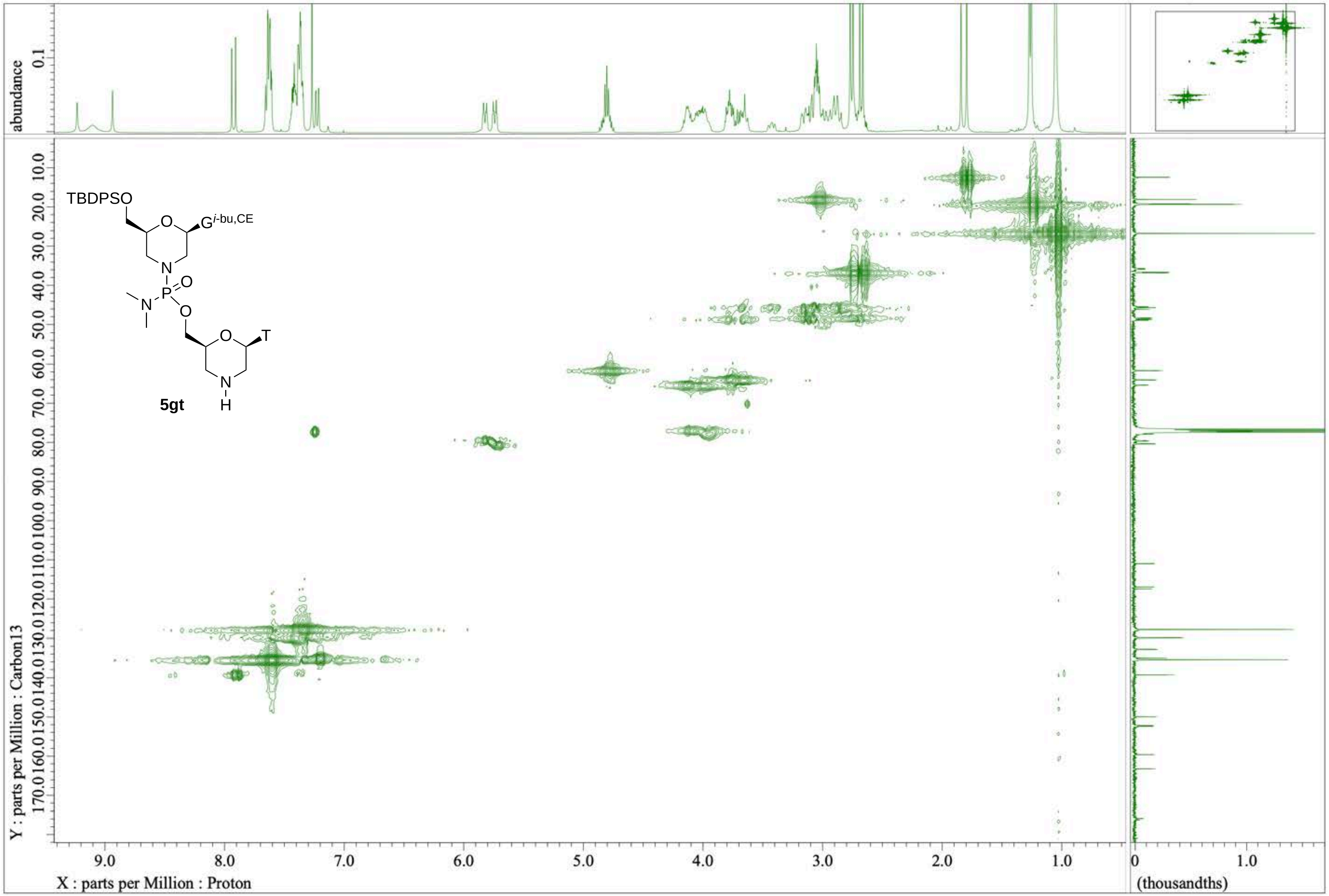
abundance
0.1
TBDPSO
$G^{i\text{-bu,CE}}$
N
P
O
T
H
5gt
Y : parts per Million : Carbon13
X : parts per Million : Proton
(thousandths)
9.0
8.0
7.0
6.0
5.0
4.0
3.0
2.0
1.0
10.0
20.0
30.0
40.0
50.0
60.0
70.0
80.0
90.0
100.0
110.0
120.0
130.0
140.0
150.0
160.0
170.0

HMBC ($CDCl_3$)

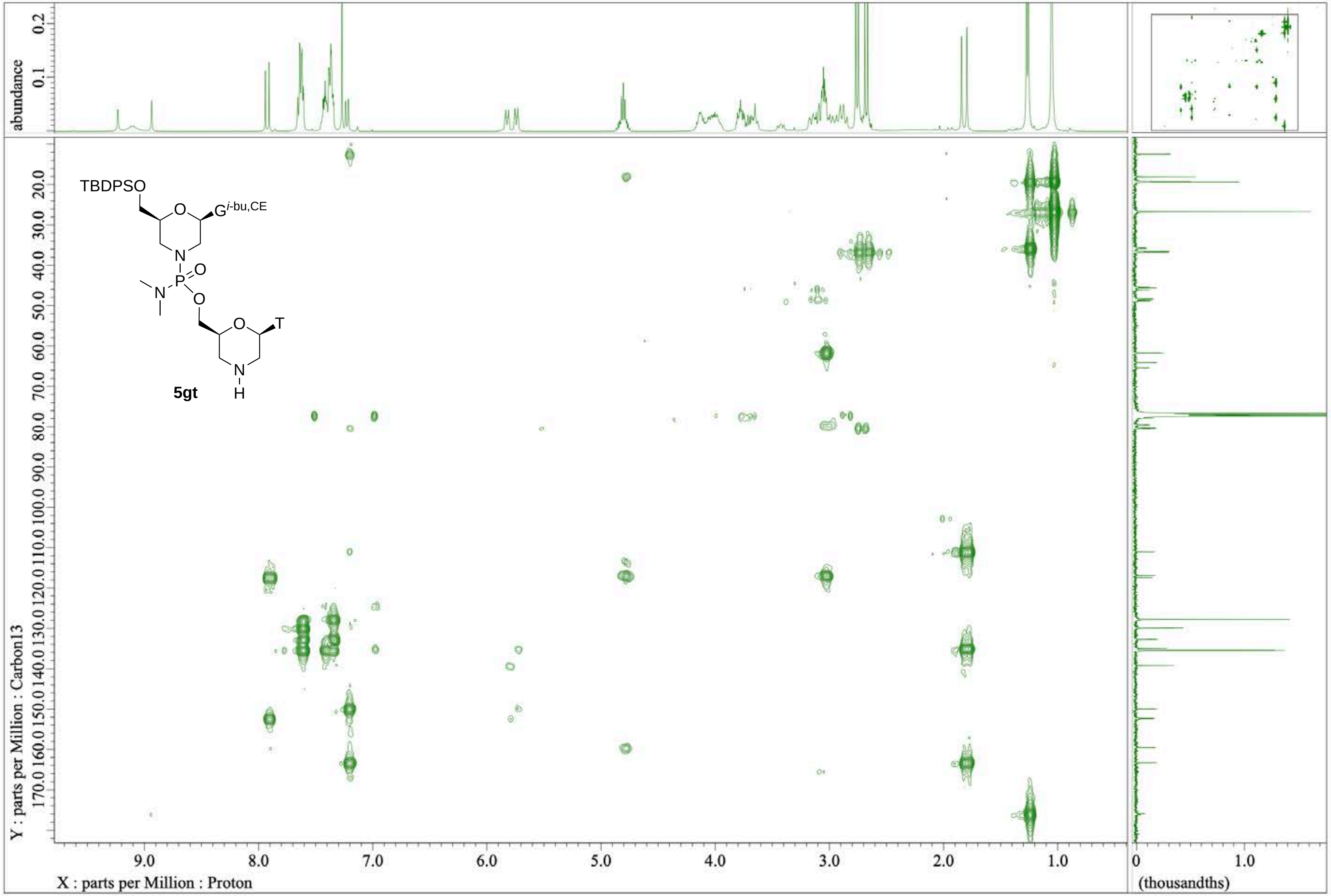

TBDPSO
O
G^{i-bu,CE}
N
P
O
N
O
O
T
N
H
5gt
abundance
0.1
0.2
Y : parts per Million : Carbon13
X : parts per Million : Proton
(thousandths)

$^{31}P\{^{1}H\}$ NMR (162 MHz, $CDCl_3$)

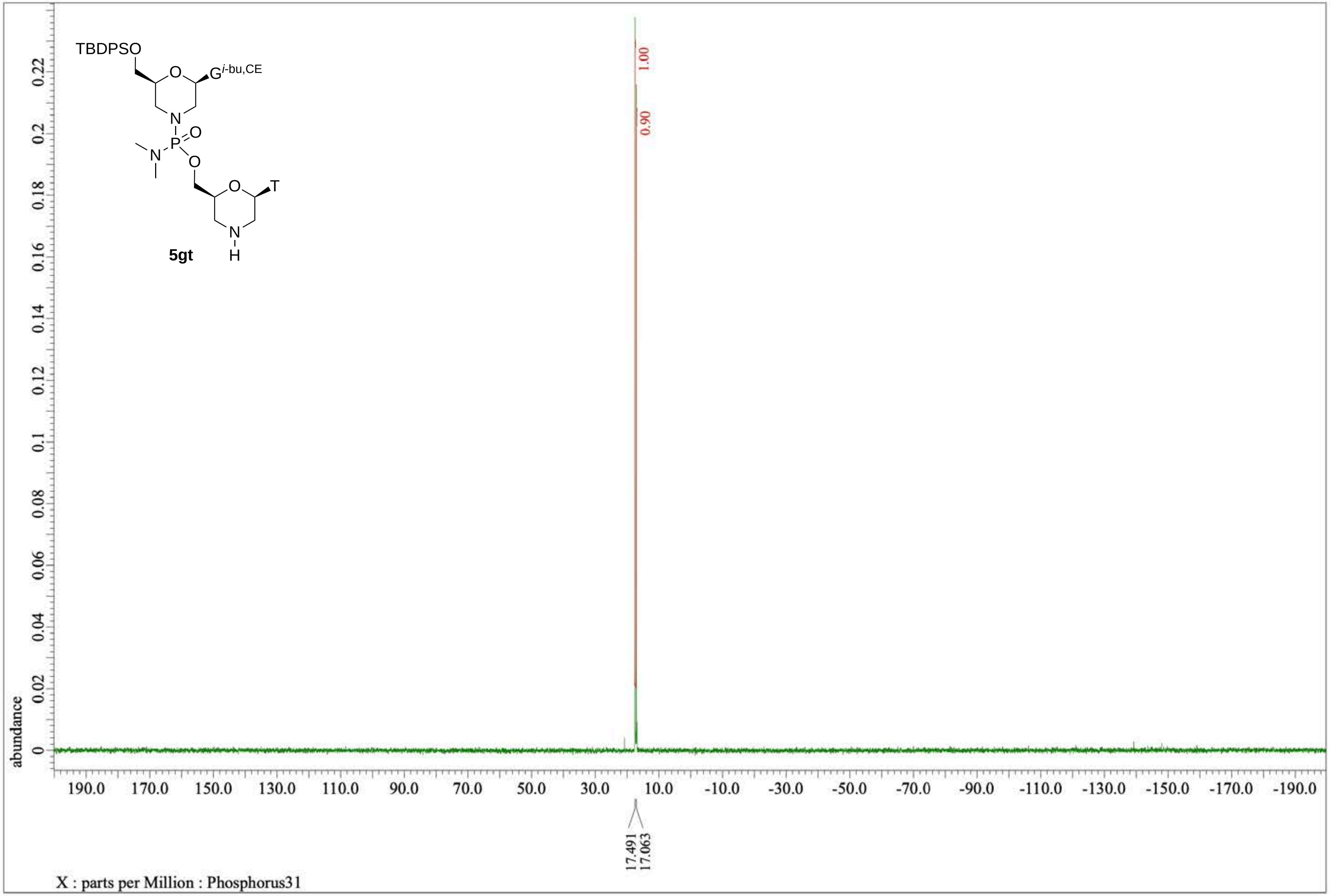

Mass spectra

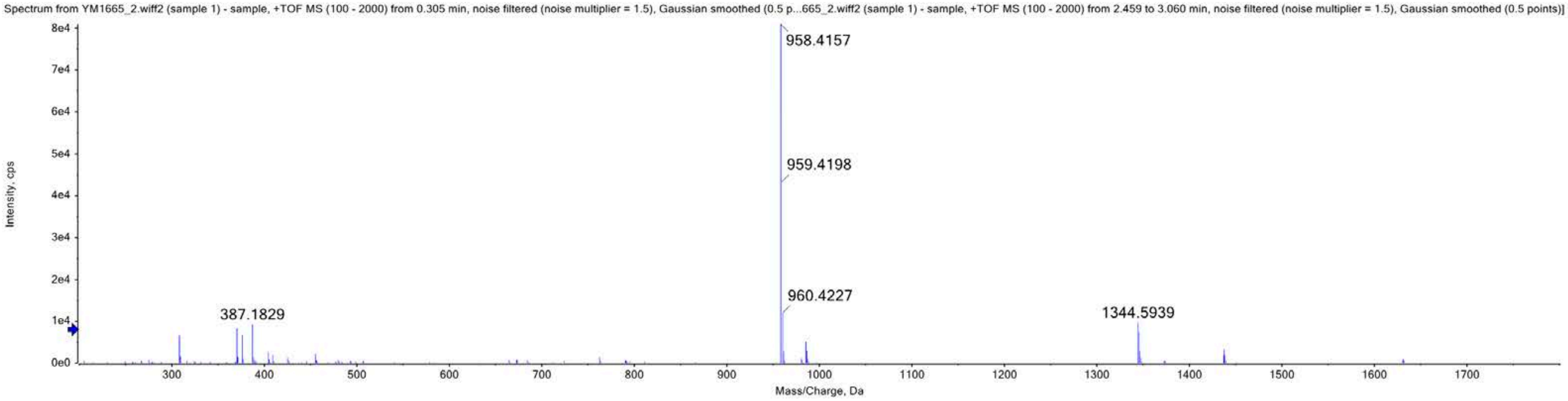

ESI-MS *m/z* calcd for $C_{45}H_{61}N_{11}O_9PSi$ $[M+H]^+$, 958.4155; found 958.4157.

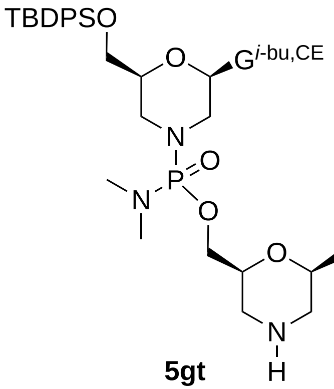

5gt

^{1}H-NMR (400 MHz, $CDCl_3$)

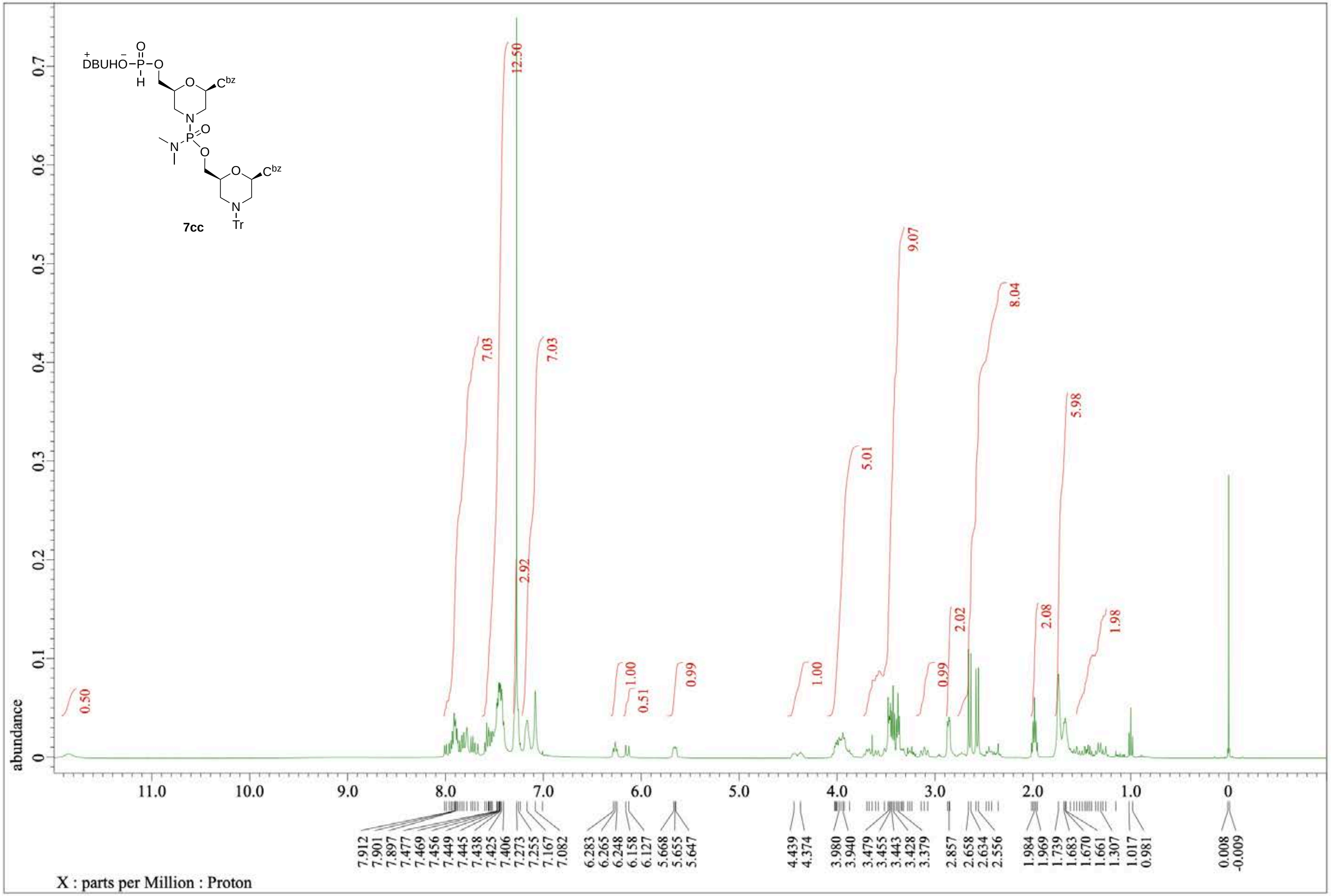

$^{13}C\{H\}$-NMR (101 MHz, $CDCl_3$)

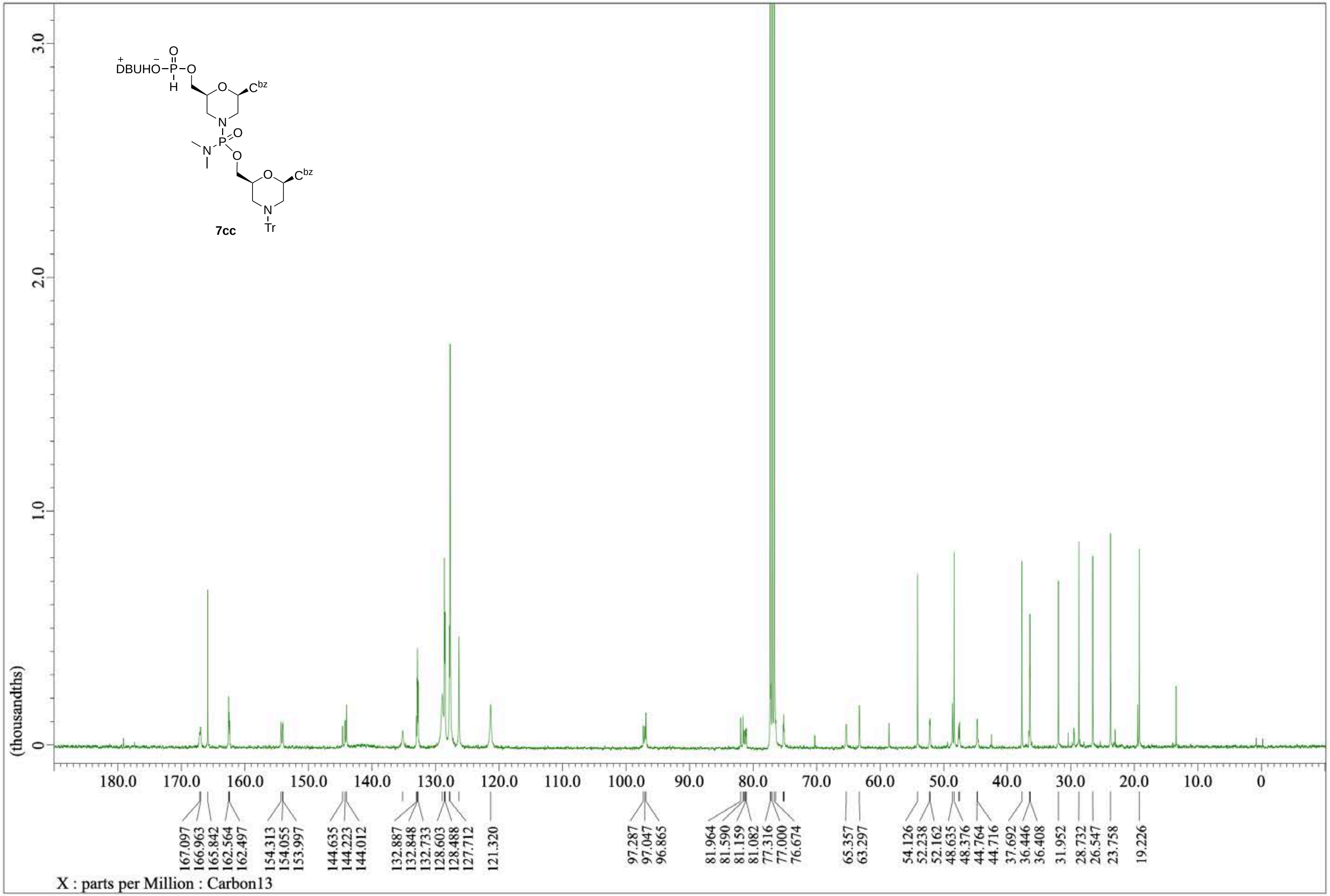

HMQC ($CDCl_3$)

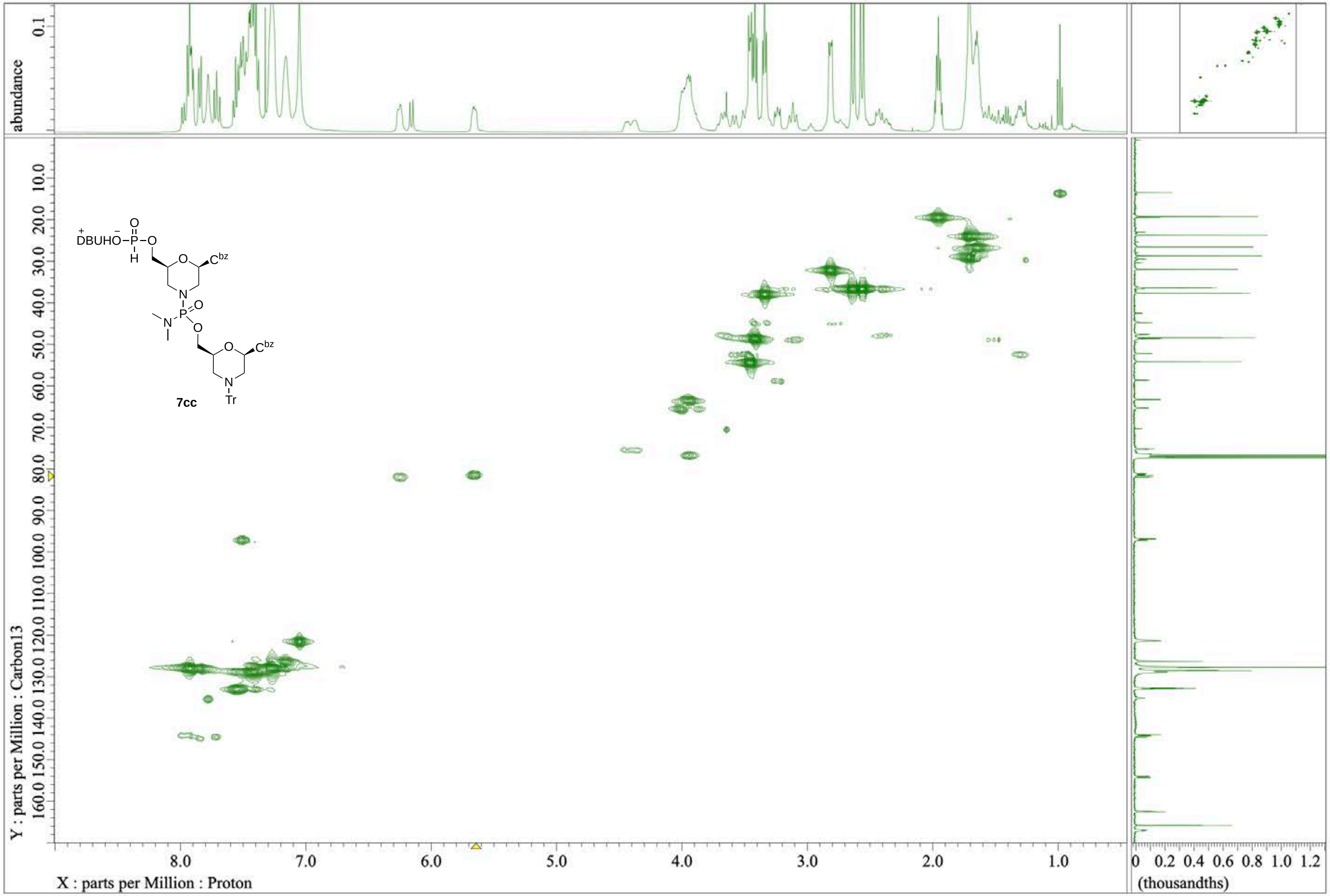

abundance
0.1
Y : parts per Million : Carbon13
10.0 20.0 30.0 40.0 50.0 60.0 70.0 80.0 90.0 100.0 110.0 120.0 130.0 140.0 150.0 160.0
X : parts per Million : Proton
8.0 7.0 6.0 5.0 4.0 3.0 2.0 1.0
0 0.2 0.4 0.6 0.8 1.0 1.2
(thousandths)
DBUHO
C^bz
Tr
7cc

HMBC ($CDCl_3$)

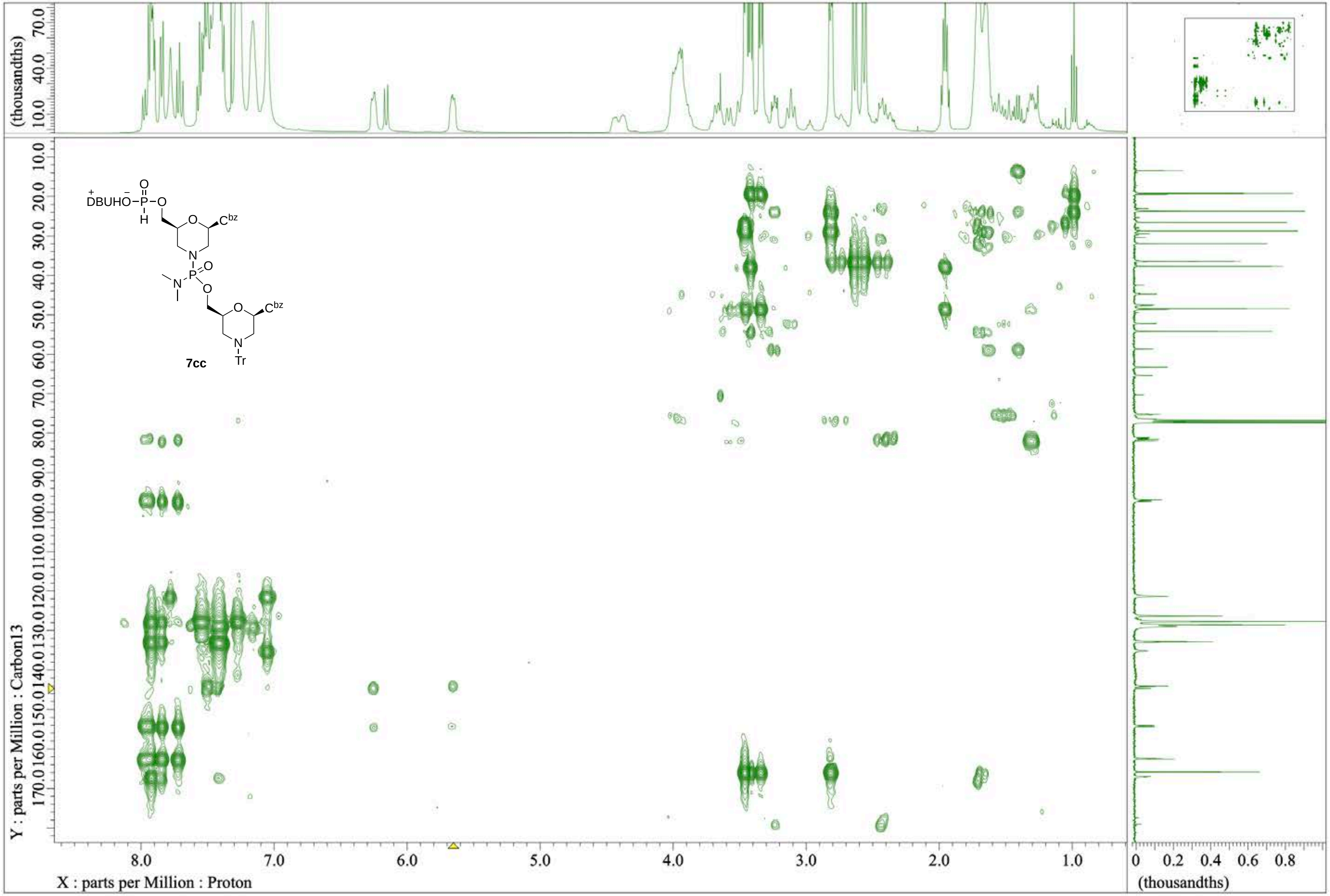
7cc
(thousandths)
Y : parts per Million : Carbon13
X : parts per Million : Proton
(thousandths)

$^{31}P\{^1H\}$ NMR (162 MHz, $CDCl_3$)

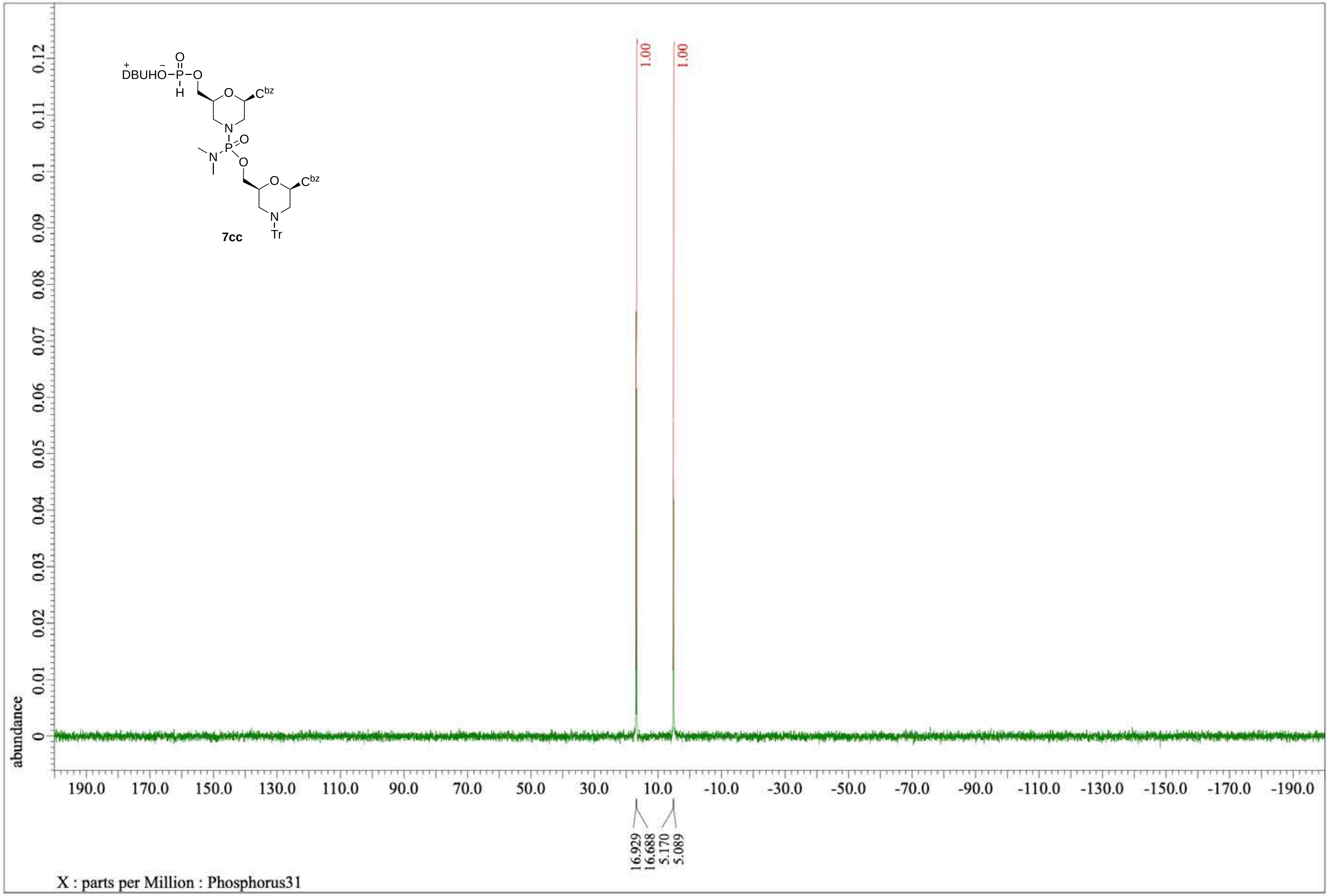

Mass spectra

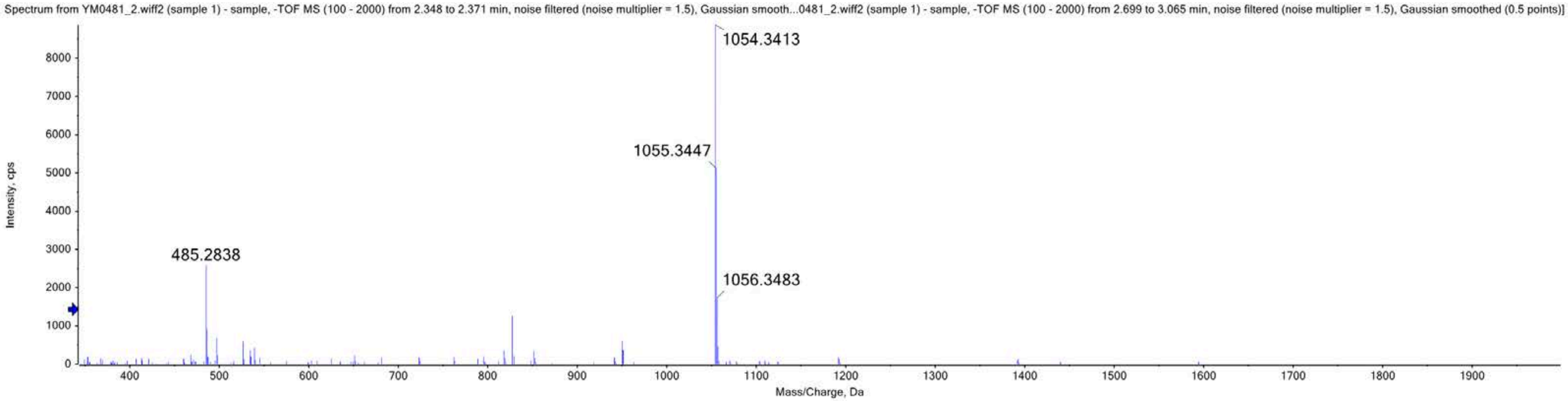

ESI-MS *m/z* calcd for $C_{53}H_{54}N_9O_{11}P_2$ [M-H-DBU]$^-$, 1054.3424; found 1054.3413.

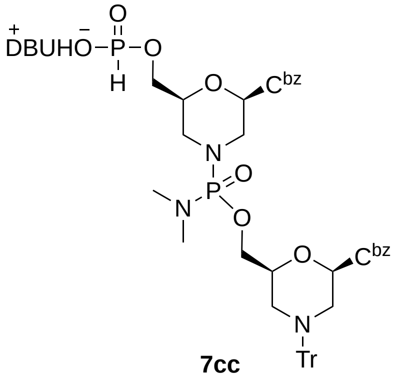

7cc

^{1}H-NMR (400 MHz, $CDCl_3$)

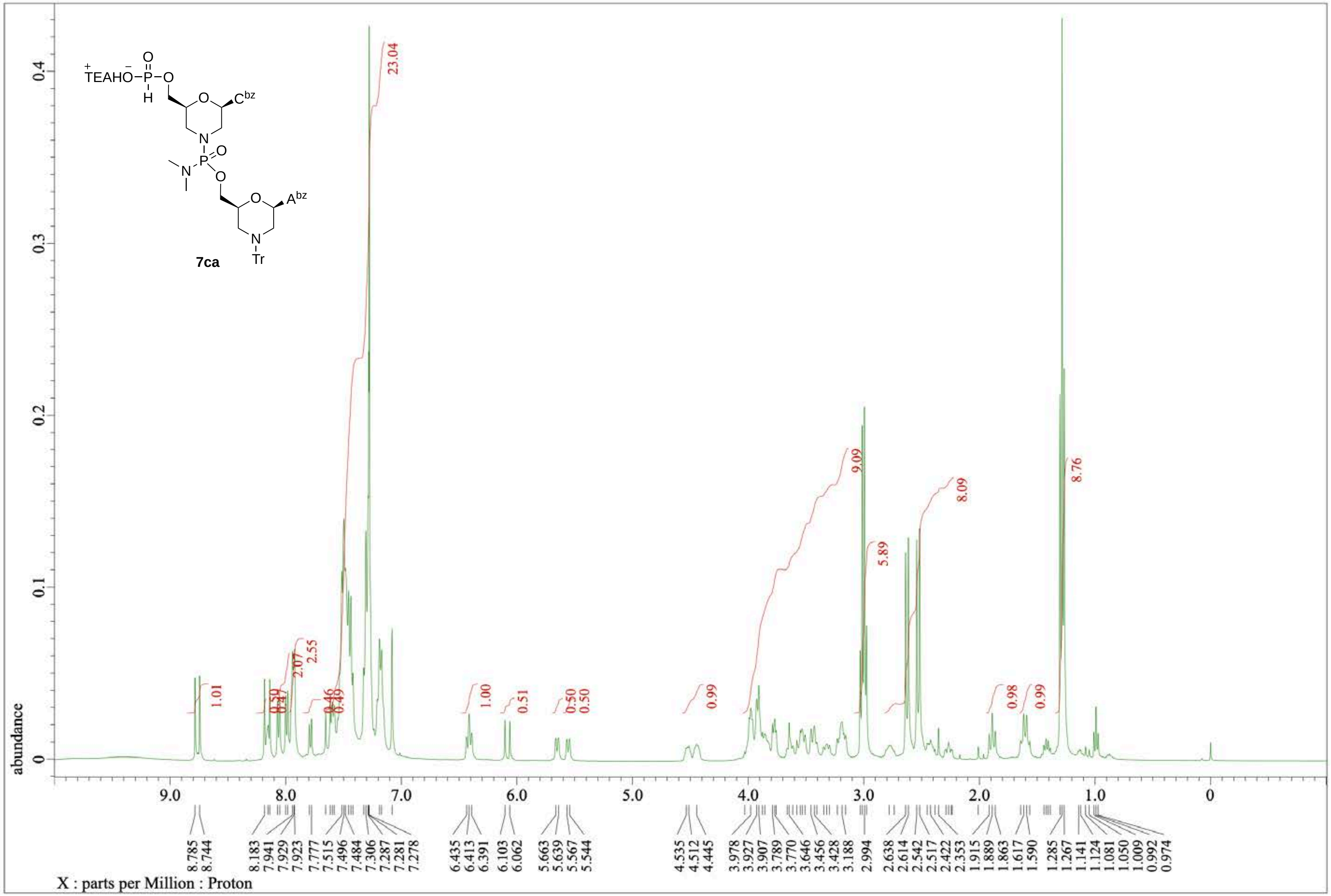

$^{13}C\{H\}$-NMR (101 MHz, $CDCl_3$)

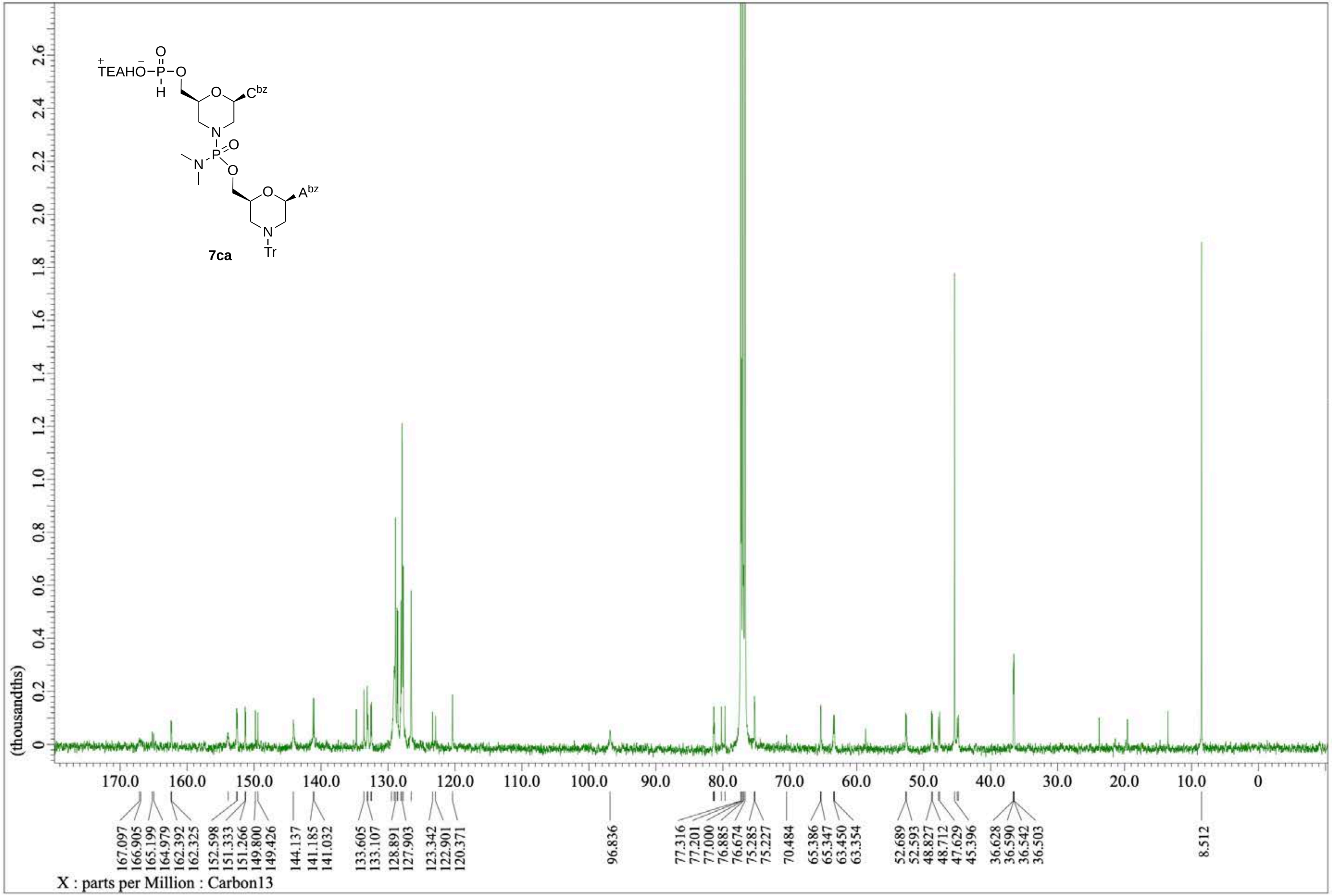

HMQC (CDCl$_3$)

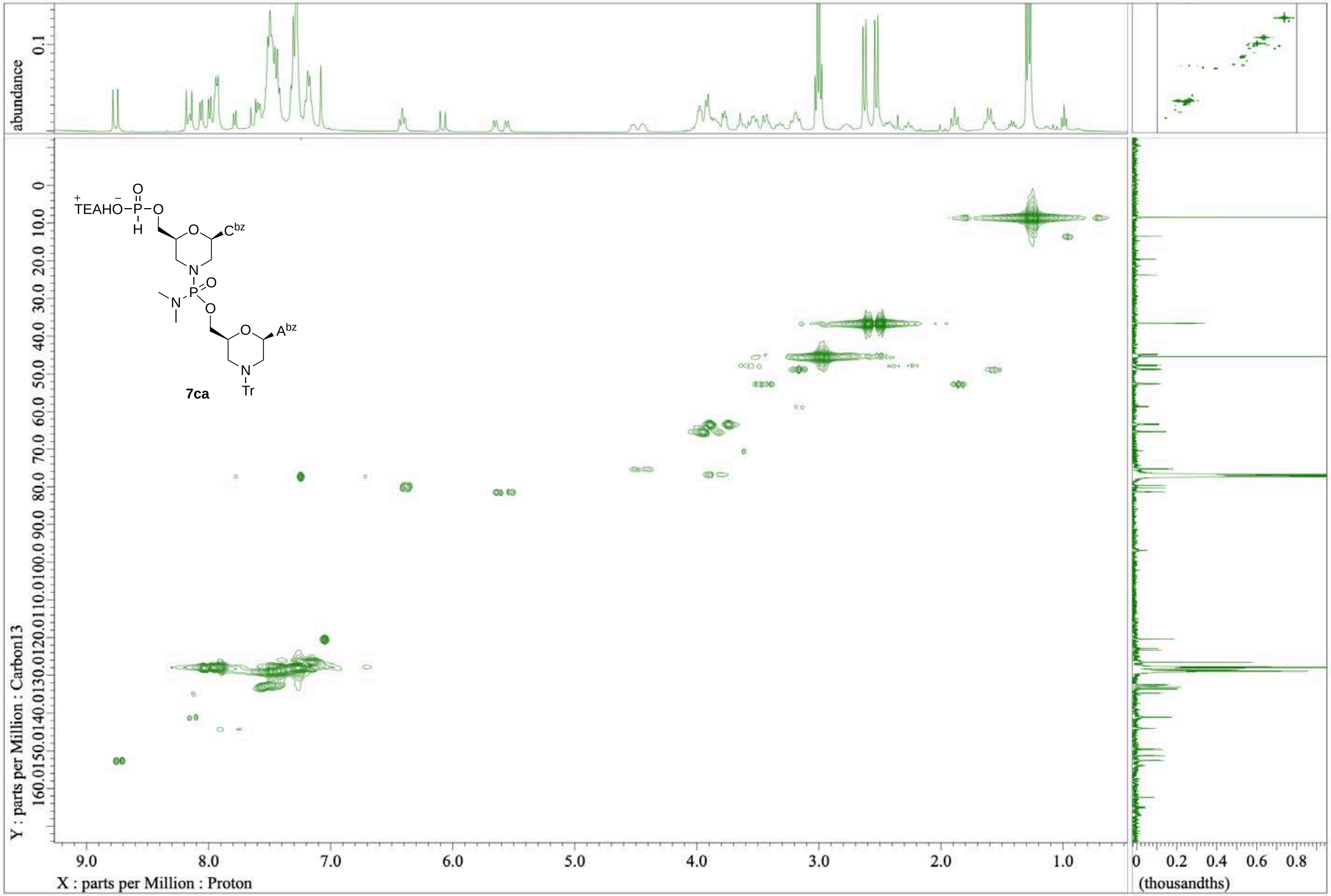

abundance
0.1
TEAHO−P−O
O
H
O
C^bz
N
P
O
N
O
O
A^bz
N
Tr
7ca
Y : parts per Million : Carbon13
X : parts per Million : Proton
9.0
8.0
7.0
6.0
5.0
4.0
3.0
2.0
1.0
0
10.0
20.0
30.0
40.0
50.0
60.0
70.0
80.0
90.0
100.0
110.0
120.0
130.0
140.0
150.0
160.0
0 0.2 0.4 0.6 0.8
(thousandths)

HMBC ($CDCl_3$)

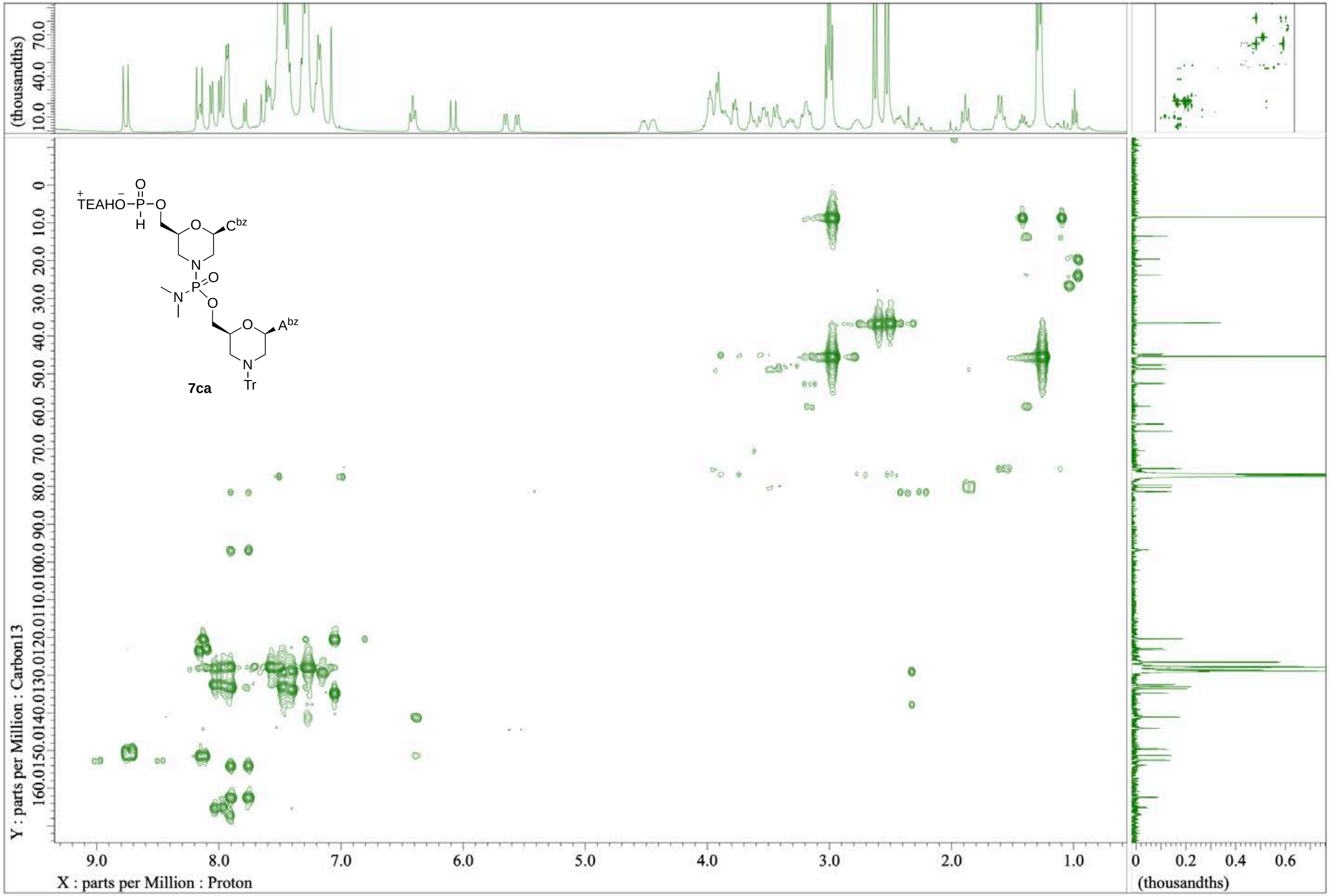

TEAHO
P
O
H
N
C^bz
A^bz
Tr
7ca
(thousandths)
Y : parts per Million : Carbon13
X : parts per Million : Proton

$^{31}P\{^{1}H\}$ NMR (162 MHz, $CDCl_3$)

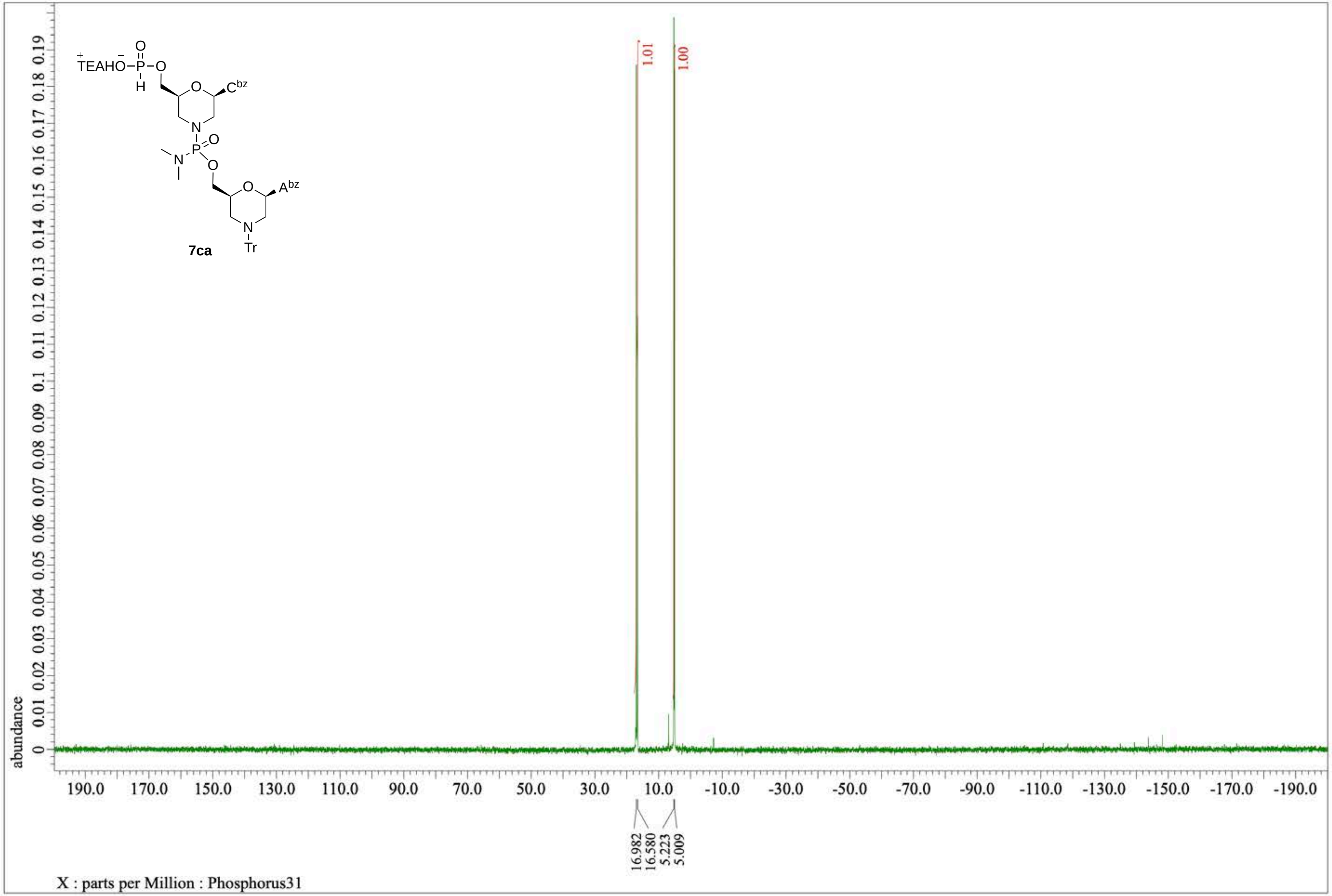

Mass spectra

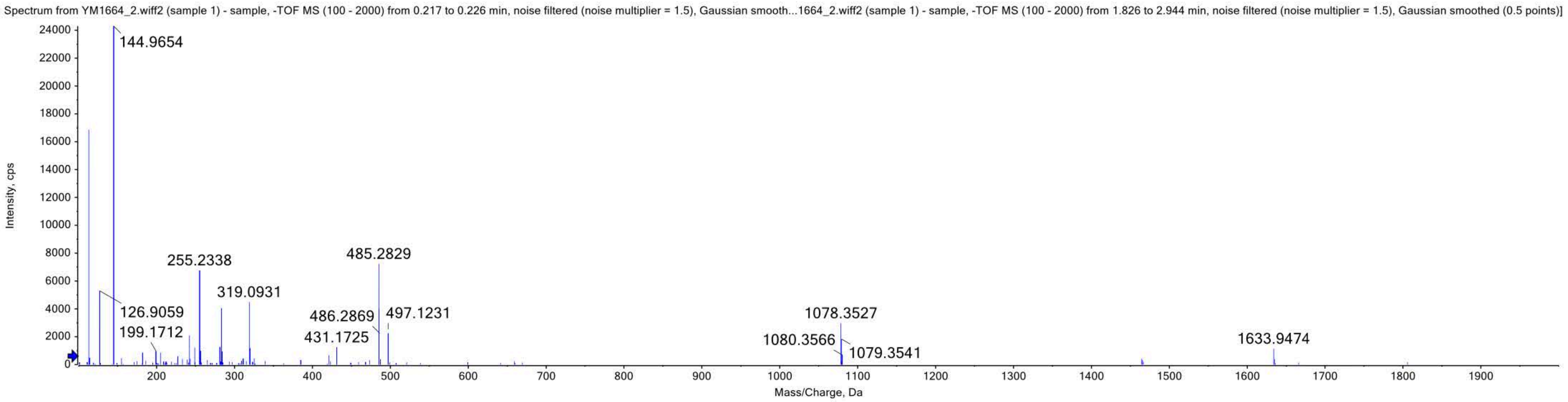

ESI-MS m/z calcd for $C_{54}H_{54}N_{11}O_{10}P_2$ [M-H-TEA]$^-$, 1078.3536; found 1078.3527.

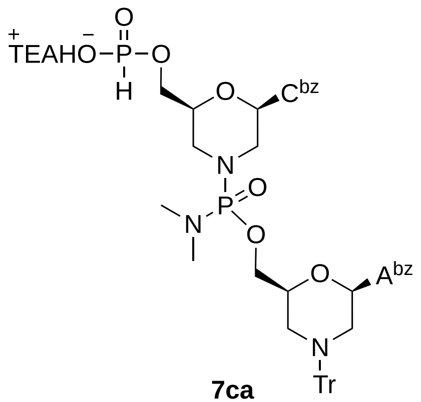

7ca

^{1}H-NMR (400 MHz, $CDCl_3$)

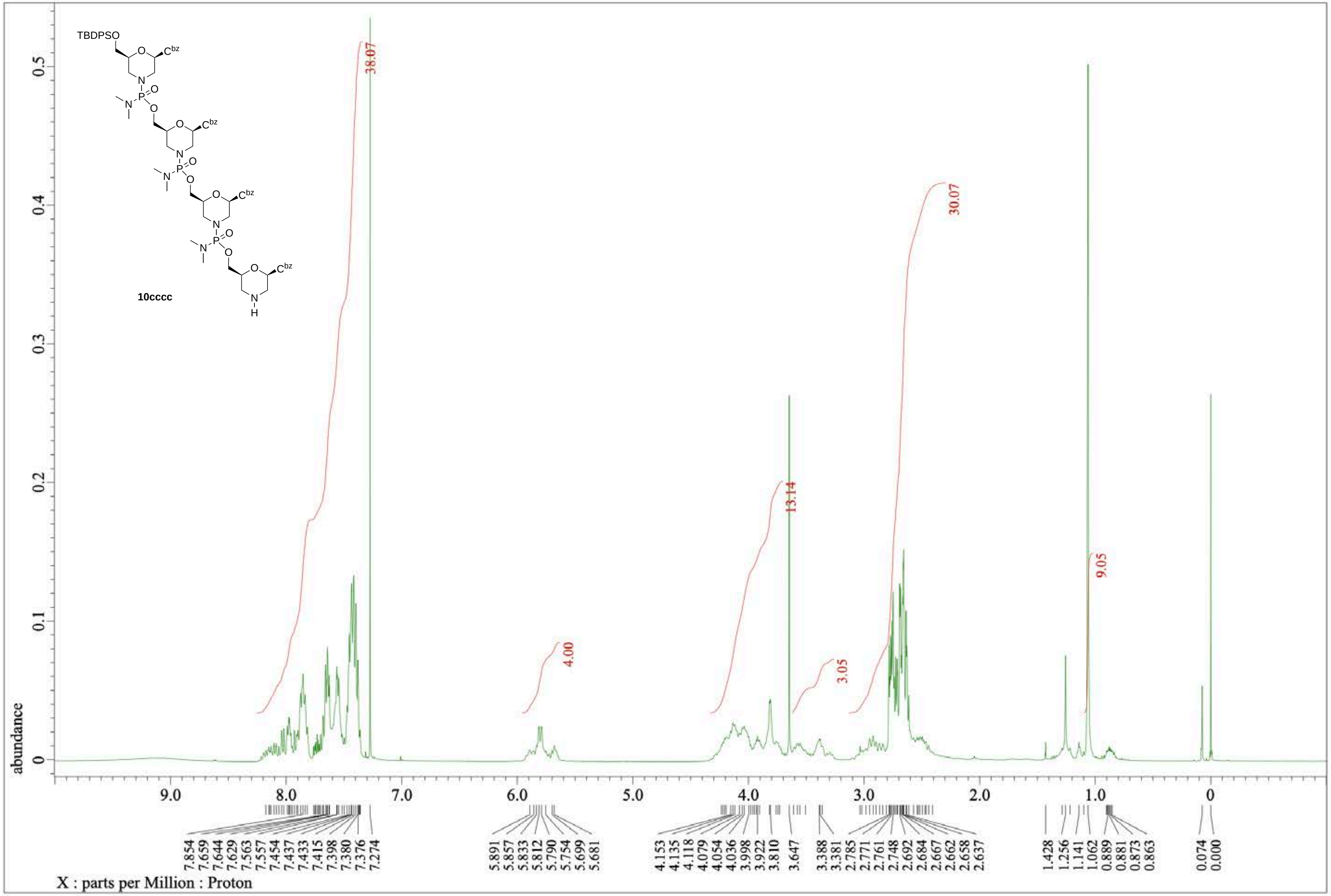

$^{13}C\{H\}$-NMR (101 MHz, $CDCl_3$)

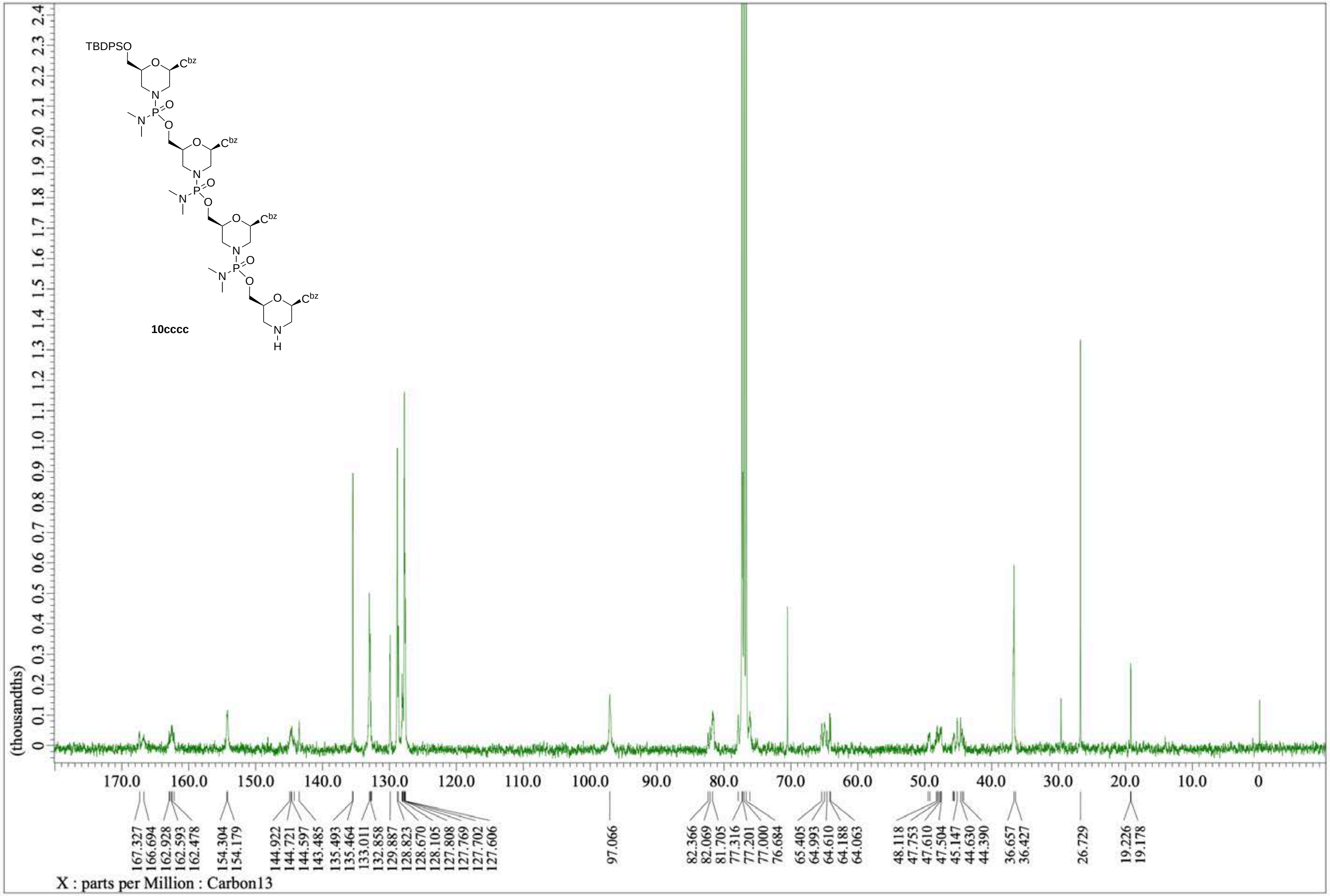

HMQC ($CDCl_3$)

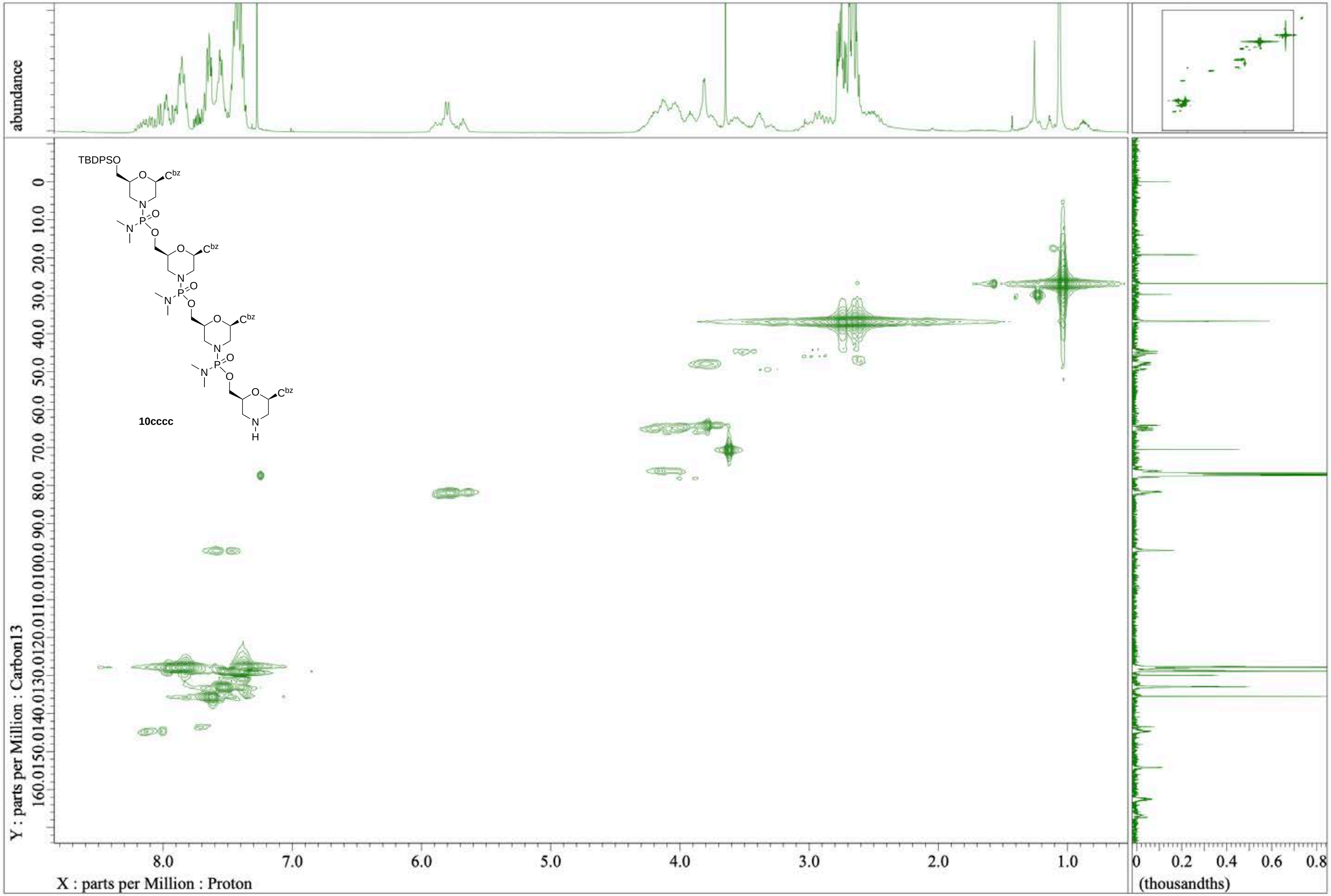
abundance
TBDPSO
Cbz
10cccc
Y : parts per Million : Carbon13
X : parts per Million : Proton
(thousandths)

HMBC ($CDCl_3$)

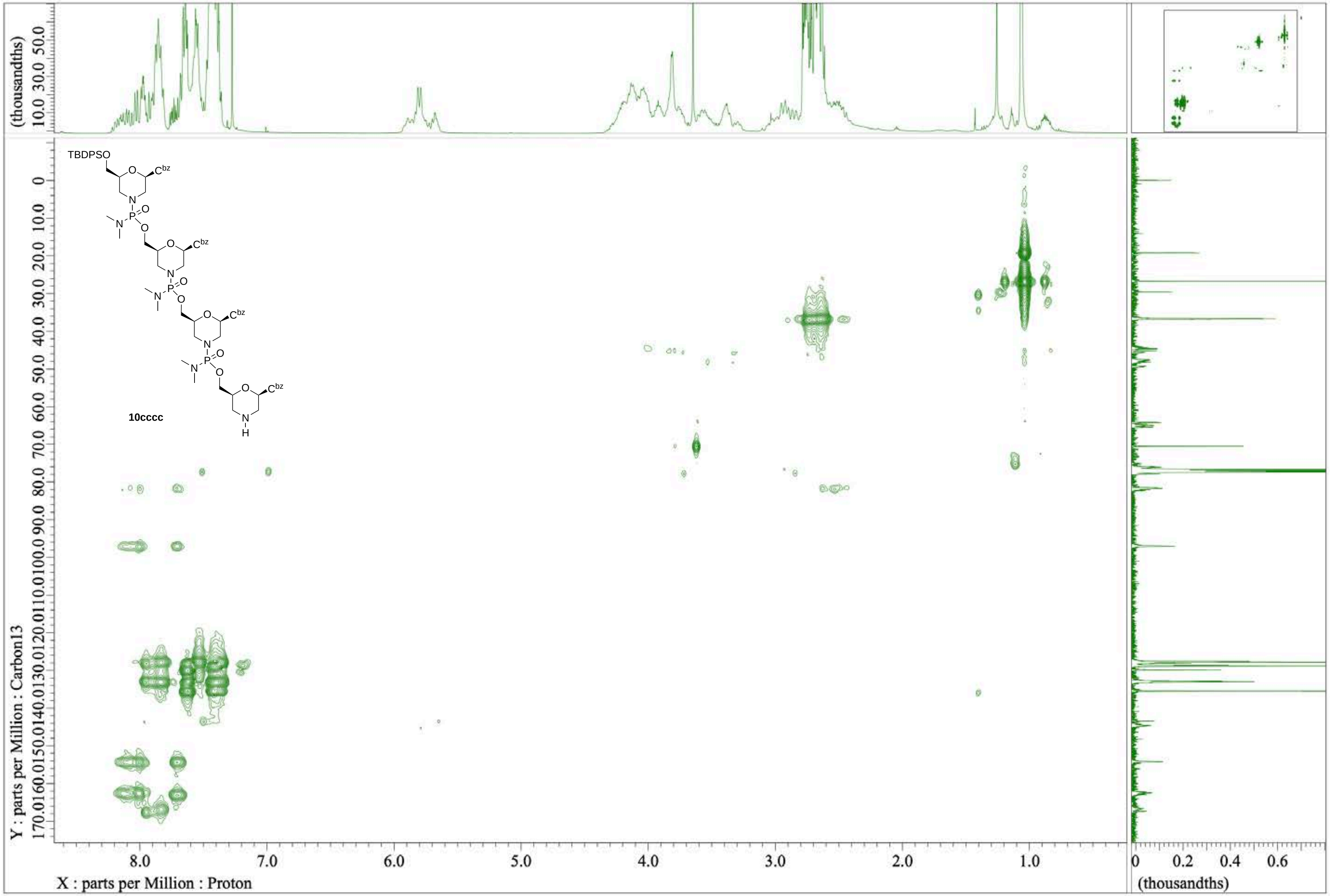

(thousandths)
10.0 30.0 50.0
TBDPSO
O
Cbz
N
P
O
N
O
10cccc
N
H
Y : parts per Million : Carbon13
0
10.0
20.0
30.0
40.0
50.0
60.0
70.0
80.0
90.0
100.0
110.0
120.0
130.0
140.0
150.0
160.0
170.0
8.0
7.0
6.0
5.0
4.0
3.0
2.0
1.0
X : parts per Million : Proton
0 0.2 0.4 0.6
(thousandths)

$^{31}P\{^1H\}$ NMR (162 MHz, $CDCl_3$)

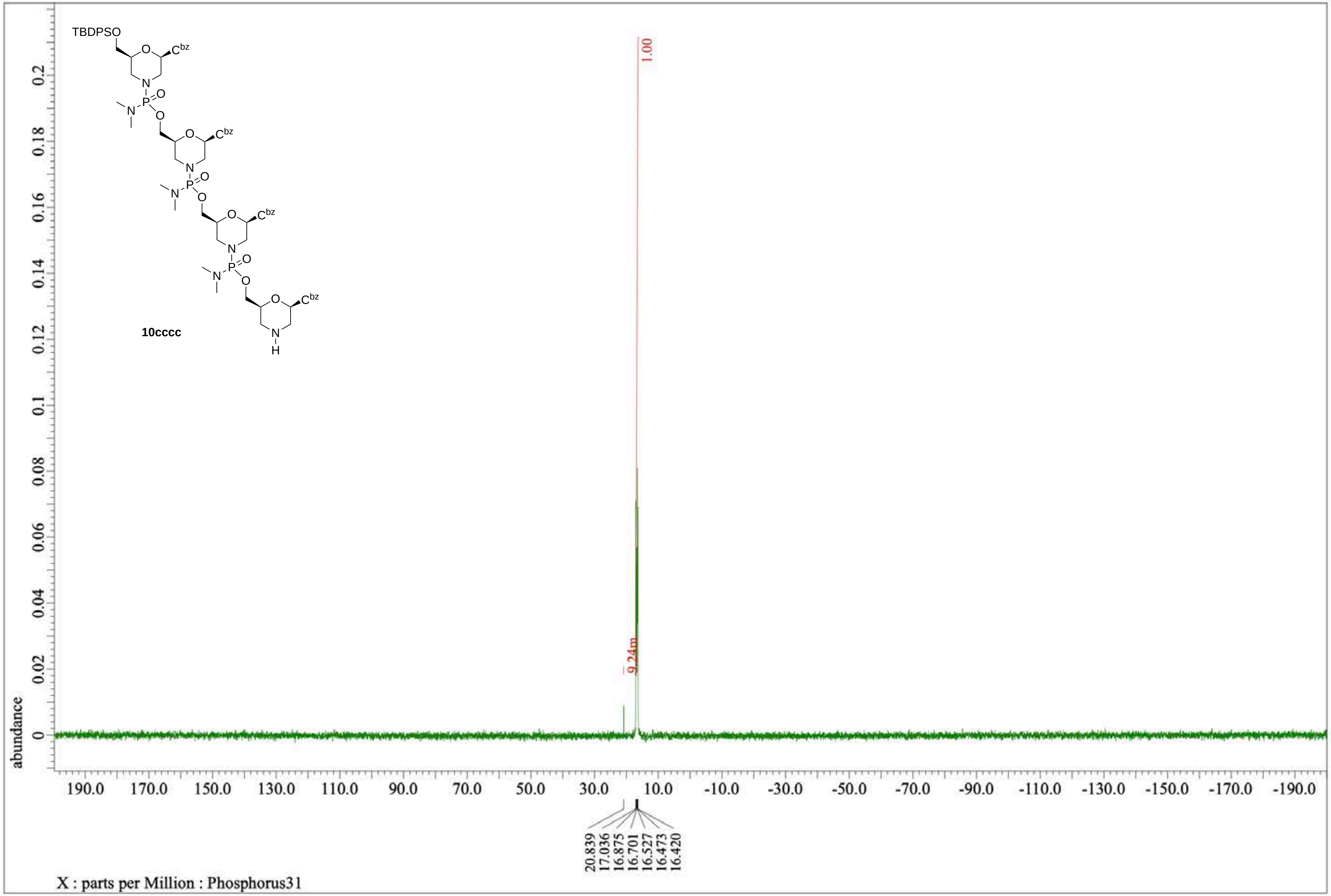

Mass spectra

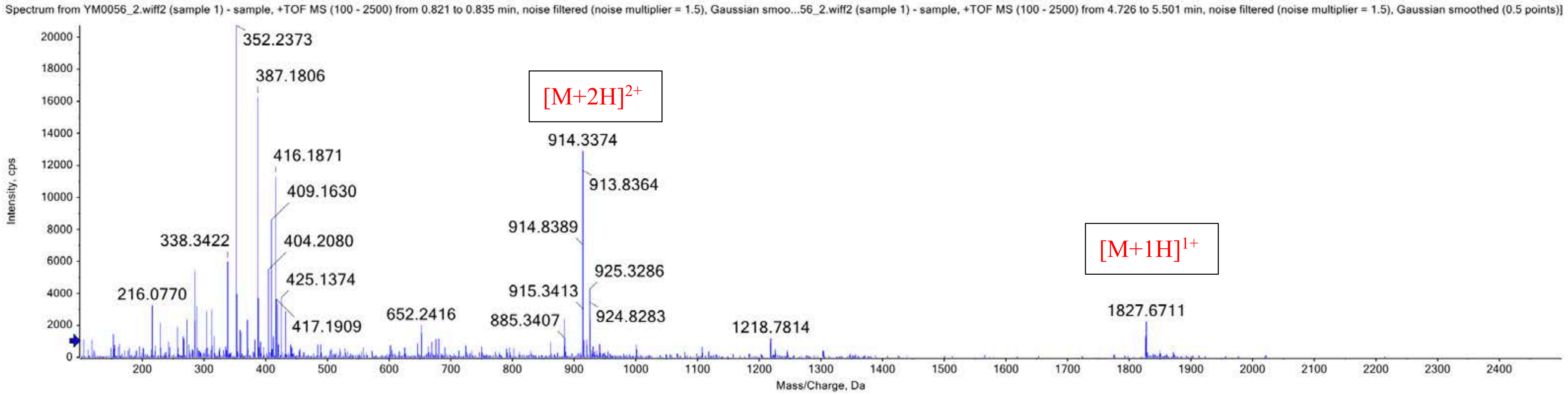

ESI-MS *m/z* calcd for $C_{86}H_{104}N_{19}O_{19}P_3Si$ $[M+2H]^{2+}$, 913.8364; found 913.8364.

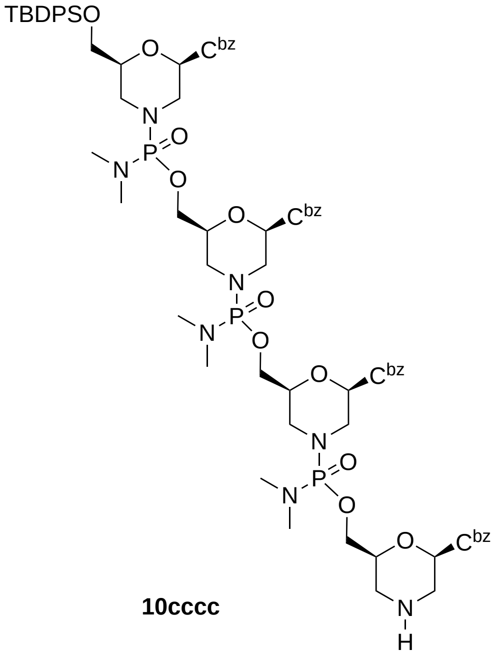

10cccc

^{1}H-NMR (400 MHz, $CDCl_3$)

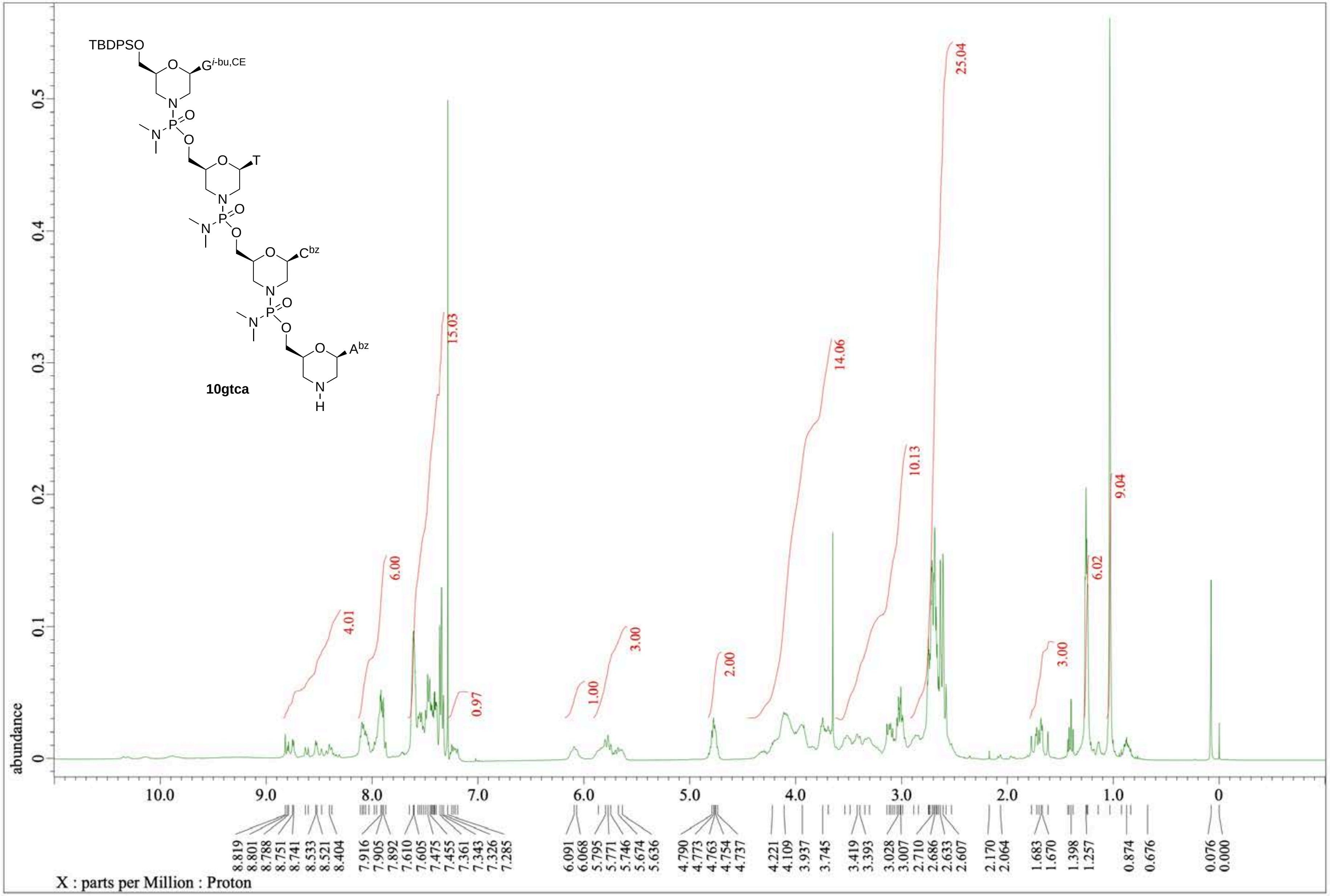

$^{13}C\{H\}$-NMR (101 MHz, $CDCl_3$)

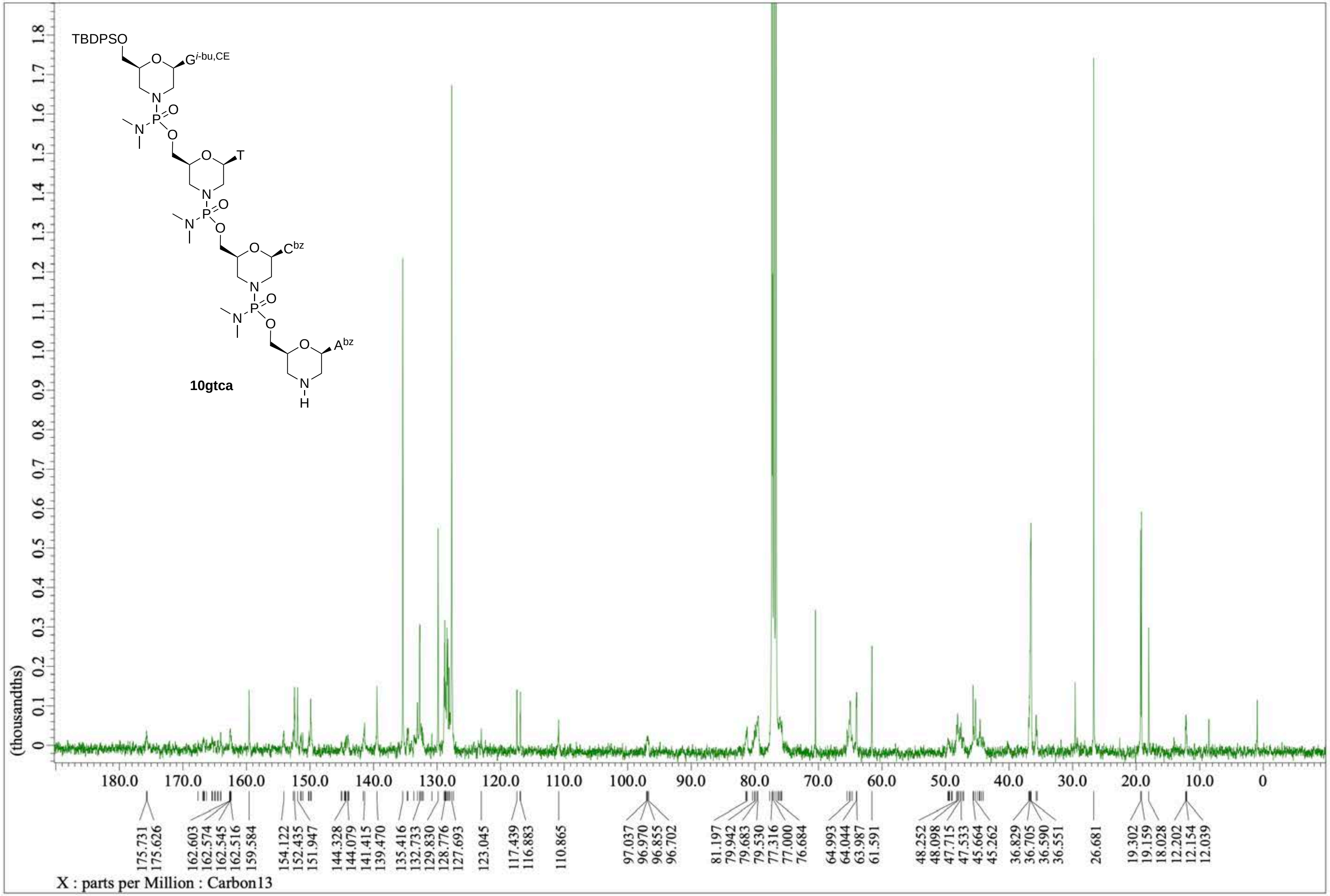

HMQC ($CDCl_3$)

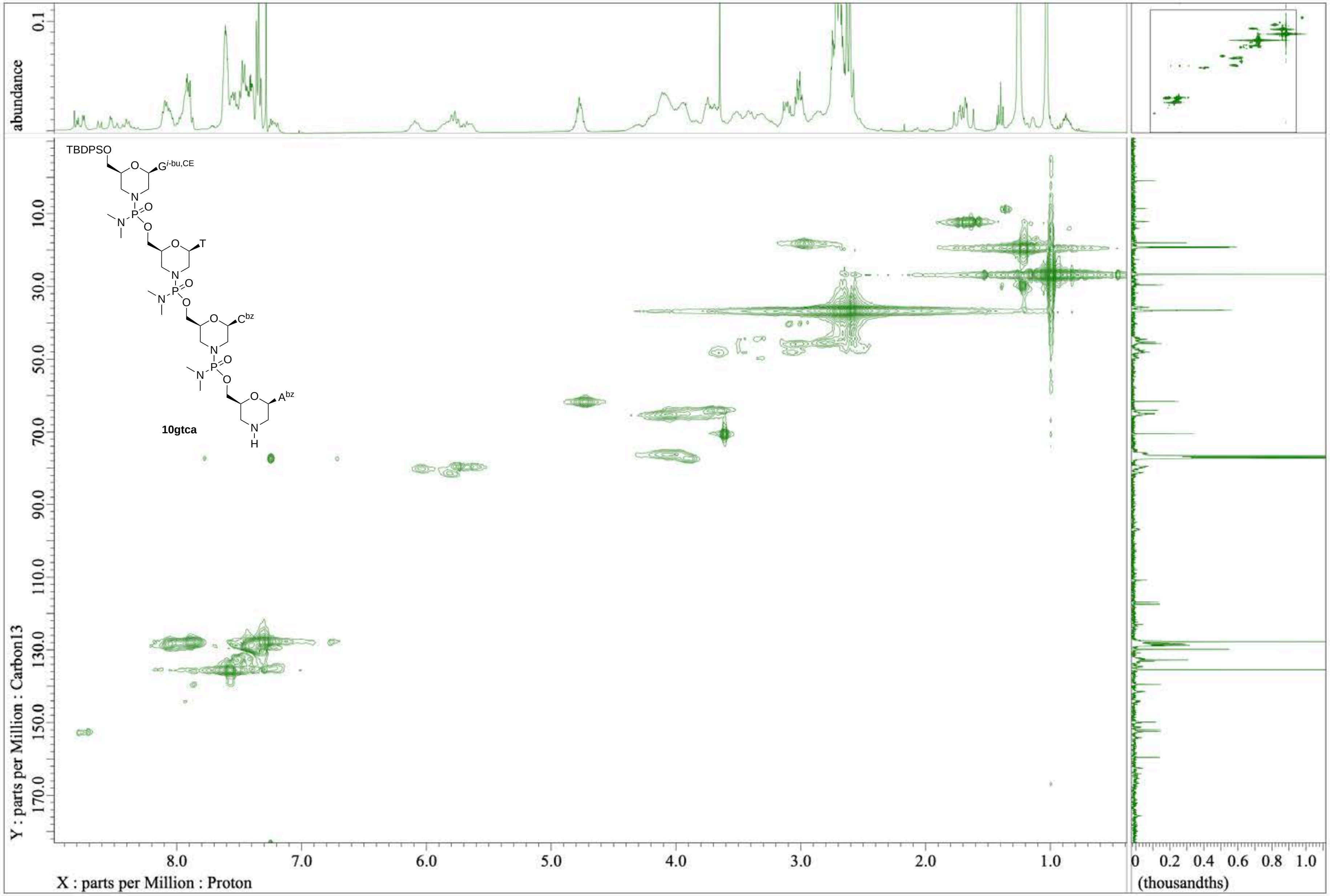
TBDPSO
G^{i-bu,CE}
T
C^{bz}
A^{bz}
10gtca
abundance
0.1
X : parts per Million : Proton
Y : parts per Million : Carbon13
(thousandths)

HMBC (CDCl₃)

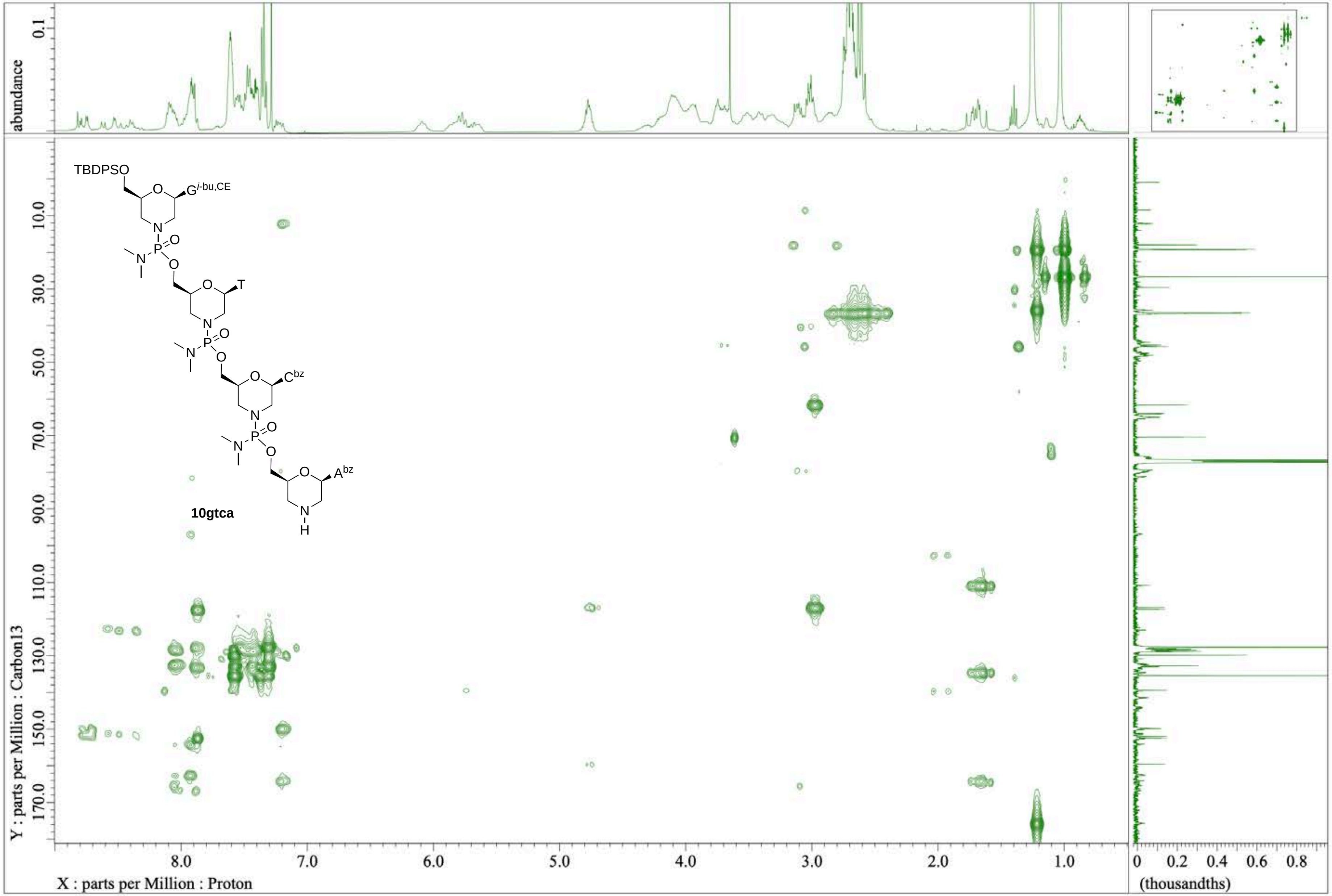
abundance
0.1
TBDPSO
$G^{i\text{-bu,CE}}$
T
C^{bz}
A^{bz}
10gtca
Y : parts per Million : Carbon13
X : parts per Million : Proton
(thousandths)

$^{31}P\{^1H\}$ NMR (162 MHz, $CDCl_3$)

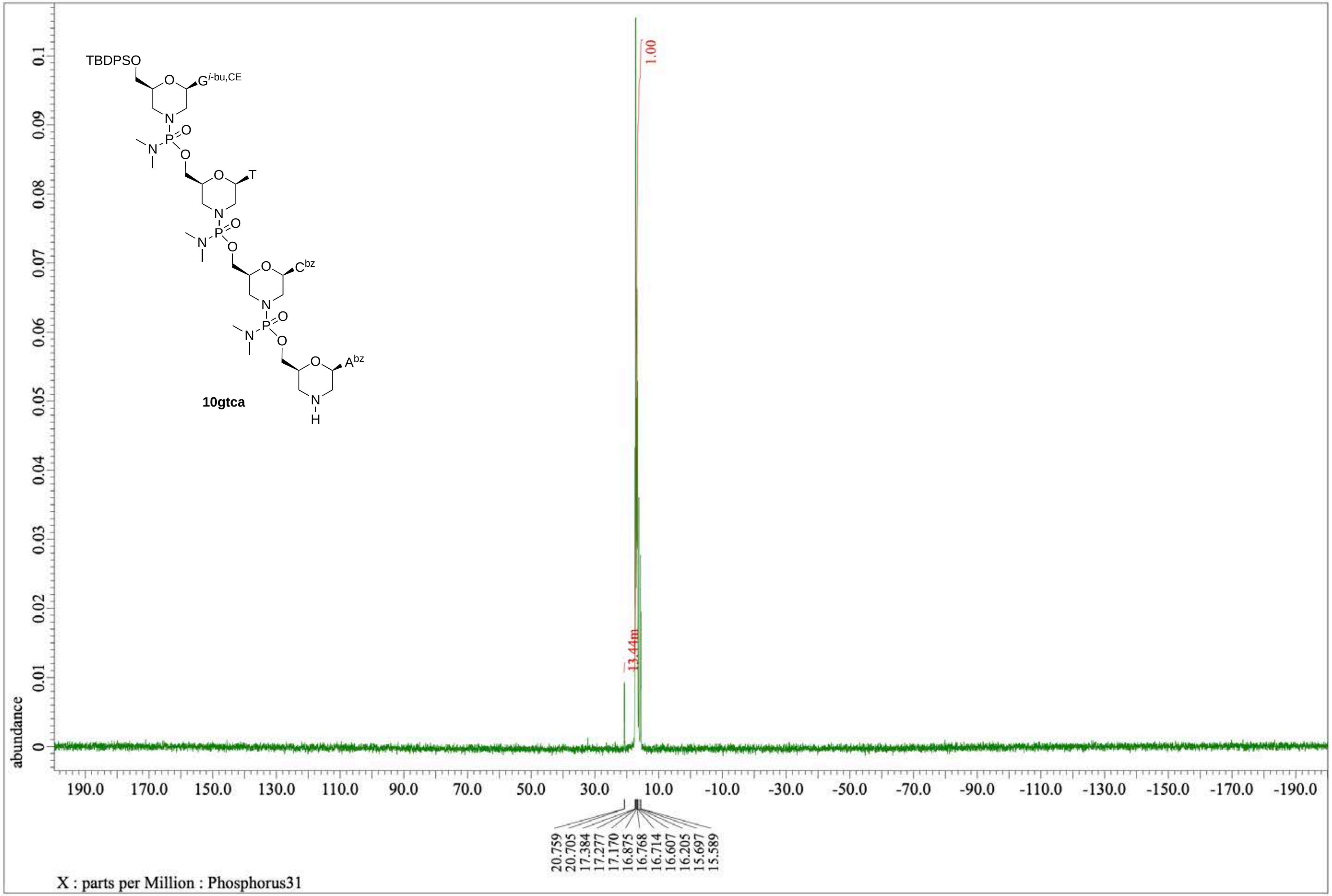

Mass spectra

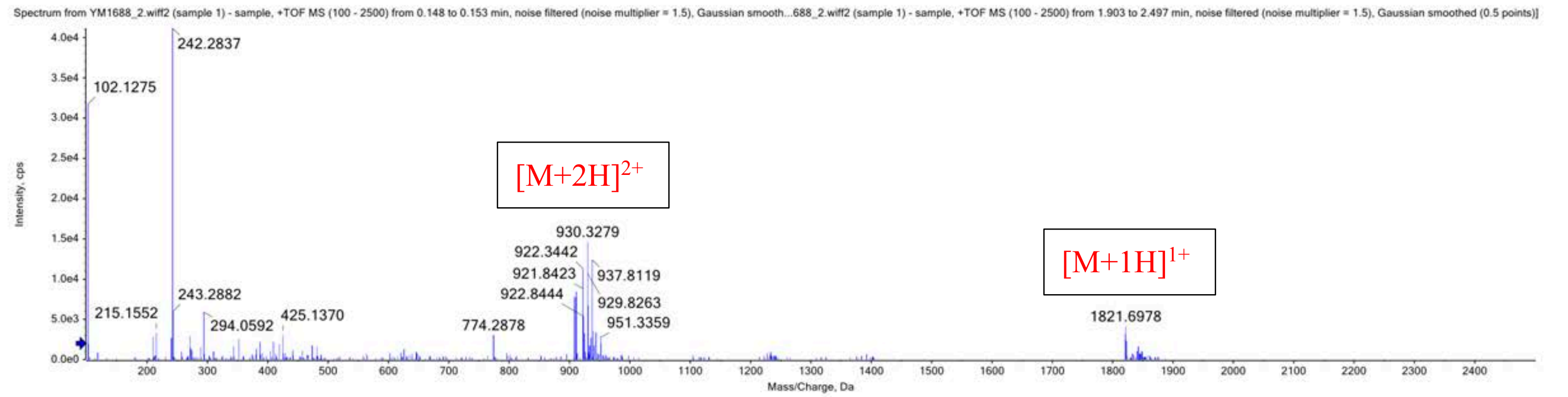

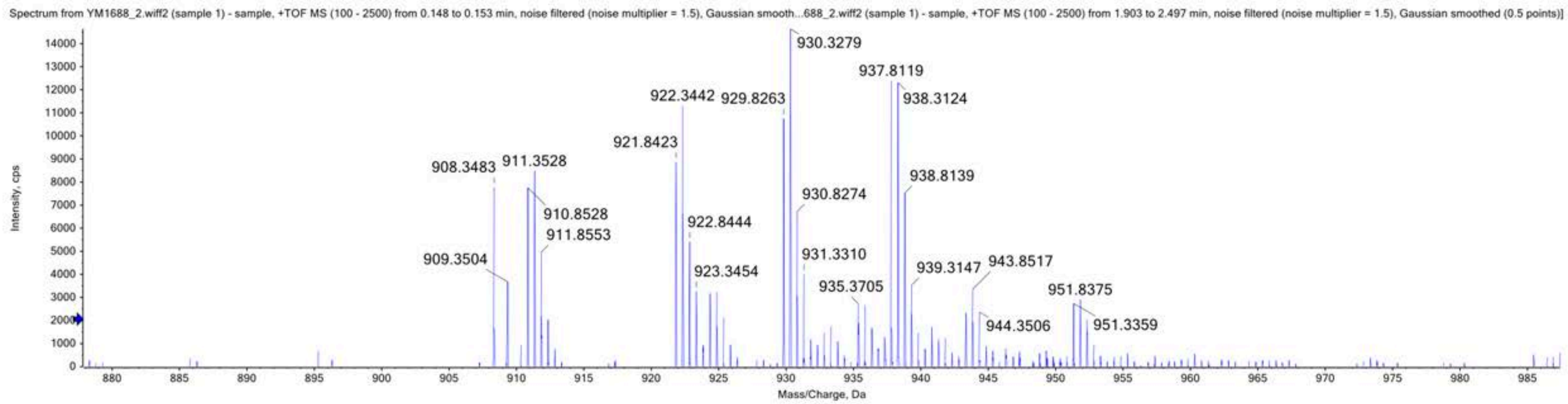

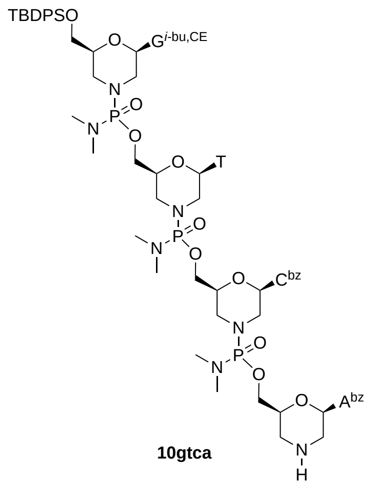

10gtca

ESI-MS *m/z* calcd for $C_{82}H_{106}N_{23}O_{18}P_3Si$ $[M+2H]^{2+}$, 910.8529; found 910.8528.

^{1}H-NMR (400 MHz, $CDCl_3$)

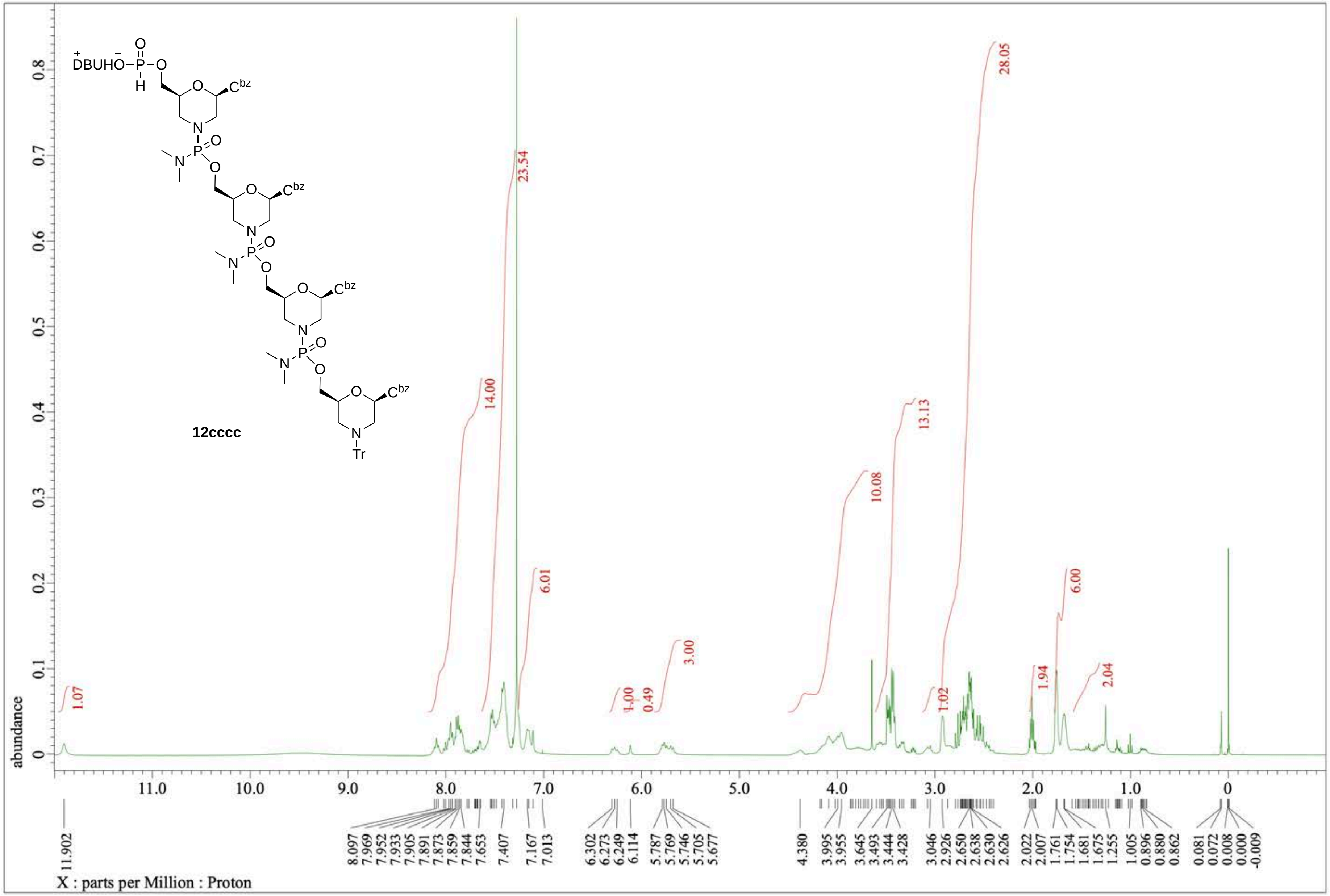

^{13}C{H}-NMR (101 MHz, $CDCl_3$)

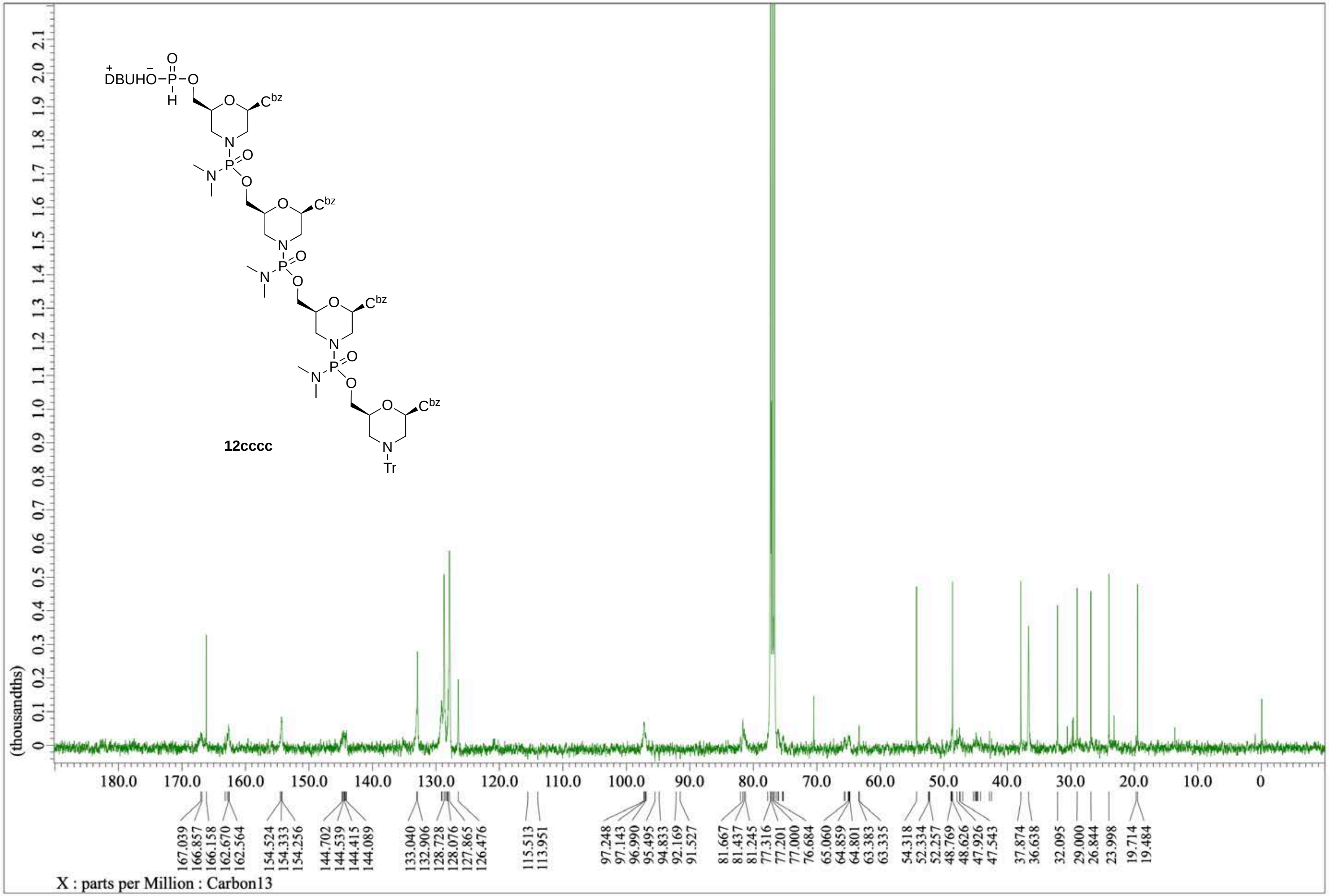

HMQC ($CDCl_3$)

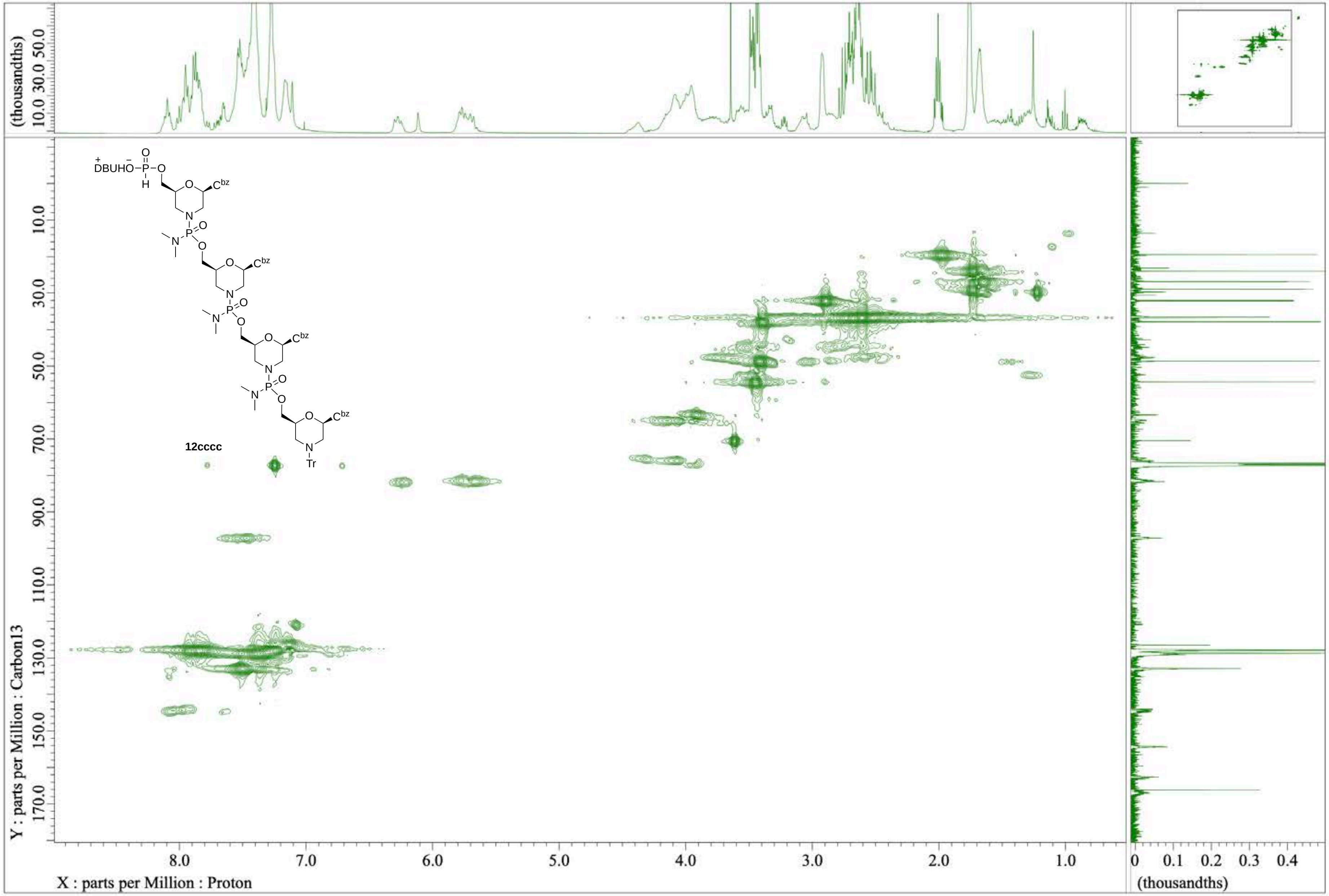

12cccc
Y : parts per Million : Carbon13
X : parts per Million : Proton
(thousandths)

HMBC ($CDCl_3$)

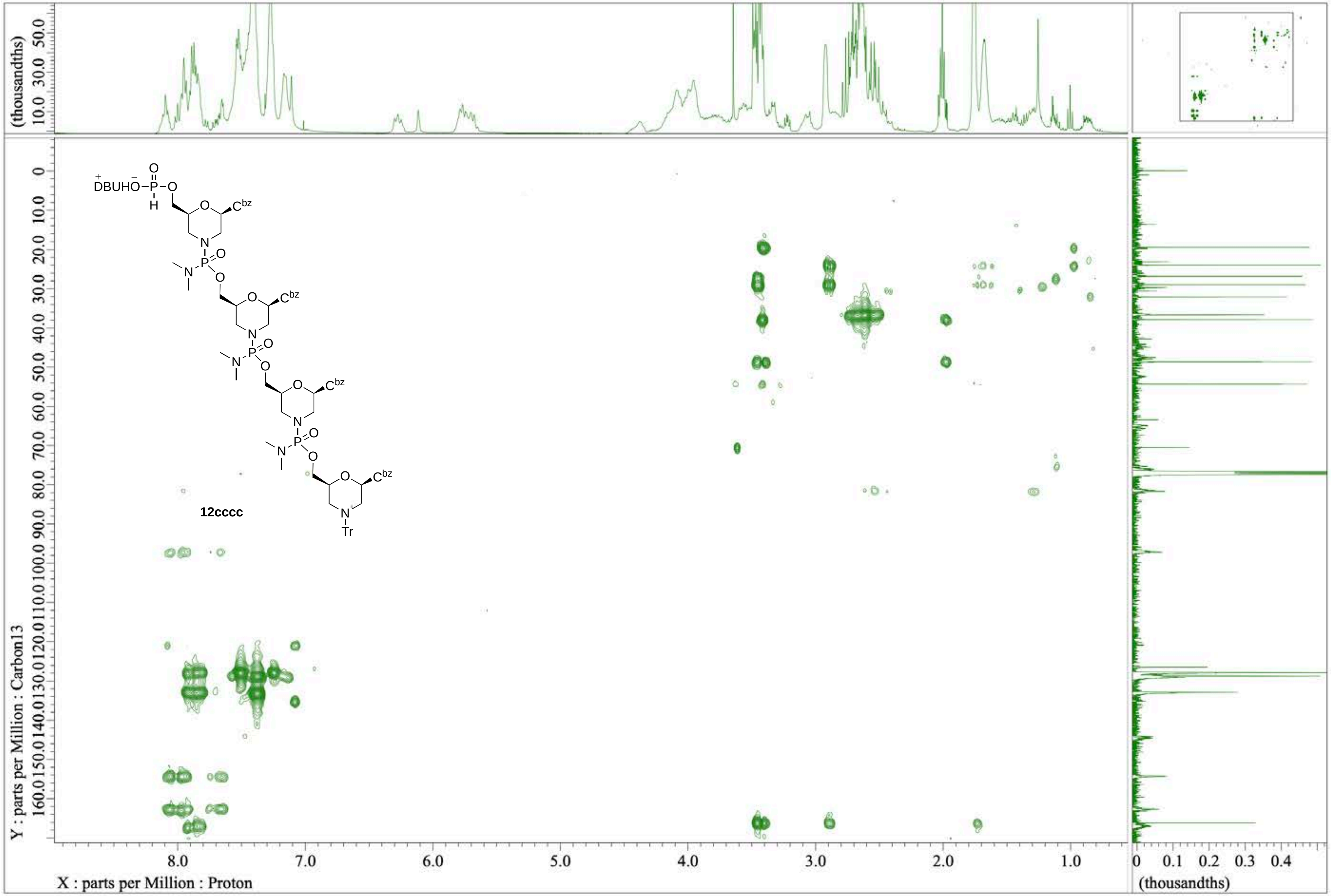
12cccc
(thousandths)
Y : parts per Million : Carbon13
X : parts per Million : Proton
(thousandths)

$^{31}P\{^1H\}$ NMR (162 MHz, $CDCl_3$)

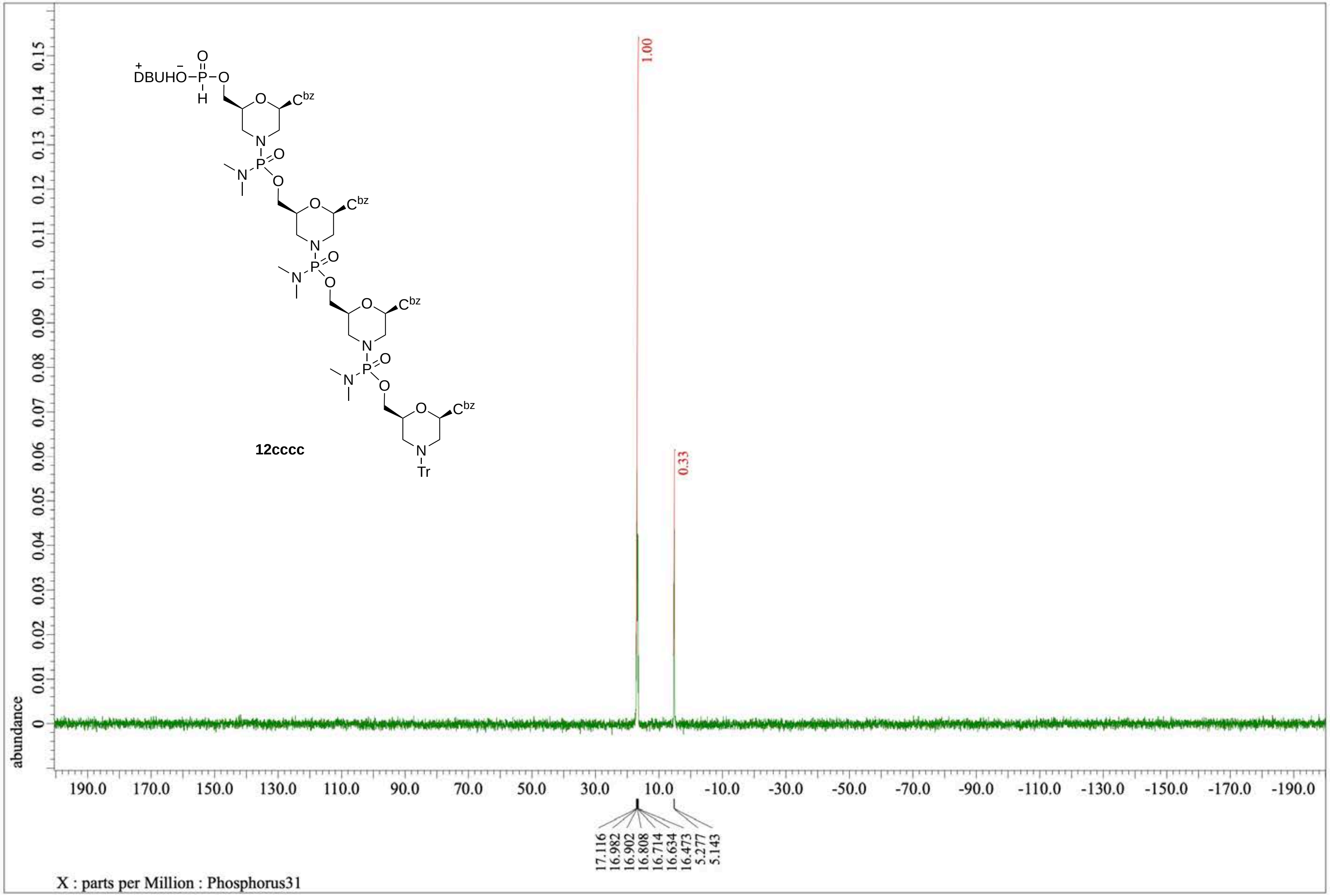

Mass spectra

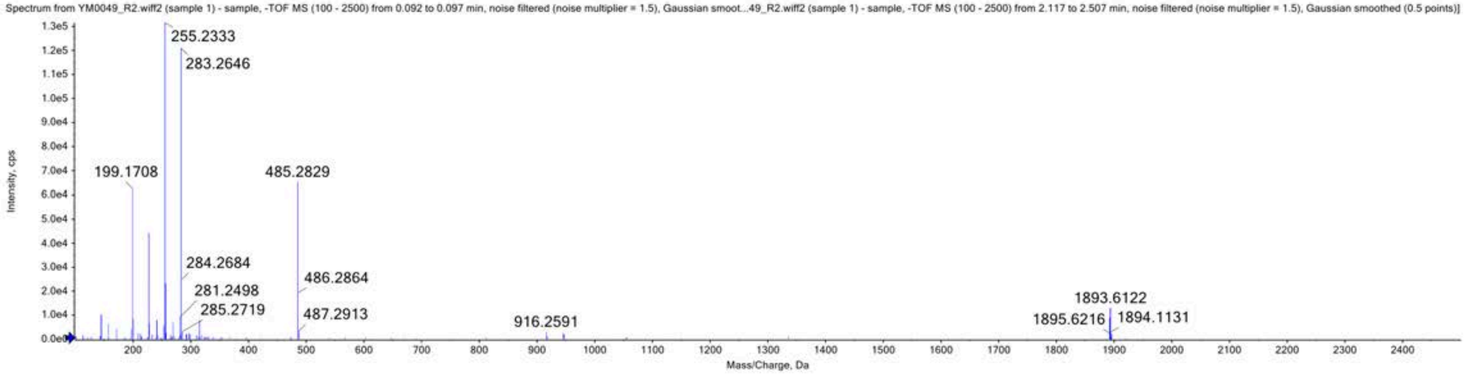

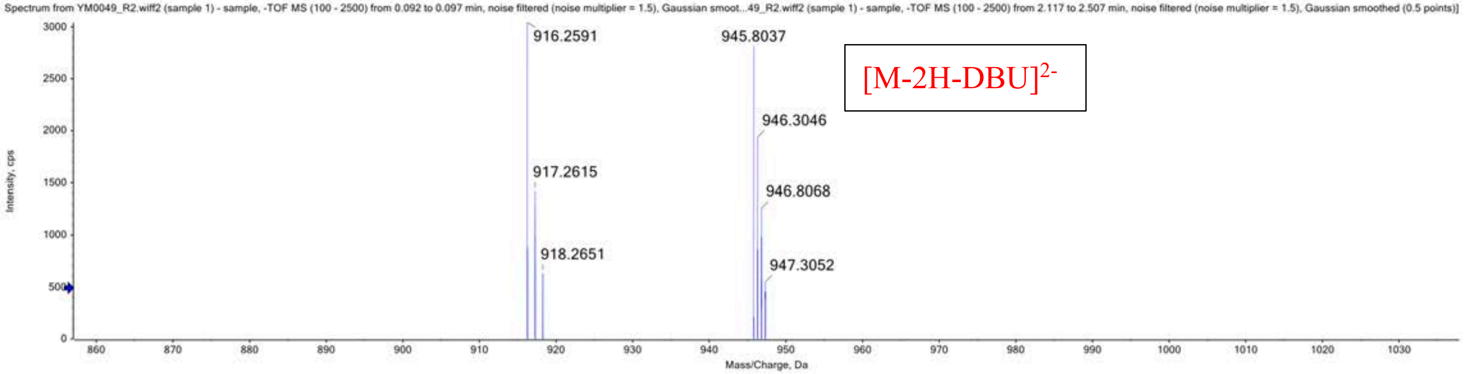

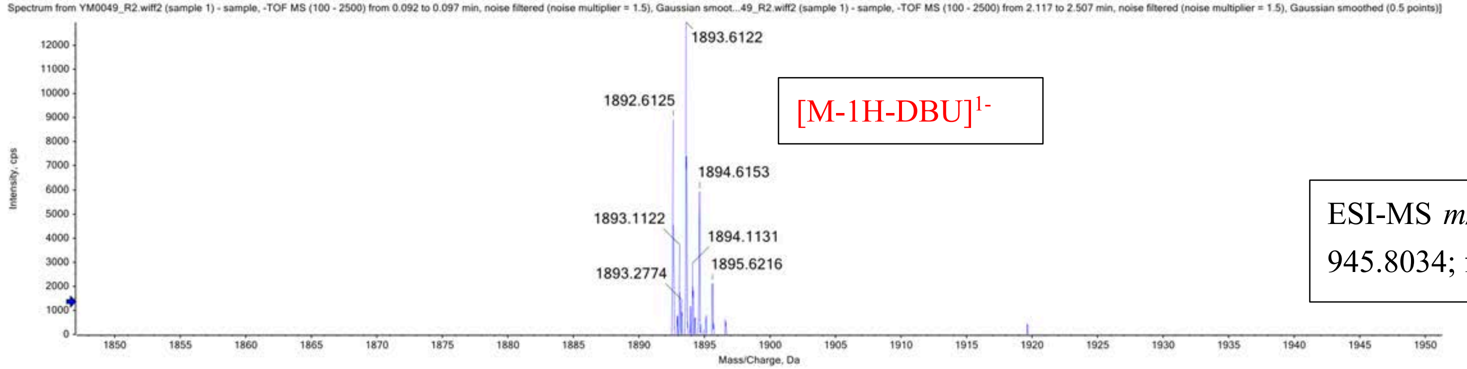

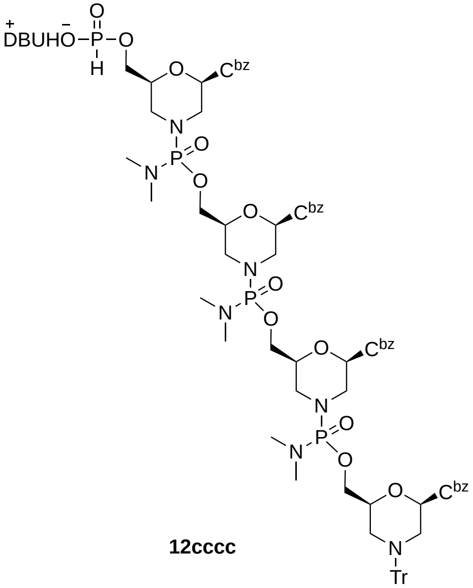

12cccc

ESI-MS *m/z* calcd for $C_{89}H_{97}N_{19}O_{21}P_4$ $[M-2H-DBU]^{2-}$, 945.8034; found 945.8037.

^{1}H-NMR (400 MHz, $CDCl_3$)

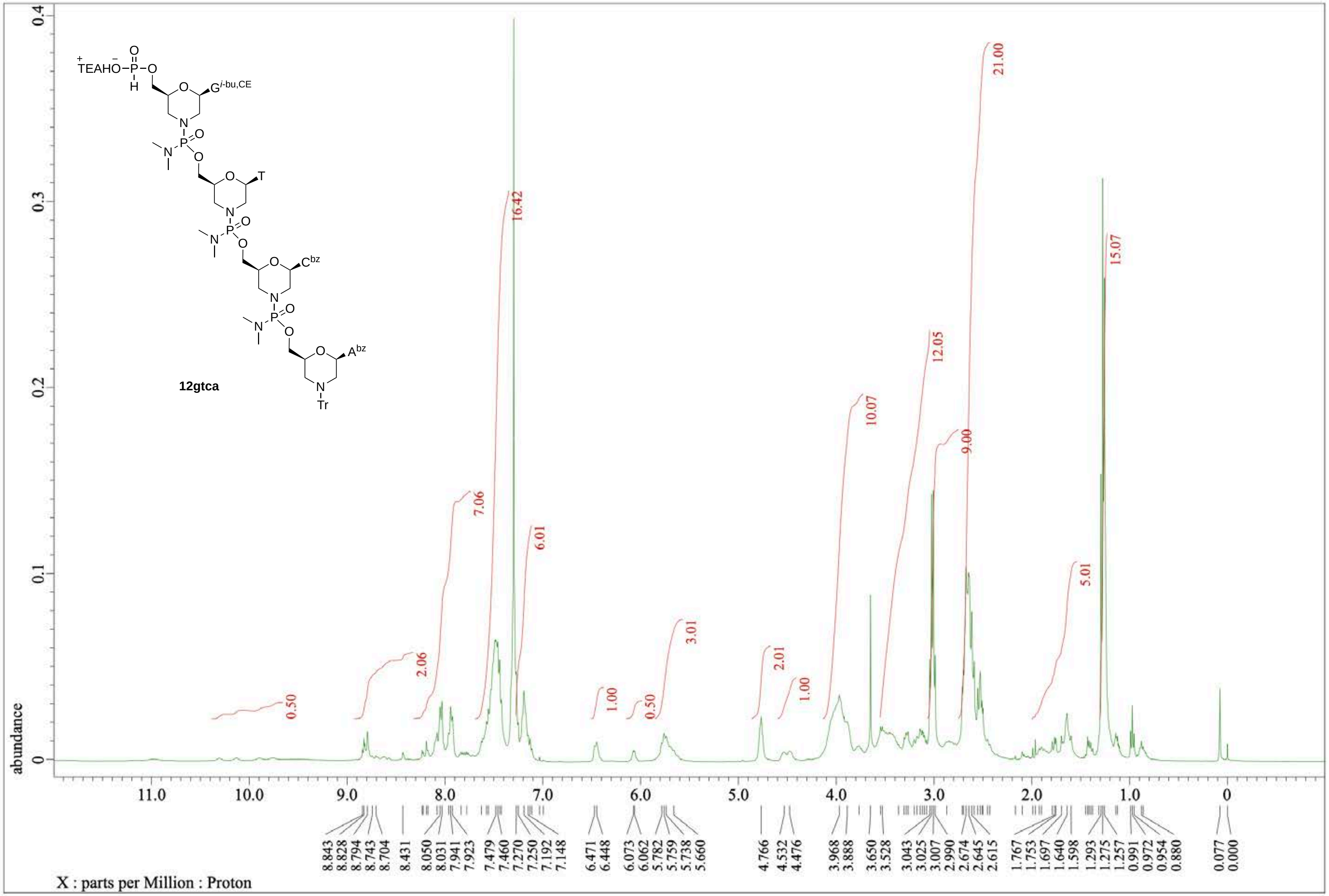

$^{13}C\{H\}$-NMR (101 MHz, $CDCl_3$)

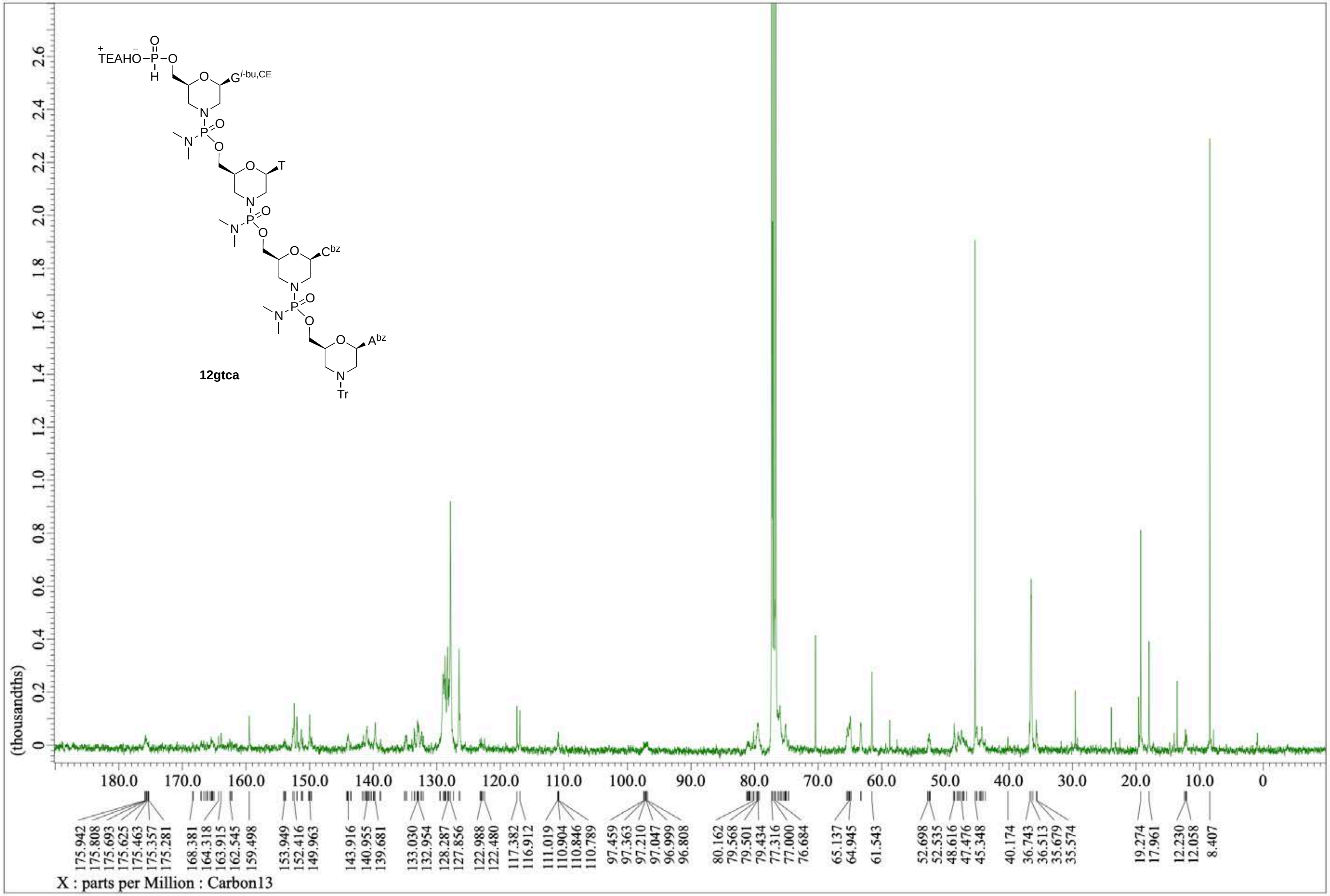

HMQC ($CDCl_3$)

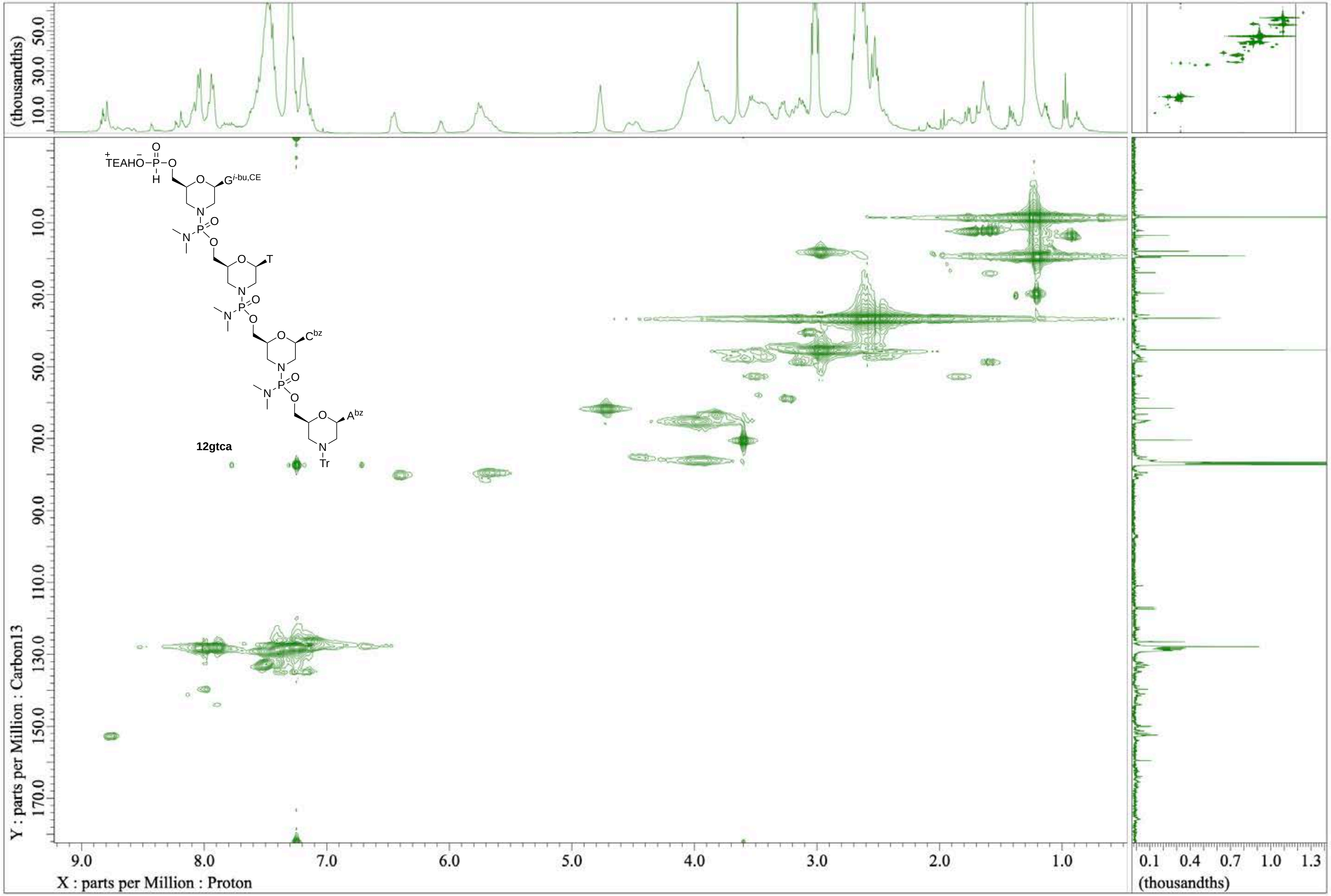
12gtca
(thousandths)
10.0
30.0
50.0
70.0
90.0
110.0
130.0
150.0
170.0
Y : parts per Million : Carbon13
9.0
8.0
7.0
6.0
5.0
4.0
3.0
2.0
1.0
X : parts per Million : Proton
0.1
0.4
0.7
1.0
1.3
(thousandths)

HMBC (CDCl3)

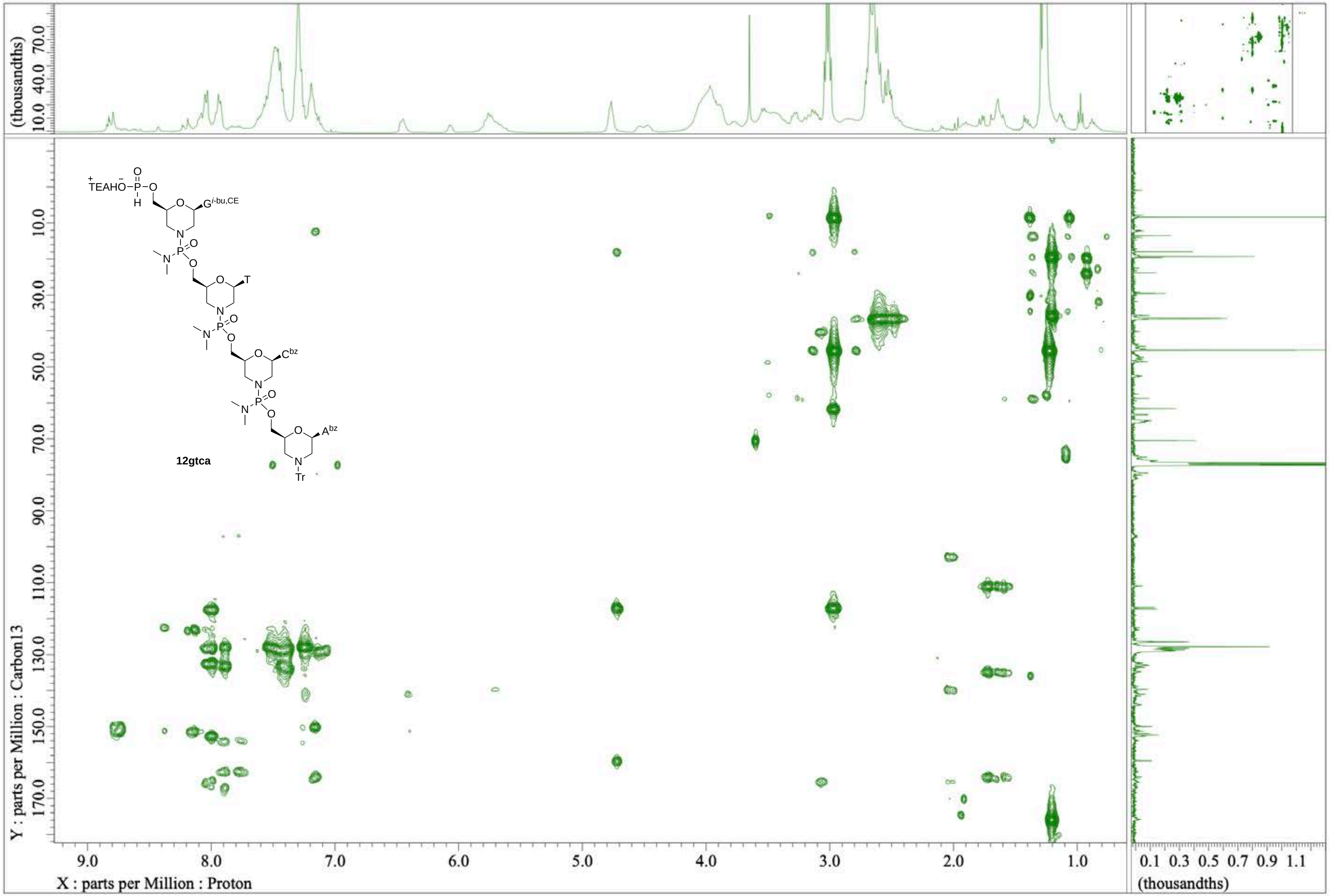

12gtca
Y : parts per Million : Carbon13
X : parts per Million : Proton
(thousandths)

$^{31}P\{^1H\}$ NMR (162 MHz, $CDCl_3$)

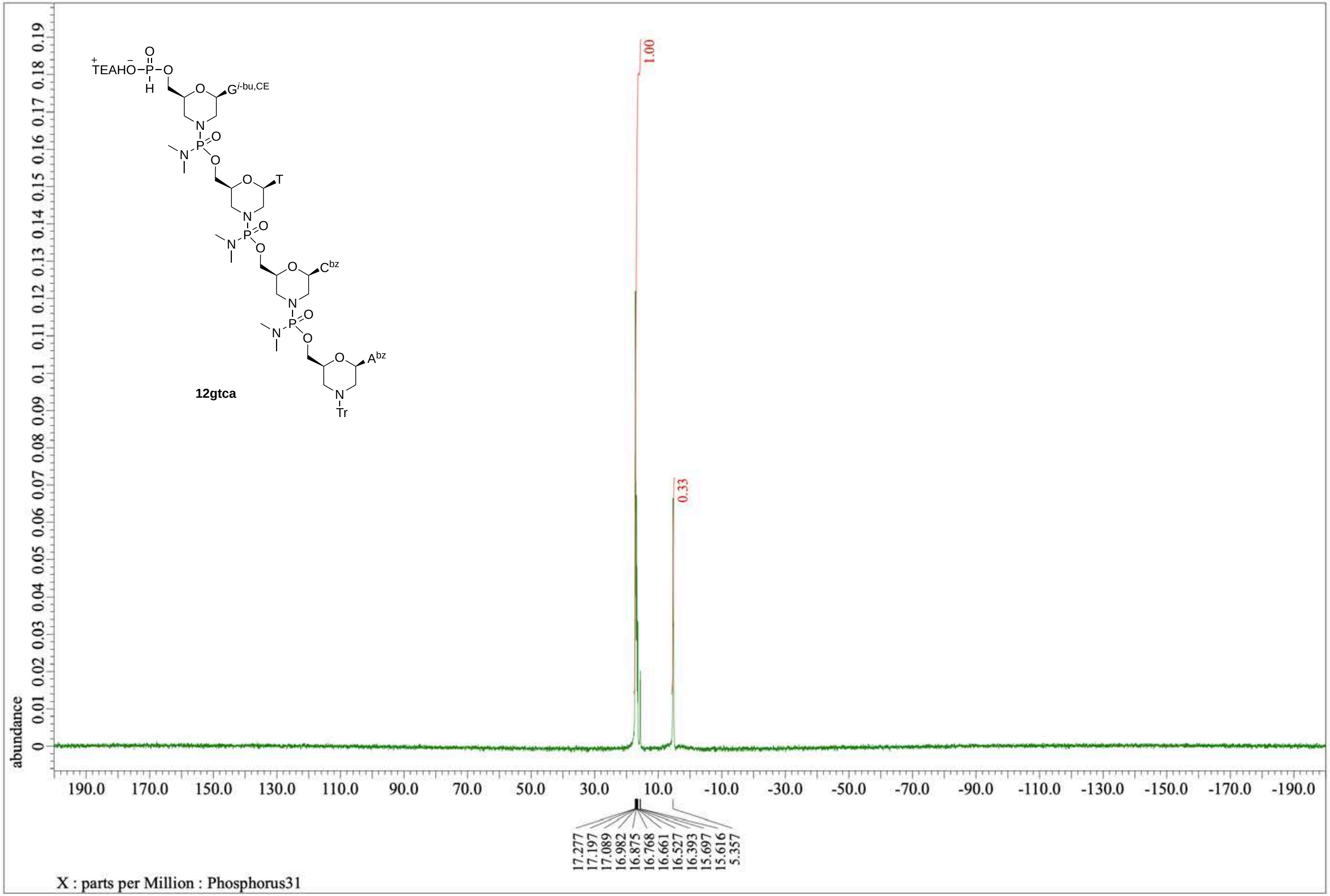

Mass spectra

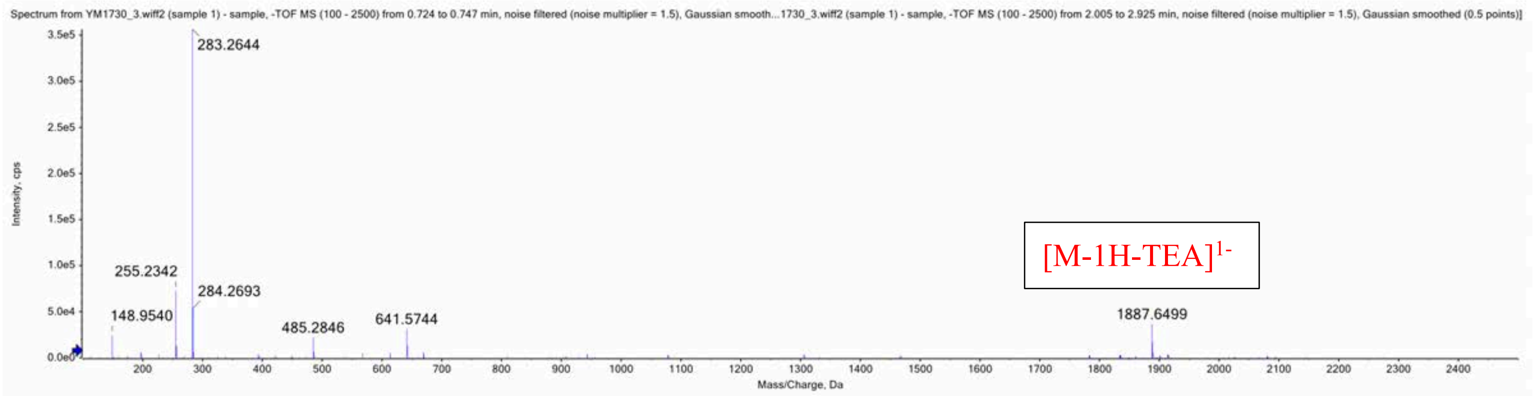

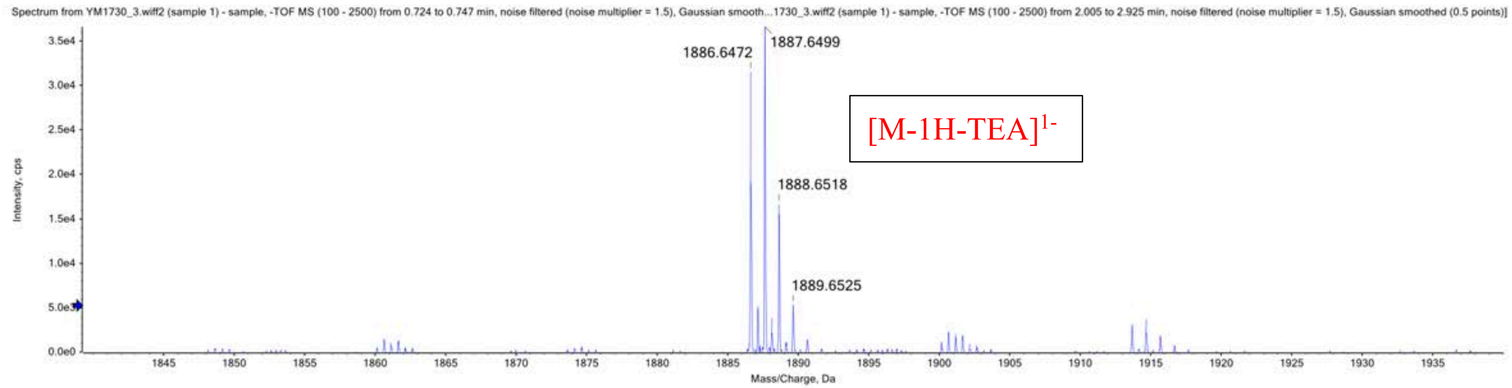

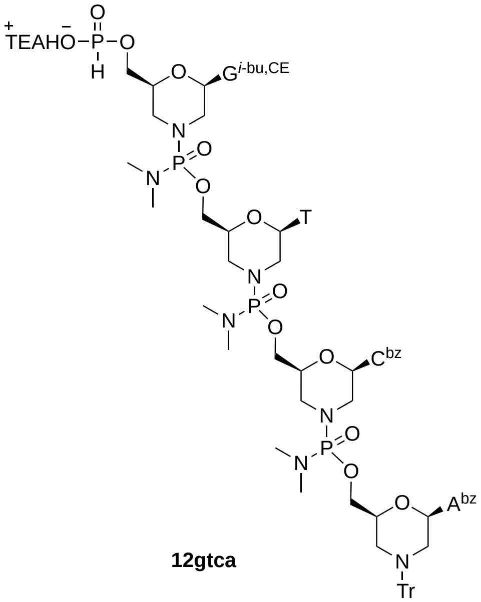

12gtca

ESI-MS *m/z* calcd for $C_{85}H_{100}N_{23}O_{20}P_4$ $[M\text{-}H\text{-}TEA]^{1-}$, 1886.6471; found 1886.6472.

^{1}H-NMR (600 MHz, D_2O)

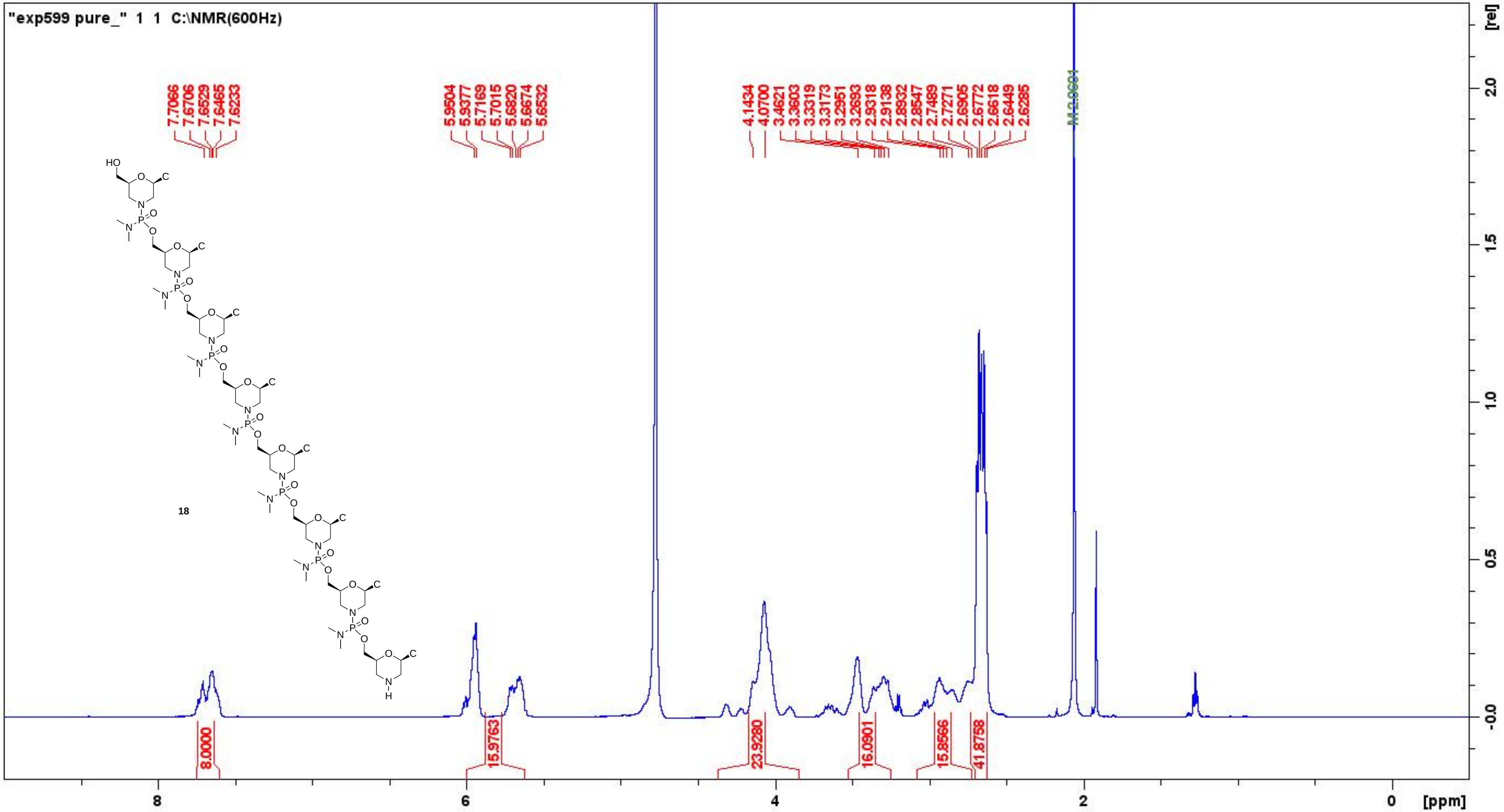

18

$^{13}C\{H\}$-NMR (151 MHz, D_2O)

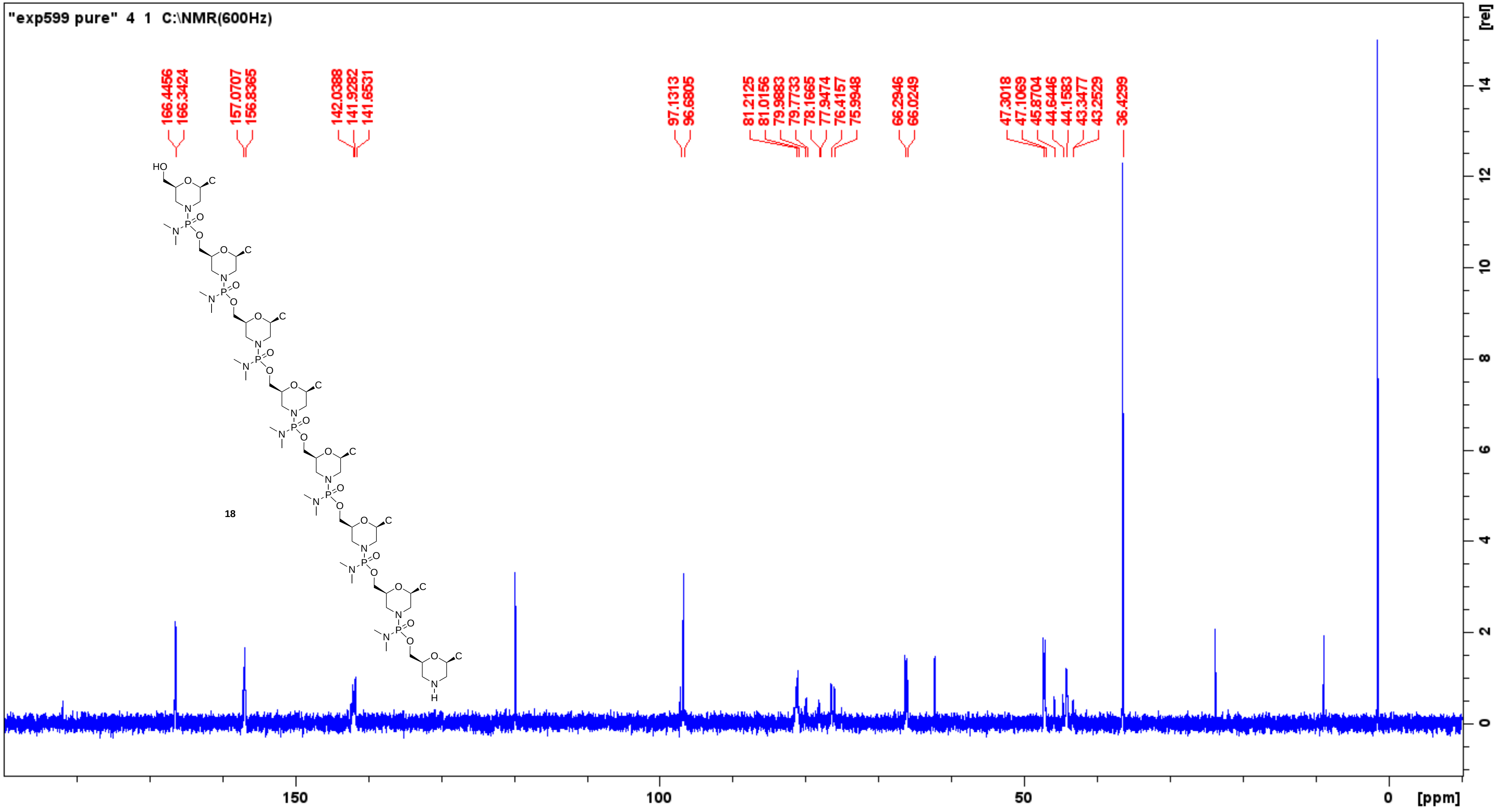

HMQC (D_2O)

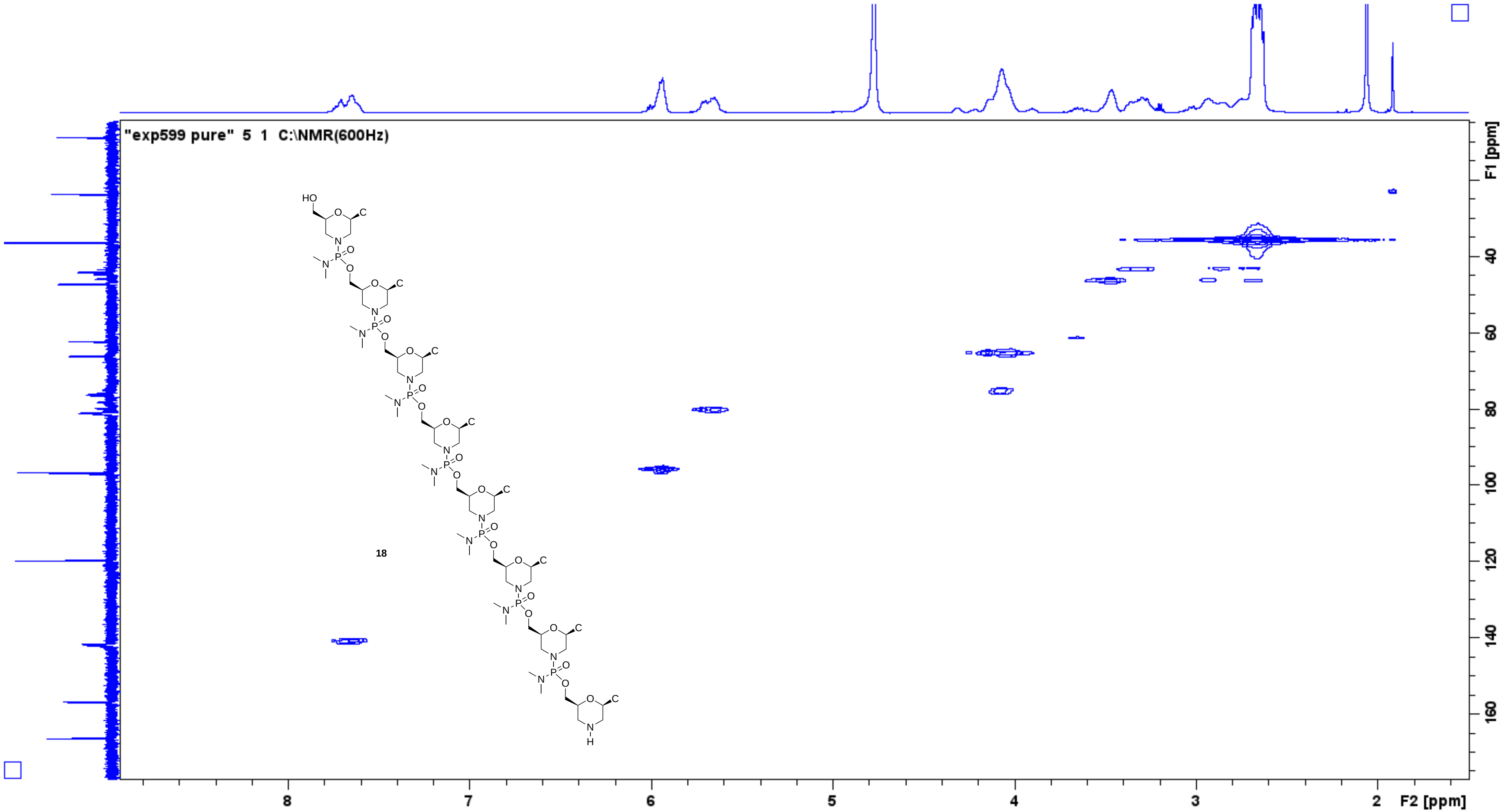

HMBC (D_2O)

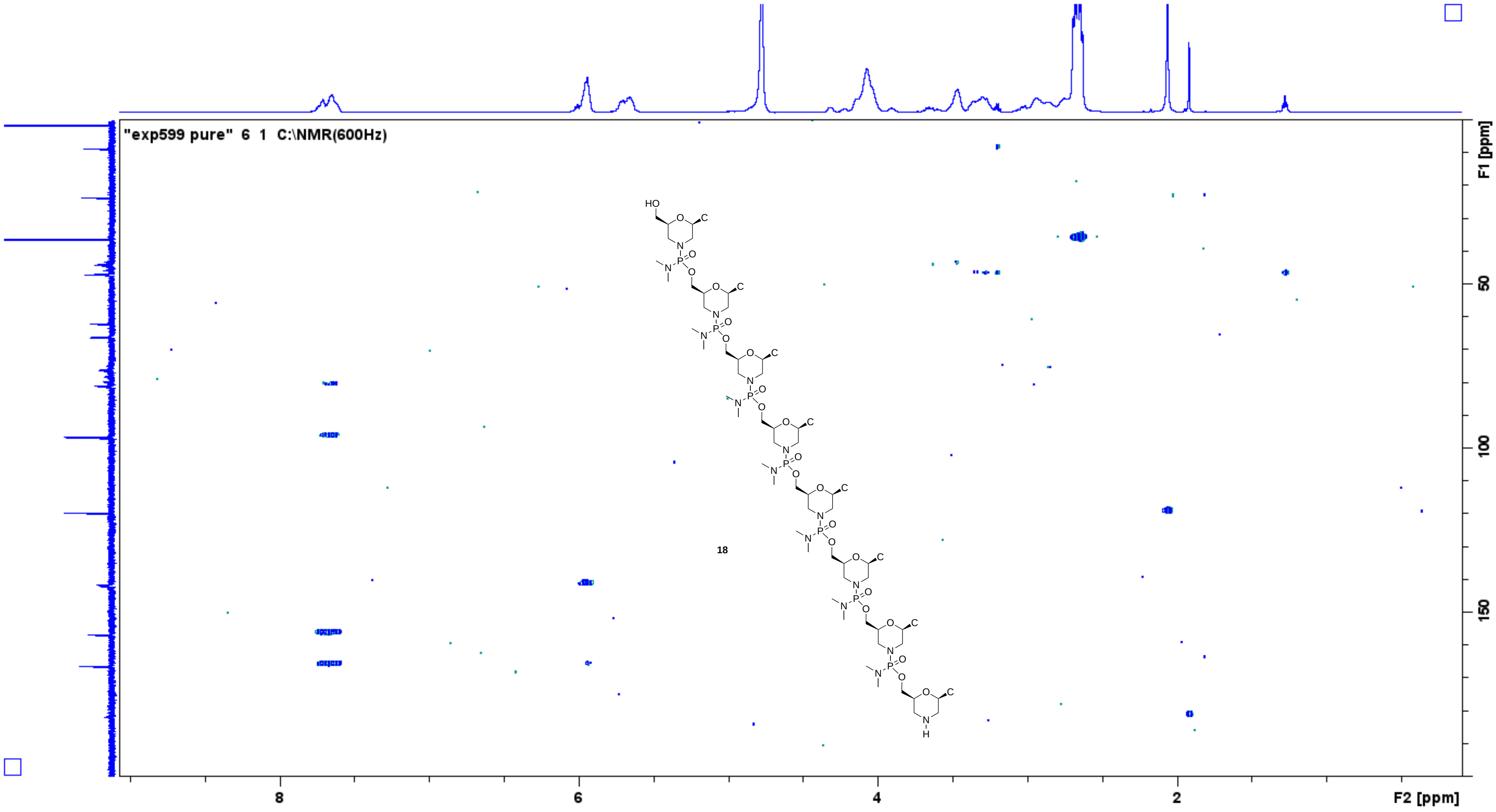

$^{31}P\{^1H\}$ NMR (243 MHz, D_2O)

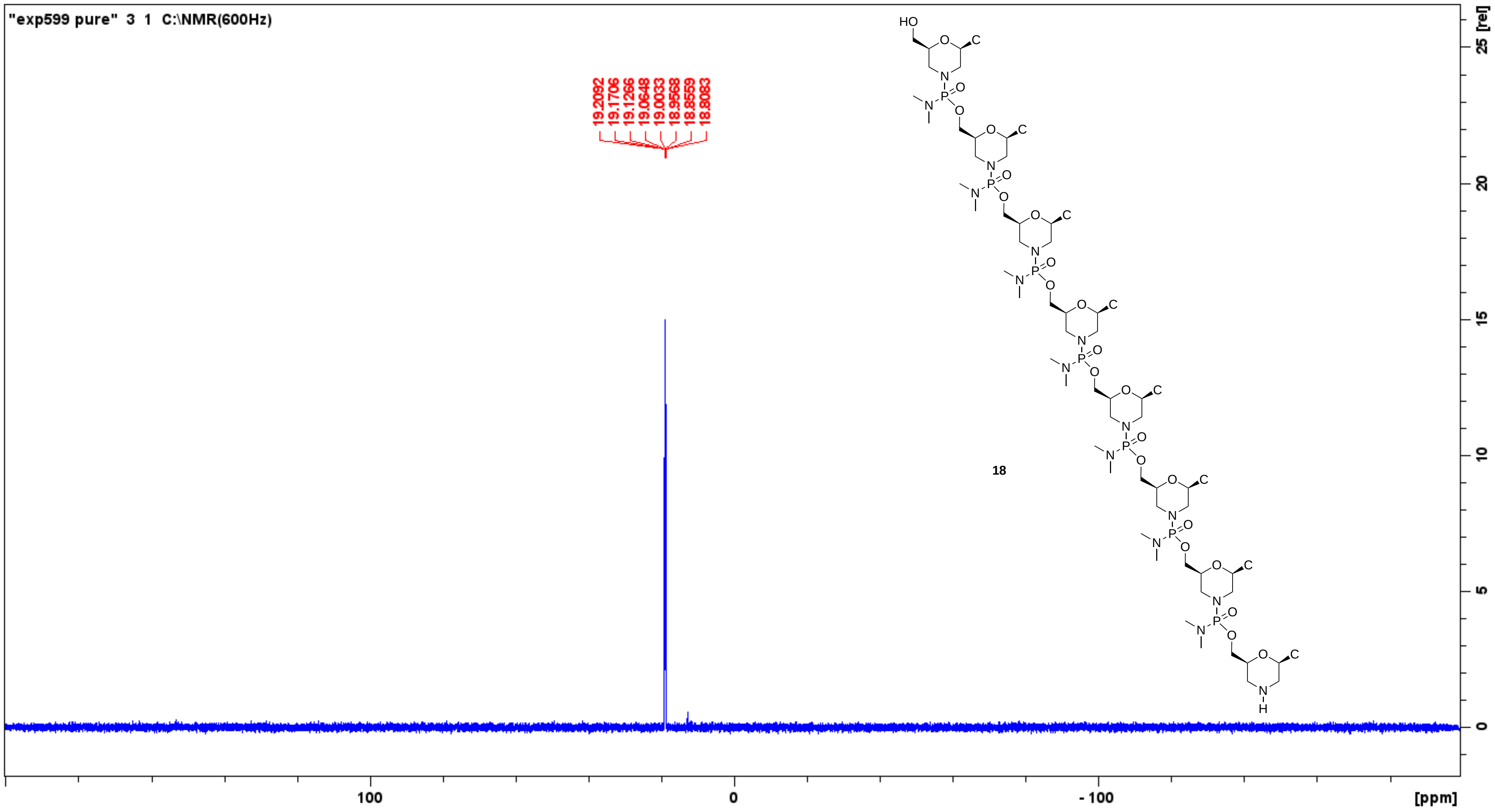

Mass spectra

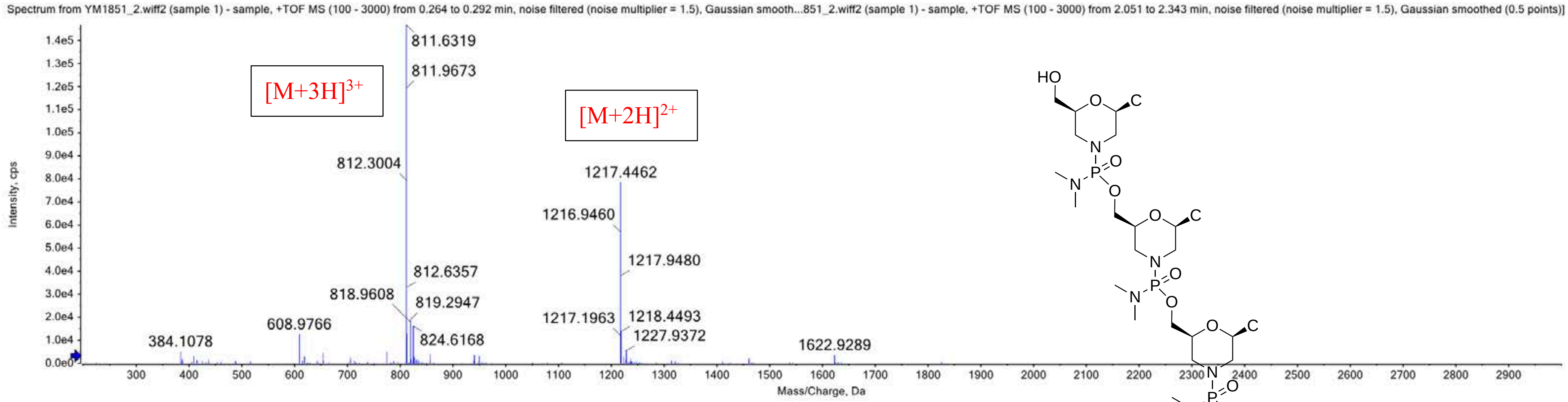

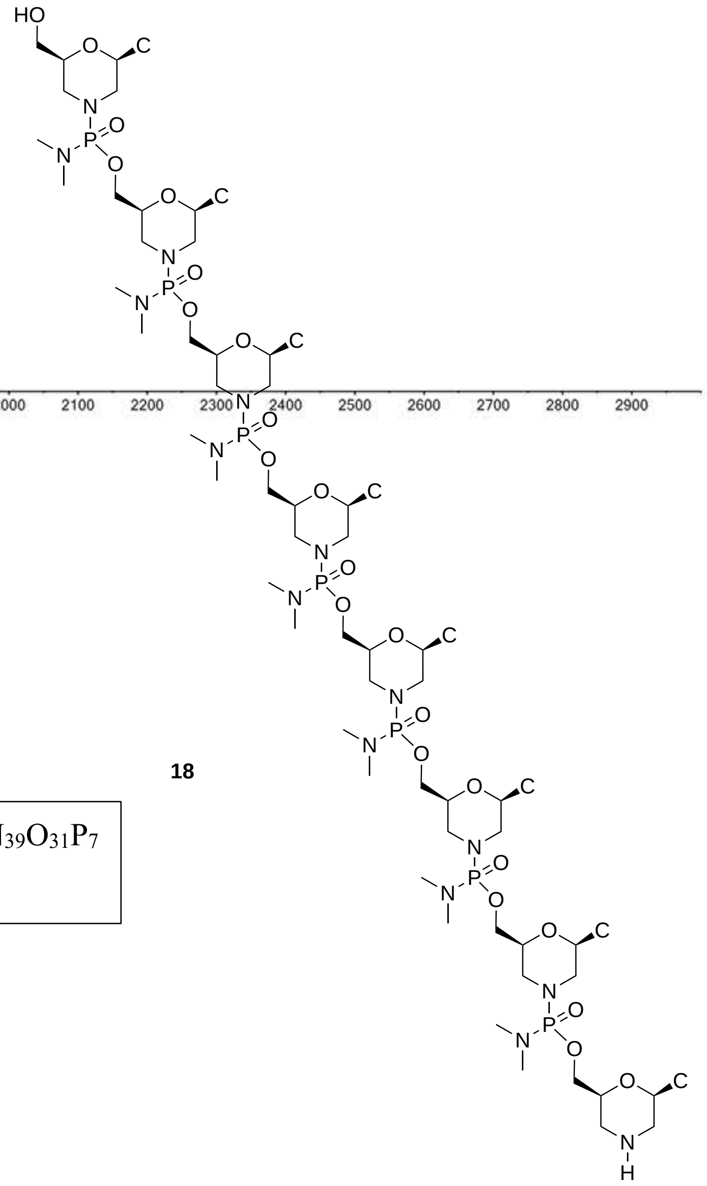

18

HRMS (ESI-TOF) *m/z* calcd for $C_{86}H_{143}N_{39}O_{31}P_7$ $[M+3H]^{3+}$, 811.6320; found 811.6319.

^{1}H-NMR (600 MHz, D_2O)

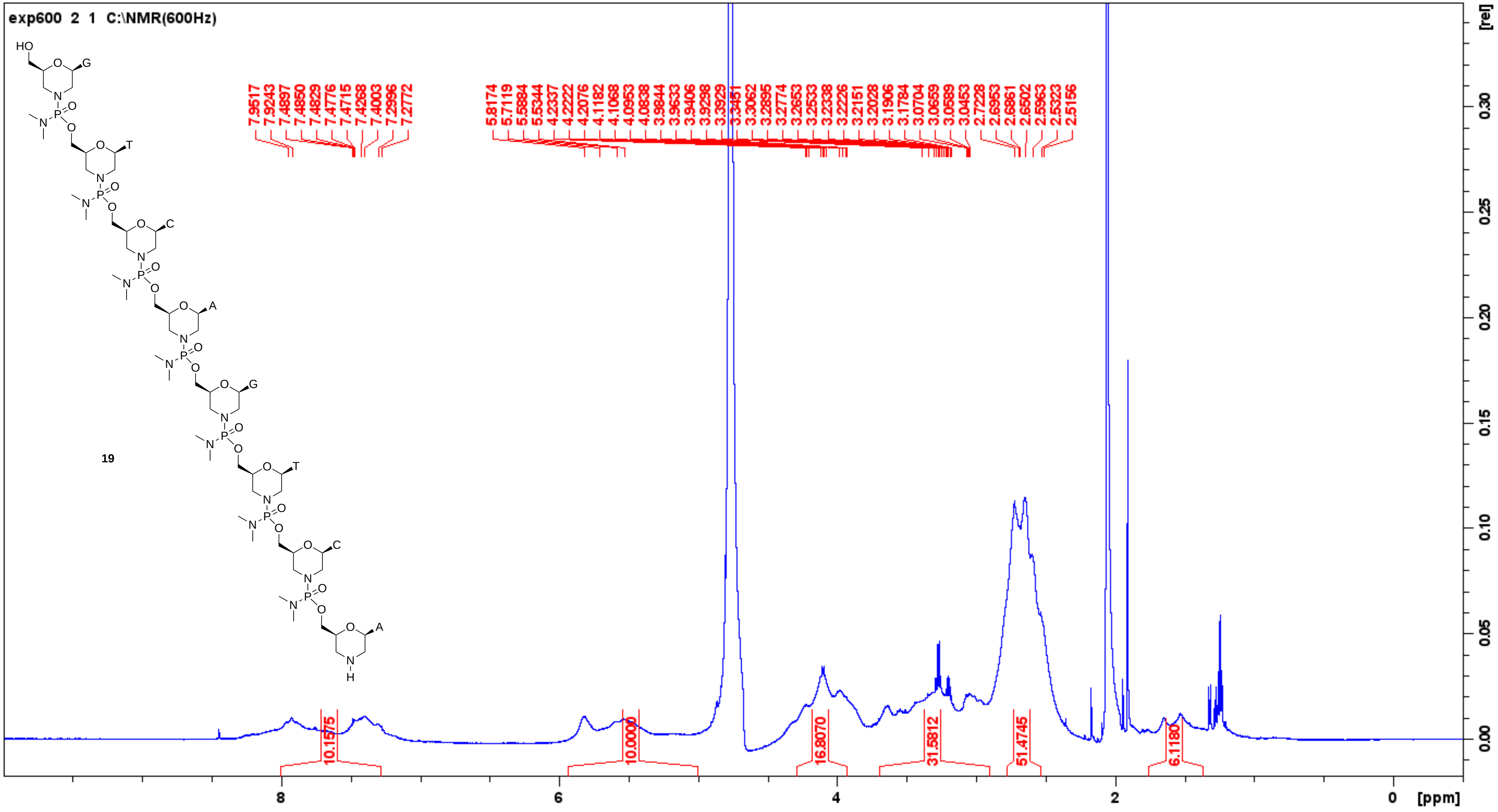

$^{31}P\{^1H\}$ NMR (243 MHz, D_2O)

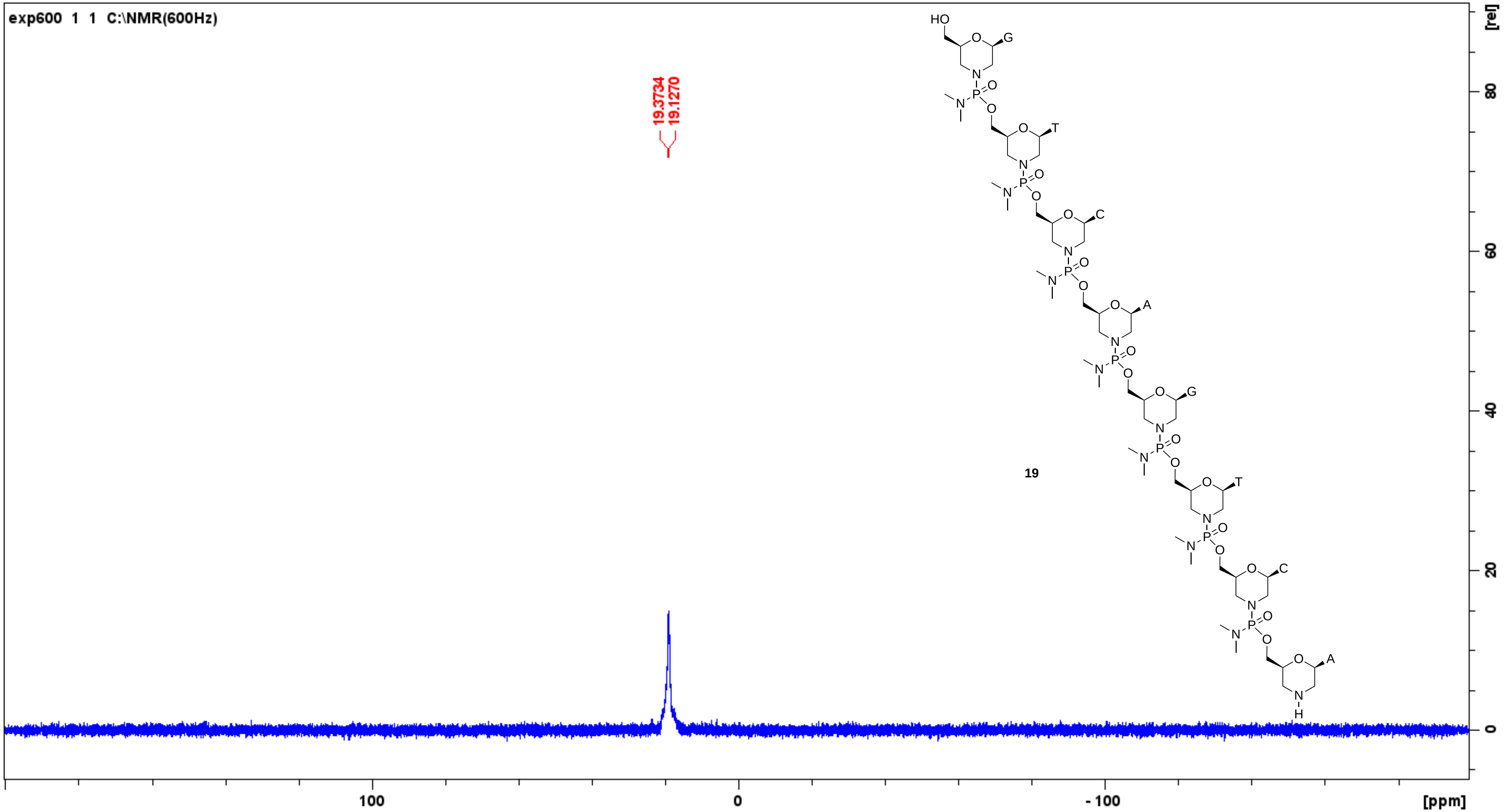

Mass spectra

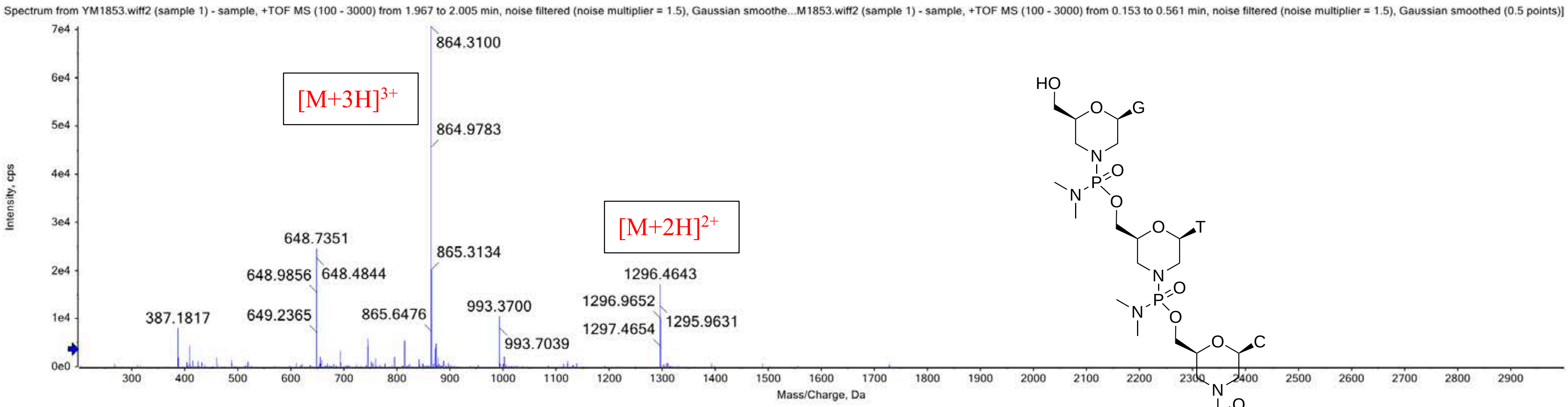

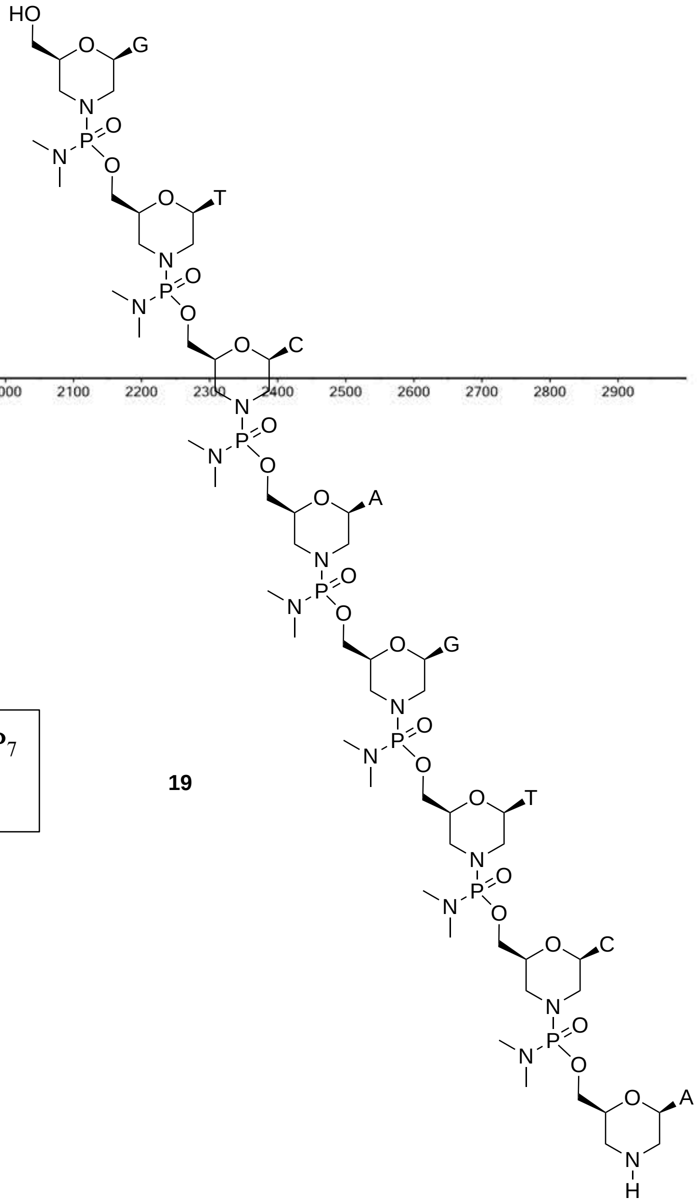

19

HRMS (ESI-TOF) *m/z* calcd for $C_{92}H_{145}N_{45}O_{31}P_7$ $[M+3H]^{3+}$, 864.3100; found 864.3100.
